# A comprehensive review of prioritised interventions to improve the health and wellbeing of persons with lived experience of homelessness

**DOI:** 10.1002/cl2.1154

**Published:** 2021-06-24

**Authors:** Aliza Moledina, Olivia Magwood, Eric Agbata, Jui‐Hsia Hung, Ammar Saad, Kednapa Thavorn, Kevin Pottie

**Affiliations:** ^1^ Faculty of Medicine University of Ottawa Ottawa Canada; ^2^ C.T. Lamont Primary Health Care Research Centre Bruyere Research Institute Ottawa Canada; ^3^ Bruyere Research Institute, School of Epidemiology Public Health and Preventive Medicine Ottawa Canada; ^4^ Faculty of Medicine, School of Epidemiology and Public Health University of Ottawa Ottawa Canada; ^5^ Department of Epidemiology, C.T. Lamont Primary Care Research Centre, Bruyere Research Institute University of Ottawa Ottawa Canada; ^6^ Clinical Epidemiology Program Ottawa Hospital Research Institute Ottawa Canada; ^7^ Family Medicine University of Ottawa Ottawa Canada

## Abstract

**Background:**

Homelessness has emerged as a public health priority, with growing numbers of vulnerable populations despite advances in social welfare. In February 2020, the United Nations passed a historic resolution, identifying the need to adopt social‐protection systems and ensure access to safe and affordable housing for all. The establishment of housing stability is a critical outcome that intersects with other social inequities. Prior research has shown that in comparison to the general population, people experiencing homelessness have higher rates of infectious diseases, chronic illnesses, and mental‐health disorders, along with disproportionately poorer outcomes. Hence, there is an urgent need to identify effective interventions to improve the lives of people living with homelessness.

**Objectives:**

The objective of this systematic review is to identify, appraise, and synthesise the best available evidence on the benefits and cost‐effectiveness of interventions to improve the health and social outcomes of people experiencing homelessness.

**Search Methods:**

In consultation with an information scientist, we searched nine bibliographic databases, including Medline, EMBASE, and Cochrane CENTRAL, from database inception to February 10, 2020 using keywords and MeSH terms. We conducted a focused grey literature search and consulted experts for additional studies.

**Selection Criteria:**

Teams of two reviewers independently screened studies against our inclusion criteria. We included randomised control trials (RCTs) and quasi‐experimental studies conducted among populations experiencing homelessness in high‐income countries. Eligible interventions included permanent supportive housing (PSH), income assistance, standard case management (SCM), peer support, mental health interventions such as assertive community treatment (ACT), intensive case management (ICM), critical time intervention (CTI) and injectable antipsychotics, and substance‐use interventions, including supervised consumption facilities (SCFs), managed alcohol programmes and opioid agonist therapy. Outcomes of interest were housing stability, mental health, quality of life, substance use, hospitalisations, employment and income.

**Data Collection and Analysis:**

Teams of two reviewers extracted data in duplicate and independently. We assessed risk of bias using the Cochrane Risk of Bias tool. We performed our statistical analyses using RevMan 5.3. For dichotomous data, we used odds ratios and risk ratios with 95% confidence intervals. For continuous data, we used the mean difference (MD) with a 95% CI if the outcomes were measured in the same way between trials. We used the standardised mean difference with a 95% CI to combine trials that measured the same outcome but used different methods of measurement. Whenever possible, we pooled effect estimates using a random‐effects model.

**Main Results:**

The search resulted in 15,889 citations. We included 86 studies (128 citations) that examined the effectiveness and/or cost‐effectiveness of interventions for people with lived experience of homelessness. Studies were conducted in the United States (73), Canada (8), United Kingdom (2), the Netherlands (2) and Australia (1). The studies were of low to moderate certainty, with several concerns regarding the risk of bias. PSH was found to have significant benefits on housing stability as compared to usual care. These benefits impacted both high‐ and moderate‐needs populations with significant cimorbid mental illness and substance‐use disorders. PSH may also reduce emergency department visits and days spent hospitalised. Most studies found no significant benefit of PSH on mental‐health or substance‐use outcomes. The effect on quality of life was also mixed and unclear. In one study, PSH resulted in lower odds of obtaining employment. The effect on income showed no significant differences. Income assistance appeared to have some benefits in improving housing stability, particularly in the form of rental subsidies. Although short‐term improvement in depression and perceived stress levels were reported, no evidence of the long‐term effect on mental health measures was found. No consistent impact on the outcomes of quality of life, substance use, hospitalisations, employment status, or earned income could be detected when compared with usual services. SCM interventions may have a small beneficial effect on housing stability, though results were mixed. Results for peer support interventions were also mixed, though no benefit was noted in housing stability specifically. Mental health interventions (ICM, ACT, CTI) appeared to reduce the number of days homeless and had varied effects on psychiatric symptoms, quality of life, and substance use over time. Cost analyses of PSH interventions reported mixed results. Seven studies showed that PSH interventions were associated with increased cost to payers and that the cost of the interventions were only partially offset by savings in medical‐ and social‐services costs. Six studies revealed that PSH interventions saved the payers money. Two studies focused on the cost‐effectiveness of income‐assistance interventions. For each additional day housed, clients who received income assistance incurred additional costs of US$45 (95% CI, −$19, −$108) from the societal perspective. In addition, the benefits gained from temporary financial assistance were found to outweigh the costs, with a net savings of US$20,548. The economic implications of case management interventions (SCM, ICM, ACT, CTI) was highly uncertain. SCM clients were found to incur higher costs than those receiving the usual care. For ICM, all included studies suggested that the intervention may be cost‐offset or cost‐effective. Regarding ACT, included studies consistently revealed that ACT saved payers money and improved health outcomes than usual care. Despite having comparable costs (US$52,574 vs. US$51,749), CTI led to greater nonhomeless nights (508 vs. 450 nights) compared to usual services.

**Authors' Conclusions:**

PSH interventions improved housing stability for people living with homelessness. High‐intensity case management and income‐assistance interventions may also benefit housing stability. The majority of included interventions inconsistently detected benefits for mental health, quality of life, substance use, employment and income. These results have important implications for public health, social policy, and community programme implementation. The COVID‐19 pandemic has highlighted the urgent need to tackle systemic inequality and address social determinants of health. Our review provides timely evidence on PSH, income assistance, and mental health interventions as a means of improving housing stability. PSH has major cost and policy implications and this approach could play a key role in ending homelessness. Evidence‐based reviews like this one can guide practice and outcome research and contribute to advancing international networks committed to solving homelessness.

## 1 PLAIN LANGUAGE SUMMARY

### 1.1 Housing, income assistance, and case management improve housing outcomes for persons with lived experience of homelessness

Permanent supportive housing (PSH) interventions appear to improve short‐ and long‐term housing stability for persons with lived experience of homelessness. Income assistance and intensive mental health interventions show moderate benefits in housing outcomes, and evidence on standardised case management suggests potential to improve housing stability. Peer support alone does not impact housing stability. Inconsistent results on mental health, substance use and other social outcomes require additional research.

#### 1.1.1 What is this review about?

Homelessness greatly magnifies morbidity and mortality and worsens preventable health and social inequities. We present evidence on a wide range of interventions targeting homelessness: PSH; income assistance; standard case management (SCM) and peer support; mental health interventions such as assertive community treatment (ACT), intensive case management (ICM), critical time intervention (CTI), and injectable antipsychotics; and substance use interventions such as SCFs, managed alcohol programmes (MAPs) and pharmacological interventions for opioid use disorders.

**What is the aim of this review?**

This systematic review and meta‐analysis examines the effects of a broad range of interventions on housing stability, mental health, quality of life, substance use, hospitalisations and health service utilisation, as well as employment and income among individuals with lived experience of homelessness.

### 1.2 What studies are included?

We included 86 studies across 128 publications among individuals with lived experience of homelessness. The vast majority of studies followed a randomised controlled design. Most took place in the United States (73). The rest were undertaken in Canada (8), the UK (2), the Netherlands (2) and Australia (1).

### 1.3 What are the main findings of this review?

Studies on housing interventions showed significant improvements in housing stability, with potential sustained benefit for up to 5.4 years. Income assistance interventions also appeared to be effective in improving housing outcomes. SCM carried the potential to improve housing, with mixed evidence suggesting its added benefit, whereas peer support programmes demonstrated no impact on housing relative to usual care.

Intensive mental health interventions demonstrated moderate improvements in housing stability and often worked in synergy with permanent housing.

Cost‐analysis studies of housing interventions reported mixed economic results. Income assistance was associated with increased costs that were offset by its added benefits. Intensive mental health interventions, such as ACT, ICM and CTI, were found to be economically beneficial. In contrast, SCM did not offer good value for money compared to other interventions.

No economic evidence was found for peer support, injectable antipsychotics or substance use interventions.

### 1.4 What do the findings of this review mean?

PSH may improve and maintain housing stability. Further examination of implementation barriers of housing programmes is needed to inform decisionmakers.

Income assistance, SCM and intensive mental health interventions carry the potential to improve housing outcomes, but more research is needed to examine their mechanisms. Our results on mental health and other social outcomes were mixed and inconclusive. This could be attributed to the significant proportion of study participants who were suffering from chronic mental health or substance use conditions.

### 1.5 What are the implications for research and policy?

More longitudinal research is needed to better examine nonhousing outcomes. Furthermore, poor reporting, lack of blinding and allocation bias reduced the certainty and precision of our results.

There are other ongoing gaps that warrant more investigation, including peer support programmes, community substance use interventions, and programmes targeted towards special populations. Further examination of implementation barriers of housing programmes is also needed.

The absence of evidence on substance use interventions for people living with homelessness represents an important research and policy gap.

### 1.6 How up‐to‐date is this review?

The review authors searched for studies that had been published up until February 10, 2020.

## 2 BACKGROUND

### 2.1 The problem, condition or issue

Worldwide, over 1.8 billion people lack adequate housing and almost 25% of the world's urban population reside in informal accommodation (UN HRC, 2019). “People with a lived experience of homelessness” is a term coined to describe individuals who are, have been, or at risk of becoming homeless. This population lacks stable, permanent, appropriate housing, or may be without immediate prospect, means and ability to acquire it (Canadian Observatory on Homelessness, 2017). This population continues to grow, giving rise to a major international clinical and public health priority. Homelessness is strongly associated with high levels of morbidity (Hwang, 2009) and mortality (Nordentoft & Wandall‐Holm, 2003). People with lived experience of homelessness are at an increased risk for acute illnesses such as traumatic injury (including brain injury), frostbite, peripheral vascular disease, soft tissue infections, and dental decay (Hwang & Bugeja, 2000). Many homeless people also suffer from chronic medical conditions such as diabetes (Hwang & Bugeja, 2000), cardiovascular disease (Lee et al., 2005), cancer (Krakowsky et al., 2013) and respiratory illnesses (Raoult et al., 2001). Rates of serious mental illness (Fazel et al., 2014), cognitive impairment (Stergiopoulos et al., 2015b) and drug and alcohol use (Aubry et al., 2012; Grinman et al., 2010; Kennedy et al., 2017; Kerr et al., 2009; Torchalla et al., 2011) are disproportionately high, as are rates of homicide and suicide (Cheung & Hwang, 2004). Moreover, people who are homeless experience a disproportionately high prevalence of infectious diseases such as hepatitis C, HIV and tuberculosis (Beijer et al., 2012; Corneil et al., 2006; Roy et al., 2001). Despite the significant burden of disease, people with lived experience of homelessness are less likely to access and maintain the care required for their cure and treatment (Milloy et al., 2012; Palepu et al., 2011). People with lived experience of homelessness encounter many barriers to health and social care. The competing need to find food and shelter results in delays in accessing health care services (Gelberg et al., 1997) and those who do seek health care often experience discrimination that precludes adequate uptake of preventative health services (Wen et al., 2007). The structural stigma they experience when accessing health or social services is a major cause of their health inequities (Hatzenbuehler et al., 2013). For example, people with lived experience of homelessness with coexisting mental health conditions report specific barriers to accessing care, such as being unaware of the location of care, affordability, wait times and having experienced previous rejection from health or social services (Rosenheck et al., 1997). In addition, many health care recommendations, such as dietary advice, can prove impossible without access to resources, such as proper nutrition and cooking facilities (Hwang, 2001). This lack of appropriate access to community based care and reliable social contexts to implement preventive health behaviours results in disproportionately high acute care use by people with lived experience of homelessness (Saab et al., 2016). This population frequently experiences longer hospital stays and a higher risk of unplanned readmission than the general population (Saab et al., 2016), as discharge planning is compromised by inadequate housing to return to and suboptimal structures to support proper follow up care (Kushel, 2016).

Substantial research demonstrates that people with lived experience of homelessness benefit from receiving tailored, patient‐centred care within interprofessional teams with an integrated approach to community and social services (Coltman et al., 2015; Hwang & Burns 2014; James et al., 2005). A systematic review on health interventions for marginalised and socially excluded populations identified a range of potentially effective interventions that have relevance for marginalised and excluded populations, but it was not specific to people with lived experience of homelessness (Luchenski et al., 2017). Additionally, numerous studies have looked at the effectiveness of patient‐centred care for people with lived experience of homelessness within community services and social services (Coltman et al., 2015; Hwang & Burns 2014; James et al., 2005). Our review aims to evaluate current evidence on the effectiveness and cost effectiveness of interventions that directly or indirectly improve the health of those with lived experience of homelessness.

### 2.2 Description of the condition

#### 2.2.1 The intervention

**Figure 1:**
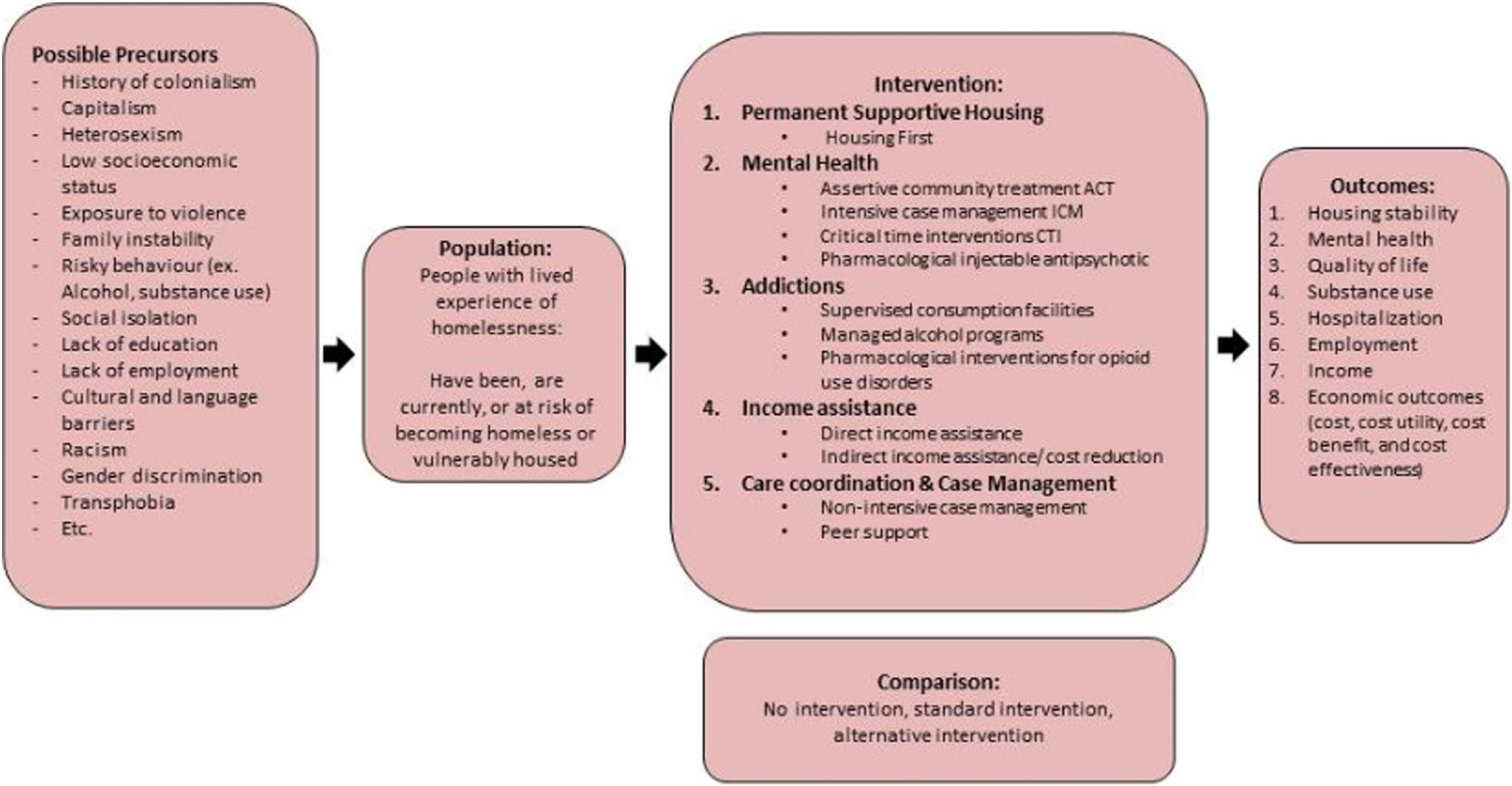
Logic model

We evaluated the effectiveness and cost‐effectiveness of interventions for people with lived experience of homelessness that aim to improve these people's health, service usage, and social outcomes. Prior to conducting this systematic review, to rank the priority topics and the needs that were the most important for this vulnerable population, we used a Delphi Consensus process (Keeney et al., 2010) to engage 76 people with lived experience of homelessness and 84 healthcare workers and researchers with professional experience in the field of homelessness (Shoemaker et al., 2020). A literature review and consensus process informed the final selection of the five categories of interventions to be included in this review (see Figure 1).

### 2.3 Description of the intervention

#### 2.3.1 Housing interventions

PSH is long‐term housing in the community combined with the provision of individualised supportive services that are tailored to participants' needs and choices. PSH follows the principles of “Housing First,” whereby the ability to access housing is not contingent on sobriety/abstinence, or the ability to follow through with treatment plans (Benston, 2015). This approach runs in contrast to what has been the orthodoxy of “treatment‐first” approaches, whereby people experiencing homelessness are placed in emergency services and must address certain personal issues (e.g., addictions, mental health) prior to being deemed “ready” for housing. Rather, in “Housing First,” the priority is to provide an individual with permanent housing and the choice to access treatment and supports, such as ACT or ICM offered by a multidisciplinary team (Aubry, Nelson, et al., 2015; Somers et al., 2017).

#### 2.3.2 Income assistance interventions

- Income assistance is a fundamental intervention for preventing and addressing homelessness. It consists of interventions that directly increase an individual's available income or improve their access to basic living necessities. Some examples include government assistance (i.e., income‐supplement programme (Brownell et al., 2016), charity donation or panhandling (Poremski et al., 2015), provision of cheques, tax benefits or cash transfers. Cash transfers are a form of financial aid offered on either a conditional or an unconditional basis (Lagarde et al., 2009). Further examples include support finding and maintaining employment or offering information on income benefits or financial literacy/debt‐management counselling (Abbott & Hobby, 2000), and the provision of food, daycare and fuel or rent supplements (Gruber et al., 2000; Power et al., 2015; Whittle et al., 2015).

#### 2.3.3 SCM and peer support

- Case management entails a myriad of services in which people with multiple morbidities or issues are supported by case managers who assess, plan and facilitate the access to health and social services that are necessary for the person's plan of care and recovery (De Vet et al., 2013). There are many different types of care‐coordination models, and these vary according to approach and caseload. The more intensive models of case management will be examined under “Mental‐health interventions”. Here, we will examine two specific areas of less intensive‐care coordination.

##### Standard case management

- SCM allows for the coordination of an array of social, health‐care and other services to help individuals maintain good health and strong social relationships. This is achieved by “including engagement, assessment, planning, linkage with resources, consultation with families, collaboration with psychiatrists, patient psycho‐education, and crisis intervention” (Kanter, 1989). The Case Manager or navigator's role is performed by either a clinician, nurse, community outreach worker or social worker with an average caseload of 35 clients (De Vet, 2013; Guarino, 2011). The target population for standard case‐management models are people who are experiencing homelessness or those who are vulnerably‐housed and have complex health concerns and are presenting to primary‐care practitioners and are often provided with this type of care as a time‐limited service (De Vet, 2013).

##### Peer support

- Peer support includes the sharing of knowledge, experience, emotional, social or practical help by or with an individual who has experienced a similar background to the service user (Mead et al., 2001). Peer support workers may be termed differently in different settings, either as mentors, recovery coaches, or life coaches, and all offer emotional and social support to individuals who are newly homeless or on a treatment plan or path to recovery from substance use or homelessness (Barker & Maguire, 2017). Thus, due to their shared experiences and ability to establish a relationship built on trust, peers are uniquely positioned to assist persons experiencing homelessness due to their shared experiences and ability to establish a relationship built on trust (Barker & Maguire, 2017; Faulkner & Basset, 2012; Finlayson et al., 2016).

#### 2.3.4 Mental health interventions

- We assessed four evidence‐supported interventions that are relevant to serious mental illness, which is defined as conditions that substantially limit major life activities due to functional impairment (SAMHSA, 2016).

##### Assertive community treatment

- ACT consists of a multidisciplinary group of healthcare workers in the community, that offers team‐based care to persons with high levels of needs. This team has 24‐h/day, 7‐days/week availability and provides services tailored to the needs and goals of each service user (Coldwell & Bender, 2007; De Vet et al., 2013). There is no time limit on the services provided, but transfer to lower intensity services is common after a period of stability (Homeless Hub, n.d.) Ten service users per case manager is the typical caseload, and services are offered in a natural setting, such as the workplace, home or social setting (De Vet et al., 2013).

##### Intensive case management

- ICM is offered to persons with serious mental illness but who typically have moderate needs, such as fewer hospitalisations or less functional impairment, as well as for people experiencing addictions (Dieterich et al., 2017). ICM helps service users through the support of a case manager that brokers access to an array of services. The case manager accompanies the service user to meetings and can be available for up to 12 h/day, 7 days a week. Case managers for ICM often have caseloads of 15–20 service users each (De Vet, 2013).

##### Critical time intervention

- CTIs are a form of time‐limited ICM, defined as a service that supports continuity of care for service users during times of transition; for example, from a shelter to independent housing or following discharge from the hospital. This service strengthens the person's network of support in the community (Silberman School of Social Work, 2017). It is administered by a CTI worker and is usually limited to a period of 6–9 months after institutional discharge or placement in housing. It comprises of three phases: Phase 1: Transition—Provide support and begin to connect the client to people and agencies that will assume the primary role of support; Phase 2: Tryout—Monitor and strengthen support network and client's skills; Phase 3: Transfer of care—Terminate CTI services with support network safely in place (Gaetz et al., 2013; Herman & Mandiberg, 2010).

##### Injectable antipsychotics

- Injectable antipsychotics have a major role to play in the treatment for psychosis of patients living in precarious situations as these individuals often have a limited ability to follow through with oral medication treatment plans (Llorca et al., 2013). The clinical effectiveness of newer (aripiprazole, olanzapine, paliperidone and risperidone) and older antipsychotics (haloperidol, fluphenazine, flupenthixol) is similar (Castillo & Stroup, 2015), but their effectiveness among homeless and vulnerably housed persons is unknown.

#### 2.3.5 Interventions for substance use

- We assessed three interventions relating to substance‐use disorders (SUDs) that apply to people experiencing homelessness and those who are vulnerably housed.

##### Supervised consumption facilities (SCFs)

- SCFs are legally sanctioned facilities where people who use substances can consume pre‐obtained substances under supervision (Drug Policy Alliance, n.d.). There exist various terminologies for these facilities, including supervised injection facilities (SIF), supervised consumption sites (SCS), medically supervised injection centres (MCIS), among others. Such facilities are frequently used as safe spaces for people experiencing homelessness and those who are vulnerably housed as well as substance users.

##### Managed alcohol programmes

- A MAP provides shelter, medical assistance, social services and the provision of regulated alcohol to help residents cope with severe alcohol use disorder (Shepherds of Good Hope Foundation, n.d.). This programme is provided by professional staff and nurses.

##### Pharmacological interventions for opioid use disorder

- The effectiveness and cost‐effectiveness of opioid therapy medications, including buprenorphine/naloxone, naloxone, naltrexone (British Columbia Centre on Substance Use, 2017) methadone, and injectable diacetylmorphine (heroin; Haasen et al., 2007), have been documented in general‐population studies. The literature demonstrates the effectiveness of naltrexone (Krupitsky et al., 2011), buprenorphine (with or without naloxone), and methadone (McKeganey et al., 2013), as well as injectable diacetylmorphine (heroin; Haasen et al., 2007) for treating opiate dependence, but the evidence specific to homeless populations is yet to be synthesised.

### 2.4 How the intervention might work

#### 2.4.1 Housing interventions

- Access to adequate housing is an end‐all objective of most homeless individuals. The unconditional provision of stable housing that is permanent in tenure, supportive in nature, and scattered across the rental market has been associated with a positive impact on the long‐term residential stability of homeless individuals (Aubry et al., 2016; Tsemberis et al., 2004). As well, synchronising the provision of permanent housing with supportive services is found to improve social functioning and quality of life (Aubry et al., 2016; Stergiopoulos et al., 2015).
- Another model of providing PSH is to congregate accommodation units with supportive services in a single location that may be situated in a residential or commercial area and equipped with commonly used facilities. Such models were found to positively improve the housing and health‐related outcomes of homeless individuals, as per the scattered model (Somers et al., 2017).

#### 2.4.2 Income assistance interventions

- Financial hardships halt all efforts to end the cycle of homelessness (Institute of Medicine (US) Committee on Health Care for Homeless People, 1988). Evidence suggests that increasing income is an effective strategy to improve both access to health services and health status (Lagarde et al., 2009). This correlation may, very well, be the result of reduced financial stressors and increased ability to afford fundamental life necessities, such as housing, food, and medications (Richards et al., 2008). Moreover, it was found that providing information on income‐assistance resources has the potential to improve the physical and psychosocial health of disadvantaged populations (Adams et al., 2006).

#### 2.4.3 SCM and peer support

##### Standard case management

- Regardless of the heterogeneity and complexity of case‐management models, literature suggests that case management is associated with improved residential stability and substance‐use outcomes (De Vet, 2013). Patients who are provided with the services of a case manager are more likely to feel supported and guided in their quest to access and maintain fundamental health and social services (Conrad et al., 1998).

##### Peer support

- The peer‐support model employs workers with shared life experiences to provide social support, advocacy, education, and role modelling to homeless individuals (Barker & Maguire, 2017). Among homeless populations, these elements have been found to improve quality of life and reduce problematic substance use (Barker & Maguire, 2017).

#### 2.4.4 Mental health interventions

##### Assertive community treatment

- Evidence suggests that individuals experiencing homelessness may benefit from receiving support in the form of ACT. This model of care is found to reduce days on the street or hospitalised, increase community functioning, and improve life satisfaction and mental‐health‐related outcomes for this vulnerable population (Coldwell & Bender, 2007; De Vet, 2013). The positive impact of this model of care could be attributed to the strength of the provider‐service‐user relationship and the sincere effort of the multidisciplinary team providing the care (Stuart, 2009).

##### Intensive case management

- Literature is abundant with evidence that suggests the added benefits of providing ICM to disadvantaged populations. This model of care carries the potential to decrease hospitalisations, increase access to care and reduce economic hardships (De Vet, 2013; Dieterich et al., 2017).

##### Critical time intervention

- The benefit of CTI lies in its ability to prevent the discontinuity of care during periods of transition. Providing CTI to homeless individuals was associated with increased residential stability, decreased mental‐health symptomatology and strengthened ties with services, family, and friends (De Vet, 2013; Jones et al., 2003).

##### Injectable antipsychotics

- Injectable antipsychotics have been found to be effective for managing serious mental illnesses and reducing episodes of mental‐health emergencies (Llorca et al., 2013).

#### 2.4.5 Interventions for substance use

##### Supervised consumption facilities

- There is increasing evidence suggesting the benefit of SCFs in reducing precarious substance‐use‐related public behaviours and increasing access to and maintenance of treatment services (Kennedy et al., 2017; Wood et al., 2007). These facilities are believed to provide a safe environment for accessible services that provide nonjudgemental staff who help connect clients to health and social services as needed.

##### Managed alcohol programmes

- MAPs follow a harm‐reduction approach by providing clients with shelter and regulated alcohol dispensing, accompanied by health and social support as needed (Podymow et al., 2006). The scarce literature on these interventions among homeless individuals suggest its effectiveness in reducing alcohol intake, hospitalisations and incarcerations (Podymow et al., 2006).

##### Pharmacological interventions for opioid use disorder

Opioid agonist therapy (OAT) such as methadone, buprenorphine/naloxone, and oral morphines have been associated with decreased mortality and morbidity (British Columbia Centre on Substance Use, 2017). As well, evidence suggests that opioid antagonist therapy such as naltrexone is successful in mitigating overdose‐related mortalities and incarceration rates (Roozen et al., 2006).

### 2.5 Why it is important to do this review

There has been a long‐standing social discourse on effectively tackling the negative consequences of the urban homelessness that has plagued communities internationally. Persons with lived experience of homelessness face higher rates of infectious and chronic disease and disproportionately poorer outcomes, with reduced rates of access to effective quality care. In order to better understand the needs and resources available for people with lived experience of homelessness, policymakers, practitioners, and allied health professionals need high‐quality systematic reviews and knowledge‐translation strategies on interventions that are specific to this population. Our review aims to provide a comprehensive overview of the benefits and cost‐effectiveness of interventions designed to indirectly or directly improve the health and well‐being of persons with lived experience with homelessness. We present updated perspectives on the effect of PSH, income assistance, SCM/peer support and mental‐health and substance‐use interventions, on the housing stability, mental health, quality of life, hospitalisations, earned income and employment statuses of persons with lived homelessness. This review is part of a series of other publications that inform a national practice guide on homelessness (Pottie et al., 2020), and serves to provide best‐practice updates to health‐care professionals and policymakers and guide the future care of persons with lived experiences of homelessness.

## 3 OBJECTIVES

The objective of this systematic review is to identify, appraise, and synthesise the best available evidence on the effectiveness and cost‐effectiveness of interventions to improve the health and social outcomes of people experiencing homelessness and those who are vulnerably housed. Our outcomes of interest include housing stability, mental health, quality of life, substance use, hospitalisations, employment and income. The following research questions were developed to guide the formation of the systematic review:

1. What is the effectiveness of PSH on the health and social outcomes of people experiencing homelessness and those who are vulnerably housed?
2. What is the effectiveness of income assistance on the health and social outcomes of people experiencing homelessness and those who are vulnerably housed?
3. What is the effectiveness of SCM and/or peer support on the health and social outcomes of people experiencing homelessness and those who are vulnerably housed?
4. What is the effectiveness of mental‐health interventions (ACT, ICM, CTI and injectable antipsychotics) on the health and social outcomes of people experiencing homelessness and those who are vulnerably housed?
5. What is the effectiveness of interventions for substance use (SCFs, MAPs and pharmacological interventions for opioid use disorder) on the health and social outcomes of people experiencing homelessness and those who are vulnerably housed?
6. What are the costs and cost‐effectiveness of the aforementioned interventions for people experiencing homelessness and those who are vulnerably housed?

## 4 METHODS

### 4.1 Criteria for considering studies for this review

#### 4.1.1 Types of studies

The protocol was registered with the Campbell Collaboration (Pottie et al., 2019) and reported according to the preferred reporting items for systematic reviews and meta‐analyses for protocols (PRISMA‐P; Moher et al., 2015). The results of the review are reported using the PRISMA reporting guidelines (Moher et al., 2009).

We included studies as recommended by the Cochrane Effective Practice and Organisation of Care (EPOC) Cochrane group for reviews of effectiveness (Effective Practice and Organisation of Care (EPOC)‐Cochrane, 2015). We included randomised control trials (RCTs), non‐RCTs, controlled before‐after studies, interrupted time‐series studies, and repeated‐measures studies. We considered studies published in both peer‐reviewed journals and grey literature.

#### 4.1.2 Types of participants

We included populations experiencing homelessness, defined as those who lack stable, permanent, appropriate housing, or who may be without immediate prospects, means, and ability to acquire it. Such physical living situations can include emergency shelters or provisional accommodations (Canadian Definition of Homelessness, 2017). Studies must have reported whether participants were experiencing homelessness in order to be included in this review. We included studies that included a subset of the sample experiencing homelessness as long as 50% of the participants were homeless. We included studies that were among individuals or families and we did not restrict our inclusion criteria by age, sex or gender. We excluded studies that were specific to indigenous populations experiencing homelessness, as this line of inquiry is being pursued by an indigenous‐specific research team (Thistle & Smylie, 2020). We excluded all other populations.

#### 4.1.3 Types of interventions

We included the following interventions, as outlined in Section 1.1.2: PSH: income assistance, SCM, ICM, ACT, CTI, peer support, SCFs, MAPs, injectable antipsychotics, and OAT. We included studies that had multicomponent interventions as long as one of the interventions applied to those mentioned above.

All of the included interventions were either compared to an inactive control (i.e., a placebo, no treatment, standard care) or to an active control intervention (alternative or variant of the intervention; Effective Practice and Organisation of Care (EPOC)‐Cochrane, 2015). In the case of an interrupted time series, the control must have been a historical control with three data points. If a study with more than two intervention arms was included, then we only included the intervention and control arms that met the eligibility criteria.

#### 4.1.4 Types of outcome measures

Studies were included in this review if they reported the use of validated measures and reported at least one of the following outcomes.

##### Primary outcomes

1. Housing stability: Any measures assessing participants housing status, such as the number of days in stable housing, number of days homeless (on the street or in shelters), number of participants in stable housing, and number of participants homeless (on the street or in shelters). Typical tools to measure housing stability include the Residential Timeline Followback Inventory (RTLFB) (Tsemberis et al., 2007).

##### Secondary outcomes

2. Mental health: any measures assessing psychological status and wellbeing, including but not limited to, psychological distress, self‐reported mental health status, or mental illness symptoms. Typical tools to measure mental health include the Colorado Symptom Index (CSI) (Boothroyd & Chen, 2008), and the self reported mental status SF‐12 (Nelson et al., 2012).
3. Quality of life: which include any assessment of well‐being, life and personal satisfaction, quality of social relationships, and any specific physical or mental quality of life measures. Typical tools to measure quality of life include the Lehman Quality of Life Interview (Lehman et al., 1996), and the EuroQoL 5‐D scale (Lamers et al., 2006).
4. Hospitalisation: any measures of participants' use of hospital and emergency services, such as the number of days hospitalised or number of visits to the emergency department. Such measures can be assessed through a standard questionnaire asking participants for their service use, or through accessing the hospital data sets.
5. Substance use: As measured by the number of days using alcohol or substance, the rate and frequency of using alcohol or substances, number of days of abstinence from alcohol or substances or physical and mental consequences of using alcohol or substances. Typical tools to measure substance use outcomes include the Global Appraisal of Individual Needs‐Short Screener of Substance Use Problems GAIN‐SS (Dennis et al., 2006).
6. Income: Any measures of money that participants acquire from different resources including social assistance, disability benefits, donations, and part or full time employment. Such measures can be assessed through a standard questionnaire asking participants for their weekly, monthly or annual income from different sources.
7. Employment: Any measures of employment that participants partake during the study period, including but not limited to, number of days of paid employment, number of employed or unemployed participants, hourly wage, and employment tenure. Such measures can be assessed through a standard questionnaire asking participants about their employment rates during the study period.
8. Economic outcome: any measures of cost, cost benefit, cost utility or incremental cost‐effectiveness ratio. Such measures can be obtained from administrative databases and cost reports. An example of how an individualised programme cost could be calculated is dividing the sum of all on‐site operation and services costs (maintenance, utilities, insurance, etc.) by the capacity of the project (Larimer, 2009).

###### Duration of follow‐up

All durations of follow‐up were included. We collected data at each available time‐point.

###### Types of settings

We included studies where the intervention took place in any setting where the primary care of people experiencing homelessness takes place. Primary care is known as the “entry point to the larger health care system” (Tarlier, 2007) and can be provided by professionals from many disciplines, such as family physicians, psychiatrists, social workers, emergency physicians, and so forth. We also included community‐based interventions provided in social‐service or shelter/supervised consumption locations, private or nonprivate clinics, hospital emergency rooms, outreach care, street patrols, mobile care units, and so forth.

We included studies that occurred in high‐income countries and excluded studies that occurred in low‐ and middle‐income countries (World Bank, 2019).

### 4.2 Search methods for identification of studies

We searched for relevant literature in consultation with an information specialist (librarian).

#### 4.2.1 Electronic searches

We searched the following bibliographic databases from database inception to February 10, 2020:

- Ovid MEDLINE(R) and Epub Ahead of Print, In‐Process & Other Non‐Indexed Citations, Daily and Versions(R) (1946 to February 7, 2020)
- EBM Reviews—Cochrane Central Register of Controlled Trials (January 2020)
- EBM Reviews—Cochrane Database of Systematic Reviews (2005 to February 4, 2020)
- EBM Reviews—Database of Abstracts of Reviews of Effects (1st Quarter 2016)
- EBM Reviews—Health Technology Assessment (4th Quarter 2016),
- EBM Reviews—NHS Economic Evaluation Database (1st Quarter 2016)
- Embase (1974 to February 7, 2020)
- EBSCO CINAHL (–2020)
- EBSCO PsycINFO (–2020)
- Epistemonikos (–2020)

We used a combination of subject headings and keywords including “homeless”, “marginalized” and “shelter”. Full strategies for each database can be found in Appendix A.

#### 4.2.2 Searching other resources

The reference lists of all articles selected for full‐text review were manually searched for relevant citations. These were cross‐referenced against our original search results and any additional potentially relevant citations were screened. Further, we consulted content experts for any publications or resources that might enrich our findings and we screened their suggestions against our inclusion criteria.

### 4.3 Data collection and analysis

We collected and analysed data according to our protocol (Pottie et al., 2019).

#### 4.3.1 Selection of studies

Teams of two reviewers independently screened titles and abstracts in duplicate. We pilot tested the screening criteria at both the title‐and‐abstract‐screening stage and the full‐text stage. We used the PRISMA flow diagram to report the eligibility of studies. We retrieved the full text of all of the studies that passed this first‐level screening. The full‐text reviews were also done in duplicate by two reviewers, and agreement was reached by consensus. Disagreements were resolved by consultation with a third reviewer.

#### 4.3.2 Data extraction and management

We developed a standardised extraction sheet for each topic variable and created a table of characteristics to describe a summary of findings from our included studies. The data extraction sheet was piloted by two independent reviewers. We collected and utilised all relevant numerical data (SDs, effects estimates, confidence intervals [CIs], test statistics, *p* values, etc.). Teams of two reviewers extracted data in duplicate and independently. The reviewers compared their results and resolved disagreements by discussion or with help from a third reviewer.

#### 4.3.3 Assessment of risk of bias in included studies

We assessed risk of bias according to guidance for Cochrane Effective Practice and Organisation of Care (EPOC) reviews (Effective Practice and Organisation of Care (EPOC)‐Cochrane, 2015). Nine standard criteria are suggested for all randomised trials, nonrandomised trials and controlled before‐after studies, including random‐sequence generation, allocation concealment, baseline‐outcome measurements, baseline characteristics, incomplete‐outcome data, knowledge of the allocated interventions adequately prevented during the study, protection against contamination, selective outcome reporting, and other biases.

Risk of bias was assessed by teams of two review authors, in duplicate. Discrepancies were resolved through discussion or by a third reviewer.

It is important to highlight that we did not subject any of the identified economic evaluations to critical appraisal, in accordance with guidance from the Cochrane Handbook (Shemilt et al., 2019).

#### 4.3.4 Measures of treatment effect

Whenever possible, we performed statistical analyses using RevMan 5. For dichotomous data, we used odds ratios (OR) and risk ratios (RR) with 95% CIs. For continuous data, we used the mean difference (MD) with 95% CI, if the outcomes were measured in the same way between trials. We used the standardised mean difference (SMD) with 95% CI to combine the trials that measured the same outcome but used different methods of measurement.

#### 4.3.5 Unit of analysis issues

The results from some studies were reported in multiple publications. Therefore, to prevent the double counting of data, individual records were screened to identify unique studies and were evaluated for potential overlap by comparing the study design, enrolment and data‐collection dates, authors and their associated affiliations, and the reported selection and eligibility criteria. When reviewing multiple publications, we included only unique data from each study. Several studies included outcome data for multiple time points. Comparisons were therefore carried out separately for periods of 6 months and less (short term), 6–18 months (midterm), and 18 months or more (long‐term). If multiple measures of the same outcome were reported, we prioritised outcomes measured using validated scales for meta‐analyses and reported all available outcome data narratively.

#### 4.3.6 Dealing with missing data

We will contact authors once for any missing data. We will use any supplementary data provided by authors in our analysis, or report findings as extracted otherwise.

#### 4.3.7 Assessment of heterogeneity

We assessed heterogeneity among studies in two ways. First, we assessed clinical heterogeneity: heterogeneity in population, interventions or outcomes. We used *I* ^2^ statistics as a guide to assess heterogeneity along with a visual inspection of forest plots.

#### 4.3.8 Assessment of reporting biases

There were very few outcomes that provided enough data for a meta‐analysis; therefore we could not assess for reporting bias. For future updates, funnel plots would be used if there are 10 or more studies in a meta analysis for one outcome and an investigation would be conducted for reporting biases, for example, publication bias.

#### 4.3.9 Data synthesis

We aimed to conduct a separate meta‐analysis for each outcome and intervention. We assessed clinical heterogeneity by considering the study population, intervention, comparison, outcome measure, and timing of outcome assessment. The assessments were made in consultation with members of the research team with clinical and statistical expertise. We pooled data from studies we judged to be clinically homogeneous. If more than one study provided usable data in any single comparison, we performed a meta‐analysis. We standardised all the reported effect sizes as RRs for the dichotomous outcomes and MDs or SMDs for the continuous outcomes. For the majority of findings, characteristics of studies (such as study designs, intervention types, or outcomes) were too diverse to yield a meaningful summary estimate of effect. Similarly, we deemed forest plots of single studies to be of limited value to the review. When heterogeneity precluded a meta analysis, we synthesised our findings narratively as recommended by the synthesis without meta‐analysis (SWiM) reporting guideline (Campbell et al., 2020).

#### 4.3.10 Subgroup analysis and investigation of heterogeneity

Based on the availability of the data, we had planned to conduct subgroup analyses for the following subgroups: women, youth, and people with disabilities. However, since very few studies were included in each comparison within the review, we could not conduct any of the aforementioned subgroup analyses.

#### 4.3.11 Sensitivity analysis

We had planned to conduct sensitivity analyses; however, since very few studies for each intervention were included in the review, we could not conduct any sensitivity analyses.

#### 4.3.12 Summary of findings and assessment of the certainty of the evidence

We assessed the certainty of evidence for housing‐stability outcomes by using the GRADE (Grading of Recommendations, Assessment, Development and Evaluations) approach (Balshem et al., 2011). Housing‐stability outcomes were selected as critical patient‐important outcomes by our review team, in consultation with content experts and people with lived experience of homelessness. GRADE rates certainty of evidence are as follows:

**Table:** 

Certainty of evidence	Definition
High	There is a lot of confidence that the true effect lies close to that of the estimated effect
Moderate	There is moderate confidence in the estimated effect: The true effect is likely to be close to the estimated effect, but there is a possibility that it is substantially different
Low	There is limited effect on the estimated effect: The true effect might be substantially different from the estimated effect
Very low	There is very little confidence in the estimated effect: The true effect is likely to be substantially different from the estimated effect

## 5 RESULTS

### 5.1 Description of studies

We included 86 studies, described below.

#### 5.1.1 Results of the search

**Figure 2:**
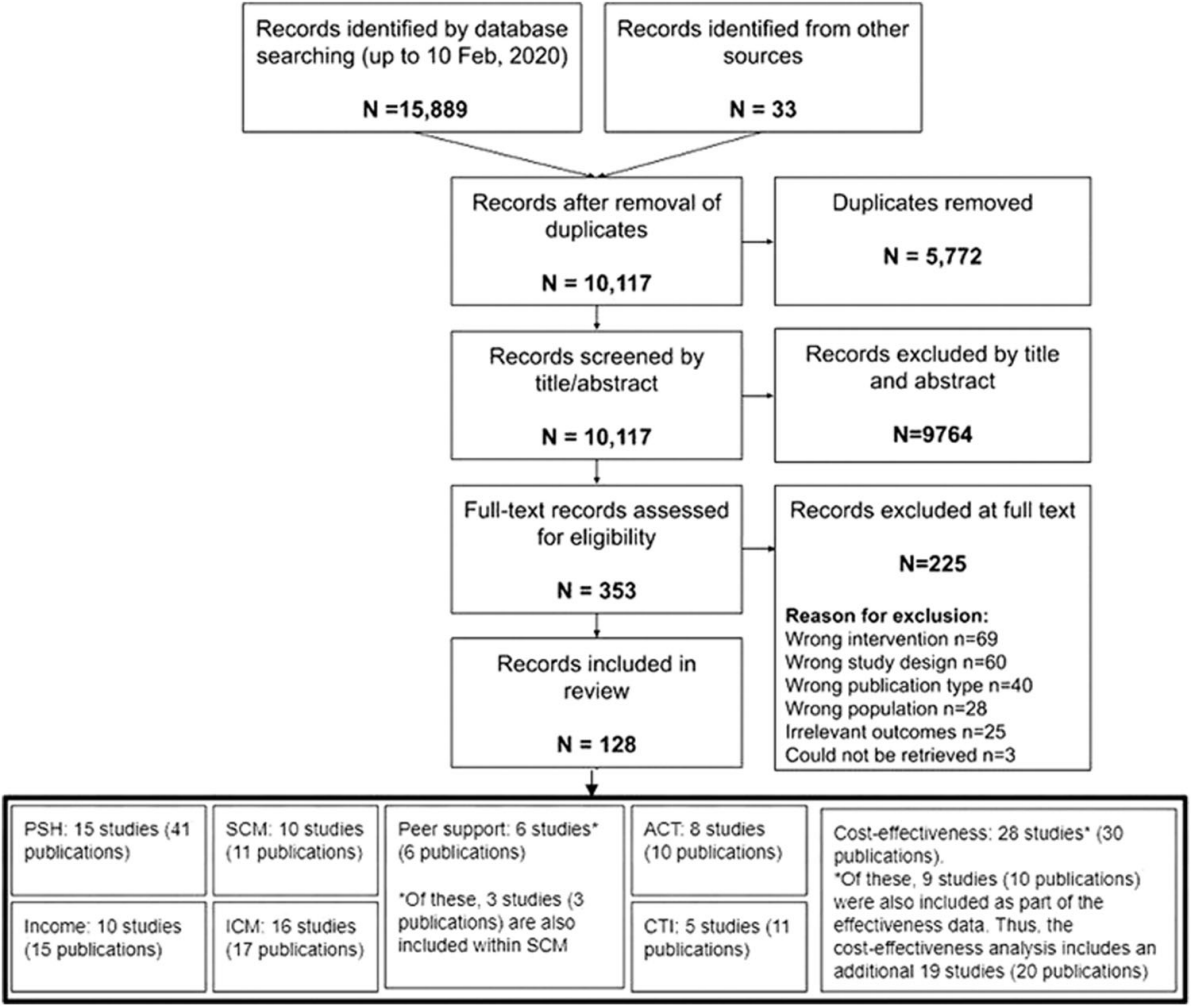
PRISMA flow diagram

The search was performed from database inception up to February 10, 2020. The search resulted in 15,889 citations, and an additional 33 were identified from other sources. After removal of duplicates we screened 10,117 unique citations by title and abstract, leaving 353 citations that were potentially eligible for inclusion in this review. Full‐text reviews of these works identified 128 citations for inclusion in this review. Figure 2 depicts the search‐and‐selection flow diagram.

#### 5.1.2 Included studies

We included a total of 86 studies (128 publications), broken down by the interventions below:

##### Permanent supportive housing

We included 15 studies (41 publications) examining the effectiveness of PSH (Aubry et al., 2016; Cherner et al., 2017; Goldfinger et al., 1999; Hwang et al., 2011; Lipton et al., 1988; Martinez & Burt, 2006; McHugo et al., 2004; Rich & Clark, 2005; Sadowski et al., 2009; Siegel et al., 2006; Stefancic & Tsemberis, 2007; Stergiopoulos et al., 2015; Stergiopoulos et al., 2019; Tsemberis et al., 2004; Young et al., 2009). Four studies were conducted in Canada (Aubry et al., 2016; Cherner et al., 2017; Hwang et al., 2011; Stergiopoulos et al., 2019) and the remaining were conducted in the United States. All studies provided PSH with either ACT or ICM, depending on the severity of the mental‐health symptoms and participants' needs. PSH models included scattered‐site and congregate settings. All interventions were delivered to individuals and no studies were specific to families, women or youth.

##### Income assistance

We included 10 studies (15 publications) examining the effectiveness of income‐assistance interventions (Booshehri, 2017; Ferguson, 2018; Forchuk et al., 2008; Gubits et al., 2018; Hurlburt et al., 1996; Kashner, 2002; Pankratz et al., 2017; Poremski et al., 2015; Rosenheck et al., 2003; Wolitski, 2009). Five studies investigated the impact of housing subsidies with (Hurlburt et al., 1996; Pankratz et al., 2017; Rosenheck et al., 2003; Wolitski, 2009) or without (Gubits et al., 2018) case management. One study offered assistance finding housing and rental supplements (Forchuk et al., 2008) and the remaining four studies assessed the effectiveness of financial education (Booshehri, 2017), compensated work therapy (CWT; Kashner, 2002), or individual‐placement support (IPS; Ferguson, 2018; Poremski et al., 2015). Two studies were conducted among families (Booshehri, 2017; Gubits et al., 2018) and one among youth (Ferguson, 2018). Three studies were conducted in Canada (Forchuk et al., 2008; Pankratz et al., 2017; Poremski et al., 2015) and the remaining seven took place in the United States.

##### Standard case management

We included 10 studies (11 publications) examining the effectiveness of SCM (Conrad et al., 1998; Graham‐Jones et al., 2004; Hurlburt et al., 1996; Lapham et al., 1996; Nyamathi et al., 2001; Nyamathi et al., 2016; Sosin et al., 1995; Towe et al., 2019; Upshur et al., 2015; Weinreb et al., 2016). All studies were conducted among populations either experiencing or at‐risk for homelessness, with varying degrees of need. Three studies were specific to women (Nyamathi et al., 2001; Upshur et al., 2015; Weinreb et al., 2016); two were specific to men (Conrad et al., 1998; Nyamathi et al., 2016), and five contained mixed‐gender populations (Graham‐Jones et al., 2004; Hurlburt et al., 1996; Lapham et al., 1996; Sosin et al., 1995; Towe et al., 2019). Most (*n* = 9) studies were set in the United States and one study was conducted in the United Kingdom (Graham‐Jones et al., 2004). The interventions focused on care coordination, including links to primary care (Graham‐Jones et al., 2004; Nyamathi et al., 2001; Weinreb et al., 2016), housing (Hurlburt et al., 1996; Sosin et al., 1995), mental‐health counselling (Lapham et al., 1996; Upshur et al., 2015), and skills provision, such as relapse prevention skills (Conrad et al., 1998), infectious‐disease risk reduction (Nyamathi et al., 2001), and coping skills (Nyamathi et al., 2016). Two studies also included peers with lived experience in their intervention delivery (Nyamathi et al., 2001; Nyamathi et al., 2016).

##### Peer support

We included six studies (six publications) examining the effectiveness of peer‐support interventions (Corrigan et al., 2017; Ellison et al., 2020; Lapham et al., 1996; Nyamathi et al., 2001; Nyamathi et al., 2016; Yoon et al., 2017). Of these, three studies were three‐arm trials where the third arm is also included under SCM (Lapham et al., 1996; Nyamathi et al., 2001; Nyamathi et al., 2016). All studies were conducted in the United States. In four studies, peers played the role of navigators and mentors, providing training in effective coping skills, self‐management, goal‐setting and assistance in navigating the health‐care and social‐care systems (Corrigan et al., 2017; Nyamathi et al., 2001; Nyamathi et al., 2016; Yoon et al., 2017). Two studies integrated peers into housing programmes, where peers offered support services focused on mental health and substance‐use recovery and community integration (Ellison et al., 2020; Lapham et al., 1996).

##### Intensive case management

We included 16 studies (17 publications) examining the effectiveness of ICM (Braucht, 1995; Burnam, 1995; Cauce, 1994; Clark & Rich, 2003; Cox et al., 1998; Felton et al., 1995; Grace & Gill, 2014; Korr & Joseph, 1996; Malte et al., 2017; Marshall et al., 1995; Orwin et al., 1994; Rosenblum et al., 2002; Shern et al., 2000; Shumway et al., 2008; Stahler et al., 1995; Toro et al., 1997). Most studies were conducted among homeless individuals with mental illness and/or substance‐use problems, with two studies conducted among adolescents and young adults (Cauce, 1994; Grace & Gill, 2014) and one conducted among adults with children (Toro et al., 1997). Fourteen studies were conducted in the United States, one study was conducted in the United Kingdom (Marshall et al., 1995) and one in Australia (Grace & Gill, 2014). All interventions had a low caseload (12–20 clients) and one intervention included peers with lived experience (Felton et al., 1995).

##### Assertive community treatment

We included 8 studies (10 publications) examining the effectiveness of ACT (Clarke et al., 2000; Essock et al., 1998, 2006; Fletcher et al., 2008; Lehman et al., 1997; Morse et al., 1992, 1997, 2006). All studies were conducted among homeless adults, in the United States, who had serious and persistent mental illness. All participants received care from a multidisciplinary team that included a psychiatrist. Two studies also integrated a substance‐abuse specialist to their staff (termed “Integrated Assertive Community Treatment” [IACT]) (Fletcher et al., 2008; Morse et al., 2006). Three studies also included peers with lived experience of homelessness and/or poor mental health (often termed “consumers”, “consumer advocates” or “community workers”) in their ACT teams (Clark et al., 1998; Lehman et al., 1997; Morse et al., 1997).

##### Critical time intervention

We included five studies (11 citations) examining the effectiveness of CTI (De Vet et al., 2017; Herman et al., 2011; Lako et al., 2018; Shinn et al., 2015; Susser et al., 1997). Four studies were conducted among single adults and one study was conducted among families with children (Shinn et al., 2015). One study was conducted following participants' hospital discharge (Herman et al., 2011) and the remaining four studies were conducted while participants were residing in shelters. Two studies were conducted in the Netherlands (De Vet, 2017; Lako et al., 2018) and the other three were conducted in the United States. All CTI interventions followed the same three phases: (1) transition to the community, (2) tryout and (3) transfer of care.

##### Supervised consumption facilities

We did not identify any eligible studies on SCFs (empty review).

##### Managed alcohol programmes

We did not identify any eligible studies on MAPs (empty review).

##### Injectable antipsychotics

We did not identify any eligible studies on injectable antipsychotics (empty review).

##### Opioid agonist therapy

We did not identify any eligible studies on OATs (empty review).

##### Cost‐effectiveness

We identified 30 publications that reported on the cost‐effectiveness of our interventions (Aubry et al., 2016; Chalmers McLaughlin, 2011; Clark et al., 1998; Culhane et al., 2002; Dickey et al., 1997; Essock et al., 1998; Evans et al., 2016; Gilmer et al., 2009, 2010; Holtgrave et al., 2013; Hunter et al., 2017; Larimer, 2009; Latimer et al., 2019; Lehman, 1999; Lenz‐Rashid, 2017; Lim et al., 2018; Mares & Rosenheck, 2011; Morse et al., 2006; Nyamathi et al., 2016; Okin et al., 2000; Pauley et al., 2016; Rosenheck et al., 2003; Sadowski et al., 2009; Schinka et al., 1998; Shumway et al., 2008; Srebnik et al., 2013; Stergiopoulos et al., 2015; Susser et al., 1997; Tsemberis et al., 2004; Wolff, 1997). Of these, 10 publications were also included as part of the effectiveness data (Aubry et al., 2016; Essock et al., 1998; Morse et al., 2006; Nyamathi et al., 2016; Rosenheck et al., 2003; Sadowski et al., 2009; Shumway et al., 2008; Stergiopoulos et al., 2015; Susser et al., 1997; Tsemberis et al., 2004). Thus, the cost‐effectiveness analysis includes an additional 19 studies (20 publications). Eighteen of these studies took place in the United States, and only one examined cost‐effectiveness in the Canadian context (Latimer et al., 2019).

Twenty‐one publications provided cost‐effectiveness data on PSH and/or income‐assistance interventions (Aubry et al., 2016; Chalmers McLaughlin, 2011; Culhane et al., 2002; Dickey et al., 1997; Evans et al., 2016; Gilmer et al., 2009, 2010; Holtgrave et al., 2013; Hunter et al., 2017; Larimer, 2009; Latimer et al., 2019; Lenz‐Rashid, 2017; Lim et al., 2018; Mares & Rosenheck, 2011; Pauley et al., 2016; Rosenheck et al., 2003; Sadowski et al., 2009; Schinka et al., 1998; Srebnik et al., 2013; Stergiopoulos et al., 2015; Tsemberis et al., 2004). Of these, five publications also provided effectiveness data (Aubry et al., 2016; Rosenheck et al., 2003; Sadowski et al., 2009; Stergiopoulos et al., 2015; Tsemberis et al., 2004).

For mental‐health interventions, twelve articles provided evidence on cost‐effectiveness: three on SCM (Nyamathi et al., 2016; Okin et al., 2000; Shumway et al., 2008), 6 on ACT (Aubry et al., 2016; Clark et al., 1998; Essock et al., 1998; Lehman, 1999; Morse et al., 2006; Wolff, 1997), 2 on ICM (Rosenheck et al., 2003; Stergiopoulos et al., 2015); and 1 for CTI (Susser et al., 1997). Eight of the mental‐health cost‐effectiveness articles were also included in the effectiveness analysis (Aubry et al., 2016; Essock et al., 1998; Nyamathi et al., 2016; Morse et al., 2006; Rosenheck et al., 2003; Shumway et al., 2008; Stergiopoulos et al., 2015; Susser et al., 1997).

No cost‐effectiveness studies were identified for the other interventions (peer support, SCFs, MAPs, injectable antipsychotics and OAT).

#### 5.1.3 Excluded studies

The search process is diagrammed in Figure 2, which also shows the number of records excluded (*n* = 225), along with a summary of reasons. Further details of the excluded studies are also available in Excluded studies.

### 5.2 Risk of bias in included studies

**Figure 3:**
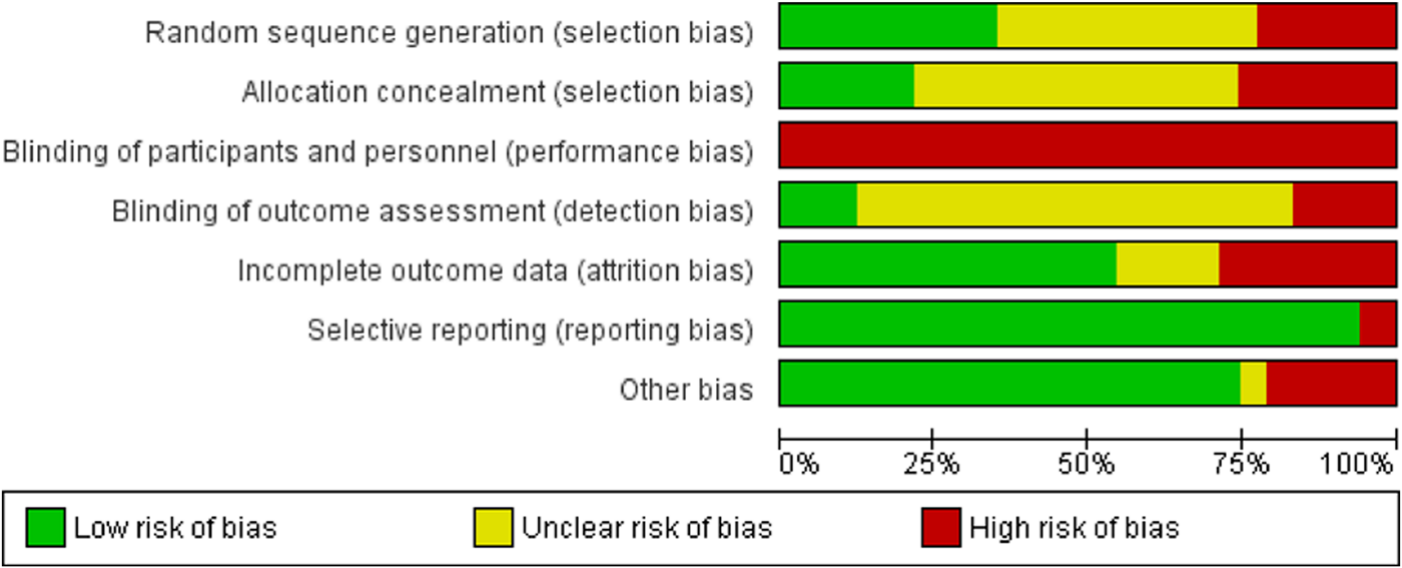
Risk of bias summary

**Figure 4:**
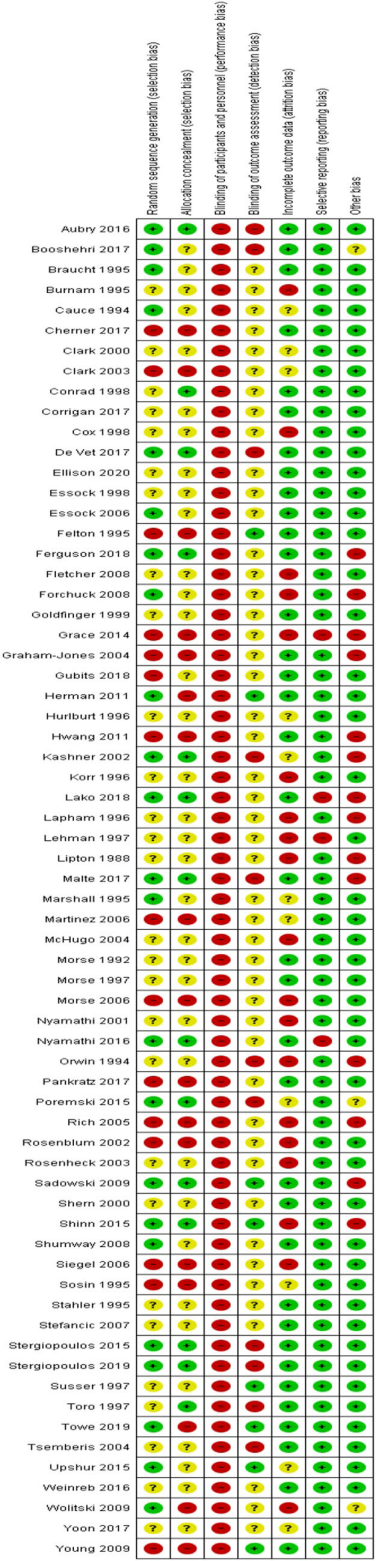
Risk of bias in individual studies

We judged there to be a moderate or high risk of bias in most categories in each of the studies reviewed, see Figure 3 for a summary of judgements on bias across the studies reviewed. Figure 4 provides an insight into the level of potential bias within each study. Characteristics of included studies provides the rationale for each of these judgements. Furthermore, given that the risk of bias associated with economic evaluations differs from that associated with standard RCTs, we did not critically appraise these studies using a standardised tool.

#### 5.2.1 Allocation (selection bias)

Selection bias occurs in intervention studies when there are systematic differences between comparison groups in response to treatment or prognosis (Henderson & Page, 2007). Intervention studies are especially susceptible to selection bias unless particular efforts are made to minimise it. The most effective method is random allocation to treatment and control groups. We included both randomised and nonrandomised trials of interventions in our review. As a result, 15 studies were assessed as having high risk of selection bias due to inadequate or absent random sequence generation, while 28 studies had unclear risk of bias on this item. Concerns with allocation concealment were similar, with only 14 deemed as low risk of bias.

#### 5.2.2 Blinding (performance bias and detection bias)

This potential bias is counteracted by the blinding of study participants and personnel, so that they are unaware of their group assignment, and the blinding of outcome assessors. Given the nature of the interventions and the impossibility of blinding of participants, all studies were assessed as high risk for performance bias. Blinding of outcome assessors was often poorly described in our included studies, resulting in the majority of studies having unclear risk of detection bias. Eleven studies did not blind their outcome assessors (assessed as high risk of bias), resulting in only eight studies having low risk of detection bias.

#### 5.2.3 Incomplete outcome data (attrition bias)

Attrition bias refers to the biasing effect of study participants, or study participant data becoming unavailable during the study. This bias can be counteracted by keeping accurate records of participants who drop out of the study, and by using intention‐to‐treat analysis so that drop‐outs do not have a biasing effect on final results. Nineteen studies had high risk for attrition bias, while 11 studies had unclear risk of bias on this item.

#### 5.2.4 Selective reporting (reporting bias)

This bias refers to the selection of a subset of the original recorded outcomes, on the basis of the results, for inclusion in publication. Evidence of this bias was assessed by examining studies for an existing protocol. Among included studies, only four studies had high risk of bias on this item.

#### 5.2.5 Other potential sources of bias

We considered publication bias and funding source bias as other potential sources of bias. Seventeen studies had unclear or high risk of the presence of these other biases.

### 5.3 Effects of interventions

#### 5.3.1 Permanent supportive housing

##### Primary outcome: Housing stability

**Figure 5:**
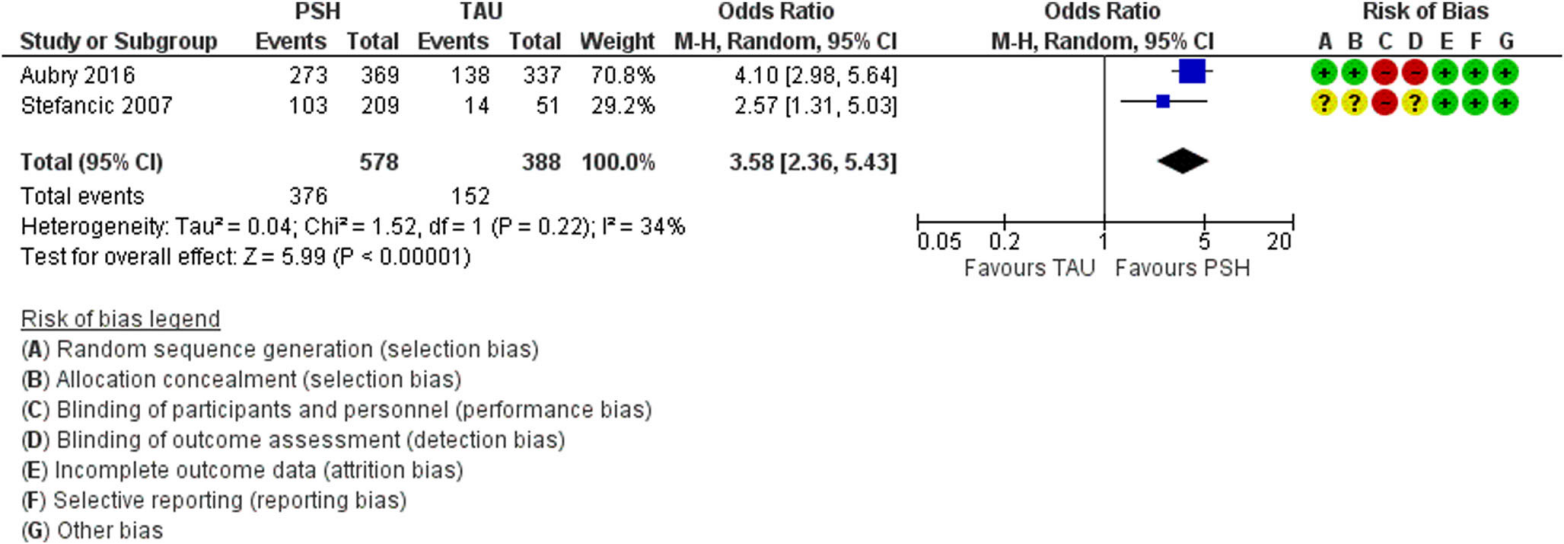
(Analysis 1.1) Figure 3. Forest plot of meta‐analysis comparing PSH to TAU on number of days in stable at 18 months or later. PSH, permanent supportive housing; TAU, treatment as usual

The effect of PSH on housing stability was examined in 15 studies (41 citations) (Aubry et al., 2016; Cherner et al., 2017; Goldfinger et al., 1999; Hwang et al., 2011; Lipton et al., 1988; Martinez & Burt, 2006; McHugo et al., 2004; Rich & Clark, 2005; Sadowski et al., 2009; Siegel et al., 2006; Stefancic & Tsemberis, 2007; Stergiopoulos et al., 2015; Stergiopoulos et al., 2019; Tsemberis et al., 2004; Young et al., 2009). Ten studies were from the United States (Goldfinger et al., 1999; Lipton et al., 1988; Martinez & Burt, 2006; McHugo et al., 2004; Rich & Clark, 2005; Sadowski et al., 2009; Siegel et al., 2006; Stefancic & Tsemberis, 2007; Tsemberis et al., 2004; Young et al., 2009) and 5 were from Canada (Aubry et al., 2016; Cherner et al., 2017; Hwang et al., 2011; Stergiopoulos et al., 2015; Stergiopoulos et al., 2019). Between the 15 studies, follow‐up ranged from 6 months to up to 6 years. Eight studies were RCTs, 1 was an extension of a multicentre RCT, 5 were quasi‐experimental trials and 1 was a controlled before‐and‐after study. Almost all trials included mental illness within the study's inclusion criteria, and many participants also had comorbid substance‐abuse disorders. Due to heterogeneity in the outcome measures and the time points measured, it was not possible to complete a meta‐analysis of all of the studies. However, data analysis from the Canadian At‐Home Chez‐Toi and US Pathways trials were pooled and is presented in Analysis 1.1 (see Figure 5).

Overall, most studies (*n* = 10) demonstrated significant short‐ and/or long‐term benefits of PSH on housing stability (Aubry et al., 2016; Cherner et al., 2017; Lipton et al., 1988; Martinez & Burt, 2006; Sadowski et al., 2009; Stefancic & Tsemberis, 2007; Stergiopoulos et al., 2015; Stergiopoulos et al., 2019; Tsemberis et al., 2004; Young et al., 2009). One study showed that PSH had a benefit not only in improving the number of days spent in stable housing but also in similar improvements in residential stability over time, compared to treatment as usual (TAU). Of note, however, is that this study did have an unexpectedly large number of participants who were already in stable housing prior to enrolment in the study (Hwang et al., 2011). Of the 15 studies evaluating housing stability, one found no difference with supportive housing (Siegel et al., 2006) and another favoured staffed group homes over independent living, though only for minority groups (Goldfinger et al., 1999). An additional study found differing results, based on interaction with gender (Rich & Clark, 2005). In one study that examined hybrid models of housing, integrative services were found to have improved residential stability compared to parallel housing (McHugo et al., 2004).

Pooled data from the multicity Canadian At Home‐Chez Toi (*n* = 2148) and US Pathways (*n* = 225) trials found that an increased number of participants remained in stable housing within the PSH intervention group, compared to TAU, with an OR of 3.58; 95% CIs, 2.36, 5.43 (GRADE certainty of evidence: Moderate) (Aubry et al., 2016; Tsemberis et al., 2004). Both trials were RCTs, enlisting participants with serious mental illness, and were based on the “Pathways to Housing” Housing First (HF) Model, which involved providing participants with scattered‐site apartments alongside ACT or ICM (Aubry et al., 2016). The At Home‐Chez Toi initiative found that over the 2‐year follow up period, participants in the intervention arm spent 73% of the time in stable housing, compared to 32% in the TAU group (Aubry et al., 2016). The individuals who obtained stable housing also moved into housing more rapidly than the TAU participants (72.9 vs. 219.7 days, AAD = 146.4, CI, 118.0–174.9, *p *< .001). Long‐term analysis of a single site of the At Home‐Chez Toi study, in Toronto, Canada, found that the number of days spent stably housed remained significantly higher in participants in the HF groups than participants in the TAU groups, at all time points, with a median duration of follow up of 5.4 years (Stergiopoulos et al., 2019). The Pathways to Housing Project, based in New York City, similarly found that participants in the intervention group experienced faster reductions in their homelessness statuses and increased their housing stability relative to participants in TAU (F‐4137 = 10.1, *p *< .001; *F* 4137 = 27.7, *p *< .001) over a 2‐year follow‐up period, with an approximately 80% housing‐retention rate. Over 90% of participants in this study had coexistent SUDs (including alcohol) in conjunction with severe mental illness (Tsemberis et al., 2004).

Several other studies also showed improvements in housing status with supportive housing compared to TAU (Cherner et al., 2017; Lipton et al., 1988; Martinez & Burt, 2006; Sadowski et al., 2009; Stefancic & Tsemberis, 2007; Young et al., 2009). Among homeless individuals (*n* = 236) with a high prevalence of mental illness (86%) and SUDs (91%) in a study out of San Francisco, 81% of participants remained in supportive housing for at least 1 year, 63% for at least 2 years, and 48% for at least 3 years (Martinez & Burt, 2006). A Canadian study in Ottawa found improved rates of housing retention at 2 years in the HF Group (76% vs. 50.8%, *p *< .001) in participants with problematic substance use (*n* = 178), with the HF intervention group demonstrating higher housing stability and decreased time to move into housing, compared to the TAU group (MD −68.73 days, 95% CI, −125, −12.08, *p *< .05) (Cherner et al., 2017). However, another Canadian quasi‐experimental study from Toronto (*n* = 112) had mixed findings; participants in supportive housing had spent significantly more days in stable housing in the 6 months prior to the study, compared to the TAU group (*F*1,87 = 15.65, *p* < .01), but residential stability over time improved equally between the two groups. A large number of participants had already been in stable housing before their study enrolment and independent assignment into groups; only five participants remained homeless throughout the study period (Hwang et al., 2011).

In contrast, some studies either showed no difference between PSH or favoured more traditional or a continuum of interventions. Goldfinger et al., (1999) compared the provision of independent housing to staffed group‐home sites and reported a reduction in days spent homeless for staffed group homes over the independent living group, though only for minority groups (Goldfinger et al., 1999). Another quasi‐experimental study found no significant difference in the percentage of tenants in initial housing placement (*n* = 157) at 6, 12 and 18 months, between supportive housing versus community residences reliant on sobriety as a precondition for housing (Siegel et al., 2006). McHugo et al. studied two hybrid approaches to housing, “parallel housing” (resembling supportive housing + ACT) and “integrated housing” (resembling the traditional continuum model, whereby decisions regarding housing are influenced by clinicians), due to cited practical considerations, such as the shortage of safe low‐income housing. Within the parallel‐housing model, ACT teams helped participants to find affordable low‐income housing but did not have control over the housing supply itself. Similar to the PSH model, housing services were not dependent on participant's mental‐health treatment. Within the integrated housing model, a single agency provided both comprehensive mental health and controlled housing but did not necessarily require scattered‐site accommodation; congregate settings were felt to be appropriate for some patients. In this study, though both approaches reduced the incidence of participants obtaining functional housing and increased their time in stable housing, the integrated‐housing approach showed benefits over the parallel‐housing‐services group, in terms of the proportion of days of functional homelessness (group × time F value 0.56, *d* = −0.52, *p *< .05) and days in stable housing (group × time *F* value 1.38, *d* = 0.51) (*p* < .05) (McHugo et al., 2004).

###### Effect of gender

Interestingly, McHugo et al. found that the effect of the group on stable housing interacted with the effect of gender (*F* 1,107 = 8.32, *p* = .005,) with males in the parallel‐housing‐services group spending significantly less time in stable housing compared to other groups for whom the outcomes were all approximately equal, specifically for females in parallel housing and males and females in the integrated housing services (McHugo et al., 2004).

The gender effect was also explored by Rich and Clark (2005). In contrast to the above study by McHugo et al, this quasi‐experimental study found that homeless men within the comprehensive housing programme actually improved their time in stable housing (similar to PSH model), as compared to those in specialised case management. Men in the comprehensive housing programme increased their number of days in stable housing by 76 out of an average of 180 days, whereas males in specialised case management increased their housing by only 37 days (a difference of 39 days). Women significantly improved their homelessness in both programmes, but women in the specialised case management had more time stably housed than women in the comprehensive housing programme, likely due to women in comprehensive housing spending more time in psychiatric hospitals (Rich & Clark, 2005).

###### Adolescents

Two subgroup analyses of the At Home‐Chez Toi study were presented. One subgroup analysis, adjusting for study city and ethnoracial and Aboriginal status, found that homeless youth aged 18–24 in the intervention arm were stably housed for a mean of 65% of days, compared to 31% for the TAU youth (*p* < .001) (Aubry et al., 2016). The difference in the changes in the mean for housing stability for those aged 18–24 years compared to adults more than 24 years old was nonsignificant. Another analysis comparing older (50 years or older) and younger (18–49 years old) homeless adults demonstrated that HF had a similar effect of housing stability on those in the older and younger age groups at 24 months, compared to the TAU group (Aubry et al., 2016).

###### Substance Use

The evidence was mixed for the effects of substance use on housing retention. Some studies found there was no relationship between SUD and housing stability (Aubry et al., 2016; Martinez & Burt, 2006) whereas one study found it correlated with more days homeless (Goldfinger et al., 1999) (though this may be because of a relatively small number of participants, in the sample, without SUD). A subanalysis of the multicity Canadian At Home‐Chez Toi study found that people with SUD spent less time in stable housing in both the HF and TAU groups but that the HF initiative improved housing stability equally among participants with and without SUD (OR 1.17, 95% CI, −0.77, 1.76) (Aubry et al., 2016). A 5‐site (4 city) study in the United States found that, depending on the treatment site, the effect of intervention varied with substance abuse (Goldfinger et al., 1999).

##### Outcome 2: Mental health

Eleven studies examined the effects of PSH on mental health (Aubry et al., 2016; Cherner et al., 2017; Hwang et al., 2011; Lipton et al., 1988; McHugo et al., 2004; Rich & Clark, 2005; Sadowski et al., 2009; Siegel et al., 2006; Stergiopoulos et al., 2015; Tsemberis et al., 2004; Young et al., 2009). Only one study showed a benefit of PSH, over the TAU, on mental‐health outcomes (Rich & Clark, 2005) In three studies, psychiatric symptoms were somewhat improved in the comparison group, compared to supportive housing (Aubry et al., 2016; Cherner et al., 2017; Young et al., 2009). One study examined hybrid housing models and found improved psychiatric symptoms in integrated housing, compared to parallel housing (McHugo et al., 2004).

Most studies (*n* = 5) found no significant differences between PSH and TAU (Hwang et al., 2011; Lipton et al., 1988; Sadowski et al., 2009; Stergiopoulos et al., 2015; Tsemberis et al., 2004). Some studies (*n* = 2) found that mental‐health symptoms decreased over time for both groups, even though there was no group difference between intervention arms (Sadowski et al., 2009; Tsemberis et al., 2004), while two studies (of which one was a moderate‐needs population of the At Home‐Chez Toi study) did not find any significant differences, either with time or between groups (Hwang et al., 2011; Stergiopoulos et al., 2015). One study reported that participants in PSH experienced fewer psychiatric symptoms over time (*p *< .06) (Rich & Clark, 2005). However, significant differences between the groups, on these measures at baseline, suggest that their initial values may have accounted for these differences.

A few studies (*n* = 3) found some improved mental‐health symptoms in the comparison group, as opposed to supportive housing (Aubry et al., 2016; Cherner et al., 2017; Young et al., 2009). The multicity At Home‐Chez Toi trial found that though there were improvements in mental‐health symptoms and psychological integration in both groups (pooled SMD 0.70 and pooled SMD 0.53 respectively), the TAU had a small group benefit at final follow‐up (ASMD, 0.17; CI, 0.05–0.30; *p *= .01) (Aubry et al., 2016). Another 2‐year study of homeless adults with problematic substance use similarly found improved mental‐health symptoms in the TAU group at the end of the study (*p* < .01) (Cherner et al., 2017).

There may be a differential effect of the intervention, based on the type of mental‐health symptom. A 2‐year quasi‐experimental study using the 53‐item Brief Symptom Inventory, found that both interventions similarly improved symptoms in the following areas: psychoticism, depression, anxiety, obsessive‐compulsive disorder, interpersonal sensitivity and phobic anxiety (Young et al., 2009). However, greater improvements in somatisation, paranoid ideation and global mental‐health‐symptom severity was noted in the “Comprehensive, Continuous Integrated System of Care (CCISC)” model, (an intervention that is based out of a traditional substance‐abuse treatment agency), as compared to the ACT and supportive‐housing (ACT‐SH) intervention. Of note, however, is that compared to the ACT‐SH intervention, the residential CCISC programme had a higher intensity of mental‐health interventions, with on‐site services designed to address mental‐health symptoms in a population with severe coexisting mental‐health and SUDs (Young et al., 2009).

In one published study with no data reported, the effect on mental health was unclear (Siegel et al., 2006).

McHugo et al's study that compared two hybrid approaches to housing, “parallel housing” (resembling supportive housing + ACT) and “integrated housing” (resembling the traditional model) found that participants in the integrated‐housing model had less severe psychiatric symptoms during the 18 month follow‐up period as reported by the Colorado Symptom Index (CSI) (McHugo et al., 2004).

The At Home‐Chez Toi study also published an analysis of past‐month suicidal ideation over a 2‐year follow‐up period. In both groups suicidal ideation decreased from baseline (*p* < .001), though there was no differential effect of the intervention arm compared to TAU. There was also no significant difference between groups in the number of suicide attempts (HF 11.9%, TAU 10.5% (Aubry et al., 2016).

###### Gender

One study found that changes in psychiatric symptoms were not dependent on the participant's gender (Rich & Clark, 2005).

###### Adolescents

A subgroup analysis of the At Home‐Chez Toi study, comparing younger (18–49 years old) and older (>50 years old) homeless adults, found that older adults in the intervention arm experienced significantly more improvement from baseline to 24 months, compared to the TAU group, in both the mental‐component summary score of the SF‐12 (+3.82, 95% CI, 0.46–7.19) and the severity of mental‐health symptoms (−3.39, 95% CI, −6.24 to −0.54). (Chung et al.), as measured by the modified Colorado Symptom Index (Aubry et al., 2016). Another subgroup analysis of the same trial showed no significant group x time interaction in mental‐health symptoms, also using the SF‐12 Mental Health and Colorado Symptom Index tools, in the HF vs the TAU groups among youth aged 18–24 years (Aubry et al., 2016).

##### Outcome 3: Quality of life

The subjective quality of life was examined in eleven studies (Aubry et al., 2016; Cherner et al., 2017; Goldfinger et al., 1999; Hwang et al., 2011; McHugo et al., 2004; Rich & Clark, 2005; Sadowski et al., 2009; Siegel et al., 2006; Stergiopoulos et al., 2015; Stergiopoulos et al., 2019; Tsemberis et al., 2004). The data on the differential benefits of the HF versus the comparison groups was mixed. Some condition‐specific quality‐of‐life parameters may be improved with HF strategies. The housing approach may have a gender‐related effect, though this requires more research.

The At Home‐Chez Toi RCT found that the quality of life of both groups improved over time, with a moderate to large effect size (pooled SMD 0.76). HF participants improved more rapidly in the 1st year and had higher average scores over the study period (ASMD, 0.15; *p* < 0.01, CI, 0.04–0.24) but the difference narrowed over time and was no longer significant at the final interview (21 or 24 months) (ASMD at the final interview, 0.05; CI, 0.08–0.18, *p* = .43) (Aubry et al., 2016). Additional exploratory analyses showed that although there was no significant improvement over time in the generic quality of life via the EuroQOL 5 dimensions (EQ‐5D) visual analogue scale, within the HF intervention group there was a significant improvement in the condition‐specific quality of life from the baseline to 24 months, specifically the total condition‐specific quality‐of‐life measures and the subscales relating to leisure, living and safety, as reported from the Lehman Quality of Life Interview (QOLI‐20) index. The family domain of the condition‐specific quality of life had a significant 3‐way interaction with the treatment x the time x the site, due to the differing treatment effects by site at 24 months (Stergiopoulos et al., 2015). A single‐site extension of this study, with a median duration of follow‐up of 5.4 years, showed no difference in either the generic or disease‐specific quality of life between the HF and TAU groups. However, for this long‐term analysis, it is notable that within the disease‐specific QOL, only the global quality of life (“How do you feel about your life as a whole”) within Lehman's 20 item QOL interview was analysed (Stergiopoulos et al., 2019).

Two other studies reported improvements in quality of life in the intervention arm, compared to TAU (Hwang et al., 2011; Tsemberis et al., 2004). Hwang et al., 2011 assessed the quality of life over 18 months, with the EQ‐5D and the Lehman Brief Quality of Life Interview, of homeless individuals (*n* = 112) in Toronto, Canada, with severe and persistent mental illness. This study also found some improvements in the quality of life that were independent of the housing group, assessed via Lehman's Brief Quality of Life interview; these improvements were specifically in general life satisfaction (*p* = .02), satisfaction with finances (*p* < .01) and satisfaction with safety (*p* < .01). However, the HF intervention arm had additional significant benefits in terms of the score for satisfaction with the living situation, compared to the usual care (*p* < .01, time F, 3, 3, 261 = 47.68; group × time, F3,3,261 = 14.60, *p* < .01). Similar to other studies, there were no significant benefits between groups or with the time, as measured in the EQ‐5D quality‐of‐life assessment tool (Hwang et al., 2011). Another study also found benefits to the quality of life in the experimental arm, whereby participants who suffered from severe psychiatric illnesses had significantly improved life satisfaction in six of seven life domains within the Lehman Quality of Life scale (Tsemberis et al., 2004).

A study by Sadowski et al. (2009) noted improvements in quality of life over time compared to baseline in both groups, though it did not show any appreciable statistically significant differences between treatment arms. However, this trial used mental‐health and physical functioning subscales from the AIDS Clinical Trials Group 21‐Item Short Form instrument to determine and define “quality of life” (Sadowski et al., 2009). Schutt et al. (1997) also reported that quality of life, measured via life satisfaction, was unrelated to housing type (Goldfinger et al., 1999).

Two studies reported a benefit by comparison group over HF strategies (Cherner et al., 2017; McHugo et al., 2004). In assessing the effect of HF on homeless adults with problematic substance use, Cherner et al., 2017 found that the comparison group reported a higher quality of life at 24 months than the HF clients (*d *= −0.38; 95% CI, −0.74, −0.02; *p *< .025), though both groups had an improvement in their total score for quality of life from the baseline. McHugo et al. (2004) investigated the effect of two hybrid approaches to housing for adults with severe mental illness:“parallel housing” (resembling supportive housing + ACT) and “integrated housing” (resembling the traditional continuum model). Improvements over time for general life satisfaction were demonstrated over the study period for both populations, though the integrated‐housing‐intervention arm had a significant additional group benefit (F 6.35, *p* < .05) (McHugo et al., 2004).

The effect of housing intervention on quality of life was unclear in one study (no data reported) (Siegel et al., 2006).

###### Effect on gender

One study that examined hybrid approaches to housing investigated the interaction between gender and housing. At the end of the study, male and female participants in integrated housing services and female participants in supportive housing reported similar levels of life satisfaction whereas, in comparison, males in the parallel housing services had less satisfaction (McHugo et al., 2004).

The effect of gender on changes in perceived quality of life was unclear in one study (Rich & Clark, 2005).

###### Adolescents

In one subgroup analysis, older adults (>50 years) as compared to younger adults (18–49 years) in the HF intervention had significantly greater improvements in condition‐specific quality of life (+6.99, 95% CI, 1.39–12.59) versus the TAU over a 24‐month follow‐up period. Changes from the baseline compared to the TAU for generic quality was not statistically significant between older and younger homeless adults (Aubry et al., 2016). Another subgroup analysis of the same trial showed that in adults 24 years or younger, satisfaction with leisure (the leisure category of the QOLI‐20) at 24 months as well as total‐condition‐specific quality of life at 6 months significantly improved in the HF group compared to the TAU, though these differences were not significant in the overall treatment group × the time analysis (Aubry et al., 2016). Of note, however, is that the study size may have not been sufficient to detect differences between groups.

##### Outcome 4: Substance use

The effect of permanent housing on substance use was reported in nine trials (Aubry et al., 2016; Cherner et al., 2017; Hwang et al., 2011; McHugo et al., 2004; Rich & Clark, 2005; Stergiopoulos et al., 2015; Stergiopoulos et al., 2019; Tsemberis et al., 2004; Young et al., 2009). The results were mixed, though most studies found no difference in substance use with PSH.

Three studies reported improvements in alcohol and drug use in both groups, over time, but no differential benefit of the HF initiative (Aubry et al., 2016; Stergiopoulos et al., 2019; Young et al., 2009). The first, the multicity At Home‐Chez Toi RCT had a pooled decrease in the mean symptom count of 30% in both the HF and TAU groups, without an independent group benefit (Aubry et al., 2016). Similarly, a single‐site long‐term extension of the At Home‐Chez Toi study reported improvements in substance‐use severity in both groups over time, regardless of the intervention (Stergiopoulos et al., 2019). Another trial, a quasi‐experimental study with 163 participants with coexisting severe mental‐ and SUDs, also found reductions in substance use (rates of drug abstinence and average number of days of alcohol use in the past month) in both study groups, over time, with no significant difference between groups at 6 months (Young et al., 2009).

In addition to the lack of difference in substance use between groups, three other studies either did not report differences from the baseline or found no difference from the baseline over the study period (Hwang et al., 2011; Stergiopoulos et al., 2015; Tsemberis et al., 2004).

One study on homeless clients with problematic substance use found a benefit of the TAU group over the HF intervention, with regard to substantial or severe problems with drug use at 24 months. (OR = 0.34; 95% CI, 0.16–0.72; *p *< .01). The same study reported no group differences in problems associated with alcohol use, though both groups decreased their alcohol consumption over time (Cherner et al., 2017). In contrast, a single‐site study of the At Home‐Chez Toi trial within an ethnically diverse population in Toronto, Canada, noted that, at the end of 24 months, there was a 53% reduction in the days spent experiencing alcohol problems as well as a significant reduction in the amount of money spent on alcohol in the last 30 days (−$52.86, 95% CI, −104.29 to −1.43) in the HF‐ICM group compared to the TAU group. Secondary analysis showed a significant reduction in the number of days experiencing alcohol problems that was higher in the foreign‐born HF compared to Canadian‐born participants (ratio of rate ratios = 0.19, 95% 0.04‐0.88) (Stergiopoulos et al., 2015).

Hybrid approaches to housing (“integrative” versus “parallel” housing) were investigated in a single study. It found no statistically significant difference in the reported days of alcohol use or reported days of drug use both between groups as well as over time. Of note, analysis for this outcome had a lower statistical power as it was restricted to individuals who reported use at the baseline (McHugo et al., 2004).

###### Gender

The interaction between gender, type of interaction and days of alcohol use and days of illegal drug use was not significant in one study, though there was a trend for males in the specialised case‐management programme to have higher scores on those measures (Rich & Clark, 2005).

###### Adolescents

The severity of substance‐use problems was not statistically significant between older (>50 years) and younger (18–49 years) adults, for the HF compared with the TAU participants after 24 months, in one subgroup analysis (Aubry et al., 2016). Another publication depicting a subgroup analysis of the same study found no significant group × time interaction in substance use in the HF vs TAU groups in adolescents aged 18–24 years (Aubry et al., 2016).

##### Outcome 5: Hospitalisation

Ten studies examined hospitalisations data (Aubry et al., 2016; Goldfinger et al., 1999; Hwang et al., 2011; Lipton et al., 1988; Martinez & Burt, 2006; McHugo et al., 2004; Sadowski et al., 2009; Siegel et al., 2006; Stergiopoulos et al., 2015; Tsemberis et al., 2004) In multiple studies, HF strategies reduced the number of days in hospital and inpatient admissions.

The multicity At Home‐Chez Toi RCT found that both the HF and TAU groups had similar decreases in the number of days hospitalised and emergency department (ED) visits, with a pooled decrease of 62% and 53%, respectively. The HF group initially showed a significant reduction in ED visits at 6 months (*p* = .007), though this difference narrowed at the end of the study period (21 or 24 months, incident rate ratio = 0.80; CI, 0.65–1.00; *p* = .05). The rates of hospital admissions were not significantly different between the two groups, though the risk of 1 or more hospitalisations varied by site; for example, Site C showed 1.67 (95% CI, 1.08–2.59) (Aubry et al., 2016; Stergiopoulos et al., 2015).

Multiple other trials also found benefits of the HF intervention over the TAU (Lipton et al., 1988; Martinez & Burt, 2006; Sadowski et al., 2009; Tsemberis et al., 2004). A RCT from Chicago, Illinois (*n* = 405), found that after adjusting for the baseline characteristics and using zero‐inflated negative binomial models, there were statistically significant 29% reductions in hospital days and hospitalisations and a 24% reduction in ED visits in the HF group compared to the usual care (Sadowski et al., 2009). PSH also reduced the likelihood of being hospitalised (from 19% to 11%), the mean number of inpatient admissions per person (from 0.34 to 0.19 admissions per resident) and total number of inpatient admissions (45% decline, from 80 to 44) in a sample of 236 adults (most of whom had a dual diagnosis of mental illness and SUD), in San Francisco, California (Martinez & Burt, 2006). In this trial, emergency‐room visits from year 1 to 2 were also reduced in the intervention group compared to the control group (regression coefficient *B* = −7.07, SE = 1.29; *p* < .01 (Martinez & Burt, 2006). In another US‐based trial, a Pathways to Housing RCT, the control group spent more time in hospital compared to the HF group (F1195 = 74, *p* < .01) over the 2‐year follow‐up period. (Tsemberis et al., 2004). There were also statistically significant differences in the number of days hospitalised in a study by Lipton et al. (1988), where the experimental group spent a mean of 55 nights or 15% of the study year in a hospital compared to the control who spent a mean of 168 nights or 46% of the year in hospital (*t* = 3.74, df = 20, *p* < .001), with no differences in the number or length of readmissions to psychiatric hospitals (Lipton et al., 1988).

Two studies found no statistically significant differences in health‐care utilisation (including both inpatient and outpatient care) between supportive housing and usual care over time (Hwang et al., 2011; Goldfinger et al., 1999). One study (Hwang et al., 2011) reported health‐care utilisation by participants who self‐report, whereas the second reported service use via case management records from community mental‐health centres and affiliated clinics, and the Massachusetts Department of Mental Health and Medicaid for inpatient admissions (Goldfinger et al., 1999).

###### Adolescents

A sub‐group analysis of the At Home‐Chez Toi study found no significant group x time interaction in adolescents aged 18–24 years between the HF and TAU groups, for the number of ED visits or percentage of persons who visited a clinical provider (Aubry et al., 2016).

##### Outcome 6: Employment

One study, the At Home‐Chez Toi study reported the effect of PSH on employment outcomes (Aubry et al., 2016). Here, 2148 people who were homeless and were diagnosed with a mental illness were recruited from five Canadian sites. They were subsequently grouped into “moderate” or “high needs” groups, where participants with high needs received ACT and those with moderate needs received ICM, with a median follow‐up after 745 days. Compared with the control group of moderate‐needs participants, the HF ICM participants had lower odds of obtaining employment. Over time, the odds of obtaining employment in both the HF‐ICM and ACT groups improved compared to the control, but despite this, the rates of employment in the HF group never exceeded those of the control group. For the ACT high‐needs group, the odds of obtaining employment was not statistically significant compared to the control group. Men, younger participants and those employed at the baseline had increased odds of obtaining competitive employment in both the HF‐ICM and HF‐ACT groups compared to the control. In the HF‐ICM group, individuals with more than 12 years of education and a higher MCAS (Multinomah Community Ability score) also had a higher chance of obtaining competitive employment. Secondary employment outcomes, such as job tenure in days, hours worked per week and hourly wage, were not statistically significant between the HF and control groups. Interestingly, the low rates of employment in the study were in contrast to the high percentage (74%) of individuals who stated a desire to return to employment (Aubry et al., 2016).

##### Outcome 7: Income

The At‐Home‐Chez Toi study also examined the effect of HF on income. There were no statistically significant differences between Housing First and control‐group income from various sources over time (Aubry et al., 2016). These results were consistent with a study by Rich and Clark (2005), where the three‐way interaction between time, gender and type of intervention for income in average dollars/month was also not statistically significant at the 12‐month follow up (Rich & Clark, 2005).

#### 5.3.2 Income‐assistance intervention

##### Outcome 1: Housing stability

Ten eligible studies assessed the effectiveness of income‐assistance interventions on housing stability. Five studies assessed the effectiveness of income assistance in the form of rental subsidies (Gubits et al., 2018; Hurlburt et al., 1996; Pankratz et al., 2017; Rosenheck et al., 2003; Wolitski, 2009). For people with acquired immunodeficiency syndrome (AIDS), Wolitski et al. (2009) examined a federal programme that provided immediate housing opportunities in the form of rental assistance along with case management. They found that this programme was associated with a higher percentage of participants housed in their own place up to 18 months into the programme, compared with customary housing services and case management (at 6 months: OR = 6.20; 95% CI, 4.18–9.20; *p* < .0001; at 18 months: OR = 4.60; 95% CI, 3.10–6.83; *p *< .0001) (Wolitski 2009). Participants receiving priority access to housing vouchers along with case management demonstrated more days stably housed in the past 90 days (over 3 years: MD = 8.58; *p *< .004; GRADE Certainty of Evidence: Low) (Rosenheck et al., 2003) and were significantly more likely to achieve stable independent living arrangements than the controls who only received case management (over 24 months: 57.5% vs. 30.4%; OR = 3.09; 95% CI, 2.00–4.76; *p *< .0001) (Hurlburt et al., 1996).

Five studies investigated the effect of income‐assistance interventions delivered in alternative formats and which included financial‐empowerment education, compensated‐work therapy, and IPS (Booshehri, 2017; Ferguson, 2018; Forchuk et al., 2008; Kashner, 2002; Poremski et al., 2015). A 28‐week financial‐empowerment education programme was compared with employment training for low‐income families with children. No significant differences in the housing conditions were identified between the study and the control groups after the intervention was undertaken (Ferguson, 2018). A compensated work‐therapy programme (CWT) that combined supported employment with stepwise clinician‐run therapy offered to homeless veterans with substance dependency was compared with standard access to comprehensive rehabilitation, addiction, and psychiatric and medical services (Kashner, 2002). A significantly reduced odds of an episode of homelessness was detected in the study group participants who were offered supported employment, compared with the controls (OR = 0.1; 95% CI, 0.1–0.3; *p *= .001; GRADE certainty in evidence is low). However, no significant improvement on housing stability was observed among the study group of homeless youth experiencing mental illness who received extra IPS, through regular meetings with employment specialists, on top of the 20‐month social‐enterprise intervention programme that included the same vocational skills classes and case management services that were also offered to the controls (Ferguson, 2018). The diverse study designs and participant profiles, along with smaller sample sizes, might contribute to the inconsistency and inconclusive effects measures. Forchuk et al. (2008) examined the effect of adding fast‐tracked income support on top of housing assistance for individuals who were discharged from psychiatric wards to shelters and who were precariously housed. A significant improvement in the percentage of participants attaining independent housing at 6 months was detected in the study group when compared with the controls who only received the usual care with housing support by referral (100% vs. 14.2%; OR = 65; 2.23,1887.35; *p* < .001). Income assistance in the form of rental subsidies was consistently associated with better housing stability among the eligible studies.

##### Outcome 2: Mental health

Six studies assessed the impact of income‐assistance intervention on mental health (Booshehri, 2017; Ferguson, 2018; Gubits et al., 2018; Kashner, 2002; Rosenheck et al., 2003; Wolitski, 2009). The effect of income‐assistance interventions on mental health varied depending on the intervention characteristics. Immediate rental assistance with case management for people with AIDS led to improved depression symptoms and perceived stress level at 6 months but it did not show a consistent effect on self‐reported mental‐health statuses compared to the usual care (Wolitski, 2009). Permanent housing subsidies for homeless families with children aged 15 years or younger was associated with decreased psychological distress compared with the usual access to community housing services (Gubits et al., 2018). However, housing vouchers combined with case management for homeless veterans with psychiatric or addiction/alcohol disorders did not generate significant improvements in the mental‐health statuses of the participants (Rosenheck et al., 2003). One study that assessed IPS programmes for homeless youth reported higher self‐esteem scores (23.00(SD = 4.88) vs. 21.50(SD = 5.91), *p* < .05), less self‐reported depression symptoms (4.85 vs. 6.71, *p* < .10), and less self‐reported attention‐deficit hyperactivity (ADHD) (5.54 vs. 7.06, *p* < .01) symptoms (Ferguson, 2018). Nevertheless, financial‐empowerment education programmes for low‐income families, compared to existing employment training, did not provide additional benefits on the ability to manage stress or the presence of depression symptoms (Booshehri, 2017). The CWT programme offered to homeless veterans with substance dependence, in one study, was not associated with significantly different mental‐health status when compared with access to standard rehabilitation, and addiction and mental‐health services (Kashner, 2002).

##### Outcome 3: Quality of life

Three studies examined the effect of income‐assistance intervention on quality of life (Ferguson, 2018; Pankratz et al., 2017; Rosenheck et al., 2003). One study showed that additional rent assistance with a top‐up of 350 Canadian dollars led to significant improvements in quality of life at 6 months compared to the usual housing support (Pankratz et al., 2017). For veterans with a diagnosis of a major psychiatric disorder or an alcohol/drug disorder who were homeless or who had been homeless for 1 month or longer, priority access to housing vouchers did not provide additional benefits compared with the usual case management (Rosenheck et al., 2003). A social‐enterprise intervention that provided vocational and business skills classes was not associated with an increased satisfaction of life quality compared to SCM and support (Ferguson, 2018).

##### Outcome 4: Substance use

our studies examined the effects of income‐assistance intervention on substance use (Gubits et al., 2018; Kashner, 2002; Poremski et al., 2015; Rosenheck et al., 2003). For homeless veterans with substance dependence, CWT immediately reduced the consumption of alcohol and drugs within the first 3 months (*β* = −44.7%; 95% CI, −69.7% to −19.7%; *t* = 3.51; *p* = .001) (Kashner, 2002). The addition of priority access to housing vouchers for veterans with a diagnosis of a major psychiatric disorder or an alcohol/drug disorder, compared to existing case management, led to decreased days of drinking to intoxication (1.46 vs. 1.95, *t* = 0.73, *p* = .0053) and fewer days of alcohol use over the 3‐year study period (Rosenheck et al., 2003). However, no significant association was identified between priority access to housing subsidies and differences in the scores of the Addiction Severity Index (ASI) (Rosenheck et al., 2003). For homeless families with children aged 15 or younger, rental subsidies without case management did not lead to decreased use of substance compared to the usual community housing services (Gubits et al., 2018).

##### Outcome 5: Hospitalisation

Three trials evaluated the impact of income‐assistance intervention on hospitalisations (Kashner, 2002; Poremski et al., 2015; Wolitski, 2009). CWT for homeless veterans with substance dependence significantly decreased the number of mean inpatient days in medicine in 1 year (2.6 days vs. 0.3 days, *t* = 3.02, *p* = .003) but led to no significant difference in mean patient days in psychiatry or addiction wards (Kashner, 2002). The IPS programme, which aids in obtaining and maintaining competitive employment for individuals experiencing mental illness who are precariously housed or have been homeless for at least seven nights in Canada, did not show a decrease in the number of days spent in a hospital or correctional facility at a 3‐month interval (Poremski et al., 2015). Rental assistance combined with case management for people with AIDS was not associated with a decreasing trend in emergency room visits along an 18‐month study interval (Wolitski, 2009).

##### Outcome 6: Employment

Five studies assessed the effectiveness of income‐assistance interventions on employment outcomes (Booshehri, 2017; Ferguson, 2018; Gubits et al., 2018; Poremski et al., 2015; Rosenheck et al., 2003). Both long‐term housing subsidies for homeless families with children (Gubits et al., 2018) and housing subsidies with case management for homeless veterans with psychiatric or alcohol/drug disorders (Rosenheck et al., 2003) were unable to demonstrate a positive impact on employment. Likewise, the financial empowerment programme was not associated with an increase in employment status, whereas the standard job training and search programme offered to the controls demonstrated an increase in employment status (Booshehri, 2017). Furthermore, the reported effects of the IPS programme on employment outcomes also appeared inconsistent. Compared with SCM and service for homeless individuals with mental illness, the IPS programme contributed to significant improvements in obtaining employment during the 8‐month period of high‐intervention fidelity (OR = 2.418; 95% CI, 1.133–5.157; *p* = .02) (Poremski et al., 2015). On the other hand, the IPS programme with case management offered to homeless youth did not lead to improvements in employment when compared with the social‐enterprise intervention that included vocational‐ and business‐skills acquisition (Ferguson, 2018).

##### Outcome 7: Income

Six studies reported on the effectiveness of income‐assistance interventions on income outcomes (Booshehri, 2017;Ferguson, 2018; Gubits et al., 2018; Pankratz et al., 2017; Poremski et al., 2015; Rosenheck et al., 2003). For homeless families with a child aged 15 years old or younger, long‐term rent subsidies were associated with an increased percentage of households acquiring food security (61.1% vs. 51.5%, SE = 3.5, *p* < .01 at 37 months) (Gubits et al., 2018). However, self‐reported family earned income did not show a difference between the study and the control group. Homeless veterans with psychiatric or alcohol/drug disorders who received housing subsidies in addition to case management were not associated with increases in total income per month compared with the controls who only received case management (656 vs. 684, *t* = 1.56; *p* = 0.12) (Rosenheck et al., 2003). An 8‐month IPS to provide specialised assistance on employment for homeless individuals with mental illness did not result in a difference in wages per hour compared with the SCM (Poremski et al., 2015). Another IPS for homeless youth was compared with a social enterprise intervention that focused on vocational‐ and business‐skills acquisition over a 20‐month study period and was not associated with an increase in weekly income (Ferguson, 2018). In addition, a 28‐week financial‐empowerment education programme offered for low‐income families with a child and housing insecurity did not give rise to an increase in hourly earnings or relief of economic hardship when compared with standard weekly job training and search activities (Booshehri, 2017). Overall, the income‐assistance interventions were not associated with noticeable changes in income outcomes among participants in the eligible studies.

#### 5.3.3 SCM and peer support interventions

##### Standard case management

###### Outcome 1: Housing stability

Nine trials reported on the effectiveness of SCM treatment on housing stability outcomes (Conrad et al., 1998; Graham‐Jones et al., 2004; Hurlburt et al., 1996; Lapham et al., 1996; Nyamathi et al., 2016; Sosin et al., 1995; Towe et al., 2019; Upshur et al., 2015; Weinreb et al., 2016). The positive effect of SCM on housing stability in homeless (addicted) male veterans (*n* = 358) showed significantly fewer homeless nights at 12 months in the intervention group (*n* = 187), an effect that was reversed at 24 months (certainty of evidence—low) (Conrad et al., 1998). An SCM trial with enhanced or intensive housing‐placement assistance (EPHA) for homeless single adults living in HIV emergency shelters (*n* = 236) reported significantly higher percentages of participants' placements in stable housing (*p* = .02; 25% placed by 150 days vs. 243 days) (Towe et al., 2019). A three‐armed quasi controlled trial with homeless patients registered temporarily either in a health centre advocacy group or an outreach advocacy group and the TAU found no significant differences observed between groups, although a higher number of participants in one group achieved housing outcomes (Graham‐Jones et al., 2004). A four‐arm trial (*n* = 361) assessing different CM intensities in persons who are homeless or at risk of homelessness and with severe or persistent mental illness reported no significant improvement observed in their housing outcomes (Hurlburt et al., 1996). A similar study reported no significant between‐group improvement in stable housing in groups of homeless alcohol abusers, however, significantly more favourable housing‐stability outcomes were observed among programme graduates than among dropouts (Lapham et al., 1996). Nyamathi et al. (2016) found no significant between‐group differences in either the peer‐coach‐non‐comprehensive case management group (NCM) or the usual care group after 12‐months in a population of homeless men recently released from California jails and prisons. Another study on homeless women with alcohol‐use problems found no significant difference between groups on housing outcomes between Project RENEWAL intervention and TAU. While those in the intervention group spent double the time in their apartments in 6 months, women in the TAU group spent significantly fewer nights in shelters (Upshur et al., 2015). A short‐term inpatient case‐management‐only intervention study reported a significant increase in the average number of days in residential stability (Sosin et al., 1995). Finally, a 6‐month clustered evaluating integrated care model for homeless mothers (ICMHM) versus TAU reported modest but no significant housing improvements, with more than half of participants still living in a shelter (Weinreb et al., 2016).

###### Outcome 2: Mental health

Four trials reported on the effectiveness of SCM on mental‐health outcomes (Conrad et al., 1998; Nyamathi et al., 2001; Upshur et al., 2015; Weinreb et al., 2016). A three‐arm trial (*n* = 948) with a nurse‐led case management (NCM) programme, peer‐mentored programme and standard/usual care for women in emergency or sober‐living shelters reported higher levels of hostility (*p* < .001) and depression symptoms (*p* < .05) compared to those female participants receiving standard care in the NCM after 6 months (Nyamathi et al., 2001). However, no significant difference in psychological well‐being was reported between groups using the Mental Health Index (MHI‐5). Another trial in homeless addicted male veterans with 3–6 months of stays at hospital facilities (residential care) plus case management versus TAU for 21 days reported no significant differences between groups on ASI's psychiatric‐symptoms sub‐score (Conrad et al., 1998). Another 6‐month trial of guideline‐based primary care provider brief intervention and referral to the CM for ongoing follow‐up visits (*n* = 42) for homeless women with alcohol use problems found reduced odds of depression at 3 months (OR, 0.38; 95% CI, 0.14–0.99) (Weinreb et al., 2016). The women did not show improvements in their overall mental‐health statuses, with no significant between‐group differences (MD, 4.50; 95% CI, −0.98–9.98), and a significantly lower percentage of participants met depression criteria at follow‐up, (Upshur et al., 2015).

###### Outcome 3: Quality of life

Two RCTs reported the effects of different SCM treatment on groups' quality of life outcomes (Graham‐Jones et al., 2004; Nyamathi et al., 2001). A three‐armed quasi controlled trial with homeless patients registered temporarily either in a health centre or under the care of outreach advocacy groups and TAU reported better improvements than the control group on the social‐isolation dimension only Graham‐Jones et al. (2004). In contrast, in a Nottingham health profile, the outreach group achieved better improvements in participants' emotional distress and sleep subscores (Graham‐Jones et al., 2004). Both intervention groups showed improvements on different subscales in the life‐fulfilment scale, while only the outreach advocacy group improved significantly on the aggregated fulfilments scales (Graham‐Jones et al., 2004). There were no significant between‐group differences in the Delighted‐Terrible Faces scale (Graham‐Jones et al., 2004). Also, a nurse‐led case‐managed programme compared with peer‐intervention for homeless women and their intimate partners with standard care programme reported no significant differences in the quality‐of life‐outcomes (Nyamathi et al., 2001).

###### Outcome 4: Substance use

Six studies reported on substance‐use outcomes with different SCM treatments (Conrad et al., 1998; Lapham et al., 1996; Nyamathi et al., 2001; Nyamathi et al., 2016; Sosin et al., 1995; Upshur et al., 2015). Homeless male veterans with addictions problems with three to 6 months of hospital stays plus case management showed better improvements in the alcohol‐ and drug‐use composite scores, but these were short‐lived compared to TAU after 24 months (Conrad et al., 1998). A three‐arm trial reported reductions in average days of alcohol and drug consumption in a case‐management‐only group, a CM‐with‐supported‐housing group in graduates of short‐term in‐patient substance‐use programme who lacked housing (Sosin et al., 1995). Homeless alcohol abusers who were offered different intensities of SCM (*n* = 469) showed no significant between‐group differences in days of alcohol use (Lapham et al., 1996). Similarly, a three‐arm trial with homeless women and their intimate partners found no significant differences in substance‐use outcomes between groups: a peer‐mentored group, a group in a nurse case‐managed programme, and one in an SCM programme (Nyamathi et al., 2001). Another 12‐months trial on homeless men recently released from jails/prisons who were exposed to weekly peer coaching, intermediate peer coaching compared with the usual treatment did not find any significant effects on substance‐use outcomes (Nyamathi et al., 2016). An RCT with homeless women with alcohol‐use problems who received brief guideline‐based interventions from primary care providers and referrals to the CM for ongoing follow‐up visits for 6 months did not find any significant between‐group differences on alcohol‐consumption rates, percentages of completely abstained participants, or negative consequences of alcohol use, respectively (Upshur et al., 2015).

###### Outcome 5: Hospitalisation

One trial evaluated the impact of a health advocate‐SCM (with or without outreach registration) on hospitalisations over 3 months (Graham‐Jones et al., 2004). Only five percent of all participants in the study accessed the emergency department, with no significant difference between health advocacy or usual‐care groups (Graham‐Jones et al., 2004).

###### Outcome 6: Employment

Four trials reported on the effects of different SCM interventions on the employment of participants (Conrad et al., 1998; Lapham et al., 1996; Nyamathi et al., 2016; Weinreb et al., 2016). The impact of SCM interventions on the employment outcomes of homeless (addicted) male veterans (residents‐to‐case managers ratio of 10:1 and 25:1 for the residency and community follow‐up phases) reported significant improvements favouring the intervention group over the entire study period (Conrad et al., 1998). In another four‐arm trial on homeless alcohol abusers (*n* = 469) there were no significant between‐group differences in improvements in employment (Lapham et al., 1996). Also, a three‐arm trial (*n* = 600) with a nurse case‐managed programme, and peer‐mentored intervention compared to TAU in homeless women and their intimate partners reported some improvements in employment but no statistically significant differences between groups after 6 months. A 6‐month clustered trial of an integrated‐care model for homeless mothers (ICMHM) with major depressive disorder showed that participants' employment improved but there were still no significant between‐group differences (Weinreb et al., 2016).

###### Outcome 7: Income

None of the included RCTs reported on the effects of SCM on income.

#### 5.3.4 Peer support interventions

Six randomised trials examined the effect of peer support interventions among individuals with lived experience of homelessness (Corrigan et al., 2017; Ellison et al., 2020; Lapham et al., 1996; Nyamathi et al., 2001; Nyamathi et al., 2016; Yoon et al., 2017). All six trials took place in the United States and among diverse and heterogeneous cohorts of patients, preventing us from pooling effect estimates using a random effects model.

##### Outcome 1: Housing stability

Of the six trials on peer‐support interventions among individuals with lived experience of homelessness, four examined housing stability (Corrigan et al., 2017; Ellison et al., 2020; Lapham et al., 1996; Nyamathi et al., 2016). Overall, none of the trials reported any added benefit associated with receiving peer‐support interventions compared to usual care; one randomised controlled trial (Corrigan et al., 2017) examined an ethnically matched peer‐navigator programme, compared to usual care among homeless African Americans with serious mental illness, and found that even though participants in both the intervention and usual‐care groups showed decreased homelessness rates over 12 months, the between‐group difference was not significant at 12 months (Intervention: 91%, control 84%, OR = 1.84; 95% CI, 0.40–8.43; *p *= .42). Another randomised controlled trial (Ellison et al., 2020) examined peer‐specialist intervention compared to usual care among homeless veterans receiving housing vouchers and measured the number of days participants spent housed in the past month, when using housing vouchers, as well as the number of days spent housed in any community accommodation in the past month. Investigators found no significant differences between the intervention and usual care groups across 12 months (*p* > .05). Comparably, one randomised controlled trial (Lapham et al., 1996) examined peer‐manager‐delivered support services alongside temporary housing, compared to temporary housing alone, among homeless individuals with problematic alcohol use, and measured the number of days in stable housing in the past month and over 10 months, using the Personal History Form (PHF). Investigators found that assignment to interventions with a peer‐support component was not significantly associated with increased housing stability compared to assignment to temporary housing alone (the coefficient estimate for assignment to peer support with temporary housing = 11.0 (SE 1.7); the coefficient estimate for assignment to temporary housing only = 13.3 (SE 2.0); the overall model coefficient of determination R2 = 0.09; *p* > .001). Finally, one randomised controlled trial (Nyamathi et al., 2016) examined a peer‐coaching programme compared to usual care among homeless men with a history of substance use who had recently been released from county jails. This study used a structured questionnaire to measure the percentage of participants who lived on the street or in shelters over 12 months. It found no added benefit of being assigned to the peer‐support programme compared to the usual care (intervention *n* = 20 (11.4%), the control (*N *= 19 (10.1%); OR = 1.14; 95% CI, 0.59–2.23; *p *= .68).

##### Outcomes 2: Mental health

Three trials examined mental‐health outcomes (Corrigan et al., 2017; Ellison et al., 2020; Nyamathi et al., 2001) and provided mixed evidence on the effect of peer‐support interventions on participants' mental health. One randomised controlled trial (Corrigan et al., 2017) examined an ethnically matched peer‐navigator programme compared to the usual care among homeless African Americans with serious mental illness and measured psychological distress using the Texas Christian University Health Forms (TCU‐HF). Investigators found that those in the intervention group reported better improvements across 12 months (4 months: intervention *M *= 23.0 (SD 8.04), control *M *= 5.2 (SD 8.92); 12 months: intervention *M* = 18.5 (SD 4.79), control *M* = 24.4 (SD 7.93); overall group by time effect *p* < .05). As well, investigators measured emotional well‐being, using the Short‐Form 36 (SF‐36) and similarly found better improvements in the peer‐support group compared to usual care across 12 months (4 months: intervention *M* = 63.0 (SD 16.6), control *M* = 71.1 (SD 16.1), overall group by time effect *p* < .05).

Conversely, one randomised controlled trial (Ellison et al., 2020) examined a peer‐specialist intervention compared to usual care among homeless veterans receiving housing vouchers and measured symptoms of mental illness using the 24‐item Behaviour And Symptom Identification Scale (BASIS‐24) up to 12 months and found no statistically significant between‐group difference (*p* > .05). Another randomised controlled trial (Nyamathi et al., 2001) examined an ethnically matched female peer‐mentorship programme compared to usual care among homeless women and their intimate partners, measuring psychological well‐being over 6 months using the Mental Health Index (MHI‐5) and found no statistically significant improvement among those receiving peer support compared to the usual care (Regression coefficient B = −5.17 (SE 2.2); *p* = .019). Moreover, the investigators used the Brief Symptom Inventory (BSI) to examine symptoms of depression, anxiety, and hostility over 6 months and reported that women and their partners who had received the peer‐support intervention were more likely to suffer from higher levels of depression (regression coefficient B=0.68 (SE, 0.3); *p* = .01), and anxiety (regression coefficient *B* = 0.76 (SE 0.3); *p* = .008), but not hostility (regression coefficient B = 0.44 (SE 0.3); *p* = .11) compared to those who received usual care. Finally, investigators measured self‐esteem over 6 months, using the revised version of the Self‐Esteem Inventory (SEI), and found no significant improvement associated with receiving peer support compared to the usual care (regression coefficient B = −1.08 (SE 0.5); *p* = .02).

##### Outcome 3: Quality of life

Evidence on the effect of peer‐support interventions on quality of life is scarce and mixed as only two trials examined this outcome (Corrigan et al., 2017; Nyamathi et al., 2001).

One randomised controlled trial (Corrigan et al., 2017) examined an ethnically matched peer‐navigator programme, compared to usual care among homeless African Americans with serious mental illness, measured participants' generic quality of life using the Lehman's quality of life interview, and found a significant between‐group difference favouring the peer‐support arm compared to the usual care across 12 months (effect size 0.1, 0.3; range of internal consistency 0.71, 0.82, no further details). However, in another randomised controlled trial (Nyamathi et al., 2001) on an ethnically matched female peer‐mentorship programme compared to the usual care among homeless women and their intimate partners examined life satisfaction over 6 months, using a series of faces with expressions ranging from very happy to very sad, and found no significant improvement associated with receiving peer support compared to the usual care (regression coefficient B = −0.27 (SE 0.2); *p* = .072).

##### Outcome 4: Substance use

Four trials examined substance‐use outcomes (Ellison et al., 2020; Lapham et al., 1996; Nyamathi et al., 2001; Nyamathi et al., 2016) and provided mixed evidence that suggests the attenuated effect of receiving peer support on substance‐use outcomes compared to the usual care. Only one randomised controlled trial found an added benefit associated with peer support (Lapham et al., 1996). Investigators examined support services delivered by peer managers alongside temporary housing compared to temporary housing alone among homeless individuals with problematic alcohol use and measured the number of days of alcohol use in the past month and over 10 months using the Addiction Severity Index. Their assessment found that assignment to interventions that came with a peer‐support component was statistically associated with decreased days of alcohol use compared to assignment to temporary housing alone (the coefficient estimate for assignment to peer support with temporary housing = 7.4 (SE 1.1); the coefficient estimate for assignment to temporary housing only = 7.1 (SE 1.3); the overall model coefficient of determination R2 = 0.14; p > .001).

One randomised controlled trial (Ellison et al., 2020) examined a peer‐specialist intervention compared to the usual care among homeless veterans receiving housing vouchers. It used the Addiction Severity Index to measure alcohol and drug use up until 12 months and found no statistically significant between‐group difference (*p* > .05). As well, another randomised controlled trial (Nyamathi et al., 2001) examined an ethnically matched female peer‐mentorship programme, compared to usual care among homeless women and their intimate partners, and measured the use of noninjection drugs over 6 months with a minimally revised Drug History Form and found no significant decrease in drug use associated with receiving peer support compared to the usual care (regression coefficient B = −0.30 (SE 0.3); *p* = .335). Finally, a third randomised controlled trial (Nyamathi et al., 2016) examined a peer‐coaching programme compared to usual care among homeless men who had recently been released from county jails and had a history of substance use. This trial measured the percentage of participants who were using different substance drugs over 12 months, using the modified version of the Texas Christian University (TCU) Drug History form, and found no statistically significant between‐group difference for marijuana use (intervention N* *= 85 (48.0%), control N* *= 85 (45.7%); OR = 1.09; 95% CI, 0.72–1.65; *p *= .65), heroin (intervention N* *= 22 (12.4%), control N* *= 24 (12.9%); OR = 0.95; 95% CI, 0.5–1.77; *p* = .89), or stimulant substances (intervention N* *= 80 (45.2%), control *N *= 95 (51.1%); OR = 0.79; 95% CI, 0.52–1.19; *p *= .26).

##### Outcome 5: Hospitalisation

Evidence on hospitalisation was limited. Only one randomised controlled trial (Yoon et al., 2017) examining a peer‐mentorship programme compared to usual care among homeless veterans measured inpatient admissions using the VA Medical Statistical Analysis System records and found no statistically significant difference between participants who received peer support and those who received usual care (intervention *M* = 0.2 (SD 0.7), control M* *= 0.2 (SD 0.7); *t* test = 0.3; *p* = .79). As well, investigators measured the number of emergency‐department visits and found a similar nonsignificant difference (intervention M = 1.4 (SD 3.0), control M = 1.3 (SD 2.3); *t* test = −0.5; *p* = .61).

##### Outcome 6: Employment

Two trials examined employment outcomes (Lapham et al., 1996; Nyamathi et al., 2016) and provided mixed evidence on the effect of peer‐support interventions on employment. One randomised controlled trial (Lapham et al., 1996) examining support services delivered by peer managers, alongside temporary housing, compared to temporary housing alone among homeless individuals with problematic alcohol use, used the Personal History Form to measure the number of days of employment in the past month and over 10 months, and found that assignment to interventions with a peer‐support component is statistically associated with increased days of employment (the coefficient estimate for assignment to peer support with temporary housing = 7.8 (SE 1.1); the coefficient estimate for assignment to temporary housing only = 7.0 (SE 1.3); the overall model coefficient of determination R2 = 0.22; *p* < .001).

Conversely, one randomised controlled trial (Nyamathi et al., 2016) examining a peer‐coaching programme, compared to usual care among homeless men recently released from county jails with a history of substance use, used structured questionnaires to measure the percentage of participants with full‐time and part‐time employment as well as the percentage of participants who were unemployed over 12 months. It found that the peer‐support intervention did not significantly increase the percentage of participants who had full‐time employment (intervention N* *= 21 (12.0%), control N* *= 35 (18.6%); OR = 0.59; 95% CI, 0.33–1.07; *p* = .08), or part‐time employment (intervention N = 24 (13.7%), control *N *= 28 (14.9%); OR =0.90; 95% CI, 0.50–1.63; *p *= .74), nor did it decrease the percentage of participants who reported being unemployed (intervention N* *= 130 (74.3%), control N* *= 125 (66.5%); OR = 1.45; 95% CI, 0.92–2.29; *p* = .1).

##### Outcome 7: Income

No trials examined the effect of peer‐support interventions on income‐related outcomes.

#### 5.3.5 Mental health interventions

##### Assertive community treatment

###### Outcome 1: Housing stability

Five trials reported on the impact of ACT on housing stability (Clarke et al., 2000, Fletcher et al., 2008, Lehman et al., 1997, Morse et al., 1992, 1997, 2006). The effect of an ACT programme on homeless persons with mental illness found no significant between‐group difference regarding the number of days homeless in shelters; however, the ACT group spent more days in community housing compared to the TAU control group (Lehman et al., 1997). A 12‐month three‐arm trial for homeless people with severe psychiatric disorders treated with integrated‐ACT, drop‐in centre care and traditional outpatient treatment, found better improvements in housing stability in participants treated with continuous ACT than those in outpatient clinics or drop‐in centres, respectively (Morse et al., 1992). Also, a follow‐up trial on homeless persons with mental illness found better improvements in housing stability using individualised ACT, ACT with community workers and brokered case management (BCM), respectively (Morse et al., 1997). Another 24‐month trial using different ACT modalities (ACT only—ACTO, integrated ACT—IACT, nonintegrated ACT—NIACT) in homeless clients with severe mental illness and SUD reported *p* more days in stable housing at 18 months (*p *= .0021) and 24 months (*p *= .03), respectively than in the control group (Morse et al., 2006). However, there was no significant difference between ACT modalities. Also, a three‐arm ACT trial on homeless clients with severe mental illness and SUD reported that participants in both IACT and ACTO modalities had more days in stable housing than those in the SC group, but there were no significant between‐group differences between the ACTO and IACT modalities (Fletcher et al., 2008).

###### Outcome 2: Mental health

Seven RCTs and a follow‐up study reported on mental‐health outcomes in different ACT interventions (Essock et al., 1998, 2006; Fletcher et al., 2008; Lehman et al., 1997; Morse et al., 1992, 1997, 2006). A three‐arm trial with IACT with a substance‐use specialist, ACTO and standard care reported reductions in the mean scores of psychiatric symptoms at 3, 15 and 30 months using BPRS scale. While psychiatric symptoms were reduced in the ACTO/ACTI versus the SC comparisons, it was not statistically significant (*p* > .1) (Fletcher et al., 2008). In another 24‐month trial, participants receiving any of the interventions reported improvements over time in mental health at 6 months—2.01 (SD0.44), 1.94 (SD0.42), and 1.98 (SD0.58) as well as at 24 months—1.88(SD0.54), 1.66 (SD0.46) and 1.86 (SD0.60) for ACTO, IACT and the control groups, respectively (Morse et al., 2006). A follow‐up trial found no between‐group differences in the group modalities and the treatment condition had no effect on psychiatric symptoms (*p* = .19) (Morse et al., 2008). A post hoc analysis of a previous three‐arm treatment of homeless mentally ill individuals with ACT, ACT with community workers and BCM showed that the participants in both ACT conditions reported fewer psychiatric symptoms in the areas of thought disorder and unusual activity, as measured on the Brief Psychiatric Rating Scale (BPRS), than those receiving broker case‐management services (Morse et al., 1997). However, no significant between‐group differences were found on anxiety‐depression, hostility‐suspicion, or self‐esteem scales (Morse et al., 1997). The mental‐health outcomes did not differ in an updated trial (Kenny et al., 2004). In contrast, in a 12‐month ACT intervention for homeless persons with mental illness, there were significant reductions (*p* < .001) in psychiatric symptoms using the Colorado Symptom Index (CSI) at 3 months (mean 4.10 (SEM 0.10) versus 3.61 (SEM 0.10), 6 months (mean 4.10 (SEM0.10) versus 3.61 (SEM 0.10) and 12 months (Mean 4.12 (SEM0.11) versus 3.77 (SEM0.11) in the ACT group, respectively, compared to the TAU group (*p* = .03) (Lehman et al., 1997). Another 18‐month ACT intervention versus SCM for homeless clients who were high service users with serious mental disorders reported significant improvement (lower symptom levels) in psychoticism subscales with time (*p* < .01) in the ACT groups than the SCM groups (Essock et al., 1998). Finally, a 3‐year ACT intervention (*n* = 99) with SCM (*n* = 99) reported no significant between‐group differences favouring the ACT group over the SCM in reductions in the severity of psychiatric symptoms, using the Expanded Brief Psychiatric Rating Scale (Essock et al., 2006).

###### Outcome 3: Quality of life

Three trials reported on the impact of ACT intervention on quality of life (Essock et al., 1998; Essock et al., 2006; Lehman et al., 1997). Lehman (1997) ACT trial (*n* = 77) reported improvements in the quality of life subscales at 6 and 12 months, respectively. The participants in the ACT programme compared to the TAU group were more satisfied with their general well‐being (ACT mean 4.70, SEM 0.16 vs. comparison mean 4.17, SEM 0.16, *p* = .02), neighbourhoods (ACT—mean 4.89, SEM 0.16 vs control mean 4.13, SEM 0.16, *p* = .001), and health (ACT mean 4.98, SEM 0.12 vs. comparison mean 4.50, SEM 0.12, *p* = .006). However, there were no between‐group differences in participants' objective quality of life or in most life‐satisfaction subscales (except for general well‐being and neighbourhood) (Lehman et al., 1997). Another ACT trial reported significant improvements in general life satisfaction (from 4.34 to 5.07) in participants in the ACT group compared to the SCM group (from 4.62 to 4.61) (*p* < .05) (Essock et al., 1998). A follow‐up trial reported no statistically significant between‐group differences observed in the participants' quality of life (Essock et al., 2006).

###### Outcome 4: Substance use

Five RCTs and a follow‐up study on different ACT interventions reported findings on substance‐use outcomes (Essock et al., 2006; Fletcher et al., 2008; Morse et al., 1992, 1997, 2006). In three RCTs, no major reductions were reported in substance‐use ratings, days of substance/alcohol use or alcohol consumption at 6‐ to 24‐month periods (Morse et al., 2006, 2008; Fletcher et al., 2008). One 12‐month three‐arm trial of ACT, a drop‐in centre and outpatient treatment reported more ounces of alcohol consumption—2.83 ounces (SD 9.11) in the ACT group compared to 0.50 (SD 1.08) and 0.95 (SD 1.98) ounces in the drop‐in centre group and the outpatient clinic group, respectively. There was no significant improvement in alcohol consumption over time (*p* = .781) (Morse et al., 1992). Another three‐arm trial reported no significant positive effect between the ACT (O) and the Broker CM interventions on the self‐esteem score, the client‐ or interviewer‐rated need for alcohol or drug treatment, or days of substance abuse at 18 months, using the addiction severity index (Morse et al., 1997). Another three‐arm trial found some improvements in substance use, with ratings assessed at 6, 12, 18 and 24 months. The mean substance‐use ratings for the ACTO and IACT participants were 2.70 (SD 1.28) and 2.76 (SD 1.11) compared to a score of 2.62 (1.15) in the control group, at 24 months (Morse et al., 2006). Even though participants receiving interventions reported improvements over time in substance‐use outcomes, no significant difference between treatment groups (*p* = .72) was observed (Morse et al., 2006). A follow‐up study compared previously published substance‐use ratings data for ACTO, IACT and standard care with new integrated assertive community treatment (NIACT) found that clients in the NIACT condition had reduced their frequency of drug use more than clients in the IACT and ACTO conditions (Morse et al., 2008). One trial found significant differences in the alcohol‐, drug‐ and substance‐use and substance‐abuse scales (*p* < .01, *p* < .0, *p* < .05 and *p* < .05) between ACT and SCM over a 3‐year period (Essock et al., 1998). Another three‐arm ACT intervention reported that the mean substance‐use rating was 2.58 (SD 1.11) and 2.73 (SD 1.25) for the ACTO and IACT groups, whereas the control group had a rating of 2.44 (1.20) (Fletcher et al., 2008). All groups improved over time; however, there were no significant differences between treatment groups. An ACT trial in two urban sites reported a score of 3 or higher for the AUS at baseline, and clients with alcohol‐use disorder showed no difference over time according to the researcher's average Alcohol Use Scale (AUS) ratings but they differed significantly over time on their self‐reported days of drinking (Essock et al., 2006). Using the drug‐use scales, with a baseline score of 3 or higher, a greater between‐group difference over time favouring ACT was observed at site 2 but not at site 1 (Essock et al., 2006). However, the groups did not differ in self‐reported days of drug use but their reported days of use declined by about one‐third overall (Essock et al., 2006). However, in site 1, the treatment groups showed steady and similar improvement over time when assessed with the Substance Abuse Treatment Scale (SATS), whereas in site 2, the ACT group showed more rapid improvement than the SCM group but there were no significant differences between groups (Essock et al., 2006).

###### Outcome 5: Hospitalisation

Four ACT trials reported on hospitalisation outcomes (Clarke et al., 2000; Essock et al., 1998, 2006; Lehman et al., 1997). One 24‐month RCT trial reported that slightly more ACT clients (46%) were hospitalised compared to 40% of those in usual care (OR, 1.22; 95% CI, 0.62–2.4) at 24 months, whereas 35% of the ACT clients (*n* = 40/114) visited the ER compared to 31% in the usual care group (*n* = 15/49) (OR, 1.23; 95% CI, 0.6–2.51) in the same period (Clarke et al., 2000). However, there were no significant between‐group differences in the number of hospitalisations or ER visits. In contrast, a trial reported marginal differences in hospital utilisation (*p* = .078) between ACT versus SCM clients after 18 months; however, the number of days hospitalised between 6 and 12 months was significantly higher for SCM clients (mean—25.8 days (SD = 57.1)) versus 17.2 days (SD = 46.9) (*p* < .05) for the ACT clients (Essock et al., 1998). Also, the ACT clients were not discharged from hospital more quickly (51 discharged clients) than those assigned to SCM, with 46 discharged within 18 months (Essock et al., 1998). A similar trial reported a slightly lower number of days hospitalised in the ACT group (mean 32, SD 91) than the SCM group (mean 41, SD 60) but this was not significant (Essock et al., 2006). Finally, one trial reported a significantly lower mean number of emergency department visits over 12 months (*p* = .005) in the ACT groups (mean 0.8 visits, SEM0.3) compared to UC (mean 2.0 visits, SEM0.3) (Lehman et al., 1997).

###### Outcome 6: Employment

No RCTs were reported on the effects of ACT on employment.

###### Outcome 7: Income

Only two trials reported evidence on the effects of different ACT interventions on participants' incomes (Morse et al., 1992, 1997). One three‐arm trial of ACT interventions—continuous community‐based services with a no‐reject policy, a drop‐in centre, and usual care (outpatient treatment) found no significant between‐group differences on participants' income outcomes (Morse et al., 1992). Also, another 18‐month trial comparing ACT groups with broker case‐management groups reported no significant between‐group differences associated with their income outcomes (Morse et al., 1997).

#### 5.3.6 ICM Interventions

Thirteen RCTs and one nonrandomised trial that compared intensive case‐management intervention with the usual care or service, SCM and other services were included in this study.

##### Outcome 1: Housing stability

Thirteen trials and one nonrandomised trial on different ICM interventions reported evidence on housing outcomes (Braucht, 1995; Burnam, 1995; Conrad et al., 1998; Grace & Gill, 2014; Felton et al., 1995; Korr & Joseph, 1996; Malte et al., 2017; Marshall et al., 1995; Orwin et al., 1994; Toro et al., 1997; Rosenblum et al., 2002; Shern et al., 2000; Shumway et al., 2008; Stahler et al., 1995). One 24‐month trial of ICM interventions (*n* = 289) focused on long‐term, open‐ended, outreach‐oriented service that focused primarily on system advocacy and linkage activities in homeless and chronic inebriate clients reported significantly reduced mean number of days homeless in the last 60 days at 6, 12 and 18 months (*p* = .0013, *p* = .0007 and *p* = .0075) respectively, for the ICM participants (Cox et al., 1998). Also, there was a notable short‐term increase in the mean number of days in stable housing (*p*= .0072) in the ICM clients (Cox et al., 1998). Another RCT on a Demonstration Employment Project—Training and Housing (DEPTH) for mentally ill homeless adults with children reported reductions in the mean number of days homeless at 12 and 18 months in the ICM groups, but this was not significant (Toro et al., 1997). However, a significantly higher proportion of ICM participants (75.0%) were housed at 6 months compared to 34.1% of those in the UC group (OR 6.40 [2.61–15.68; *p* < .0001) (Toro et al., 1997). Another intensive client‐centred case‐management trial (*n* = 235) in unemployed and homeless youth reported a higher mean number of residence moves at 12 months in the ICM groups [2.2 more moves (SD 1.9) than the UC groups [1.8 moves (SD 1.9)], an effect that was reversed at 24 months (Grace & Gill, 2014). Also, the trial observed a lower mean number of days in no‐rent/privately rented accommodations observed at 12 and 24 months for the ICM intervention group but this was not statistically significant. Furthermore, a pooled analysis of three trials on ICM versus TAU reported some reductions in the number of days individuals spent homeless in the short term; that is, 7–12 months (SMD, −0.22; 95% CI, −0.49–0.05), but participants in the ICM programme showed significant reductions in the number of days homeless (SMD, −0.22; 95% CI, −0.40 to −0.03) after 13 months (Cox et al., 1998, Grace & Gill, 2014, Toro et al., 1997). The total overall effect: *Z* = 2.33 at *p* = .02. The interventions favour the use of ICM over TAU. Also, a 4‐months quasi‐experimental ICM trial on homeless substance users (*n* = 250) reported that improvements in the number of days homeless in the last 30 days was 13.2 versus 10.2 days for the ICM and TAU groups, respectively, but no significant between‐group difference was found even at 80% follow‐up (Rosenblum et al., 2002). Another 6‐month ICM trial on homeless mentally ill adults aged 18 years or older found that over twice as many (*n* = 36, 75%) ICM participants were housed than in the control group (*n* = 15, 34.1%) (Korr & Joseph, 1996). Clients receiving ICM were 5.8 times more likely to be housed (OR = 5.8; 95% CI, 2.35–14.31) than the control at follow‐up (OR = 6.40 at *p *< .0001) (Korr & Joseph, 1996). In a 14‐months ICM trial with homeless individuals with severe psychiatric disorders, for the ICM participants, there were no significant differences between groups in days in better or worse accommodations. The ICM groups had more days in better accommodation (mean: 44.3 days) than in the control group (mean 32.3 days) and fewer days in worse accommodation than the control (mean 15.1 vs. 33.4 days) (Marshall et al., 1995). Still, a three‐arm ICM trial with ICM for homeless persons with alcohol‐ or drug‐use problems reported that fewer ICM clients 58 (0.34) achieved housing independence than the control group 100 (0.48) clients; also, an effect size of −0.28 favoured the control condition (Orwin et al., 1994). A 24‐month ICM programme for street‐dwelling individuals with psychiatric disabilities reported that participants spent significantly more time in community housing (MD 11.07, *t* = 2.28 at *p* = .023) and shelters (MD 20.29, *t* = 5.48 at *p *< .0001) and significantly less time on the streets (MD −26.71, *t *= −4.25 at *p* < .0001) (Shern et al., 2000). However, there was no significant difference between time spent in institutions in both the ICM and TAU groups (MD −2.33) (Shern et al., 2000). Amongst predominantly male and unmarried homeless veterans enroled in addiction treatment and exposed to ICM interventions, there were no statistically significant differences in housing stability in both the intervention and control groups (Malte et al., 2017). Another 6‐month three‐arm ICM trial for adult males experiencing homelessness with alcohol and/or drug problems and stable mental health reported some improvements in housing stability, but there were no statistically significant between‐group differences (Stahler et al., 1995). Also, an earlier three‐arm trial for homeless adults with both serious mental illness and substance dependence reported no significant difference in the percentage of time spent on the streets between the residential and nonresidential groups or between both treatment groups and the control over a 9‐month follow up (Burnam 1995); and another 10‐month trial reported no significant difference between ICM and TAU on housing stability (Braucht 1995). Finally, a 24‐month ICM intervention on the homeless or vulnerably housed who were frequent emergency department users with psychological problems reported significantly lower levels of homelessness in patients who were randomised to the case‐management condition (Shumway et al., 2008).

##### Outcome 2: Mental health

Twelve studies on ICM interventions reported evidence on the mental‐health outcomes (Braucht, 1995; Burnam, 1995; Clark & Rich, 2003; Cauce, 1994; Felton et al., 1995; Malte et al., 2017; Marshall et al., 1995; Orwin et al., 1994; Shern et al., 2000; Shumway et al., 2008; Stahler et al., 1995; Toro et al., 1997). A three‐arm ICM trial on homeless individuals, long‐term psychiatric inpatients, and heavy users of emergency services with serious and persistent mental illness reported no significant differences between groups (Felton et al., 1995). Another three‐arm ICM trial on homeless persons reported lower improvements in 30‐day psychiatric symptoms (0.03) compared to 0.15 in improvements in the control group measured according to the Addiction Severity Index (ASI) (Orwin et al., 1994). In contrast, the improvement rate in the psychiatric composite score was 0.15 for the ICM group and 0.21 for the control group; however, there was no statistically significant difference between these groups (Orwin et al., 1994). Another 3‐month ICM trial with homeless adolescents between 13 and 21 years old reported slight reductions in youth self‐reports of depression problems according to the Reynolds Adolescent Depression Scale (RADS) (MD: −3.70; *t* = −1.33 at *p* = .18); and in total behavioural problems (MD: −0.50; *t *= −0.27 at *p* = .78) in the ICM group; however, no improvement was found in the antisocial behaviour (MD: 0.20; *t* = 1.26 at *p* = .20) as measured using the Problem Behaviour Scale (PBS) (Cauce 1994).

Also, a randomised ICM trial for homelessness with alcohol and/or drug problems and stable mental health reported effects on the number of days experiencing psychological symptoms in the last 30 days, using the ASI, found slight improvements in mental health (Stahler et al., 1995). There were no notable changes in participants' self‐esteem or mastery scores or these youth's self‐reports on depression, antisocial behaviour, their Problem Behaviour Scale (PBS), or the group's total behavioural problems (Stahler et al., 1995).

In one 14 month trial, ICM clients who were compared to TAU clients were assessed for the frequency of items of embarrassing or disruptive behaviours, using the REHAB standardised behaviour scale showed better outcomes on three of the five variables (REHAB general and deviant behaviours and mental state) but only deviant behaviour differed significantly between the two groups (MD, 0.3; 95% CI, 0.15–0.46) (Marshall et al., 1995). There was no significant difference in the severity of psychiatric symptoms. However, there was a significant reduction (mean = 0.79; 95% CI, 0.26–1.32; *p *= .0038) in psychological symptoms (deviant behaviours) from the baseline between the ICM and UC groups, using a standardised behaviour‐rating scale (REHAB) (Marshall et al., 1995). Also, another 18‐month ICM trial with mentally ill homeless adults with children reported psychological symptoms measured at 6, 12 and 18 months (Toro et al., 1997). When using the SCL‐90‐R checklist, significant reductions in psychiatric symptoms (MD −0.19; *p* = .04) were found between the ICM and UC groups, and the mean MLEI score on stressful life events was MD: −2.10 (*p* = .04) at 18 months (Toro et al., 1997). There were no significant differences in the psychological symptoms, as measured with the SCL‐90‐R, across time or groups (Toro et al., 1997). A 24‐month ICM ‐based intervention (“Choices”) compared to TAU reported significantly greater reductions in anxiety, depression, and thought disturbances in the experimental group (*t *= 2.41, *p* < .001), as measured with Colorado Symptom Index (CSI), compared to the control group (Shern et al., 2000). In a quasi‐experimental trial of a comprehensive housing programme compared with case management for homeless adults with mental illness (*n* = 152) Clark & Rich, 2003 found no significant differences between treatment groups for low and medium impairments. Also, patients randomised to the case‐management condition in the ICM intervention for homeless or vulnerably housed frequent emergency department users (*n* = 252) found no statistically significant differences for psychiatric symptoms (Shumway et al., 2008). Another 12‐month ICM intervention (*n* = 91) versus a drop‐in housing support group (*n* = 90) for homeless veterans enroled in addictions treatment reported greater improvements in the ASI alcohol composite score in the ICM group compared to those in the control group, but this was not statistically significant (Malte et al., 2017). Also, there were no significant differences between the two groups in mental‐health outcomes as measured by the ASI Psychiatric or the SF‐36 MCS at 12 months follow up.

##### Outcome 3: Quality of life

Five included studies assessed the effects of different ICM interventions on quality of life outcomes (Braucht 1995; Cauce 1994; Felton et al., 1995; Marshall et al., 1995; Shern et al., 2000). A 3‐month ICM intervention study on homeless adolescents 13–21 years old reported = slight improvements in quality of life, using the Life Domains Scale (LDS): a mean score of 3.6 (SD 0.8) compared to the control group 3.5 (SD 0.7). The study also reported self‐esteem, using the Rosenberg Self‐Esteem Scale (RSES): mean score 1.7 (SD 1.7) compared to the control group 1.6 (SD 1.6) (Cauce 1994). Another 14‐month RCT on ICM interventions for homeless persons reported no improvements in quality‐of‐life outcomes (MD, 0.0; 95% CI, −0.42–0.42) (Marshall et al., 1995). Also, a three‐arm ICM intervention on homeless individuals found significant linear group‐by‐time interactions for satisfaction with living situations and finances as well as fewer life problems, as measured by sub scores (Felton et al., 1995). In contrast, a 10‐month trial assessing the effects of ICM reported better improvements in general life satisfaction in the control group than in the ICM group (Braucht 1995). Finally, a 24‐month ICM (“Choices”) versus TAU trial reported between‐group differences in life satisfaction across 7 life areas (Shern et al., 2000). Individuals in the experimental condition reported consistently greater improvements in life satisfaction than their peers in the control group, in 6 of the 7 life areas: overall life satisfaction (*p* = .001); leisure (*p* = .027); financial (*p* = .001); safety (*p* = .005); health (*p* = .006); family (*p* = .005); and social (*p* = .56) (Shern et al., 2000). In most cases, experimental groups showed substantial gains, which were often 0.5 SDs more than observed in the control group (Shern et al., 2000).

##### Outcome 4: Substance use

Ten ICM trials reported evidence on substance‐use outcomes (Cauce 1994; Cox et al., 1998; Braucht 1995; Burnam 1995; Felton et al., 1995; Malte et al., 2017; Orwin et al., 1994; Rosenblum et al., 2002; Shumway et al., 2008; Stahler et al., 1995; Toro et al., 1997). A 24‐month three‐arm ICM intervention reported a lower improvement rate of 0.19 ((P<0.05) in alcohol use over 30 days, as assessed by the ASI scale for ICM participants compared to 0.40 rate condition (Orwin et al., 1994). Also, the mean number of drug‐use days was higher in the ICM groups (3.3 days) than in the TAU groups (1.51 days). However, there were no significant differences in the mean alcohol use in the past 30 days and the composite alcohol‐ or drug‐use scores (Orwin et al., 1994). Another ICM trial (*n* = 289) on the substance use outcomes of homeless chronic inebriate clients reported lower numbers of days of alcohol use in the past 30 days at 18 months in the ICM interventions compared to the UC group (MD −4.00, *t* = −2.31 at *p* = .02) (Cox et al., 1998). Also, the number of days of alcohol use in the past 30 days to the point of feeling the effects was lower in the ICM groups at 6 months (MD: −3.40; *p* = .05) (Cox et al., 1998). The number of days of alcohol use since the last interview were significantly lower at 6, 12, 18 months in the ICM groups ‐: MD: −21.00, −19.00 and −29.00 at *p* = .02, *p* = .04 and *p* = .001, respectively. While the number of detoxification centre admissions was reduced as the interventions moved from short (6 or 12 months) to long term (18 months); however, this was not statistically significant (Cox et al., 1998). Furthermore, ratings on how troubled or bothered subjects were by alcohol were lower in the ICM groups compared to the UC groups at 18 months (*t *= −2.29 at *p* = .02) than at 6‐ or 12‐months as well, the composite score for alcohol use on the Addiction Severity Index (ASI) was significantly lower in the ICM group compared to the control at 6 months (*t *= −1.94; *p* = .05), but not at 12 or 18 months (Cox et al., 1998). Another 18‐month ICM intervention (*n* = 202) reported no significant differences between groups across time in the number of drinks consumed daily over the course of the past year, using an author‐developed alcohol‐drinking index (Toro et al., 1997). However, a 4‐month study reported substantial reductions between ICM and UC groups in crack use in the past 30 days: 4.1 vs. 2.2 days (MD: −1.9; *p* < .05) (Rosenblum et al., 2002). A 9‐months trial of a shelter‐based intensive case‐management programme staffed primarily by peer counsellors versus regular shelter services provided by city‐staffed case managers found no significant differences in substance use, assessed using the Addiction Severity Index (ASI) (Stahler et al., 1995). However, in another trial, recent alcohol and cocaine use across the groups from the baseline significantly improved (p<0.05) at 18 months (Toro et al., 1997). A 3‐month ICM trial for youth measured substance use with a Personal Experience Screening Questionnaire (PESQ) and reported mean scores of 25.4 (SD 8.9) and 27.0 (SD 9.7) for the ICM and control groups, respectively, but no significant differences between groups (Cauce 1994). Also, a 10‐month ICM intervention reported little to no significant effects on substance use between ICM and TAU (Braucht 1995), while another three‐arm ICM trial did not observe significant differences in substance use in the past 30 days (days using alcohol, level‐alcohol use, days using drugs or severity of drug use) between all groups(Burnam 1995). Also, patients in the ICM condition showed reduced problematic substance use compared to the control group after 24 months (Shumway et al., 2008), and ICM participants (*n* = 91), in contrast to a drop‐in housing support group (*n* = 90), showed greater improvements in the ASI alcohol composite score. However, there was no significant between‐group differences in the proportion of participants who had abstained from alcohol or drug use (Malte et al., 2017).

##### Outcome 5: Hospitalisation

Five trials reported evidence on hospitalisation outcomes (Korr & Joseph, 1996; Malte et al., 2017; Marshall et al., 1995; Rosenblum et al., 2002; Shumway et al., 2008). A 14‐month ICM trial for homeless individuals with severe and persistent psychiatric disorders reported slightly fewer days in hospital (mean14.6; SD 30.5) for the intervention group than in the control group (mean 21.8; SD 62.3) (Marshall et al., 1995). Also, another 4‐month ICM trial reported significantly fewer hospital emergency‐room visits in the ICM group compared to the control group (26% vs. 45%; *p* < .05) (Rosenblum et al., 2002). In addition, a 24‐month ICM trial consisting of homeless or vulnerably housed who were frequent emergency‐department users reported significantly lower levels of hospital emergency‐rooms visits in ICM participants compared to the control group (Shumway et al., 2008). However, a 12‐month trial reported no significant between‐group differences in the number of days in hospital after adjusting for hospital days during the baseline period (Malte et al., 2017), and another 6‐month ICM intervention for homeless mentally ill adults aged 18 or older found no statistically significant difference between groups on the number of days in hospital (Korr & Joseph, 1996).

##### Outcome 6: Employment

A 10‐month (ICM) trial (*n* = 178) that consisted of homeless individuals (18 years or older) with alcohol or other substance‐abuse problems found nonsignificant effects on employment outcomes (Braucht 1995). Also, a 2‐year ICM trial with homeless chronic inebriate clients (*n* = 150) reported no significant between‐group differences in employment outcomes compared to the standard‐care group (Cox et al., 1998). Although a 14‐month ICM trial on homeless individuals with severe and persistent psychiatric disorders reported that the ICM participants spent more days in any employment than those in the control group, the effect was not significant (Marshall et al., 1995). Another 24‐month three‐arm ICM trial found significantly better improvements in employment outcomes compared to the usual or episodic care of those in the intervention group; however, the report highlighted the data‐analysis procedures as not accurately described (Orwin et al., 1994). Finally, a 6‐month three‐arm ICM trial that consisted of adult males experiencing homelessness with alcohol and/or drug problems and who had stable mental health reported slight improvements in employment outcomes, but the between‐group differences were not statistically significant (Stahler et al., 1995).

##### Outcome 7: Income

Five trials reported on evidence of ICM interventions on participants' income (Cox et al., 1998; Grace & Gill, 2014; Rosenblum et al., 2002; Shumway et al., 2008; Toro et al., 1997). A 24‐month ICM intervention that included monthly public income assistance for homeless chronic inebriate clients (*n* = 150) found a significant group effect favouring the ICM group over those in the standard‐care group (*n* = 148) (Cox et al., 1998). A 24‐month nonRCT of intensive client‐centred case management that provided a range of brokered services through a single point of contact (YP case manager) to unemployed and homeless young people (*n* = 422) found no statistically significant between‐group differences in higher receipts of public income assistance in the ICM group than in the control group (Rosenblum et al., 2002). Another 24‐month trial reported that the patient population of homeless or vulnerably housed, frequent emergency‐department users with psychological problems and randomised to the case‐management condition showed significantly lower levels of unmet financial needs than those in the usual care (Shumway et al., 2008). Finally, an 18‐month ICM trial that consisted of mentally ill homeless adults with children (*n* = 202) found no statistically significant differences in effects on income outcomes between groups (Toro et al., 1997).

#### 5.3.7 Critical time intervention

Our systematic review yielded 5 studies (11 citations) on CTI (De Vet, 2017; Herman et al., 2011; Lako et al., 2018; Susser et al., 1997; Shinn et al., 2015). One study (2 citations) specifically explored family critical time intervention (FCTI), a multidisciplinary community‐based service model targeted towards families, that connects families with social services and forms supportive relationships with families and friends during critical times of transition from shelters to community housing (Shinn et al., 2015).

In all studies, CTI consisted of three phases. This was primarily broken down into (1) transition to the community, (2) tryout, and (3) transfer of care. One study (Susser et al., 1997) described these phases as (1) accommodation, (2) try out, and (3) termination. All trials compared the intervention to the TAU, though the scope of the interventions associated with usual care somewhat differed between trials. Between the five studies, follow up ranged from 9 to 24 months. All studies were RCTs; two were based in the Netherlands (de Vet 2017; Lako et al., 2018) and three were from the United States (Herman et al., 2011; Susser et al., 1997; Shinn et al., 2015).

##### Outcome 1: Housing stability

Three out of four studies found that CTI significantly reduced the number of days spent homeless (Herman et al., 2011; Susser et al., 1997; Shinn et al., 2015). Two studies reported on the total number of homeless nights experienced in an 18 month follow‐up period (Herman et al., 2011; Susser et al., 1997). In both studies, the participants in the CTI group received 9 months of CTI plus usual services and then usual services only for the following 9 months, while the participants in the TAU group received a total of 18 months of usual services only (Herman et al., 2011; Susser et al., 1997). In both studies, usual care was a mix of various types of case management and clinical treatment. Within Herman et al. (2011), all participants in both treatment arms also received basic discharge‐planning services and access to psychiatric treatment while living in the transitional residence. Herman et al., 2011 found that over an 18‐month period in persons with severe mental illness discharged from New York City inpatient psychiatric‐treatment facilities (*n*= 150), there were 1812 total homeless nights in the CTI group compared to 2,403 homeless nights in the control group (*p* < .001). Controlling for baseline homelessness, the OR for the CTI group was 0.28 (95% CI, 0.78 to −1.02), suggesting that the CTI group had a 5‐fold reduction in risk of homelessness within the intent‐to‐treat analysis. Similarly, Susser et al. (1997) examined 96 men with severe mental illness who had been discharged from an NYC men's shelter's on‐site psychiatric programme to return to community housing. The number of homeless nights over the 18‐month follow up period was also statistically significant, with 1415 homeless nights in the CTI group and 4,370 in the TAU group. The risk of a major homeless episode was significantly lower in the CTI than in the usual‐services group (*p* = .003) and the survival curves comparing the differences between major homeless episodes between both groups widened over the duration of the study.

Susser et al. (1997) subsequently divided homelessness into extended homelessness (more than 54 nights), intermediate homelessness (30–54 nights), and transient homelessness (1–29 nights). There was a significantly reduced amount of extended homelessness in the CTI group (*X* ^2^ = 4.0, df = 1, *p* = .045), with a relative risk of 0.53 (95% CI Taylor series, 0.27–1.01). There was no significant difference between episodes of intermediate homelessness and transient homelessness.

In examining time spent in conventional housing, Shinn et al. (2015) studied 200 newly homeless families in which mothers had diagnosable mental illness or substance‐use problems. They found that, after random assignment, families in the FCTI treatment group spent 43% of the first 3 months and 91% of the next 6 months in conventional housing in the community, compared to 8 and 45% for families in the usual‐care control group. The FCTI services ended at 9 months. Families in the FCTI group spent 89% of the time from 9 to 15 months and 86% of the time from 15 to 24 months in community housing, compared to 76 and 73%, respectively, in the control group.

FCTI may also reduce transition time from a shelter to community housing. A longitudinal RCT (*n* = 210) examined homeless mothers with a mental illness/substance‐abuse disorder as they moved from homeless shelters into affordable housing (Samuels et al., 2015). Families in the FCTI intervention group were not required to meet specific housing‐readiness criteria, compared to the control group. Hence, a higher number of FCTI families left the shelter (98%) compared to the control group (84%) and the average number of days until they moved into stable housing was significantly reduced, at 91.25 (SD 82.3) days compared to the control group average of 199.15 days (SD 124.4). Over the course of the study, the control group became significantly less likely to spend a longer period in the shelter than the intervention group. Because of the early success of the FCTI in rehousing families in a timely manner, county officials increased the availability of permanent‐housing vouchers to families involved in the homeless service system, enabling families in the control condition to also receive housing more quickly.

One trial, based out of the Netherlands, did not find a significant difference in the number of days rehoused with CTI versus TAU (de Vet, 2017). The number of days re‐housed was defined as living in conventional independent housing; i.e., property or legal (sub) tenancy, or accommodation permanently provided by relatives, friends or acquaintances. The Residential Follow‐Back Calendar was used to assess participants' residential history in this multicentre parallel‐group RCT.

##### Outcome 2: Mental health

In two studies, there were no significant differences in levels of psychological distress between CTI versus TAU, as measured using the Global Severity Index, which provides an average score of a 53‐item symptom inventory (de Vet, 2017; Lako et al., 2018). However, a longitudinal RCT by Samuels et al. (2015) (*n* = 210) examined the effect of FCTI on homeless mothers with mental illness and found that both the FCTI and the usual services resulted in improvements in these mothers' mental‐health problems over time, even if there were no significant differences between the intervention and control groups. The Global Severity Index was also used to assess mental health in this study, with data from nine primary‐symptom sub‐scales of the Brief Symptom Inventory: psychopathology, including somatisation, obsessive‐compulsive, interpersonal sensitivity, depression, anxiety, hostility; phobic anxiety; paranoid ideation; and psychoticism. At baseline, the mothers in the study had relatively high levels of mental‐health symptoms, with the mean standard score for the GSI falling close to the borderline problem range (*M* = 57.7; SD = 12). Between the baseline and the 15‐month assessment, the GSI scores dropped by an average of 9 points, bringing most mothers into the normal range of mental health relative to the general adult population. The mean score for those in the top 25th percentile at baseline was *t* = 66, dropping to *t* = 59 at 15 months. The mean score for mothers in the lowest 25th percentile dropped from *t* = 50 to *t* = 33. Both the control and intervention groups experienced similar improvements in symptoms over time, with the elevated symptoms in mothers in the treatment group declining from 77% at the baseline to 42% at the 9‐month follow up, and the percentages in the control condition declining from 73% to 38%, respectively.

In one of these studies—a multicentre RCT in the Netherlands that examined 183 adults who were moving from shelters to supported or independent housing— the CTI had an added differential effect on psychological distress for participants who were experiencing less social support, with an estimated difference in the intervention effect = 0.19 (*p* = .013, 95% CI, 0.004–0.34) suggesting that a CTI may provide more benefit to less supported individuals (de Vet, 2017).

Two studies measured self‐esteem, using the 10‐item Rosenberg Self Esteem scale, and found no difference with the CTI intervention (de Vet, 2017; Lako et al., 2018). In one study, there was also no impact on depression as measured with the 20‐item Centre for Epidemiological Studies Depression Scale (Lako et al., 2018). However, in the same study, CTI appeared to significantly improve symptoms of PTSD during follow up (adjusted MD, 7.27; 95% CI, −14.31 to −0.22, *p *= .04). The PTSD symptoms were measured by the sum score of the 15‐item Impact of Event scale (Lako et al., 2018 **)**. Further analysis revealed that the significant effect was primarily on non‐Dutch speaking women, compared to Dutch‐speaking women. No other studies examined the effect of CTI on PTSD.

CTI may also have a benefit for improving negative symptoms of schizophrenia. One RCT assessed 96 men with schizophrenia and other psychotic disorders who had been discharged from a homeless shelter (Herman et al., 2011). Symptom severity at the baseline and at 6 months was assessed using the Positive and Negative Symptom Scale (PANN), a 30‐item, 7‐point severity scale that is used to examine the three domains of positive symptoms, negative symptoms and general psychopathology. Data was collected from the 76 subjects for which there was a complete symptom set. The CTI was associated with significantly fewer negative symptoms, at the 6‐month follow up, defined as a cumulation of ratings of blunted affect, emotional withdrawal, poor rapport, passive‐apathetic social withdrawal, difficulty in abstract thinking, lack of spontaneity, and flow of conversation and stereotyped thinking. The mean change in the CTI group was −2.6, compared to a mean change of +1.0 in the usual treatment group. In the regression analysis that controlled for baseline score, in the 6‐month outcomes, there was a significant group effect for negative symptoms only (*F* = 6.7, *p* = .02). There was no significant effect on positive or general psychopathology symptoms.

Regarding the effect of FCTI on children, one RCT compared FCTI to usual care for children of 200 newly homeless families with maternal diagnosable mental illness or SUDs (Shinn et al., 2015). Mothers completed the Child behaviour Checklist, and youth ages 11–16 reported their own internalising and externalising behaviours using the Youth Self‐Report. Teachers reported externalising behaviours using the Teacher Report Form. The FCTI appeared to reduce internalising behaviours in children until 24 months when both groups had similar levels. The greatest difference between the 2 groups (0.6 SDs) was at 9 months. The FCTI also had an impact on externalising behaviours, with an improvement in group differences in T scores by 6.2 (0.5 SDs). There was also an improvement in 0.4 SDs in internalising and 0.2 in externalising for children aged 1.5–5 years. For children between 6 and 10 years, the intervention on mental health had no effect but both the CTI and usual‐care treatment groups showed significant improvements, over time, for mother‐reported internalising and externalising behaviours and child‐reported depressive symptoms. In adolescents aged 11–16 years, the FCTI group had a reduction in mother‐reported externalising behaviour that was not seen in the usual care group. At 24 months, the difference between these two groups was 6.2 (0.5 SDs). The children's self‐reports showed that there were no effects on externalising behaviours.

##### Outcome 3: Quality of life

Quality of life was assessed in two studies (de Vet, 2017; Lako et al., 2018). CTI was found to have no statistically significant impact on the quality of life in either study. Both studies used general questions on life satisfaction from Lehman's Brief Quality of Life Interview to assess quality of life. Both studies were based out of the Netherlands, were RCTs and had a 9‐month follow‐up period. In one study, there were also no significant differences in re‐abuse between groups, using the question, “[H]ave you been abused since the last interview?” The Impact of Event Scale (IES) at 9 months was lower in the CTI group (M 29.04) compared to care as usual (M 32.19), with a *p* value of .04 (de Vet, 2017).

##### Outcome 4: Substance use

One study examined the effect of CTI vs care as usual on substance use (de Vet, 2017). Excessive alcohol use (i.e., five or more drinks a day) or cannabis use in the past 30 days was assessed using the European Addiction Severity Index. The baseline characteristics between the CTI versus the control group differed, with a higher proportion of participants in the CTI reporting excessive alcohol use in the past 30 days (21%) compared to the control group (20%), with a p value of 0.04. At the 9‐month follow up, there was a trend towards reduced substance use in the CTI group, though it did not reach significance. Twenty‐two percent of participants in the CTI group reported excessive alcohol use compared to 26% in the control group, with an adjusted OR of 0.71, a 95% CI of 0.24–2.09. Cannabis use was reported in 15% of the CTI participants vs 23% in the control group, with an adjusted OR of 0.89 (95% CI, 0.26–3.05).

##### Outcome 5: Hospitalisation

A study by Tomita 2012 found that participants receiving CTI had significantly reduced odds of psychiatric re‐hospitalisations during the final three observation intervals (OR = 0.11; 95% CI, 0.01–0.96). The usual services and CTI groups had a total of 1508 and 1183 psychiatric re‐hospitalisation nights, respectively, during the final three intervals, with both the proportion (27% vs 18%, *p* < .05) and frequency of re‐hospitalisation nights above the median (49 vs. 31, *p* < .05) being significantly higher for the usual‐services group. Psychiatric re‐hospitalisation rates were higher in the usual‐services group during all intervals except one.

##### Outcome 6: Employment

No trials studied the effect of CTI on employment outcomes.

##### Outcome 7: Income

One trial showed that CTI had no significant impact on income‐related outcomes compared to usual services over an 18‐month study period (Susser et al., 1997).

#### 5.3.8 Cost‐effectiveness studies

##### Permanent supportive housing

Fourteen studies performed a cost analysis of PSH interventions and reported mixed results regarding resource requirements. Seven studies showed that the PSH interventions were associated with increased cost to the payers and that the cost of the interventions were only partially offset by savings in medical‐ and social_services costs as a result of the intervention (Aubry et al., 2016; Culhane et al., 2002; Dickey et al., 1997; Gilmer et al., 2009, 2010; Mares & Rosenheck, 2011; Stergiopoulos et al., 2015). Six studies revealed that PSH interventions saved the payers money (Hunter et al., 2017; Larimer, 2009; Lenz‐Rashid, 2017; Chalmers McLaughlin, 2011; Schinka et al., 1998; Srebnik et al., 2013). However, some studies were based on a pre‐post design (Hunter et al., 2017; Lenz‐Rashid, 2017; Chalmers McLaughlin, 2011; Srebnik et al., 2013); therefore, estimated savings cannot be fully attributed to PSH. We identified three economic evaluations: the first study (Holtgrave et al., 2013) was a cost‐utility analysis of PSH which suggested that the provision of housing services was associated with increased costs and increased quality‐adjusted life years (QALY), with an incremental cost‐effectiveness ratio of US$62,493 per QALY. However, the methodological quality of this study was poor and lacked transparency, there were insufficient details on the design and the results of the effectiveness study, and no justification was given for the choice of mathematical model and the key parameters used. Another study concluded that, compared with usual care, PSH was more costly to society (C$7868, 95% CI 4409–11405) but it increased the number of days spent stably housed (140 days, 95% CI, 128–153) (Latimer et al., 2019). If the payer is willing to pay C$56 per 1 day of stable housing, then the PSH is considered cost‐effective.

##### Income assistance

Two studies focused on the cost‐effectiveness of income‐assistance interventions. Rosenheck and colleagues (Rosenheck et al., 2003) considered income‐assistance interventions in the form of rental assistance and showed that intervention clients had greater annual costs but fewer days homeless than the standard‐care and the case‐management‐only groups. For each additional day housed, clients who received income assistance incurred additional costs of US$58 (95% CI, $4–$111) from the perspective of VA, US$50 (95% CI, −$17, $117) from the perspective of the health‐care system, and US$45 (95% CI, −$19, −$108) from the societal perspective. In addition, the benefits gained from temporary financial assistance were found to outweigh the costs, with a net savings of US$20,548 (Evans et al., 2016). We did not identify any studies that reported the cost‐effectiveness of social‐assistance programmes and employment support.

##### SCM and mental health interventions

The economic implications of case‐management interventions (SCM, ICM, ACT, CTI) was highly uncertain. SCM clients were found to incur higher costs than those receiving the usual or standard care (Nyamathi et al., 2016; Shumway et al., 2008) and ACT (Clark et al., 1998; Essock et al., 1998) but lower compared to those receiving a US clinical case‐management programme that included housing vouchers and ICM (Rosenheck et al., 2003). If the cost‐effectiveness studies used a societal perspective and considered the benefits gained by and costs borne to all payers, then SCM did not offer good value for money compared to the ACT for persons with serious mental disorders or those with a concurrent SUD as SCM was more costly but led to more days in unstable housing (Essock et al., 1998) and poorer quality of life (Clark et al., 1998). For ICM, all included studies suggested that the intervention may be cost‐offset or cost‐effective. The Canadian study (Stergiopoulos et al., 2015) showed that the cost of supporting housing with ICM could be partially offset by the reduction in the use of emergency shelters and single‐room occupancy. Consistently, the US study reported that ICM was more likely to be cost‐effective when all of the costs and benefits to society were considered (Rosenheck et al., 2003). A pre‐post study showed that the ICM provided to high users of emergency departments resulted in net hospital cost‐savings of USD $132,726 (Okin et al., 2000). Regarding ACT, included studies that focused on individuals with severe mental illness or dual disorders consistently revealed that ACT interventions were dominant, meaning that they saved the payers money and improved health outcomes (i.e., reduced substance abuse, improved quality of life, resulted in a greater number of community days and a greater number of days in stable housing) than the usual care. (Clarke et al., 2000; Essock et al., 1998; Lehman et al., 1999; Morse et al., 2006; Wolff, 1997). We identified only one cost‐effectiveness study of CTI (Susser et al., 1997), which reported that the CTI was dominant. In other words, despite having comparable costs (US$52,574 vs. US$51,749), the CTI led to greater nonhomeless nights (508 vs. 450 nights) compared to the usual services provided to men with severe mental illness.

No studies reported on the costs, cost‐effectiveness or resource requirements of peer support, SCFs, MAPs, injectable antipsychotics and OAT interventions.

## 6 DISCUSSION

### 6.1 Summary of main results

#### 6.1.1 Permanent supportive housing

The majority of studies found both significant short‐ and long‐term benefits of PSH on our primary outcome of housing stability, as compared to usual care. One study in Toronto, Canada, presented a long‐term analysis, with a median follow up of 5.4 years, and found that the number of days spent stably housed remained significantly higher within the PSH group (Stergiopoulos et al., 2019). Findings from this study suggest that, when provided with housing arrangements that are not contingent on abstinence and coupled with supportive services, individuals who had experienced homelessness were able to maintain lasting housing stability. Most study populations consisted of individuals with severe mental illness who were experiencing chronic homelessness, often with a high degree of comorbid substance use. Subgroup analyses revealed a similar benefit of PSH for both youth and adults over the age of 50, as well as within both high‐ and moderate‐needs populations, suggesting that PSH caters to different demographics and levels of needs among individuals with lived experience of homelessness (Aubry et al., 2016). There may also be a differential effect related to gender and substance use on housing stability, although the results were mixed and require further study. Future research should focus on examining the equity impact of PSH and how patient characteristics affect the magnitude of effect as it relates to housing stability (Welch et al., 2010). The housing‐intervention models studied included both single‐site models with on‐site support and scattered‐site models with portable support. Our review did not set out to determine the most effective housing model. Further research using a realist lens is mandated to explore the elements and characteristics of PSH that truly contribute to lasting housing stability. Nonetheless, our findings suggest that PSH initiatives can be adapted to different contexts and sites and remain effective in improving housing stability, within populations with a high burden of comorbid mental illness and substance use disorders.

Most studies (*n* = 5) found no benefit of PSH on mental health, though two studies found that mental‐health symptoms improved in both the PSH and TAU groups, despite there being no significant difference between groups (Tsemberis et al., 2004; Sadowski et al., 2009) Three studies found some improved mental‐health symptoms in the comparison group, as opposed to the group in supportive housing (Aubry et al., 2016; Cherner et al., 2017; Young et al., 2009). In one of these studies, both interventions similarly improved symptoms of psychoticism, depression, anxiety, obsessive‐compulsive disorder, interpersonal sensitivity and phobic anxiety, but greater improvements in somatisation, paranoid ideation and global mental‐health‐symptom severity were noted in the “Continuous Integrated System of Care (CCISC)” model, an intervention based out of a traditional substance‐abuse treatment agency (Young et al., 2009). Only one study compared hybrid approaches to housing, such as “parallel housing” (resembling supportive housing + ACT) and “integrated housing” (resembling the traditional model); in this study, those in the integrated‐housing model had less severe psychiatric symptoms during the 18 month follow‐up period (McHugo et al., 2004). Lack of benefit of PSH on mental health outcomes likely represents the complexity of mental illness in this population (Harvard Health Publishing, 2014; Winiarski et al., 2020). Improving health outcomes in persons with lived experience of homelessness therefore may require a multipronged approach, combining multiple evidence‐based interventions. In addition, independence and privacy achieved with PSH may affect social networks and reduce interaction (Cherner et al., 2017). Prior research has demonstrated a positive relationship between perceived social support and psychological health (Harandi et al., 2017) and hence the provision of PSH could result in slower improvements in mental health particularly in early stages (Cherner et al., 2017). Future qualitative research is required to further examine whether PSH is inferior to temporary housing arrangements in regards to social isolation and mental health functioning. Moreover, older homeless adults may have significantly higher improvements in mental health with PSH compared to adolescents, based on subgroup analyses, though further research is required in this area (Aubry et al., 2016). It is possible that this effect may be mediated by lower substance‐use rates in older adults compared to adolescents, due to correlations between substance use and poor mental health in prior research (Aubry et al., 2016).

The effects of PSH on quality of life were mixed, with multiple studies finding a benefit of PSH over TAU, while others favouring the comparison group. Within the two studies that favoured the comparison groups over PSH, improvements in quality of life were still demonstrated in both the intervention and comparator groups (Cherner et al., 2017; McHugo et al., 2004). In one of these studies, the authors suggested that greater improvements in nonhousing outcomes in the comparator group compared to the intervention group may be related to the selection of participants (Cherner et al., 2017). The second study examined hybrid models of housing (parallel vs integrated housing), thus, creating limitations in direct comparison (McHugo et al., 2004). PSH may benefit condition‐specific quality of life (for example, within subscales relating to leisure, living and safety and family), though this requires further exploration (Aubry et al., 2016). As well, previous research on the correlation between quality of life and mental health is limited (Hubley et al., 2014). Nonetheless, the homeless samples in all of our included studies have reported a long lasting struggle with chronic mental health conditions which, in turn, could have been the confounding factor behind the attenuated effect of PSH on quality of life. Interestingly, one study noted a strong association of satisfaction with life with personality (Goldfinger et al., 1999). The authors of this study postulated that satisfaction with life in general may be strongly influenced by one's overall personality, with only some variation in response to residential circumstances. Specific social circumstances informing expectations could be incorporated into future research to better understand these results (Goldfinger et al., 1999).

The effects of PSH on substance abuse were also mixed, though most studies found no difference in substance use with PSH compared to the usual care. Only one study found a benefit over the comparison group, though notably, it was a single‐site trial of a larger multicity study that found no significant difference between groups (Stergiopoulos et al., 2015). One study on homeless clients with problematic substance use found a benefit of the TAU group over the HF intervention, with regard to substantial or severe problems with drug use at 24 months (Cherner et al., 2017). However, the authors postulated that improvements in the comparison group over PSH may have been related to the study's participant selection. Individuals who continued to have difficulties with substance use, despite past substance‐use treatments, were prioritised for admission into the programme; these individuals may have required more intensive interventions than could be provided by the provision of PSH with ICM (Cherner et al., 2017).

Several studies found that PSH reduced emergency‐department visits and days spent hospitalised. Interestingly, one study noted that the Housing‐First (HF) intervention appeared most beneficial for individuals who were recruited from psychiatric hospitals, as compared to individuals recruited from the street, who showed overall fairly low and consistent levels of hospitalisations throughout the study (Tsemberis et al., 2004). Sadowski et al. (2009) identified several factors which may have facilitated the success of PSH in reducing hospitalisations in their study, including the creation of a city‐wide comprehensive and coordinated effort between clinicians, social workers, housing and advocacy groups to secure case management and housing for every participant. Further exploration of factors corresponding to the success of intervention may aid in continued success of PSH implementation and lasting effectiveness.

There were a minimal number of studies that examined the effects on income and employment. The At‐Home‐Chez Toi study found that, compared with the control group of moderate‐needs participants, the HF ICM participants actually had lower odds of obtaining employment (Aubry et al., 2016). Over time, the odds of obtaining employment in both the HF‐ICM and ACT groups improved, compared to the control group, but despite this, the rates of employment in the HF group never exceeded those of the control group. For the ACT high‐needs group, the odds of obtaining employment was not statistically significant compared to the control group. Notably, there were several limitations of this study, including removal of a significant number of participants employed at baseline (11 of 61) due to insufficient data, and a high rate of attrition in the control group. Nonetheless, based on the results, the authors hypothesised that HF may have an initial effect of de‐incentivize work in the moderate‐needs participants whose functional levels are higher. With longer follow up, it is possible that employment in the HF group could actually surpass that of the control. It is unclear from the current research whether this difference may be ameliorated with the integration of evidence‐based supported‐employment programmes (Aubry et al., 2016). Future qualitative research should explore this phenomenon to understand reasons for the trivial effect of PSH on employment. In one study, there were no statistically significant differences in income (Aubry et al., 2016).

#### 6.1.2 Income assistance

Our current review provided some evidence on the effectiveness of income‐assistance intervention in association with broad‐gauged social services delivered to the homeless population in the context of mental health, social health, and healthcare utilisation. The effect of income‐assistance interventions appeared most significant on measures of housing stability, specifically in the format of rental subsidies (Rosenheck et al., 2003; Hurlburt et al., 1996; Pankratz et al., 2017; Wolitski 2009). One study that assessed the effects of CWT showed reduced odds of an episode of homeless in the study group (Kashner, 2002). The effect of financial‐empowerment education (Ferguson, 2018) and IPS programmes (Ferguson, 2018) on housing stability were assessed in limited quantities of studies that did not demonstrate significant findings. In terms of the effects on mental‐health status, rental subsidies for the homeless population with AIDS and homeless families with one child demonstrated benefits on their mental‐health status based on their self‐reported depression scores and psychological distress levels (Wolitski, 2009; Gubits et al., 2018), but the effect did not appear significant among homeless veterans with psychiatric or addiction disorders who received housing vouchers (Rosenheck et al., 2003; Rosenheck et al., 2003). An IPS programme was reported to be associated with less self‐reported depression and ADHD symptoms as well as higher scores on a self‐reported self‐esteem scale (Ferguson, 2018). However, programmes in financial empowerment and CWT did not show significant benefits on mental‐health status (Booshehri, 2017; Kashner, 2002).

The effect of income‐assistance interventions on quality of life, substance‐use conditions and hospitalisations were not consistent across eligible studies due to diverse intervention characteristics, participant profiles, and units of measure (Rosenheck et al., 2003; Ferguson, 2018; Pankratz et al., 2017). One study on a CWT programme for homeless veterans with substance dependence found that this approach was reported to immediately reduce the consumption of alcohol and drugs within the first 3 months as well as mean number of inpatient days in medicine wards but not in psychiatric wards (Kashner, 2002).

The effect of income‐assistance interventions on employment and income improvement was reported as insignificant or uncertain. The only significant improvement on employment outcomes associated with income‐assistance interventions was reported among the homeless individuals with mental illness who were receiving the IPS, compared to those under SCM (Poremski et al., 2015). Among the six eligible studies that assessed income outcomes, o none demonstrated an association between income‐assistance interventions and increased income at the end of the study period (Booshehri, 2017; Ferguson, 2018; Gubits et al., 2018; Pankratz et al., 2017; Poremski et al., 2015; Rosenheck et al., 2003). Further research is needed to clarify the role of income‐assistance interventions on the earned incomes of homeless populations.

#### 6.1.3 Standard cases management and peer support interventions

##### Standard case management

Case‐management methods appeared to show some benefit in some of the health and social outcomes that were examined in this review. In varied cases and study conditions, SCM interventions significantly improved housing stability and reduced the odds of homelessness in homeless individuals (Conrad et al., 1998; Upshur et al., 2015; Towe et al., 2019). In contrast, some studies observed small but not significant improvements in housing outcomes with mixed effects and no significant differences between groups (Nyamathi et al., 2016; Hurlburt et al., 1996). Although the effectiveness of SCM interventions on housing outcomes varied by type, duration and the contextual conditions of those in the intervention groups, the SCM interventions had better outcomes among the programme graduates of the interventions, which underscores the fidelity to this type of intervention (Graham‐Jones et al., 2004; Upshur et al., 2015; Lapham et al., 1996). Generally, SCM interventions had mild effects on mental health by reducing the odds of depression, with very slight improvements in both psychological well‐being and psychiatric symptoms; however, the overall mental‐health status remained largely unaffected (Conrad et al., 1998; Nyamathi et al., 2001; Upshur et al., 2015; Weinreb et al., 2016). Similarly, SCM interventions had negligible or no effect on participants' quality of life or substance‐use outcomes, with some studies reporting even better improvements in the nonintervention groups (Conrad et al., 1998; Graham‐Jones et al., 2004; Nyamathi et al., 2001; Sosin et al., 1995; Upshur et al., 2015).

##### Peer support

Our systematic review yielded six studies that examined the effectiveness of peer‐support programmes. All studies were based in the United States but implemented heterogeneous approaches to providing their cohorts with peer‐support services. Two studies linked homeless individuals to peer‐support workers who matched their ethnicities (Corrigan et al., 2017; Nyamathi et al., 2001). Two trials examined peer‐support programmes that were delivered in conjunction with housing interventions (Ellison et al., 2020; Lapham et al., 1996). One trial examined a peer‐coaching programme delivered to homeless men who had been released from incarceration during a time of transition (Nyamathi et al., 2016), whereas one trial provided peer mentorship to homeless veterans seeking continuous primary care (Yoon et al., 2017).

Regardless of the nature and complexity of the peer‐support programmes that were delivered to individuals with lived experience of homelessness, our findings suggest that these interventions cannot stand alone as an approach to decreasing housing instability, alleviating the pressure of urgent health services, or assisting individuals in maintaining long‐term and proper substance‐use behaviours. Four trials examined housing stability and found no added benefits to peer‐support programmes, even when coupled with housing assistance. The reason behind this inappreciable benefit in housing stability is unclear but it could be attributed to the limited intensity and tenure of peer‐support programmes. A companion systematic review on PSH found that coupling housing programmes with more intensive supportive programmes, such as ACT or ICM, promoted long term housing stability (Aubry et al., 2020). Moreover, four trials examined substance‐use outcomes following the receipt of peer‐support programmes. Findings from these trials suggested preliminary benefits pertaining to days of substance use but that these benefits seemed to have been trivial in magnitude and to have attenuated with time. Understanding the reasons for this attenuated and inconsiderable effect requires using a realist lens to explore the programme specifics that contribute to such preliminary benefits.

Findings on mental health were mixed and polarised as one randomised controlled trial found that pairing homeless African Americans with ethnically matched peer‐support workers decreased their psychological distress and improved their overall mental‐health status (Corrigan et al., 2017). Nyamathi et al. (2001) (Nyamathi et al., 2001), however, reported that providing peer‐support services to homeless women was associated with harms pertaining to their depression and anxiety symptoms. This finding is suggestive, yet again, of a relationship between programme intensity and prospective effectiveness as a companion review found similar harms in anxiety and hostility associated with delivering SCM to homeless women (Ponka et al., 2020), whereas more intensive interventions showed promise in improving mental‐health outcomes (Ponka et al., 2020). Another explanation of these harmful effects could be linked to the nature of the relationship between peer supporters and individuals with current homelessness.

A systematic review of qualitative studies found that promoting trust is therapeutic and preferable in the context of delivering care to individuals with lived experience of homelessness (Magwood et al., 2019). More in‐depth research that explores the perceptions behind receiving support from peers is mandated. Moreover, only one randomised trial examined hospitalisation outcomes and found no significant decrease in hospitalisation rates or emergency‐department utilisation associated with receiving peer‐support services (Yoon et al., 2017). More research is needed to explore health‐service utilisation, in the context of receiving peer support, before a more conclusive decision on its benefit could be put forward.

#### 6.1.4 Mental health Interventions

Our analyses allowed us to comprehensively examine the effectiveness of a myriad of interventions and explore which carry the potential to improve the housing, health, and social statuses of individuals with lived experience of homelessness. Our findings suggest that more ICM programmes, such as ACT, ICM, and CTIs, carry the potential to improve housing outcomes. When examining mental health, quality of life, and substance use, evidence showed heterogeneous results as the majority of interventions contributed little to no improvements to these outcomes. Nonetheless, CTIs, as well as PSH coupled with ICM support, showed promise in reducing hospitalisations and emergency‐department utilisations. Finally, across all interventions, evidence on employment and income was scarce and nonsignificant, indicating the need for more trials to examine these financial‐related outcomes.

##### Intensive case management

ICM had a moderately significant positive effect on the housing stability of homeless persons, with evidence of immediate significant reductions in the number of days homeless as well as increased likelihood of being housed, by a magnitude of 5.8, with better accommodation or less time spent on the streets (Cox et al., 1998; Marshall et al., 1995; Korr & Joseph, 1996; Orwin et al., 1994; Shern et al., 2000; Shumway et al., 2008; Toro et al., 1997). However, no between‐group differences were found in long‐term reductions in homelessness in a variety of homeless populations (Burnam, 1995; Cox et al., 1998; Grace & Gill, 2014; Malte et al., 2017; Stahler et al., 1995; Toro et al., 1997). The findings on ICM interventions on mental health were mixed. In ICM groups we found modest reductions in psychiatric symptoms as well as psychological symptoms (deviant behaviour), youth self‐reports of depression problems, and total behavioural problems but not of antisocial behaviour (Cauce, 1994; Marshall et al., 1995; Toro et al., 1997). In contrast, other studies reported no notable between‐group differences in self‐esteem or mastery scores, youth's self‐reports on depression, antisocial behaviour on the Problem Behaviour Scale (PBS) or total behavioural problems (Clark & Rich, 2003; Felton et al., 1995; Marshall et al., 1995; Malte et al., 2017; Orwin et al., 1994; Shumway et al., 2008; Stahler et al., 1995). On the quality of life, ICM had a small effect with slight improvements in the quality of life and self‐esteem of homeless adolescents 13–21 years old, and satisfaction with living situations, finances as well as fewer life‐problems sub‐scores in ICM groups (Braucht 1995; Cauce 1994; Felton et al., 1995). Some studies reported no major changes in quality‐of‐life scores or better outcomes in the control group (Marshall et al., 1995; Shern et al., 2000). On substance use, ICM intervention had a small effect on substance‐ and drug‐use outcomes. Two studies found significantly (*p* < .05) lower composite scores for alcohol or recent alcohol and cocaine use across groups (Cox et al., 1998; Stahler et al., 1995); Other studies reported minimal improvements in problematic substance use or no significant between‐group differences in measures of alcohol or drug use (Burnam, 1995; Cauce, 1994; Malte et al., 2017; Orwin et al., 1994; Stahler et al., 1995; Toro et al., 1997). In three trials, ICM interventions resulted in reductions in hospitalisations and health‐care utilisation (Marshall et al., 1995; Rosenblum et al., 2002; Shumway et al., 2008). Overall ICM interventions showed no significant effects on participants staying employed (Braucht 1995; Cox et al., 1998; Orwin et al., 1994; Stahler et al., 1995). Regarding participants' income, some group effects favouring ICM groups using monthly public income assistance were found; however, there were no significant between‐group differences (Rosenblum et al., 2002; Toro et al., 1997).

##### Assertive community treatment

Overall, ACT interventions had a moderately positive effect on housing stability in homeless persons with mental illness who were treated with either continuous ACT or other ACT modalities—ACTO, IACT, NIACT (Clarke et al., 2000; Fletcher et al., 2008; Morse et al., 1997, 2006, 2008). Similarly, two trials found significant reductions in psychiatric symptoms or improved psychoticism subscales over time (Essock et al., 1998; Lehman et al., 1997). However, some studies that reported noticeable improvements in mental health did not find any group differences favouring using ACT interventions (Essock et al., 2006; Fletcher et al., 2008; Morse et al., 1997; Morse et al., 1997; Morse et al., 2006). On quality‐of‐life measures, the ACT intervention demonstrated some positive but not significant effects on general life satisfaction and QOL subscales, such as leisure and personal safety (Essock et al., 1998; Essock et al., 2006; Lehman et al., 1997). In the majority of the ACT trials, there were no significant improvements in alcohol consumption or substance use over time (Morse et al., 1992, 1997, 2006; Essock et al., 2006; Fletcher et al., 2008), except in one trial which found significant reductions in the alcohol‐, drug‐ and substance‐use and substance‐abuse scales in favour of ACT over SCM (Essock et al., 2006). Findings on alcohol or drug use were likely influenced by the use of varied tools of assessment as well as the rating scale used. Apparently, ACT interventions reduced days hospitalised or emergency‐department visits, among participants, compared to SCM interventions (Clarke et al., 2000; Essock et al., 1998; Essock et al., 2006; Lehman et al., 1997). Subsequently, ACT interventions had no significant impact on income compared to standard service or care (Morse et al., 1992, 1997). No evidence was found on the effects of ACT on employment outcomes in the selected trials.

##### Critical time intervention

Our systematic review yielded 5 studies on CTI. One study specifically explored family critical time intervention (FCTI) in a multidisciplinary community‐based service model targeted towards families and aiding in connecting them with social services and in forming supportive relationships with families and friends during critical times of transition from a shelter to community housing (Shinn et al., 2015).

Three of the four studies found that CTI significantly improved housing stability and reduced days spent homeless (Susser et al., 1997; Shinn et al., 2015) One study found a significant reduction in the amount of extended homelessness (more than 54 nights) specifically, with a relative risk of 0.53 (95% CI Taylor series, 0.27–1.01) (Susser et al., 1997). Within the trial that explored FCTI, families in the FCTI treatment group (in which the mother had a diagnosable mental illness or substance use problem) spent more time in community housing and had a lower average number of days until they moved into stable housing (91 compared to 199 days) (Shinn et al., 2015). One trial based out of the Netherlands did not find a significant difference in the number of days rehoused with CTI compared to TAU. The lack of significance of CTI vs TAU was thought to be partially explained by the very low rate of recurrent homelessness in both the CTI and care‐as‐usual treatment arms in this study (de Vet, 2017). The Netherlands' extensive social housing programme and increased intensity of follow up for those in usual care, compared to the United States, may have contributed to the low recurrent homelessness in both groups and the subsequent lack of significance of the type of intervention care (de Vet, 2017).

The results of CTI compared to TAU on mental health were mixed. Two studies found no significant difference in psychological distress, though in one study CTI had an added differential effect on psychological distress for participants experiencing less social support, suggesting that CTI may have more benefit in less‐supported individuals (de Vet, 2017). There was also no difference in mothers' mental health in the FCTI intervention compared to the TAU, though both the intervention and the control arms had significant improvements, over time, in mental‐health symptomatology. One study found that CTI appeared to significantly improve the symptoms of PTSD during follow up (adjusted MD, 7.27; 95% CI, −14.31 to −0.22, *p *= .04) Lako et al., 2018. Another trial demonstrated that CTI may also have a benefit for negative symptoms of schizophrenia (Herman et al., 2011). This finding was particularly intriguing, as negative symptoms of schizophrenia have a known disabling effect on patients' quality of life and are often extremely difficult to treat with medications (Correll & Schooler, 2020). Hierarchical regression analysis in this trial could not explain this improvement, by accounting for the reduction in homelessness. The authors proposed that CTI may provide “cognitive remediation”, encouraging participants to carry out executive tasks, which potentially re‐activated prefrontal cortical functions that were previously underutilized. Alternatively, the provision of social support and strengthening of relationships within times of critical transition may improve negative symptoms by improving feelings of hopelessness and loneliness (Herman et al., 2011). The findings of CTI benefits on particularly vulnerable subgroups—that is, participants with less social support, PTSD or schizophrenia—is important and requires further study.

Within children, one study found that FCTI appeared to reduce internalising behaviours and externalising barriers in children up to 5 years old (Shinn et al., 2015). For children between 6 and 10 years old, there were no effects of the intervention on mental health but both the CTI and usual‐care treatment groups had significant improvements over time, for mother‐reported internalising and externalising behaviours and child‐reported depressive symptoms. In adolescents aged 11–16 years, the FCTI group had a reduction in mother‐reported externalising behaviour, and this was not seen in the usual‐care group. At 24 months, the difference between these two groups was 6.2 (0.5 SD). Children's self‐report found no difference in externalising behaviours (Shinn et al., 2015). This trial suggests that FCTI has benefits beyond improving maternal symptomatology; further research is required to examine the effects of FCTI in children.

Two studies examined the effect of CTI on quality of life and found no between‐group differences during follow up (Lako et al., 2018; de Vet 2017). In one of these studies, however, the fidelity of results may have been impacted by the implementation of strengths‐based approaches in shelters just before the start of the study. Within this approach, a significant amount of post‐discharge services were also delivered to a majority of members in the control group, limiting the ability to detect between‐group outcome differences. (Lako et al., 2018). There was a trend towards reduced substance use in the CTI group, though it did not reach significance (De Vet 2017). Of note, however, in this study, baseline characteristics between the CTI versus the control group differed, with a significantly higher proportion of participants in the CTI group reporting excessive alcohol use in the past 30 days (21%), compared to the control group (20%) at baseline. Differences in baseline differences may have subsequently had an impact on the lack of significance of substance use outcome at follow up.

One study by Tomita (2012) found that participants receiving CTI had significantly reduced odds of psychiatric re‐hospitalisations during the final three observation intervals (OR = 0.11; 95% CI, 0.01–0.96). This impact was noted even after the completion of CTI intervention, and persisted even after controlling for housing stability. Reduced psychiatric re‐hospitalisation may be related to the impact of CTI on increasing social and community support and facilitating connection to external resources (Tomita 2012). In one study, FCTI participants (homeless mothers with high mental‐health needs) used more mental‐health services than the control group. These included mental‐health treatment in a hospital/clinic where the participant stayed overnight, services to help deal with a crisis, individual and/or group mental‐health counselling/therapy, services of a psychiatrist or other medical practitioner for the monitoring of psychiatric medications, and services of a case manager or some other person to help coordinate mental‐health services (Shinn et al., 2015; Susser et al., 1997). Another study examined mental‐health‐service utilisation (Susser et al., 1997) but provided only preliminary data; in this study, mental‐health utilisation was similar between the experimental groups but the CTI group used more services within each group than the control, particularly within outpatient clinics. These findings offer further support to the impact of CTI in connecting participants with mental health and community resources, during times of critical transition.

#### 6.1.5 MAPs, SCFs and pharmacological interventions

This systematic review was intended to assess the effectiveness of SCFs, MAPs, pharmacological interventions for opioid use disorder and injectable antipsychotics for people who are homeless or vulnerably housed. No evidence from trials was identified; 20 articles were considered potentially relevant and were excluded. As SCFs begin to emerge, studies on such pilot projects are often observational due to ethical concerns, but often include a large proportion of homeless participants (Magwood, 2020). Existing reviews on SCFs primarily include observational studies (Kennedy et al., 2017; Potier et al., 2014). Studies of the effectiveness of MAPs have historically been conducted as case studies and small sample pilot projects that target individuals with severe alcohol dependence or who consume nonbeverage alcohol, as reported in one identified empty systematic review on MAPs (Muckle et al., 2012). Finally, the effectiveness of pharmacotherapeutic interventions is rarely assessed in transient and hard to reach populations. These results accurately reflect the existing evidence of MAPs, SCFs and pharmacotherapeutic interventions among homeless or vulnerably housed populations, as experimental evidence is often scarce in this transient population. To fill this gap, we have recently published an overview of reviews on this topic (Magwood, 2020).

### 6.2 Overall completeness and applicability of evidence

#### 6.2.1 Permanent supportive housing

This systematic review presents evidence that PSH increases housing stability compared to usual care, promotes earlier exits from homelessness and fewer days spent homeless. Most study populations possessed a high degree of comorbid mental health and/or substance use, suggesting that PSH can be helpful even within high‐needs populations. The reproducibility of “Housing First” strategies across multiple cities demonstrates that this intervention has adaptability and relevance across multiple contexts.

Previously, access to housing was contingent on the attainment of abstinence and/or treatment and the stabilisation of underlying mental‐health disorders. Instead, our systematic review provides support for a “Housing First” strategy, advocating for the separation of housing and clinical services and the immediate provision of housing, with the aim of reducing homelessness, facilitating community integration and providing support and treatment with a recovery orientation (Aubry 2015). Long‐term data, up to 5.4 years, was examined in one single‐city site (Stergiopoulos et al., 2019); more research is required to further investigate the long‐term implications and effects of PSH.

Individuals who have a lived experience of homelessness are a diverse and heterogeneous population with different life courses and experiences that have shaped their vulnerabilities and resiliency (Aubry et al., 2012). For instance, individuals who become homeless at an older age tend to have less substance‐use and mental‐health comorbidities, compared to younger adolescents with a lived experience of homelessness (Aubry et al., 2016). Further research should focus on identifying specific populations and demographic groups within the overall group of persons with lived experiences of homelessness, who might benefit from targeted interventions, including improved success with PSH. Some subgroup analyses of gender and adolescent versus older participants were presented in this review, but this was sometimes under powered and hence primarily served as exploratory analyses. Mental health, quality of life, substance use, hospitalisations, income‐ and employment‐status outcomes would also benefit from further research within dedicated subgroup populations. Prior studies have shown some differences in outcomes between demographic groups. For instance, Tsemberis et al. (2004) found there were significant differences in rates of psychiatric‐symptom change over time, suggesting that decreases in symptomatology were more rapid for some individuals compared to others. A study within an ethnically diverse population in Toronto found differences in outcomes between racialized and nonracialized participants as well as foreign‐born vs Canadian‐born individuals (Stergiopoulos et al., 2015). Tsemberis et al. (2004) found that the HF programme was most successful in reducing hospitalisations for samples of people recruited from psychiatric hospitals, compared to those recruited from the street, who showed an overall low level of hospitalisations throughout the study period. Hence, research gaps that may be pursued in the future could include an examination of the differential magnitude of the effects of PSH based on age and gender, as well as among disabled persons and within minority groups.

Further study is also required to delineate improvements of PSH on quality of life, substance use, income and employment (as findings from existing studies are either limited or quite mixed). There is some limited evidence suggesting that PSH participants have improvements in their subjective condition that is specific to quality of life, compared to those receiving standard care. Only one study examined hybrid approaches to PSH (integrative vs continuum housing) (McHugo et al., 2004), though practical concerns of the actualisation of Housing‐First strategies may result in some policy‐makers favouring this strategy.

#### 6.2.2 Income assistance

Income assistance interventions have been much less reviewed than other socioeconomic interventions due to the following challenges: (1) the complexity of income‐assistance interventions and the difficulty of isolating the specific effects from the impact of other socio‐demographic factors on populations with coexisting social and economic needs; (2) challenges in study design in terms of being able to assess the impacts on subjective outcomes (e.g., quality of life), which often requires significant follow up or funding to detect meaningful effect size; (3) individual outcome measurements can be affected by political‐economic dynamics and systemic barriers within a designed study period (Shahidi et al., 2019). Recently Aubry et al. (2020) appraised the evidence on the effect of income‐assistance interventions on the social and health outcomes and reported the promising positive effect of housing subsidies with case management on housing stability. However, evidence on the effects of income‐assistance interventions for the homeless populations remains inconclusive and lacking realist review on the long‐term effects and the interaction with other social service components for the homeless population (Pawson et al., 2005). Our current review assessed the effect of income‐assistance interventions in the context of health and social outcomes and provided an up‐to‐date summary of a growing body of primary research on the effect of income‐assistance interventions over the past decades. Inconsistent findings were reported on those programmes that were associated with income‐assistance interventions that ranged from rental assistance in the form of housing subsidies (Gubits et al., 2018; Hurlburt et al., 1996; Pankratz et al., 2017; Rosenheck et al., 2003; Wolitski, 2009), IPS (Ferguson, 2018; Poremski et al., 2015), housing assistance with income support (Forchuk et al., 2008), financial education (Booshehri 2017), to CWT (Kashner, 2002). The diversity of the intervention characteristics, participant profiles, and outcome measurements contributed to the inconsistency of the effectiveness. There was a lack of standardised controls or comparisons across the eligible studies. Extra caution is required when interpreting the results for future implications.

#### 6.2.3 SCM and peer support interventions

##### Standard case management

The nature of the case‐management interventions in this review varied in the intensity of the programme and study population, with the majority of the trials conducted in the United States. Previously, evidence on the SCM model was associated with short‐term outcomes but limited effects regarding treatment retention, employment, and housing stability in individuals with both mental illness and substance use disorders. Our review findings were similar, with little or no significant long‐term benefit across the study outcomes such as housing stability, mental health, and substance use. In contrast, a study reported improvements in housing stability, reduced substance use and employment barriers in homeless substance users, evidence in our study supported only a short‐term positive effect on housing stability (Magwood et al., 2019). In one trial, SCM was associated with elevated levels of hostility and depression in a homeless female population (Nyamathi et al., 2001).

##### Peer support

Our systematic review examined six studies that provided mixed evidence on the effectiveness of peer support interventions among individuals with lived experience of homelessness. This inconsistency could, very well, be attributed to the heterogeneity in the design and implementation of peer support programmes. Two studies used peer coaches to provide support to homeless individuals in the context of receiving temporary housing (Ellison et al., 2020; Lapham et al., 1996), and even though we were able to isolate the effect of this peer support component from the housing programme, we were not able to determine whether the conjugation of temporary housing to peer support may have put study participants in an advantage to benefit from this intervention. In another two studies (Corrigan et al., 2017; Nyamathi et al., 2001), peer supporters were recruited to reflect participants' ethnic background, but this ethnic matching did not seem to promote added benefit among individuals with lived experience of homelessness. Across all studies, assigning participants to peer support programmes was not conclusively associated with housing, mental health, or social improvements relative to usual care, which suggests that peer support cannot serve as a stand‐alone intervention to promote stability in these outcomes. More research, however, is needed to explore whether coupling peer support with more permanent housing programmes could improve housing and health.

#### 6.2.4 Mental health interventions

Our systematic review examined mental health interventions that supported people with mental illness and substance use disorders who were also in homeless populations and was similar to a recent review by Ponka et al. (2020) and an earlier review by De Vet (2013). To our knowledge, this systematic review offers a comprehensive evaluation of effectiveness and outcomes of interventions including ICM, ACT and CTI; and incorporated wider definitions for specific interventions and studies from other high‐income countries and regions reporting evidence on case management in homeless populations as global public health problems. In addition, Aubry (2020) found evidence that case management intervention can have significant benefits when provided in conjunction with PSH (Aubry et al., 2020). Our review findings agree with reports of the increased rate of initiation, engagement retention, reduced hospital visits, and better treatment outcome with twice as many clients with substance use problems treated with ICM, although client's hospital stays remained same with TAU groups (Morgenstern et al., 2006; Kuerbis, 2011/03; Stergiopoulos et al., 2015). However, only ICM was found to consistently improve income outcomes, with significant improvements in access to financial assistance and reductions in unmet financial needs.

Our study supports strong evidence on the effectiveness of ACT in homeless clients with both mental health and substance use disorders and demonstrates substantial benefits with better outcomes such as housing stability, quality of life as well as lesser hospital stays and emergency visits. ACT interventions were cost‐effective offering both crisis intervention and psychosocial interventions for persons with complex needs such as persons with severe mental illness or dual disorders and homeless. Hence, there are cost benefits when the overall health‐care systems and society are considered, however, some researchers find the approach somewhat authoritarian, forced and inconsistent with recovery‐oriented mental health practice (Tsai et al., 2011). Furthermore, CTI appears to reduce homelessness and increase days spent in stable housing, with three out of four studies showing improvements in housing stability (de Vet, 2017). Also, CTI may have a differential benefit in reducing symptoms of PTSD and negative symptoms of schizophrenia, though more research should be done to confirm these findings. The effectiveness of family critical time intervention (FCTI) showed some promising effects on the impact of homelessness, with mixed effects on reducing internalising/externalising behaviours in children in a single study. While we identified positive impacts in housing stability in the homeless population using interventions with greater intensity like ICM, ACT and CTI, our review found insufficiency studies on the effects of ACT and CTI on income and employment outcomes. Also, the heterogeneity of these complex interventions, participant profiles, coupled with the inexactness in the mechanisms and key features that promote effectiveness varies which presents a challenge in the assessment of outcomes as well as contributes to the variance in effectiveness. Regardless, the effectiveness of strength‐based case‐management and mental health interventions appears to be related to both the intensity of the models and the fidelity of the programmes to comprehensively address the needs of homeless populations, particularly those with severe mental health conditions or who are experiencing transitions in care (Dieterich, 2017). Again, the more intensive models of case management (ICM or ACT) and with lower caseloads and potential longer engagement with case‐managers provide services that extend beyond care coordination and outreaches, therefore have a greater chance and effects in improving causal social determinants of health that maintain the cycle of homelessness (De Vet, 2013; Ponka et al., 2020). Another critical factor is the role of caseworkers as advocates for clients' access to income benefits and locating housing options leads to improvements in housing stability (Sosin et al., 1995). Also, of particular interest, is the role of trust in the case‐manager‐client relationship as well the continuity of care being an integral element in the efficacy of case‐management programmes (Magwood et al., 2019). Other capable variants of the programmes with unique and novel case‐manager roles that include peer‐led initiatives and people with lived experience appear promising in addressing the cycle of homelessness in populations, but these needs further evaluation (Tsai et al., 2011; Greene et al., 2015; Gagne et al., 2018). Our findings suggest that case management and mental health interventions need to be continuous, community‐based and intensive to maintain and increase the gains achieved. These findings are in agreement with other previously published reviews, such as Mueser (1998), Coldwell & Bender (2007), Hwang (2005); and Vanderplasschen et al., (2007), respectively.

### 6.3 Quality of the evidence

Risk of bias in included studies provides a detailed description, and summary, of primary study bias judgements. Furthermore, these risk of bias assessments were considered as part of the GRADE assessment of the certainty of evidence. We assessed the certainty of evidence for housing stability outcomes across all interventions. Overall, the certainty of evidence for interventions aimed at reducing homelessness ranged from very low to moderate. For PSH, there is moderate certainty evidence that PSH increases housing stability (summary of findings Table 1). This evidence comes from two randomised trials with concerns regarding selection, performance and detection biases. Low certainty evidence on income assistance interventions reduced episodes of homelessness and increased number of days stably housed (summary of findings Table 2). Certainty of evidence pertaining to SCM (summary of findings Table 3), ICM (summary of findings Table 4) and CTIs (summary of findings Table 6) were also deemed low. Finally, the evidence on housing stability outcomes from ACT (summary of findings Table 5) and peer support interventions (summary of findings Table 7) ranged from low to very low certainty, often due to single studies with small sample sizes (<300 participants) and few events (<100 observations).

**Table 1:** Housing

Certainty assessment							No of patients		Effect			
No of studies	Study design	Risk of bias	Inconsistency	Indirectness	Imprecision	Other considerations	HF + ACT/ICM	UC	Relative (95% CI)	Absolute (95% CI)	Certainty	Importance
Number of participants in stable housing long term (Aubry et al., 2016 ^(13)^; HF + ACT, Stefancic, 2007^(64)^; HF + ACT) (follow up: +20 months)												
2	Randomised trials	Serious*	Not serious	Not serious	Not serious	None	376/578	152/388	OR 3.58 (2.36–5.43)	306 more per 1000 (from 211 more to 386 more)	⊕⊕⊕◯ MODERATE	CRITICAL
Percentage of participants in stable housing long term (Aubry et al., 2016; ^(13)^ HF+ACT) (follow up: 24 months)												
1	Randomised trial	Serious^a^	Not serious	Not serious	Not serious	None	273/369 (74%, CI 69%‐78%)	138/337 (41%, CI 35%‐46%)	OR 4.10 (2.98–5.63)	330 more per 1000 (from 264 more to 387 more)	⊕⊕⊕◯ MODERATE	CRITICAL
									RR 1.81 (1.58–2.08)			
Total number of days housed long term (Aubry et al., 2016; ^(13)^ HF+ACT, Stergiopoulos et al., 2015; ^(56)^ HF+ICM) (follow up: 24 months)												
1	Randomised trial	Serious^a^	Not serious	Not serious	Not serious	None	411 (280.74 days, SD278.92)	369 (115.33 days, SD191.43)	‐	AAD 161.8 more days (82.5 more to 241.1 more)	⊕⊕⊕◯ MODERATE	CRITICAL
1	Randomised trial	Serious^a^	Not serious	Not serious	Not serious	None	689	509	‐	SMD 1.38 more days (1.03 more to 1.73 more days)	⊕⊕⊕◯ MODERATE	CRITICAL
Percentage of days stably housed over 24 months (Aubry et al., 2016; ^(13)^ HF+ACT, Stergiopoulos et al., 2015; ^(56)^ HF+ICM)												
1	Randomised trial	Serious^a^	Not serious	Not serious	Not serious	None	469	481	At Home/Chez Soi: High needs participants (HF+ACT): Adjusted absolute difference [AAD] = 42%; 95% CI [38%‐45%; p<0.01] (Aubry et al., 2016).		⊕⊕⊕◯ MODERATE	CRITICAL
1	Randomised trial	Serious^a^	Not serious	Not serious	Not serious	None	689	509	Moderate needs participants (HF+ICM): Adjusted mean difference:		⊕⊕⊕◯ MODERATE	CRITICAL
									Site A, 33.0% [95% CI, 26.2–39.8%];			
									Site B, 49.5% [95% CI, 41.1–58.0%];			
									Site C, 35.6% [95% CI, 29.4–41.8%];			
									Site D, 45.3% [95% CI, 38.2–52.5%]; (Stergiopoulos et al., 2015). (C).			

Abbreviations: AAD, adjusted absolute difference; CI, confidence interval; OR, odds ratio; MD, mean difference; RR, risk ratio.

* One study reported unclear risk of selection bias (random sequence generation and allocation concealment) and unclear risk of detection bias (blinding of outcome assessment).^(64)^ One study showed high risk of detection bias (blinding of outcome assessment),^(13)^ both study showed high risk of performance bias (blinding of participants and personnel)^(64, 13)^.

^a^ High risk of performance bias (blinding of participants and personnel), and high risk of detection bias (blinding of outcome assessment).^(13)^

**Table 2:** Income assistance

Certainty assessment							No.of patients		Effect		Certainty	Importance
No. of studies	Study design	Risk of bias	Inconsistency	Indirectness	Imprecision	Other considerations	Intervention	Comparison	Relative (95% CI)	Absolute (95% CI)		
Number of homeless episodes (Compensated Work Therapy (CWT) versus control services; Kashner, 2002 ^(23)^)												
1	Randomised trials	Serious^a^	Not serious	Not serious	Serious^b^	None^c^	Not reported	Not reported	OR 0.10 (0.1–0.3)	‐	⊕⊕◯◯ LOW	CRITICAL
Number of days spent homeless (HUD Section 8 housing vouchers paired with ICM versus treatment as usual; Rosenheck 2003 ^(12)^)												
1	Randomised trials	Serious^d^	Not serious	Not serious	Serious^e^	None	182 (13.5 days)	188 (20.45 days)	‐	36.2% fewer days (*p *< .001)	⊕⊕◯◯ LOW	CRITICAL
Number of days stably housed (HUD Section 8 housing vouchers paired with ICM versus treatment as usual; Rosenheck, 2003 ^(12)^)												
1	Randomised trials	Serious^d^	Not serious	Not serious	Serious^e^	None	182 (59.39 days)	188 (47.60 days)	‐	25% more days (*p *< .001)	⊕⊕◯◯ LOW	CRITICAL

Abbreviations: CI, confidence interval; OR, odds ratio.

^a^ High risk of performance bias (blinding of participants and personnel), and high risk of detection bias (blinding of outcome assessment).^(23,83)^

^b^ Number of events at follow up not reported. Number needed to treat cannot be calculated.^(23)^

^c^ Large effect, however consistent evidence from at least 2 studies required for upgrade.^(23)^

^d^ High risk of performance bias (blinding of participants and personnel) and high risk of attrition bias (incomplete outcome data)^.(12)^

^e^ Measure of variance and confidence intervals not reported. No further data available from the authors.^(12,83)^

**Table 3:** Standard case management

Certainty assessment							No. of patients		Effect		Certainty	Importanc
No. of studies	Study design	Risk of bias	Inconsistency	Indirectness	Imprecision	Other considerations	Case management	Comparison	Relative (95% CI)	Absolute (95% CI)		
Percentage of participants homeless (Shumway et al., 2008) (follow up: 24 months)												
1	Randomised trials	Serious^a^	Not serious	Not serious	Serious^b^	None	38/141 (27.0%)	21/85 (24.7%)	OR 1.12 (0.61–2.08)	26 more per 1000 (from 101 fewer to 177 more)	⊕⊕◯◯ LOW	CRITICAL
Percentage of participants who lived in one residence in the last 3 months (Upshur et al., 2015) (follow up: 6 months)												
1	Randomised trials	Serious^c^	Not serious	Not serious	Serious^b^	None	9/40 (22.5%)	16/36 (44.4%)	OR 0.36 (0.13–0.97)	221 fewer per 1000 (from 350 fewer to 8 fewer)	⊕⊕◯◯ LOW	CRITICAL
Percentage of participants who lived in three residences or more in the last 3 months (Upshur et al., 2015) (follow up: 6 months)												
1	Randomised trials	Serious^c^	Not serious	Not serious	Serious^b^	None	12/40 (30.0%)	3/36 (8.3%)	OR 4.71 (1.20 to 18.39)	216 more per 1000 (from 15 more to 542 more)	⊕⊕◯◯ LOW	CRITICAL
Mean number of days in stable residence (Sosin et al., 1995) (follow up: 6 months)												
1	Randomised trials	Serious^a^	Not serious	Not serious	Serious^d^	None	‐	‐	The case management intervention increases residential stability by a statistically significant 9 days (8.650; *t* = 2.35; *p *< .05)		⊕⊕◯◯LOW	CRITICAL

^a^ High risk of performance bias, and unclear risk of detection bias.

^b^ Too few events (<300).

^c^ Unclear risk of selection bias and attrition bias, high risk for performance bias.

^d^ Too few participants (<400).

**Table 4:** Intensive case management

Certainty assessment							No. of patients		Effects			
No. of patients	No. of studies	Risk of bias	Inconsistency	Indirectness	Imprecision	Other considerations	Case Management	Comparison	Relative (95% CI)	Absolute (95% CI)	Certainty	Importance
Number of days homeless (Cox et al., 1998, Grace & Gill, 2014, Toro et al., 1997) (follow up: 13+ months)												
3	Randomised trials	Very serious^a^	Not serious	Not serious	Not serious	None	358	308		SMD 0.22 fewer (0.4 fewer to 0.03 fewer)	⊕⊕◯◯ LOW	CRITICAL
Proportion of participants housed (Korr & Joseph, 1996) (follow‐up: 6 months)												
1	Randomised trials	Serious^b^	Not serious	Not serious	Serious^c^	None	36/48 (75%)	15/44 (34.1%)	OR 5.80 (2.35 to 14.31)	409 more per 1000 (from 208 more to 540 more)	⊕⊕◯◯ LOW	CRITICAL
Time spent on the street (Shern et al., 2000) (follow‐up: 24 months)												
1	Randomised trials	Serious^d^	Not serious	Not serious	Serious^e^	None	91	77	‐	MD 26.71 fewer (39.21 fewer to 14.2 fewer)	⊕⊕◯◯ LOW	CRITICAL
Time spent in shelters (Shern et al., 2000) (Follow‐up: 24 months)												
1	Randomised trials	Serious^d^	Not serious	Not serious	Serious^e^	None	91	77	‐	MD 20.29 more (13.38 more to 27.19 more)	⊕⊕◯◯ LOW	CRITICAL
Time spent in community housing (Shern et al., 2000) (follow‐up: 24 months)												
1	Randomised trials	Serious^d^	Not serious	Not serious	Serious^e^	None	91	77	‐	MD 11.07 more (1.52 more to 20.61 more)	⊕⊕◯◯ LOW	CRITICAL

Abbreviations: CI, confidence interval; MD, mean difference OR, odds ratio; SMD, standardised mean difference.

^a^ Two trials with unclear selection bias and one trial with high risk for selection bias (non randomised). Two trials with unclear detection bias, one trial with high risk of detection bias. Two studies with high risk of attrition bias.

^b^ Unclear risk of selection bias, high risk of performance bias, unclear risk of detection bias, and high risk of attrition bias.

^c^ Small sample size, <100 events, wide confidence interval.

^d^ Unclear selection bias, unclear detection bias, and high risk for performance bias.

^e^ Small sample size, <300 participants.

**Table 5:** Assertive community treatment

Certainty assessment							No. of patients		Effect			
No. of studies	Study design	Risk of bias	Inconsistency	Indirectness	Imprecision	Other considerations	Case management	Comparison	Relative (95% CI)	Absolute (95% CI)	Certainty	Importance
Number of days homeless on streets (Lehman 1997) (follow up: 12 months)												
1	Randomised trials	Very serious^a^	Not serious	Not serious	Serious^b^	None	77	75		MD 14.2 fewer(28.75 fewer to 0.35 more)	⊕◯◯◯ VERY LOW	CRITICAL
Number of days homeless in the previous month (Morse et al., 1992) (follow‐up: 12 months)												
1	Randomised trials	Serious^c^	Not serious	Not serious	Serious^b^	None	52	62	‐	MD 8.11 days fewer (12.32 fewer to 3.89 fewer)	⊕⊕◯◯ LOW	CRITICAL
Number of days homeless in shelter (Lehman 1997) (follow up: 12 months)												
1	Randomised trials	Very serious^a^	Not serious	Not serious	Serious^b^	None	77	75		MD 6.2 fewer (9.5 fewer to 2.89 fewer)	⊕◯◯◯ VERY LOW	CRITICAL
Number of days in community housing (Lehman 1997) (follow up: 12 months)												
1	Randomised trials	Very serious^a^	Not serious	Not serious	Serious^b^	None	77	75	‐	MD 50.1 more (46.15 more to 54.04 more)	⊕◯◯◯ VERY LOW	CRITICAL
Number of days in stable housing in previous month (Morse et al., 2006) (Follow‐up: 24 months)												
1	Randomised trials	Serious^d^	Not serious	Not serious	Serious^b^	None	54	49	‐	MD 5.7 days more (0.59 more to 10.8 more)	⊕⊕◯◯ LOW	CRITICAL

Abbreviations: CI, confidence interval; MD, mean difference; OR, odds ratio; SMD, standardised mean difference.

^a^ High risk of performance bias, attrition bias and reporting bias. Unclear risk of selection bias and detection bias.

^b^ Small sample size (<300 participants).

^c^ Unclear risk of selection bias (random sequence generation and allocation concealment), high risk of performance bias (blinding), and unclear risk of detection bias (outcome assessment).

^d^ High risk of performance bias, unclear risk of selection and detection bias.

**Table 6:** Critical time intervention

Certainty assessment							No of patients		Effect		Certainty	Importance
No of studies	Study design	Risk of Bias	Inconsistency	Indirectness	Imprecision	Other considerations	Case management	Comparison	Relative (95% CI)	Absolute (95% CI)		
Odds of homelessness at the end of the follow‐up period (the final three 6‐week intervals) (Herman et al., 2011) (follow up: 18 months)												
1	Randomised trial	Serious^a^	Not serious	Not serious	Serious^b^	None	3/58 (5.2%)	11/59 (18.6%)	OR 0.22 (0.06–0.88)	138 fewer per 1000 (from 173 fewer to 19 fewer)	⊕⊕◯◯ LOW	CRITICAL
Mean number of: days rehoused (De Vet, 2017) (follow up: 9 months)												
1	Randomised trial	Serious^c^	Not serious	Not serious	Serious^d^	None	80	82	‐	MD 8.29 fewer	⊕⊕◯◯ LOW	CRITICAL
Percentage of participants who were homeless at the last month of trial (Susser et al., 1997) (follow up: 18 months)												
1	Randomised trial	Serious^e^	Not serious	Not serious	Serious^b^	None	4/48 (8.3%)	11/48 (22.9%)	OR 0.30 (0.08–1.04)	147 fewer per 1000 (from 206 fewer to 7 more)	⊕⊕◯◯ LOW	CRITICAL
Odds of extended homelessness—more than 54 nights (Susser et al., 1997) (follow up: 18 months)												
1	Randomised trial	Serious^e^	Not serious	Not serious	Serious^b^	None	10/48 (20.8%)	19/48 (39.6%)	OR 0.40 (0.16–0.99)	188 fewer per 1000 (from 301 fewer to 2 fewer)	⊕⊕◯◯ LOW	CRITICAL
Earned income in the second nine months (Jones et al., 2003) (follow up: 18 months)												
1	Randomised trial	Serious^e^	Not serious	Not serious	Serious^d^	None	47	44	‐	MD 27 more (198.36 fewer to 252.36 more)	⊕⊕◯◯ LOW	IMPORTANT

Abbreviations: CI, confidence interval; MD, mean difference, NR, not reported; OR, odds ratio; SMD, standardised mean difference.

^a^ High risk of selection bias and blinding of participants and personnel.

^b^ Less than 100 events.

^c^ High risk of blinding of participants and personnel (performance bias), and high risk of blinding of outcome assessment (detection bias).

^d^ Small sample size (<300 participants).

^e^ Unclear risk of selection and high risk of performance bias.

**Table 7:** Peer support

Certainty assessment							No. of patients		Effect		Certainty	Importance
No. of studies	Study design	Risk of bias	Inconsistency	Indirectness	Imprecision	Other considerations	Peer support	Usual care	Relative (95% CI)	Absolute (95% CI)		
Rates of homelessness (Corrigan et al., 2017) (follow up: 12 months)												
1	Randomised trials	Serious^a^	Not serious	Not serious	Serious^b^	None	31/34 (91.2%)	28/33 (84.8%)	OR 1.84 (0.40 to 8.43)	63 more per 1,000 (from 157 fewer to 131 more)	⊕⊕◯◯ LOW	CRITICAL
Number of days that participants spent housed in the past month (Ellison et al., 2020) (follow up: 12 months)												
1	Randomised trials	Serious^a^	Not serious	Not serious	Serious^b^	None	There was no significant difference between the intervention and usual care groups across 12 months (p>.05)				⊕⊕◯◯ LOW	CRITICAL
Number of days that participants spent in stable housing in the past month (Lapham et al., 1996) (follow up: 10 months)												
1	Randomised trials	Very serious^c^	Not serious	Not serious	Serious^d^	None	Assignment to interventions with a peer support component was not significantly associated with increased housing stability compared to assignment to temporary housing alone (intervention coefficient estimate = 11.0 (SE 1.7), control coefficient estimate = 13.3 (SE 2.0), overall model Coefficient of determination *R* ^2^ = .09; p>.001)				⊕◯◯◯ VERY LOW	CRITICAL
Percentage of participants who were homeless (street/shelter) (Nyamathi et al., 2016) (follow up: 12 months)												
1	Randomised trials	Very serious^e^	Not serious	Not serious	Serious^d^	None	20/177 (11.3%)	19/186 (10.2%)	OR 1.14 (0.59 to 2.23)	13 more per 1000 (from 39 fewer to 100 more)	⊕◯◯◯ VERY LOW	CRITICAL

^a^ High risk of performance bias, unclear risk of detection bias, randomisation, and allocation concealment.

^b^ Too few participants (<400).

^c^ High risk of performance and attrition biases and high risk of deviation from assignment to intervention. Unclear risk of detection bias.

^d^ Too few event (<300).

^e^ High risk of performance bias and reporting bias, and unclear risk of detection bias.

Overall, for PSH interventions, there is moderate confidence in the estimated effect of housing stability, the true effect is likely to be close to the estimated effect, but there is a possibility that it is substantially different. For all other interventions (income, SCM, ICM, ACT, and peer support) there is very little to limited confidence in the estimated effect, the true effect is likely to be substantially different from the estimated effect reported in this review.

### 6.4 Potential biases in the review process

Our review provides a comprehensive and robust analysis on the effectiveness of several community‐based interventions targeting individuals with lived experience of homelessness. This work, however, is not without certain inherent contextual and methodological limitations that challenged our efforts to draw generalisable and certain conclusions from our findings. First, the differences in how interventions were conceptualised and implemented in pragmatic conditions while falling under the same definition introduced a degree of clinical heterogeneity that prevented us from undertaking a more inclusive pooling of effect estimates, and therefore we resorted to narratively synthesising the majority of findings for this review. This, as well, was true for how usual care or “treatment as usual” conditions were defined and implemented, which affected the magnitude of effect estimates within a certain comparison of outcomes based on the intensity of usual care services relative to the intervention of interest. Second, the between‐study heterogeneity in the characteristics and levels of need of target populations has precluded us from generalising our findings to a wider population of individuals with lived experience of homelessness, as the majority of study participants had suffered from long term mental health or problematic substance use conditions prior to their enrolment into the study. This was apparent in our GRADE certainty of evidence assessments as most of the findings were of low to moderate certainty, indicating that prospective studies with a larger sample size that encompasses individuals with low levels of mental health or substance use needs will likely change the magnitude of effect estimates and certainty of evidence. Thirdly, the majority of studies took place in North American countries (i.e., The United States and Canada) and thus, the interpretation of effectiveness and cost‐effectiveness findings should be taken with caution in high income countries with contextual and global differences in health care and social welfare systems. Policy making strategies and decisions to implement a certain intervention should take these contextual differences into consideration. Finally, in our attempt to curate the most accurate evidence, we restricted our inclusion criteria to experimental controlled trials with quantitative outcome measurements. This condition may have provided higher internal validity to our findings and precision to our effect estimates, but it has also excluded findings from observational and qualitative studies which, in turn, could affect the external validity of our results.

### 6.5 Agreements and disagreements with other studies or reviews

#### 6.5.1 Permanent supportive housing

To our knowledge, our systematic review is the most up‐to‐date and comprehensive review available, examining a wide range of outcomes including housing stability, mental health, quality of life, substance use, hospitalisations, income and employment, as well as presenting practical cost‐effectiveness data for each intervention.

Our review presents results that are consistent with prior published research. A prior Campbell review (Munthe‐Kaas et al., 2018), published in February 2018, also reported an improvement in housing stability with nonabstinent contingent housing. Unlike our review, this publication included only RCTs and restricted inclusion to studies which had followed patients for at least a year. All RCTs included in this review had a high risk of bias, particularly due to poor randomisation or poor allocation concealment procedures, performance bias, detection bias, attrition bias or reporting bias (Munthe‐Kaas et al., 2018). Though RCTs are touted to be a gold‐standard means of evaluating the effectiveness of an intervention, the significant vulnerability of the homeless population may result in ethical dilemmas in withholding services, leading to some investigators pursuing alternative study designs. Therefore, to ensure that our findings are based on comprehensive evidence compatible with the pragmatism of housing interventions and transient nature of individuals with lived experience of homelessness, we elicited effectiveness data from a more broad range of clinical trials. Other more recently published reviews, Baxter et al. (2019) and Aubry et al. (2020) also found benefits in PSH strategies. Similar to our review, Baxter et al. (2019) demonstrated that intervention participants spent more days housed, were more likely to be housed at 18‐24 months, and had fewer emergency‐department visits and hospitalisations. Of note however, Baxter et al. (2019) examined only four RCTs, in order to enable a meta‐analysis synthesis of the data, and chose to encompass rental incentives and vouchers in their definition of PSH. Replication of findings with a higher quantity and broader range of publications provides further evidence of the validity and reproducibility of the intervention. Peng et al. (2020) found that HF programmes reduced homelessness by 88% when compared with Treatment First programmes, and 89% compared to TAU. Similar to our review, HF programmes did not significantly change mental health and substance use as compared with TAU, but was associated with less emergency department use and hospitalisations. Unlike our study, HF programmes were also found to improve clients' quality of life score, where‐as our review demonstrated mixed findings (Peng et al., 2020). Mixed data most likely reflects subjectivity and difficulty in measurement and quantification of quality of life. Further research is needed to explore the effect of HF interventions on quality of life. It is noteworthy to mention that Peng et al. (2020) did not seek to assess the certainty of evidence derived from their data and chose to exclude studies that did not meet a certain quality threshold set by the authors.

In contrast, Bassuk et al. 2014 did not find an effect of community affordable housing on residential stability. However, this review had significant limitations. Only six studies were identified and all except two were deemed to be methodologically “weak”. Most explored only baseline and follow‐up data without a control or comparison group, and were deemed by authors to have poor reporting quality. All six studies came from “grey literature” and mostly consisted of government and foundation commission studies (Bassuk et al. 2014).

Our review incorporates new research including 6‐year longitudinal‐outcome data and offers perspectives on a wider range of important psychological and social outcomes. Unlike many prior reviews, our synthesis also provides cost‐effectiveness data, which is an integral component to inform policy and decision‐making processes. Uniquely, our review incorporated into review processes persons with lived experience of homelessness, so as to promote engagement, health equity and community‐trust relations. Agreements with the vast majority of prior reviews offer policy‐makers a more robust evidence base to guide their decision making.

#### 6.5.2 Income assistance

Based on our latest literature review, the effect of income‐assistance interventions on health and social conditions for persons experiencing homelessness had not been systematically reviewed in the context of comprehensive social interventions prioritised for the homeless population. Aubry et al. (2020) reviewed the isolated effects of income‐assistance interventions for people experiencing homelessness and reported promising results in consistence with our findings. The studies which investigated the income supplement for low‐income families and pregnant women have also demonstrated positive outcomes on health‐related outcomes and healthcare utilisation along with the importance of longitudinal support, patient advocacy, and comprehensive services (Brownell et al., 2016; Forget, 2011).

#### 6.5.3 SCM and peer support interventions

This systematic review offers a comprehensive evaluation of the effectiveness and outcomes of SCM interventions. Two previous systematic reviews (De Vet 2013; Ponka et al., 2020) reported similar mixed results that suggest SCM might carry the potential to improve housing outcomes. however, to the best of our knowledge, no systematic review or meta‐analysis has examined the isolated effect of peer support interventions among individuals with lived experience of homelessness. One systematic review (Munthe‐Kaas et al., 2018) examined the effectiveness of peer support as an adjacent component to a more ICM programme and found no significant benefit in housing stability associated with that programme relative to usual care of less ICM.

#### 6.5.4 Mental health interventions

This systematic review offers a comprehensive evaluation of the effectiveness and outcomes of mental‐health interventions, including ICM, ACT and CTI. It incorporated more extensive definitions for specific responses and studies from other high‐income countries and regions, reporting evidence on managing mental health in homeless populations as a global public health problem. A recent systematic review (Ponka et al., 2020), and an earlier report (De Vet 2013), reported similar results on the effectiveness of mental health interventions that supported people with mental illness and substance as in this review. Our review confirmed significant positive findings on housing outcomes, particularly for ICM and ACT and, to a lesser degree CTI in improving housing access and stability. However, variations in the sub‐context of social and health determinants exist.

Morandi et al. (2017) and Stergiopoulos (2015a) reported reductions in psychiatric emergency visits, substance use levels, quality of life, social functioning, and engagements where ICM is integrated into other strength‐based approaches for at‐risk populations. Also, our findings are congruent with a meta‐analysis on ACT interventions, which reported more substantial benefits over SCM models in reducing the odds of homelessness and severity of symptoms in homeless persons with severe mental illness (Coldwell & Bender, 2007; Mueser, 1998; Vanderplasschen et al., 2007). Our findings on the benefits of ACT interventions agrees with the conclusions of a reductive effect on rates of relapse amongst youth undergoing opioid rehabilitation (Fishman et al., 2020). For CTI, our results were relatively consistent with an earlier review (De Vet, 2013), except for new evidence indicating that CTI decreased psychiatric symptoms. Another recent RCT reported the feasibility of CTI with mental health, employment or education, and better housing stability favouring a housing outreach programme—collaboration (HOP‐C) (Kidd et al., 2020). Nevertheless, there is still limited evidence, particularly in assessing the impact of mental health interventions (ACT and CTI) on income and employment outcomes in selected trials. In general, Ponka et al. (2020) highlighted the context of the intervention, notably where the intensity and role of case managers in the Netherlands differed in comparison to those in the United States or North America. Hence, our review highlights the potential impact of high fidelity and intensity of the intervention. ICM interventions are crucial in comprehensively addressing the needs of homeless or vulnerably housed persons. Our findings suggest adherence to this model of case management, which is continuous, community‐based and intensive, would maintain or increase the gains achieved.

## 7 AUTHORS' CONCLUSIONS

### 7.1 Implications for practice

Our review suggests that PSH programmes improve long‐term housing stability for up to 5.4 years compared to usual services. Income assistance, and high intensity case management interventions may also improve housing stability compared to usual services. The intensity and fidelity of the PSH and case management interventions strengthen the results. However, the interventions have mixed results for mental health, quality of life, substance use, hospitalisations, income and employment outcomes. The quality of the evidence varied from very low to moderate. The risk of bias including poor reporting, lack of blinding, and allocation bias reduced the certainty of the results. Given that most studies included individuals who had transitioned to living with chronic homelessness and often had a high burden of severe mental illness, it is possible that the overall impact of housing‐related interventions is in fact underestimated.

Preventing homelessness and providing early housing interventions has major practice and policy implications, particularly in 2021–2022 as the COVID‐19 pandemic disrupts economies, displaces populations, and threatens people already living with homelessness. Policymakers must reckon with a possible epidemic of complex social and public health challenges that may arise as more people transition to chronic homelessness. This review provides solid evidence on the value of PSH interventions and also suggests benefit of income and case management strategies. Policymakers will need imagination and innovation to scale up these interventions, including increasing their intensity and fidelity when appropriate. The absence of review evidence on substance‐use interventions for people living with homelessness represents an important research and policy gap.

This review has informed the development and publication of a clinical practice guideline, geared at preventing homelessness and improving the social and health outcomes of homeless and vulnerably housed individuals. The novel guideline outlines evidence‐based approaches and recommendations for initial priority steps. It seeks to reframe practice approaches in caring for patients with lived experience of homelessness, emphasising effective upstream interventions and promoting increased collaboration in the delivery of care (Pottie et al., 2020).

Indeed, preventing homelessness with responsive housing programmes has major cost and policy implications. Further inter‐sectoral collaboration will be required by health care providers, community health organisations, public health officials and policy makers to facilitate increased coordination of services, improve accessibility and address resource fragmentation. Evidence‐based reviews like this one can guide practice and outcome research and contribute to advancing international perspectives on the effectiveness of interventions to prevent and end homelessness.

### 7.2 Implications for research

PSH interventions have emerged as effective core elements in the care of homeless and vulnerably housed populations. However, there is still a need for more pragmatic research to understand inconsistent and mixed results on mental health, quality of life, substance use, hospitalisations, income and employment outcomes. As well, there are also ongoing gaps that warrant more research, including exploring peer support programmes, SCFs and other community substance use programmes for homeless populations. Further research should also study the intersectional and differential effect of certain interventions on specific populations, including Indigenous and migrant groups, recognising the heterogeneity of the homelessness population. Factors such as age, demographics, social identities, comorbidities and pathways into homelessness are all important considerations in identifying the benefit and harm of interventions. Health equity research may offer useful interdisciplinary methods and approaches to improve outcomes for sub‐groups within the overall population of persons with homelessness.

There is also a role for expanding implementation research for community interventions. Studies report significant resource and logistical barriers implementing programmes to meet the complex social and health needs of this vulnerable population. Persons with lived experience of chronic homelessness may harbour considerable retrospective bias, earned from years of experience in and out of housing shelters, which may deter trust and slow the implementation of new interventions. Research will be needed to help guide practice, public health and policy makers in actualisation of evidence‐based recommendations. Our review did not seek to understand the mechanisms behind why certain services may hold benefit over others. By improving mechanistic knowledge, future realist reviews may be able to guide the development of multisectoral and targeted interventions to better affect downstream consequences of homelessness with respect to mental health, quality of life, morbidity and mortality.

## CONTRIBUTIONS OF AUTHORS

- Content: Aliza Moledina (Permanent Supportive Housing and Critical Time Intervention); Jui‐Hsia Hung (Income‐Assistance Intervention); Ammar Saad (Peer Support), Eric Agbata (Standard Case Management, Intensive Case Management, Assertive Community Treatment), Olivia Magwood (Supervised consumption facilities, Managed alcohol programmes, opioid agonist therapy), Kednapa Thavorn (Cost‐effectiveness)
- Systematic review methods: Ammar Saad, Olivia Magwood, Kevin Pottie
- Statistical analysis: Ammar Saad
- Information retrieval: Ammar Saad, Olivia Magwood

## DECLARATIONS OF INTEREST

None declared

### PRELIMINARY TIMEFRAME

August 25, 2020

(Approximate date for submission of the systematic review.)

### PLANS FOR UPDATING THIS REVIEW

We will update our search prior to submitting this review for publication to ensure that our findings are up‐to‐date. As well, the reviews will be updated using the PICO question in 4 years. Kevin Pottie will be responsible for updates.

## DIFFERENCES BETWEEN PROTOCOL AND REVIEW

Following the publication of our protocol (Pottie et al., 2019), we engaged key stakeholder groups, such as individuals with lived experience of homelessness, in the review development process. We conducted consultations with these stakeholders as well as experts in the field of homeless health and health equity, and decided to prioritise housing stability as a primary outcome of our review to better highlight the effectiveness of permanent supportive housing on its primary intended purpose of mitigating homelessness in its conceptual definition; increasing housing stability and decreasing days on the street or in shelters. Other health and social outcomes such as mental health, quality of life, substance use, hospitalisation and health care utilisation, employment, and income were considered to be secondary outcomes of this review. As such, our assessment of evidence certainty using the GRADE methodology (Please see Methods) was exclusive to the primary outcome of interest as our primary conclusions from this review pertain to housing stability.

## PUBLISHED NOTES

### Characteristics of studies

#### Characteristics of included studies

Aubry et al. (2016)

**Table:** 

**Methods**	**Designs**: Cost analysis and randomised controlled trial
	**Setting**: Toronto, Montreal, Moncton, Winnipeg, Vancouver, Canada
	**Follow up**: up to 24 months
	**Recruitment**: Referred by health and social service agencies
	**Randomization**: Participants were randomized by a central data collection system that used an adaptive randomization algorithm
	**Allocation**: 1:1 allocation ratio
	**Blinding**: Not possible
	**Timing of outcome assessment**: At baseline and every six months for 21 or 24 months
	**Outcome assessor**: The investigators conducted in person interviews
**Participants**	**Population**: Homeless individuals or individuals living in a single room occupancy, rooming house, or hotel who have had two or more episodes of being absolutely homeless with serious mental illness and high support needs.
	**Sample size**: Total n = 950, Intervention n = 469, TAU n = 481
**Interventions**	**Intervention**:
	Housing First provides immediate access to independent housing and mental health supports; rent supplements were provided that ensured housing costs did not exceed 30% of participant's income; housing coordinators provided assistance to find and move into housing; support services were provided via assertive community treatment; study participants agreed to observe the terms of their lease and be available for a weekly visit by programme staff.
	**Comparator**:
	Treatment as usual (TAU): Participants had access to the already existing interventions and programs available in their community including any housing and community support other than the Housing First program
**Outcomes**	Housing stability, mental health, quality of life, substance use, hospital admission, employment, income
	**Cost or cost‐effectiveness**: The Housing First programme led to a reduction in the mean cost to $21,367 per person per year; this cost offset was associated with office visits, hospital admissions, emergency shelter visits, home visits, and incarceration; the savings gained by Housing First did not fully offset its cost.
**Notes**	

Risk of bias table

**Table:** 

Bias	Authors' judgement	Support for judgement
Random sequence generation (selection bias)	Low risk	Randomization was performed via adaptive randomization procedures. Adaptive randomization can ensure better balance between groups in small and moderate sized studies than strict randomization, by continually adjusting the probability of assignment to either group, depending on existing group assignment.
Allocation concealment (selection bias)	Low risk	The allocation algorithm was concealed from both the participants and the research staff.
Blinding of participants and personnel (performance bias)	High risk	Blinding of participants and personnel was not possible due to the nature of the intervention and study design.
Blinding of outcome assessment (detection bias)	High risk	Several aspects of the study prohibited blinding, including the nature of the administered questionnaires (detailed housing history and service use), location of participant interviews (some participants elected to be interviewed at their place of residence)
Incomplete outcome data (attrition bias)	Low risk	No evidence of incomplete outcome data provided
Selective reporting (reporting bias)	Low risk	No evidence of reporting outcomes selectively is provided
Other bias	Low risk	No other potential risk of biases

Booshehri (2017)

**Table:** 

**Methods**	**Design**: 3‐arm randomized control trial
	**Setting**: Philadelphia, PA, USA
	**Follow up**: 15 months
	**Recruitment**: Participants were recruited by research staff at three county assistance offices
	**Randomization**: Single‐blind randomization was performed by assigning participants to 150 random numbers previously assigned.
	**Allocation**: Not reported
	**Blinding**: Single blinding was used so as program facilitators and instructors were instructed not to discuss allocation
	**Timing of outcome assessment**: At baseline, and 9,12, and 15 months
	**Outcome assessor**: Self‐administered survey
**Participants**	**Population**: Low‐income families with a child; 65% of whom had housing insecurity
	**Sample size**: Total n = 103, Partial intervention n = 35, Full intervention n = 37, Control n = 31
**Interventions**	**Intervention**:
	Partial intervention included assistance opening a credit union savings account, with participants' savings matched throughout trial and 28 weeks of 3 h financial empowerment education classes, which focused on developing internal and external resources to support self‐sufficiency; education included saving for education, retirement, housing, entrepreneurial activities, improving credit and reducing debt; the full intervention was the same as the partial intervention plus a simultaneous 28 weeks of trauma‐informed peer support.
	**Comparator**:
	Control group: standard TANF programming consisting of 20 h per week of scheduled supervised job training and job search activities.
**Outcomes**	Housing stability, mental health, employment, income
**Notes**	

Risk of bias table

**Table:** 

Bias	Authors' judgement	Support for judgement
Random sequence generation (selection bias)	Low risk	Through single‐blind randomization participants were assigned into each group.
Allocation concealment (selection bias)	Unclear risk	No description of allocation concealment was provided.
Blinding of participants and personnel (performance bias)	High risk	Blinding of participants and personnel was not possible due to the nature of the intervention and study design.
Blinding of outcome assessment (detection bias)	High risk	“All data are self‐reported, and therefore may be prone to bias, where people may minimize symptoms or other concerns and exaggerate income and employment experiences.
Incomplete outcome data (attrition bias)	Low risk	No evidence of incomplete outcome reporting or attrition bias was detected.
Selective reporting (reporting bias)	Low risk	No evidence of reporting outcomes selectively was reported.
Other bias	Unclear risk	Class participation rates were limited and potentially hindered our ability to assess full treatment effects.

Braucht (1995)

**Table:** 

**Methods**	**Design**: Randomized control trial
	**Setting**: Denver, Colorado, United States
	**Follow up**: 10 months
**Participants**	**Population**: Homeless individuals 18 years or older with alcohol or other substance abuse problems
	**Sample size**: Total sample n = 323, Intervention n = 163, control n = 160
**Interventions**	**Intervention**:
	Intensive Case Management (ICM) with access to services available at Arapahoe House (detoxification facility). ICM included proactive outreach, client identification and assessment, development of an individually‐tailored and comprehensive service plan for each client, establishment of linkages between service systems and clients such that services were matched to client needs, continuity of service utilization monitoring, and assertive advocacy with community agencies on behalf of clients. Case managers worked in pairs (dyads) with a caseload averaging 15 clients per dyad (and never exceeding 17 clients per dyad) and with a consequent high intensity of client contact.
	**Comparator**:
	Access to the full range of services offered by Arapahoe House. Services include a comprehensive array of substance abuse treatment and rehabilitation services, including detoxification, residential and outpatient services, substance abuse counseling, literacy and vocational assessment, and job training and placement.
**Outcomes**	Housing stability, Mental health, Quality of life, Substance use, Employment
**Notes**	

Risk of bias table

**Table:** 

Bias	Authors' judgement	Support for judgement
Random sequence generation (selection bias)	Low risk	The random assignment of clients to the experimental (ICM) and control groups in the present study was implemented with a computer program specifically designed to protect the integrity of the assignment process
Allocation concealment (selection bias)	Unclear risk	No description of allocation concealment provided
Blinding of participants and personnel (performance bias)	High risk	Blinding of participants and personnel was not possible due to the nature of the intervention and study design.
Blinding of outcome assessment (detection bias)	Unclear risk	No description of blinding of outcome assessment provided.
Incomplete outcome data (attrition bias)	Low risk	There was no evidence that differential attrition introduced meaningful nonequivalence between the groups.
Selective reporting (reporting bias)	Low risk	No evidence that outcomes were selectively reported.
Other bias	Low risk	No other biases identified.

Burnam (1995)

**Table:** 

**Methods**	**Design**: 3‐arm Randomized control trial
	**Setting**: The Westside area, Los Angeles county, United States
	**Follow up**: 9 months
**Participants**	**Population**: Homeless adults with both a serious mental illness and substance dependence
	**Sample size**: Total sample size n=276, Experimental arm1 n = 144, Experimental arm2 n = 67, Control n = 65
**Interventions**	**Experimental arm 1**: A social model residential program providing integrated mental health and substance abuse treatment
	**Experimental arm 2**: A community‐based nonresidential program using (1) curriculum‐based groups focused on substance abuse and mental health education and rehabilitation; (2) 12‐step programs including participation in community‐based AA or NA meetings (3) discussion of issues of importance to the clients (4) individual counseling and case‐management (5) psychiatric consultation and ongoing medications management (6) general community activities
	**Comparator**: Participants randomized to the control group did not receive interventions but were allowed free access to community services.
**Outcomes**	Housing stability, mental health, substance use
**Notes**	

Risk of bias table

**Table:** 

Bias	Authors' judgement	Support for judgement
Random sequence generation (selection bias)	Unclear risk	Probability of assignment to the nonresidential group was set at twice that of the other groups. Random assignment was made within two blocking variables, gender and primary type of mental disorder.
Allocation concealment (selection bias)	Unclear risk	Allocation concealment not reported
Blinding of participants and personnel (performance bias)	High risk	Participants and personnel could not be blinded to the intervention.
Blinding of outcome assessment (detection bias)	Unclear risk	Blinding of outcome assessment not reported.
Incomplete outcome data (attrition bias)	High risk	Rates of completed follow‐up were 79% for the 3‐month follow‐up, 76% for the 6‐month follow up, 70% for the 9‐month follow‐up, and 58% completed all three follow ups. At the 9‐month follow‐up the completion rate among those assigned to the control group (57%) was significantly lower than among those assigned to nonresidential treatment (76%).
Selective reporting (reporting bias)	Low risk	No evidence of selective outcome reporting.
Other bias	Low risk	None Identified.

Cauce (1994)

**Table:** 

**Methods**	**Design**: Randomized control trial
	**Setting**: Seattle, Washington, United States
	**Follow up**: 3 months
**Participants**	**Population**: Homeless adolescents 13‐21 years old
	**Sample size**: Total sample size n = 115, Intervention n = 55, control n = 60
**Interventions**	**Intervention**:
	Intensive case management: Development of treatment plans, linkage to appropriate services, monitor and track clients, advocating for basic entitlements, 24 h crisis service. Maximum caseload of 12
	**Comparator**:
	Those randomized to the control group received regular case management with caseload 18‐30
**Outcomes**	Mental health, quality of life, substance use
**Notes**	

Risk of bias table

**Table:** 

Bias	Authors' judgement	Support for judgement
Random sequence generation (selection bias)	Low risk	“Random assignment was accomplished by preparing a stack of sequentially numbered envelopes, and placing in each a card with a matching number and group assignment”.
Allocation concealment (selection bias)	Unclear risk	No description of allocation concealment was provided.
Blinding of participants and personnel (performance bias)	High risk	Blinding of participants and personnel was not possible due to the nature of the intervention and study design.
Blinding of outcome assessment (detection bias)	Unclear risk	No description of blinding of outcome assessment was provided.
Incomplete outcome data (attrition bias)	Unclear risk	Results are based on the data collected from the first 115 adolescents who completed the first quarterly follow‐up assessment of the 229 total recruited ‐ no significant difference from the overall pool of participants on any of the measures of psychological and social adjustment previously described (p<.10). Also, the exact number in each analysis varies from 104 to 115 due to missing data on some measures, there's no further explanation.
Selective reporting (reporting bias)	Low risk	No evidence of reporting outcomes selectively was detected.
Other bias	Low risk	No other potential biases were detected.

Chalmers 2010

**Table:** 

**Methods**	**Design**: A cost analysis based on administrative databases (before‐ and‐after study)
**Participants**	**Population**: Homeless people with mental illness who were formerly homeless in the state of Maine (USA)
**Interventions**	**Intervention**:
	Before and after providing permanent supported housing
	**Comparator**:
	N/A
**Outcomes**	**Cost or cost‐effectiveness**: Supportive housing was associated with a 57% reduction in mental health care costs, a 97% reduction in emergency shelter costs, a 14% reduction in jail costs, and 32% savings in ambulance costs; total savings to the system was US$584,907 after 12 months in housing, representing a mean saving of $2,182 per participant in the study
**Notes**	

Risk of bias table

**Table:** 

Bias	Authors' judgement	Support for judgement
Random sequence generation (selection bias)	Unclear risk	
Allocation concealment (selection bias)	Unclear risk	
Blinding of participants and personnel (performance bias)	Unclear risk	
Blinding of outcome assessment (detection bias)	Unclear risk	
Incomplete outcome data (attrition bias)	Unclear risk	
Selective reporting (reporting bias)	Unclear risk	
Other bias	Unclear risk	

Cherner et al., 2017

**Table:** 

**Methods**	**Design**: Quasi‐experimental trial
	**Setting**: Ottawa, Canada
	**Follow up**: 24 months
	**Recruitment**: Invitation from participants' case manager
	**Randomization**: No randomization was performed
	**Allocation**: Not mentioned
	**Blinding**: Not mentioned
	**Timing of outcome assessment**: At baseline and every 6 months for 24 months
	**Outcome assessor**: Not mentioned
**Participants**	**Population**: Homeless individuals or people at risk of homelessness with problematic substance use
	**Sample size**: Total n=178, Intervention n=89, TAU n=89
**Interventions**	**Intervention**:
	A partnership between a community mental health agency and a programme located in a community health centre; each participant received a rent supplement and paid a maximum of 30% of their income towards rent; housing comprised private market rental units of participant's choice; participants also had access to opioid agonist treatment and substance use treatment.
	**Comparator**:
	Treatment as usual (TAU) including all social and health services available in the community other than the Housing First program. The services were scattered across a service rich city and included supportive housing, mental health, and substance use services available to people who are homeless as well as services that can be accessed while people are in a shelter.
**Outcomes**	Housing stability, mental health, quality of life, substance use
**Notes**	

Risk of bias table

**Table:** 

Bias	Authors' judgement	Support for judgement
Random sequence generation (selection bias)	High risk	Description on random sequence generation was not provided.
Allocation concealment (selection bias)	High risk	Selection bias: Recruitment for the comparison group occurred after all Housing First clients participating had been admitted to the program. Since the program selected adults with the greatest health and substance needs, this sampling strategy may have resulted in Housing First clients having more needs and more severe health and substance use problems than the comparison participants.
Blinding of participants and personnel (performance bias)	High risk	Blinding of participants and personnel was not possible due to the nature of the intervention and study design.
Blinding of outcome assessment (detection bias)	Unclear risk	No description of blinding of outcome assessment was provided
Incomplete outcome data (attrition bias)	Low risk	No evidence of incomplete outcome data.
Selective reporting (reporting bias)	Low risk	No evidence that outcomes were reported selectively.
Other bias	Low risk	No other potential biases detected.

Clark et al., 1998

**Table:** 

**Methods**	**Design**: Cost‐effectiveness analysis conducted alongside an RCT, societal perspective. Costs were reported in 1995 US$
**Participants**	**Population**: Persons with schizophrenia, schizoaffective disorder, or bipolar disorder and a concurrent substance use disorder
**Interventions**	**Intervention**:
	Specialized treatment for dual disorders in an ACT team
	**Comparator**:
	SCM program with targeted substance abuse services
**Outcomes**	**Cost/Cost‐effectiveness**:
	ACT more effective and less costly than SCM in both substance abuse and quality of life comparisons.
	The relationship between the treatment received and benefit to cost ratio was curvilinear. During earlier periods SCM produced better outcomes per $10,000 invested than did ACT. During the final year of the study, ACT produced substantially better outcomes per $10,000 than SCM.
**Notes**	

Risk of bias table

**Table:** 

Bias	Authors' judgement	Support for judgement
Random sequence generation (selection bias)	Unclear risk	
Allocation concealment (selection bias)	Unclear risk	
Blinding of participants and personnel (performance bias)	Unclear risk	
Blinding of outcome assessment (detection bias)	Unclear risk	
Incomplete outcome data (attrition bias)	Unclear risk	
Selective reporting (reporting bias)	Unclear risk	
Other bias	Unclear risk	

Clarke et al., 2000

**Table:** 

**Methods**	**Design**: 3‐arm randomized control trial
	**Setting**: Portland, Oregon, United States
	**Follow up**: 24 months
**Participants**	**Population**: Clients with serious and persistent mental illness who were at risk of being homeless
	**Sample size**: Total sample n = 163, Consumer ACT n = 57, Non‐consumer ACT n = 57, Usual care n = 49
**Interventions**	**Intervention**:
	The ACT programs: Each team consisted of four full‐time and one part‐time case managers, one of whom was the team leader. Both teams shared a psychiatrist, a nurse practitioner, and a clinical director. The psychiatrist conducted initial psychiatric assessments, prescribed and monitored medication, and participated in treatment planning. The clinical director provided consultation to the two teams and handled administrative tasks. The **Consumer ACT** staff held a bachelor's degree, had more average experience in the mental health field, had more mental disorders themselves than the non‐consumer team. The average caseload for the ACT team during the study period was 4.6 clients per case manager. **Non‐consumer ACT**: Staff reported no diagnosable mental illness. The majority of the non‐consumer‐ACT staff held a master's degree. The average caseload for the ACT team during the study period was 5.4 clients per case manager.
	**Comparator**:
	The "Usual Care" control condition:
	Most subjects assigned to usual care were served by one of four community mental health centers (CMHCs) and a number of smaller, more specialized agencies in the Portland metro area usual care agencies set up special, intensive, community‐based programs to better serve these high‐need clients in the community. The intensity of services in these programs rivaled the intensity of the ACT teams. Average caseload size for usual care overall was 26.9
**Outcomes**	Hospitalization
**Notes**	

Risk of bias table

**Table:** 

Bias	Authors' judgement	Support for judgement
Random sequence generation (selection bias)	Unclear risk	No description of random sequence generation was provided.
Allocation concealment (selection bias)	Unclear risk	No description of allocation concealment was provided.
Blinding of participants and personnel (performance bias)	High risk	Blinding of participants and personnel was not possible due to the nature of the intervention and study design.
Blinding of outcome assessment (detection bias)	Unclear risk	No description of blinding of outcome assessment was provided.
Incomplete outcome data (attrition bias)	Unclear risk	No description of reporting outcome data incompletely or attrition bias was provided.
Selective reporting (reporting bias)	Low risk	No evidence of reporting outcomes selectively was detected.
Other bias	Low risk	**No other potential biases detected**.

Clark & Rich, 2003

**Table:** 

**Methods**	**Design**: Quasi experimental trial
	**Setting**: Pinellas county, Florida, United States
	**Follow up**: 12 months
**Participants**	**Population**: Homeless adults with mental illness
	**Sample size**: The total sample size n = 152, Comprehensive housing program n = 83, Case management n = 69
**Interventions**	**Intervention**:
	Comprehensive housing programs: Developed a program specifically to prevent and reduce homelessness in this population. The program features guaranteed access to housing and housing support services, case management, and priority access to everything from medication management to vocational services. Project Return in Tampa, Florida, also provides comprehensive housing services to homeless persons with severe mental illness, including guaranteed access to housing, housing support services, and case management.
	**Comparator**:
	Case management only: developed a homeless outreach and support team (HOST) to provide short‐term case management services for homeless individuals with severe mental illness. The activities of this blended case management program (11) include active outreach and engagement, some on site counseling, medication and medication management, assistance with obtaining housing, and linkages to other psychosocial services.
**Outcomes**	Housing stability, mental health
**Notes**	

Risk of bias table

**Table:** 

Bias	Authors' judgement	Support for judgement
Random sequence generation (selection bias)	High risk	This study was a quasi experimental trial with non‐random assignment design.
Allocation concealment (selection bias)	High risk	This study was a quasi experimental trial with non‐random assignment design.
Blinding of participants and personnel (performance bias)	High risk	Blinding of participants and personnel was not possible due to the nature of the intervention and study design.
Blinding of outcome assessment (detection bias)	Unclear risk	No description of blinding of outcome assessment was provided.
Incomplete outcome data (attrition bias)	Unclear risk	The attrition rate in the group receiving only case management was significant and was probably due to the time‐limited nature of these services.
Selective reporting (reporting bias)	Low risk	No evidence of reporting outcomes selectively.
Other bias	Low risk	No other potential biases detected.

Conrad et al., 1998

**Table:** 

**Methods**	**Design**: Randomized control trial
	**Setting**: Illinois, United States
	**Follow up**: 24 months
**Participants**	**Population**: Homeless addicted male veterans
	**Sample size**: Total sample n=358, Intervention n = 178, Control n = 180
**Interventions**	**Intervention**:
	3‐6 months of stay at hospital facility (residential care) plus case management. The ratio of residents to case managers was 10:1 in the residency phase and approximately 25:1 in the community follow‐up phase. Relapse prevention skills training, consisting of assertive drink and drug refusal, coping with relapse, social networking, and anger management, was an essential component of CMRC treatment. Self‐help groups such as Narcotics Anonymous (NA) and Alcoholics Anonymous (AA), both on‐site and community‐based, were emphasized for emotional support and to enhance coping behavior while achieving abstinence
	**Control**:
	21 days in customary care: During the hospital stay in customary care, patients received substance abuse and abstinence education and individual and group therapy. Patients were seen by a social worker for assessment, psychotherapy, and discharge planning.
**Outcomes**	Housing stability, Substance use, Employment
**Notes**	

Risk of bias table

**Table:** 

Bias	Authors' judgement	Support for judgement
Random sequence generation (selection bias)	Unclear risk	Participants were randomized to treatment condition. However, no description of random sequence generation was provided.
Allocation concealment (selection bias)	Low risk	“The 358 subjects were measured at the pretest on the variables on interest before random assignment and before receiving the experimental or control intervention. This procedure ensured that neither the investigators nor the subjects would know who was to be assigned to the experimental or control group after the pre‐test”.
Blinding of participants and personnel (performance bias)	High risk	Blinding of participants and personnel was not possible due to the nature of the intervention and study design.
Blinding of outcome assessment (detection bias)	Unclear risk	No description of blinding of outcome assessment was provided.
Incomplete outcome data (attrition bias)	Low risk	“… we concluded that differential attrition was not a significant problem in this study”.
Selective reporting (reporting bias)	Low risk	No evidence of reporting outcome selectively was detected.
Other bias	Low risk	No other potential biases were detected.

Corrigan et al., 2017

**Table:** 

**Methods**	**Design**: Randomized controlled trial
	**Recruitment**: by the CBPR team members' wide dissemination of flyers
	**Setting**: Clinics and homeless shelters in Metropolitan Chicago
	Follow‐up: 4, 8, and 12 months
**Participants**	**Population**: Homeless African Americans with serious mental illness
	**Eligibility criteria**: People had to self‐identify as African American and report being currently homeless. People also had to self‐report whether they currently were challenged by mental illness and their current diagnosis. Excluded were those who reported no mental illness, or who were receiving case management somewhere else.
	**Sample size**: Total n = 67, Intervention n = 33, control n = 33
	**Baseline characteristics**: “Overall, 39% (N = 26) of research participants were female, and the sample had a mean (SD) age of 52.9(68.0). The group was 87% (N=58) heterosexual and somewhat varied in education, with 64% (N = 43) having a high school diploma or less. Thirteen percent (N = 10) reported having some kind of employment. The two groups did not differ significantly on any demographic characteristics nor on primary diagnosis” (Corrigan et al., 2017)
**Interventions**	**Peer Navigator Program (PNP)**: The peer navigators conducted face‐to‐face meetings with service recipients in places and at times that were convenient to the recipient. Goals of the meetings were to review all health concerns and actions to address these concerns. Goals and actions could include activity related to alleviating homelessness, improving diet, and reducing criminal justice involvement because each of these factors will influence health. PNs were expected to contact participants at least once a week. However, frequency was as high as five times a week, depending on participants' needs. All three PNs were African Americans who were homeless during their adult life and in recovery from serious mental illness.
	**Treatment as usual**: usual care which may have included services provided by the Together for Health system (T4H), a coordinated care entity funded by the Illinois Medicaid Authority to engage and manage care for individuals with multiple chronic illnesses. T4H was a network of more than 30 mental and other health care programs in Chicago to provide integrated care to people with serious mental illness. One of the goals of T4H (and for the PNP, for that matter) was to engage and enroll people with disabilities into its network.
**Outcomes**	**Housing stability**: Frequency of homelessness
	**Mental health**: Frequency of experiencing mental health problems in the past 30 days using the Texas Christian University Health Form (TCU‐HF), and emotional wellbeing using the Short Form‐36 (SF‐36)
	**Quality of life**: Generic quality of life was assessed with Lehman's Quality of Life Scale (QLS).
**Notes**	

Risk of bias table

**Table:** 

Bias	Authors' judgement	Support for judgement
Random sequence generation (selection bias)	Unclear risk	The investigators mentioned randomization but did not report on the random sequence generation
Allocation concealment (selection bias)	Unclear risk	No reporting was made on allocation concealment
Blinding of participants and personnel (performance bias)	High risk	Blinding of participants and personnel was not possible due to the nature of the intervention and study design
Blinding of outcome assessment (detection bias)	Unclear risk	No reporting was made on blinding of outcome assessors
Incomplete outcome data (attrition bias)	Low risk	Seven participants were lost to follow‐up, with two of these participants dying during the course of the project and three being incarcerated
		Reason for dropping out were not related to the nature of the outcome
Selective reporting (reporting bias)	Low risk	No evidence of selective outcome reporting
Other bias	Low risk	No evidence of other concerns for bias

Cox et al., 1998

**Table:** 

**Methods**	**Design**: Randomized control trial
	**Setting**: Seattle, Washington, United States
	**Follow up**: 2 years
**Participants**	**Population**: Homeless chronic inebriate clients
	**Sample size**: Total sample n = 289, ICM n = 150, usual care n = 148
**Interventions**	**Intervention**:
	Intensive Case management: long‐term, open‐ended, outreach‐oriented service focused primarily on system advocacy and linkage activities. Retention was regarded as more important than compliance, so provision of the service was not conditional on client behaviour and there was no requirement that clients maintain sobriety in order to continue in the program. Caseloads averaged 15 clients per case manager
	**Comparator**:
	Those randomized to the control group received standard treatment
**Outcomes**	Housing stability, Substance use, Employment, Income
**Notes**	

Risk of bias table

**Table:** 

Bias	Authors' judgement	Support for judgement
Random sequence generation (selection bias)	Unclear risk	No description of random sequence generation was provided.
Allocation concealment (selection bias)	Unclear risk	No description of allocation concealment was provided.
Blinding of participants and personnel (performance bias)	High risk	Blinding of participants and personnel was not possible due to the nature of the intervention and study design.
Blinding of outcome assessment (detection bias)	Unclear risk	No description of blinding of outcome assessment.
Incomplete outcome data (attrition bias)	High risk	Follow‐up rate was 68% for the 24‐months follow‐up interview, this was low because the follow‐up effort was truncated by the end of funding for the project.
Selective reporting (reporting bias)	Low risk	No evidence of reporting outcomes selectively was detected.
Other bias	Low risk	No other potential biases were detected.

Culhane et al., 2002

**Table:** 

**Methods**	**Design**: Cost analysis, based on administrative databases maintained by eight agencies
**Participants**	**Population**: Homeless people who received services or support from eight agencies in New York
**Interventions**	**Intervention**:
	New York‐New York housing placement
	**Comparator**:
	No supportive housing placement
**Outcomes**	**Cost or cost‐effectiveness**: The total mean cost of service utilization for the New York‐New York placement period was US$40,451 per placement per year; the annualised cost per placement was $13,570; NY/NY housing was associated with a $12,146 net reduction in health, corrections, and shelter service use annually per person over the first 2 years of the intervention; a New York‐New York placement had a net additional cost of $1,425 per placement per year.
**Notes**	

Risk of bias table

**Table:** 

Bias	Authors' judgement	Support for judgement
Random sequence generation (selection bias)	Unclear risk	
Allocation concealment (selection bias)	Unclear risk	
Blinding of participants and personnel (performance bias)	Unclear risk	
Blinding of outcome assessment (detection bias)	Unclear risk	
Incomplete outcome data (attrition bias)	Unclear risk	
Selective reporting (reporting bias)	Unclear risk	
Other bias	Unclear risk	

De Vet 2017

**Table:** 

**Methods**	**Design**: Randomized control trial
	**Setting**: Netherlands
	**Follow up**: up to 9 months
**Participants**	**Population**: Homeless adults who had resided in a shelter for less than 14 months and were moving to housing
	**Sample size**: Total sample n = 183, Intervention n = 94, control n = 89
**Interventions**	**Intervention**:
	Critical Time Intervention (CTI). Time‐limited, strength‐based intervention for vulnerable people, which bridges the gap between services during times of transition, generally consisted of discharge planning and referral services and access to a range of community‐based services. The CTI worker provides practical and emotional support and helps to develop and strengthen links with community resources, creating a network that will continue to provide support long after CTI has ended. Delivered in three phases of 3 months: transition to the community (phase 1), try‐out (phase 2), and transfer of care (phase 3)
	**Comparator**:
	Care‐as‐usual
**Outcomes**	Housing stability, Mental health, Quality of life, Substance use
**Notes**	

Risk of bias table

**Table:** 

Bias	Authors' judgement	Support for judgement
Random sequence generation (selection bias)	Low risk	Randomization was stratified by shelter and balanced within blocks of four. A list of random numbers was computer generated by a member of the research team and concealed in a secure digital file until assignment.
Allocation concealment (selection bias)	Low risk	Clients, shelter staff, and this research assistant did not have any foreknowledge of condition assignment.
Blinding of participants and personnel (performance bias)	High risk	Clients remained blind until they met their CTI worker or case manager for the first time. However, both participants and personnel were aware of allocation once CTI intervention was initiated.
Blinding of outcome assessment (detection bias)	High risk	Condition assignment was withheld from data collectors. However, some of them did become aware of the allocated condition during follow up, because participants would sometimes spontaneously disclose this information during an interview.
Incomplete outcome data (attrition bias)	Low risk	Low attrition in study (5%)
Selective reporting (reporting bias)	Low risk	Outcomes pre‐specified in published protocol.
Other bias	Low risk	Bias limited by use of standardized instruments.

Dickey et al., 1997

**Table:** 

**Methods**	**Design**: Cost analysis based on a prospective experimental design from state or local government
**Participants**	**Population**: Individuals with a current diagnosis of severe mental illness
**Interventions**	**Intervention**:
	Evolving consumer household
	**Comparator**:
	Independent apartment living
**Outcomes**	**Cost or cost‐effectiveness**: Individuals assigned to evolving consumer households had mean annual housing expenditures of $42,829 compared with $13,042 for individuals assigned to independent living apartments; difference in annual mean treatment costs of individuals in the ECH group ($11,293) and the independent apartment living group ($14,541) were not statistically different; the mean annual cost per person the evolving consumer households group was statistically higher than those in independent living apartments ($56,434 vs $29,838).
**Notes**	

Risk of bias table

**Table:** 

Bias	Authors' judgement	Support for judgement
Random sequence generation (selection bias)	Unclear risk	
Allocation concealment (selection bias)	Unclear risk	
Blinding of participants and personnel (performance bias)	Unclear risk	
Blinding of outcome assessment (detection bias)	Unclear risk	
Incomplete outcome data (attrition bias)	Unclear risk	
Selective reporting (reporting bias)	Unclear risk	
Other bias	Unclear risk	

Ellison et al., 2020

**Table:** 

**Methods**	**Design**: Randomized controlled trial
	**Recruitment**: Potential participants were identified by the housing program case managers or by medical records.
	**Setting**: 2 Veterans Affairs medical centers, blinded city, US
	**Follow‐up**: At baseline, midpoint (about 4–6 mo), and endpoint (about 9–12 mo)
**Participants**	**Population**: Formerly homeless Veterans housed using housing vouchers (HUD‐VASH)
	**Eligibility criteria**: having a history of abusing alcohol and/or drugs, having a serious mental illness, and having a history of homelessness
	**Sample size**: Total n = 166, Intervention n = 85, control n = 81
	**Baseline characteristics**: “Participants were older mostly men with nearly equal proportions of white and African American Veterans. Modal education status was high school graduate and most were unemployed and unmarried. 40.1% of Veteran participants had been homeless 2–4 times, for 1–3 years (32.7%). There were no statistically significant differences for any baseline characteristics between treated and control group Veterans” ‐Ellison et al., 2020
**Interventions**	**Peer specialist services**: Alongside the HUD‐VASH housing vouchers, the peer specialist‐Veteran meetings were designed to focus on mental health and substance use recovery and community integration skills delivered in both 20 “structured” sessions and 20 “unstructured” meetings designed for community engagement and relationship building. The peer specialists were intended to meet with Veterans at their home or in the community for 1 h each week for 40 sessions to occur over the course of 9–12 months. The peer specialists had to be Veterans with significant recovery from mental health issues, which could include substance abuse. Seven male peer specialists were hired over the course of the study, 6 were white and 1 was African American. It was not possible to provide peer specialists matched to sex and racial profiles of the Veterans.
	**Treatment as usual**: Included the HUD‐VASH housing vouchers, as well as usual care provided by the HUD‐VASH program.
**Outcomes**	**Housing stability**: Housing stability was measured with a count of days spent in HUD‐VASH housing within the past 30 days and with a count of days housed that included HUD‐VASH housing and other types of community housing (such as with a friend or parents).
	**Mental health**: Symptoms of mental illness were identified using the 24‐item Behavior And Symptom Identification Scale (BASIS‐24).
	**Substance use**: alcohol and drug use were identified using questions from the Addiction Severity Index (ASI).
**Notes**	

Risk of bias table

**Table:** 

Bias	Authors' judgement	Support for judgement
Random sequence generation (selection bias)	Unclear risk	The investigators mentioned randomization but did not report on the random sequence generation
Allocation concealment (selection bias)	Unclear risk	No reporting was made on allocation concealment
Blinding of participants and personnel (performance bias)	High risk	Blinding of participants and personnel was not possible due to the nature of the intervention and study design.
Blinding of outcome assessment (detection bias)	Unclear risk	No reporting was made on blinding of outcome assessors
Incomplete outcome data (attrition bias)	Low risk	Missingness is minimal (n = 2) participants
Selective reporting (reporting bias)	Low risk	No evidence of selective outcome reporting
Other bias	Low risk	No evidence of other concerns for bias

Essock et al., 1998

**Table:** 

**Methods**	**Design**: Randomized control trial and cost‐effectiveness analysis
	**Setting**: Connecticut, Mas, United States
	**Follow up**: 18 months
**Participants**	**Population**: Homeless clients who were high service users with serious mental disorders
	**Sample size**: Total sample n = 262, ACT n = 131, SCM n = 131
**Interventions**	**Intervention**:
	ACT: Mobile and multidisciplinary, included nurses and at least a half‐time psychiatrist, and had relatively rich staffing ratios (one staff member for every five to seven clients). This staff provided coverage for two shifts; coverage for a third shift was provided either by ACT staff on beeper or a mobile crisis team with whom the ACT teams worked closely. The ACT teams served 50‐71 clients who received case management, outpatient clinical services (such as medication management and group therapy), mobile outreach and crisis intervention services
	**Comparator**:
	SCM interventions: SCM case managers had caseloads of 25‐30 clients. Those receiving SCM averaged one to 2 h per month with their case manager. SCM clients had access to these same services, either as provided by their individual case manager or via specialized service providers to which the case manager arranged linkage. Clients from each condition had access to the same array of residential services, psychosocial clubs, crisis/respite programs, and vocational service providers
**Outcomes**	Mental health, Quality of life, Hospitalization
	**Cost or cost‐effectiveness**: The mean annual cost to society for a client in the ACT condition was $33,473 (SD=32,838), compared to $35,656 (SD= 39,446) for those in the SCM condition. ACT clients spent significantly more time in the community than did SCM clients. The mean number of community days during the target year was 301 (SD=115.5) for ACT clients and 261 (SD=144.8) for SCM clients (p<.05). ACT led to fewer homeless days and lower societal costs than SCM. For the ACT group, an effectiveness of 301 community days was divided by $33.5 thousand in societal costs to equal nine community days expenditure; for the SCM group, 261 community days/$35.7 thousand in societal costs=7.3 community days per $1,000 expenditure. ACT was more cost‐effective for those clients who were hospitalized at study entry.
**Notes**	

Risk of bias table

**Table:** 

Bias	Authors' judgement	Support for judgement
Random sequence generation (selection bias)	Unclear risk	Randomization was counterbalanced so that, within each of the three study sites, half of the study participants were assigned to ACT and half to SCM. Group assignments were also counterbalanced within each site so that the clients hospitalized at the time of assignment had a 50% likelihood of being assigned to either ACT or SCM. It was unclear if the block randomization reduced the likelihood of foreknowledge of intervention assignment.
Allocation concealment (selection bias)	Unclear risk	No description of allocation concealment was provided.
Blinding of participants and personnel (performance bias)	High risk	Blinding of participants and personnel was not possible due to the nature of the intervention and study design.
Blinding of outcome assessment (detection bias)	Unclear risk	No description of blinding of outcome assessment was provided.
Incomplete outcome data (attrition bias)	Low risk	Of the 339 clients who met the study criteria and approached by their case managers for informed consent, 262 (77%) agreed to participate and were randomly assigned to receive ACT or SCM. “some data were available only after evaluation funding was secured…” Once the evaluation funding was in place and interviews could be scheduled, 88% of interviews due were completed.
Selective reporting (reporting bias)	Low risk	No evidence of reporting outcome selectively was detected.
Other bias	Low risk	No other potential biases were detected.

Essock et al., 2006

**Table:** 

**Methods**	**Design**: Randomized control trial
	**Setting**: Connecticut, Mas, United States
	**Follow up**: 3 years
**Participants**	**Population**: Homeless adults with mental illness and an active substance use disorder
	**Sample size**: Total sample n = 198, ACT n = 99, SCM n = 99
**Interventions**	**Intervention**:
	Assertive Community Treatment (ACT): Mobile and multidisciplinary, included nurses and at least a half‐time psychiatrist, and had relatively rich staffing ratios (one staff member for every five to seven clients). This staff provided coverage for two shifts; coverage for a third shift was provided either by ACT staff on beeper or a mobile crisis team with whom the ACT teams worked closely. The ACT teams served 50‐71 clients who received case management, outpatient clinical services (such as medication management and group therapy), mobile outreach and crisis intervention services
	**Comparator**:
	Standard case management: Delivered at least some services in the community, had clinicians work with the clients' support systems, and vigorously addressed substance use disorders. Because clinicians in the standard clinical case management group had caseloads of approximately twice as many clients as clinicians in the assertive community treatment group, they provided fewer services directly.
**Outcomes**	Mental health, Quality of life, Hospitalization
**Notes**	

Risk of bias table

**Table:** 

Bias	Authors' judgement	Support for judgement
Random sequence generation (selection bias)	Low risk	“Study participants completed baseline assessments and were randomly assigned within the two sites to either assertive community treatment or standard case management. Randomization was managed centrally by using separate computer‐generated randomization streams for each site”.
Allocation concealment (selection bias)	Unclear risk	No description of allocation concealment was provided.
Blinding of participants and personnel (performance bias)	High risk	Blinding of participants and personnel was not possible due to the nature of the intervention and study design.
Blinding of outcome assessment (detection bias)	Unclear risk	No description of blinding of outcome assessment was provided.
Incomplete outcome data (attrition bias)	Low risk	“… few data were missing and no statistical differences were found between the recruited and retained study groups”.
Selective reporting (reporting bias)	Low risk	No evidence of reporting outcome selectively was detected.
Other bias	Low risk	No other potential biases were detected.

Evans et al., 2016

**Table:** 

**Methods**	**Design**: Cost‐benefit analysis based on Chicago administrative data
**Participants**	**Population**: Chicago residents at risk of becoming homeless who contacted the Homelessness Prevention Call Center and requested for rent or security deposits
**Interventions**	**Intervention**:
	Callers who were referred for a temporal funding assistance
	**Comparator**:
	Callers who were not referred for funding assistance
**Outcomes**	**Cost or cost‐effectiveness**: The averted cost per homeless individual was US$10,300; the per‐person cost of averting a new case of homelessness among very low‐income families was $6,800 (35% lower than the per‐person cost among all eligible callers)
**Notes**	

Risk of bias table

**Table:** 

Bias	Authors' judgement	Support for judgement
Random sequence generation (selection bias)	Unclear risk	
Allocation concealment (selection bias)	Unclear risk	
Blinding of participants and personnel (performance bias)	Unclear risk	
Blinding of outcome assessment (detection bias)	Unclear risk	
Incomplete outcome data (attrition bias)	Unclear risk	
Selective reporting (reporting bias)	Unclear risk	
Other bias	Unclear risk	

Felton et al., 1995

**Table:** 

**Methods**	**Design**: 3‐arm quasi experimental trial
	**Setting**: Bronx, New York city, United States
	**Follow up**: 18‐months
**Participants**	**Population**: Homeless individuals, long‐term psychiatric inpatients, and heavy users of emergency services with serious and persistent mental illness
	**Sample size**: Total sample size n = 104, ICM‐peer n = 36, ICM‐para n = 36, ICM‐only n = 32
**Interventions**	**Intervention**:
	ICM‐peer: Three consumer peer specialists were added to one unit, received 8 week of case management training before assignment and additional training in peer counselling and self‐help.
	ICM‐para: 3 Bronx residents with no previous experience as mental mental health consumers or provided were added to a second unit as para‐professional and received 8 weeks of case management training before assignment
	**Comparator**:
	ICM‐Only: Key program features include small caseload(roughly 10 clients/worker), 24 hr availability of staff, a rehabilitation orientation, and assertive outreach and advocacy
**Outcomes**	Quality of life, Mental health
**Notes**	

Risk of bias table

**Table:** 

Bias	Authors' judgement	Support for judgement
Random sequence generation (selection bias)	High risk	This is a Quasi‐experimental trial with no random assignment to study condition.
Allocation concealment (selection bias)	High risk	This is a Quasi‐experimental trial with no allocation concealment description provided.
Blinding of participants and personnel (performance bias)	High risk	Blinding of participants and personnel was not possible due to the nature of the intervention and study design.
Blinding of outcome assessment (detection bias)	Low risk	“Data were gathered via face‐to‐face interviews blind to each client's treatment condition”.
Incomplete outcome data (attrition bias)	Low risk	No evidence of incomplete outcome data was detected.
Selective reporting (reporting bias)	Low risk	No evidence of reporting outcome selectively detected.
Other bias	Low risk	No other potential biases detected.

Ferguson, 2018

**Table:** 

**Methods**	**Design**: Randomized control trial
	**Setting**: Los Angeles, CA, United States
	**Follow up**: Over 20 months
	**Recruitment**: From one homeless youth agency
	**Randomization**: Participants were randomized using a computer generated algorithm with blocking
	**Allocation**: Participants selected a sealed envelope containing the name of the condition
	**Blinding**: Not reported in the publication
	**Timing of outcome assessment**: At baseline (study months 1‐4) and follow‐up (study months 20‐24)
	**Outcome assessor**: The primary investigator and research assistants
**Participants**	**Population**: Homeless youth with mental illness
	**Sample size**: Total n = 72, SEI n = 36, IPS n = 36
**Interventions**	**Intervention**:
	The social enterprise intervention model was implemented in four stages: vocational skill acquisition (4 months), small business skill acquisition (4 months), social enterprise intervention formation and product distribution (12 months), and case‐management services (ongoing for 20 months); social enterprise intervention participants attended vocational and small business classes twice a week (1.5 h per session) and received case‐management services continuously throughout the 20 month study period.
	**Comparator**:
	Over the 20 months, all IPS participants met individually with the employment specialist, one case manager, and one clinician at least weekly. Meetings took place either in person within the agency or in the community, by phone, or through social media check‐ins.
**Outcomes**	Housing stability, mental health, quality of life, employment, income
**Notes**	

Risk of bias table

**Table:** 

Bias	Authors' judgement	Support for judgement
Random sequence generation (selection bias)	Low risk	Random sequence was generated via computer algorithm.
Allocation concealment (selection bias)	Low risk	Eligible participants selected a sealed envelope containing the name of a study group.
Blinding of participants and personnel (performance bias)	High risk	One arm received intervention in a peer‐based group format; the other was in an individualized format. Technically difficult to blind the participants and personnel.
Blinding of outcome assessment (detection bias)	Unclear risk	Data was collected through interviews conducted by PI or research assistants. No details addressing blinding of outcome assessment were provided.
Incomplete outcome data (attrition bias)	Low risk	No evidence of incomplete outcome reporting or attrition bias was detected.
Selective reporting (reporting bias)	Low risk	No evidence of reporting outcomes selectively was detected.
Other bias	High risk	The study design was underpowered with an inadequate sample size. The fidelity of individual programs was not assessed with objective pre‐tested scales but was subjectively assessed by agency administrative staff. The outcome measures missed detailed variables to assess effect on employment quantitatively. The study design also lacked non‐treatment or placebo controls.

Fletcher et al., 2008

**Table:** 

**Methods**	**Design**: 3‐arm randomized control trial
	**Setting**: St. Louis, MO, United States
	**Follow up**: 30 months
**Participants**	**Population**: Homeless adults with severe mental illness and a substance use disorder
	**Sample size**: Total sample n = 191 [No description of allocation]
**Interventions**	**Intervention**:
	The IACT team had a substance abuse specialist on staff and provided outpatient substance abuse counseling and bi‐weekly treatment groups.
	The ACTO team referred clients to other community providers for outpatient or individual substance abuse services and to 12‐step groups.
	**Comparator**:
	Participants assigned to SC were shown a list of community agencies that provided mental health and substance abuse treatment. Research staff provided these participants with information about treatment openings and assisted individuals in making their initial contact with an agency.
**Outcomes**	Housing stability, Mental health, Substance use
**Notes**	

Risk of bias table

**Table:** 

Bias	Authors' judgement	Support for judgement
Random sequence generation (selection bias)	Unclear risk	No description of random sequence generation was provided.
Allocation concealment (selection bias)	Unclear risk	No description of allocation concealment was provided.
Blinding of participants and personnel (performance bias)	High risk	Blinding of participants and personnel was not possible due to the nature of the intervention and study design.
Blinding of outcome assessment (detection bias)	Unclear risk	No description of blinding of outcome assessment was provided.
Incomplete outcome data (attrition bias)	High risk	“… tests of independence (v2) did not show significant signs of attrition over time by condition… Attrition did not become evident until the last two time periods (i.e., 10–20% of individuals lost)”
Selective reporting (reporting bias)	Low risk	No evidence of reporting outcomes selectively was detected.
Other bias	Low risk	**No other potential biases were detected**.

Forchuk et al., 2008

**Table:** 

**Methods**	**Design**: Randomized control trial
	**Setting**: Ontario, Canada
	**Follow up**: 6 months
	**Recruitment**: From an acute care psychiatric ward within a general hospital and a tertiary care psychiatric hospital
	**Randomization**: Participants chose an envelope which determined their condition allocation
	**Allocation**: Not reported in the publication
	**Blinding**: Not reported in the publication
	**Timing of outcome assessment**: Before discharge, and then again at 3‐ and 6‐ months post discharge
	**Outcome assessor**: Not reported in the publication
**Participants**	**Population**: Individuals discharged from psychiatric wards to shelters and who are precariously housed
	**Sample size**: Total n=14, Intervention n=7, control n= 7
**Interventions**	**Intervention**:
	Assistance finding housing through a housing advocate and so‐called fast tracked income support.
	**Comparator**:
	Control group: usual care which did not include direct or immediate assistance with housing, but included referral to social work for housing support if requested by the healthcare team during inpatient stay.
**Outcomes**	Housing stability
**Notes**	

Risk of bias table

**Table:** 

Bias	Authors' judgement	Support for judgement
Random sequence generation (selection bias)	Low risk	Participants who were at risk of being discharged to No Fixed Address were enrolled in the study and completed an initial interview in hospital. At the end of the interview, they chose an envelope which determined whether they were in the intervention or control group. All participants were randomly selected using this envelope method to avoid selection bias
Allocation concealment (selection bias)	Unclear risk	No description of allocation concealment was provided.
Blinding of participants and personnel (performance bias)	High risk	Blinding of participants and personnel was not possible due to the nature of the intervention and study design.
Blinding of outcome assessment (detection bias)	Unclear risk	No description of blinding of outcomes assessment was provided.
Incomplete outcome data (attrition bias)	High risk	Most of those in the control group (five of seven) did not want a full interview at the later time periods. The primary outcome examining whether individuals were housed or not housed post‐hospitalization was obtained from all 14 participants. Only Findings of the primary outcome measure will be presented in this article
Selective reporting (reporting bias)	Low risk	No evidence of reporting outcomes selectively was detected.
Other bias	High risk	The results of this pilot study were so dramatic that randomizing to the control group stopped and plans began to routinely implement the intervention. Also, this study had a Small population size of n=14. “However, the sample was randomized and data do provide new knowledge which has the potential to contribute to mental health care and mental health nursing in inpatient setting”

Gilmer et al., 2009

**Table:** 

**Methods**	**Design**: A cost analysis based on a quasi‐experimental difference‐in‐difference design
**Participants**	**Population**: Homeless people in San Diego county
**Interventions**	**Intervention**:
	REACH clients
	**Comparator**:
	Non‐REACHindividuals(control)
**Outcomes**	**Cost or cost‐effectiveness**: Compared with the control group, case management costs increased by US$6,403 (p<0.001), inpatient and emergency costs declined by $6,103 (p = 0.034), and criminal justice system costs declined by $570 (p = 0.020); no significant differences were identified in outpatient or total costs; REACH clients incurred additional total cost of $417 over 2 years compares with the control group, but the difference was not statistically significant; the total cost of the service was $20,241.
**Notes**	

Risk of bias table

**Table:** 

Bias	Authors' judgement	Support for judgement
Random sequence generation (selection bias)	Unclear risk	
Allocation concealment (selection bias)	Unclear risk	
Blinding of participants and personnel (performance bias)	Unclear risk	
Blinding of outcome assessment (detection bias)	Unclear risk	
Incomplete outcome data (attrition bias)	Unclear risk	
Selective reporting (reporting bias)	Unclear risk	
Other bias	Unclear risk	

Gilmer et al., 2010

**Table:** 

**Methods**	**Design**: A cost analysis of San Diego County Adult and Older Adult Mental Health Services encounter‐based management information system
**Participants**	**Population**: Homeless adults and residents with severe mental illness in San Diego county
**Interventions**	**Intervention**:
	Residents who received the full‐service partnerships
	**Comparator**:
	Residents who did not receive full‐ service partnerships (control)
**Outcomes**	**Cost or cost‐effectiveness**: Full‐service partnerships increased annual per person outpatient costs by $9,180 (p<0.001), but decreased annual costs per person by $6,882 for inpatient costs (p<0.001), by $1,721 for emergency services (p=0.002), and by $1,641 for mental health services received in jail (p<0.001); the difference‐in‐difference estimate of the effect of full‐service partnerships on total costs was not significant ($2,116; p=0.45).
**Notes**	

Risk of bias table

**Table:** 

Bias	Authors' judgement	Support for judgement
Random sequence generation (selection bias)	Unclear risk	
Allocation concealment (selection bias)	Unclear risk	
Blinding of participants and personnel (performance bias)	Unclear risk	
Blinding of outcome assessment (detection bias)	Unclear risk	
Incomplete outcome data (attrition bias)	Unclear risk	
Selective reporting (reporting bias)	Unclear risk	
Other bias	Unclear risk	

Goldfinger et al., 1999

**Table:** 

**Methods**	**Design**: Randomized control trial
	**Setting**: Boston, United States
	**Follow up**: 18 months
	**Recruitment**: Residents of homeless shelters for mentally ill were identified and screened
	**Randomization**: Participants were randomly assigned. No mention of randomization procedures
	**Allocation**: No mention of allocation procedures
	**Blinding**: Not mentioned
	**Timing of outcome assessment**: Data collected for up to 18 months
	**Outcome assessor**: Not mentioned
**Participants**	**Population**: Homeless adults with mental illness
	**Sample size**: Total n = 118, Group housing n = 63, Independent apartments n = 55
**Interventions**	**Intervention**:
	Evolving Consumer Household model; a shared housing arrangement that provides more independence while minimising the presumed risks of living independently or in traditional group homes; the model is designed to offer residents permanent secure housing without the requirement of treatment compliance; staff are trained to facilitate consumer independence, and the number of staff is expected to be gradually reduced as consumers learn the skills needed to manage the house themselves.
	**Comparator**:
	Independent‐living apartments: One‐ or two‐ room single apartments in public housing projects or large multiunit sites subsidized by the Boston Housing Authority.
**Outcomes**	Housing stability, quality of life
**Notes**	

Risk of bias table

**Table:** 

Bias	Authors' judgement	Support for judgement
Random sequence generation (selection bias)	Unclear risk	Participants were randomized into treatment condition. However, no description of random sequence generation was provided.
Allocation concealment (selection bias)	Unclear risk	No description of allocation concealment was provided.
Blinding of participants and personnel (performance bias)	High risk	Blinding of participants and personnel was not possible due to the nature of the intervention and study design.
Blinding of outcome assessment (detection bias)	Unclear risk	No description of blinding of outcome assessment was provided.
Incomplete outcome data (attrition bias)	Low risk	“At 18 months, three of the original 118 study participants had died, three had moved out of state, and two who were initially assigned to independent apartments were excluded from the analysis because, by law, their criminal records precluded their being randomly assigned to live in apartments operated by the Boston Housing Authority”. “Study participants for whom follow‐up data collection was not complete did not differ significantly at baseline on any measure from those who completed the follow‐up”
Selective reporting (reporting bias)	Low risk	No evidence of reporting outcomes selectively was detected.
Other bias	Low risk	No other potential biases were detected.

Grace & Gill, 2014

**Table:** 

**Methods**	**Design**: Non‐randomized control trial
	**Setting**: Victoria, Australia
	**Follow up**: 24 months
**Participants**	**Population**: Unemployed and homeless young people
	**Sample size**: Total sample n = 422, Intervention n=235, control n = 187
**Interventions**	**Intervention**:
	Intensive client‐centered case management, involving direct provision of a range of services as well as the brokering of additional services, all through a single point of contact—the YP case manager. The intervention was not standardised in terms of duration and intensity. The defining feature was that J group participants remained eligible for joined up services, and were entitled to re‐engage with those services at any time during the service delivery phase of the trial. At the end of the service delivery phase of the trial, J group reverted to being eligible for standard services.
	**Comparator**:
	S group remained eligible for standard services available in the community, delivered by a range of government and community‐based organisations including housing, employment, counselling, and health services, but without the joining up and single point of contact that were characteristics of the YP4 joined up services that were available to J group. The mode of service delivery was the key difference between the two groups. Standard service delivery involved clients in complex circumstances receiving multiple and potentially uncoordinated services from different providers.
**Outcomes**	Housing stability, Income
**Notes**	

Risk of bias table

**Table:** 

Bias	Authors' judgement	Support for judgement
Random sequence generation (selection bias)	High risk	This was a non‐randomized control trial without randomization to treatment conditions provided.
Allocation concealment (selection bias)	High risk	This was a non‐randomized control trial with no allocation concealment provided.
Blinding of participants and personnel (performance bias)	High risk	Blinding of participants and personnel was not possible due to the nature of the intervention and study design.
Blinding of outcome assessment (detection bias)	Unclear risk	No description of blinding of outcome assessment was provided.
Incomplete outcome data (attrition bias)	High risk	n=26 participants in the J group were excluded from analysis because of missing full Centrelink data.
Selective reporting (reporting bias)	High risk	Study design included follow‐up to 36 months but data only available until the 24 months follow‐up.
Other bias	High risk	**The process for allocating participants to J group or S group varied by site as well as over time**.

Graham‐Jones et al., 2004

**Table:** 

**Methods**	**Design**: Quasi‐ experimental 3‐armed controlled trial
	**Setting**: Liverpool, United Kingdom
	**Follow up**: 3 years
**Participants**	**Population**: Homeless patients registering on a temporary basis at Prince Park Health Centre
	**Sample size**: Total sample n = 117, Health centre advocacy n = 22, Outreach advocacy n = 53, control n = 42
**Interventions**	**Health centre advocacy group**: During “intervention” months, receptionists registering temporary patients from homeless families at the health centre put these patients in touch with the health advocate (family health worker, FHW) before or soon after their first consultation with a GP.
	**Outreach advocacy group**:
	Outreach visits by the FHW to hostels and bed and breakfast hotels during intervention months allowed newly arrived homeless individuals and families to be proactively registered as temporary patients. Health advocacy work could then be initiated early in their stay in the area.
	**Control group (usual care)**: During “control” months, new temporary resident patients registered themselves at the health centre and accessed usual care (including appointments/home visits with a GP or visits to the practice nurse).
**Outcomes**	Housing stability, Quality of life
**Notes**	

Risk of bias table

**Table:** 

Bias	Authors' judgement	Support for judgement
Random sequence generation (selection bias)	High risk	This was a quasi‐experimental trial without randomization to intervention conditions.
Allocation concealment (selection bias)	High risk	This was a quasi‐experimental trial without allocation concealment.
Blinding of participants and personnel (performance bias)	High risk	Blinding of participants and personnel was not possible due to the nature of the intervention and study design.
Blinding of outcome assessment (detection bias)	Unclear risk	No description of blinding of outcome assessment was provided.
Incomplete outcome data (attrition bias)	Low risk	“The high attrition rate (47%) makes it important to consider the effects of bias and the way in which this night affect the results… The follow‐up sample was shown to be representative of the full study sample, both demographically and in terms of baseline QOL, and despite attrition, the sizes of the groups were large enough to detect significant differences”.
Selective reporting (reporting bias)	Low risk	No evidence of reporting outcomes selectively was detected.
Other bias	High risk	“Selection bias was a potential weakness of the study since the two intervention groups were recruited at different sites (at the health centre versus hostel/hotel outreach)**”**.

Gubits et al., 2018

**Table:** 

**Methods**	**Design**: 4‐arm randomized control trial
	**Setting**: Alameda country (CA), Atlanta (GA), Balitmore (M), Boston (MA), Bridgeport and New Haven (CT), Denver (CO), Honolulu (HI), Kansas City (MO), Louisville (KY), Minneapolis (MN), Phoenix (AZ), and Salt Lake City (UT), USA
	**Follow up**: Up to 37 months
	**Recruitment**: Families were recruited from emergency shelters
	**Randomization**: Families were randomized when eligible for a program. Procedures were not reported
	**Allocation**: Not reported in the publication
	**Blinding**: Not reported in the publication
	**Timing of outcome assessment**: At baseline and 20‐ and 37 months after
	**Outcome assessor**:
**Participants**	**Population**: Homeless families with a child aged 15 years or younger
	**Sample size**: Total n=2282, SUB n=599, CBRR n=569, PBTH n=368, UC n=746
**Interventions**	**Intervention**:
	Permanent housing subsidy, community‐based rapid rehousing, or project‐based temporary housing: permanent housing subsidy participants received a choice voucher; community‐based rapid rehousing participants received temporary rental assistance, renewable for up to 18 months paired with limited, housing‐focused services; project‐based temporary housing participants received temporary housing for up to 24 months in agency‐controlled buildings or apartment units, paired with supportive services; permanent housing subsidy n = 599, community‐based rapid rehousing n = 569, project‐based temporary housing n = 368
	**Comparator**:
	UC (Usual care): The control group were not offered priority access to any type of homeless or housing assistance. However, families randomized to this group were able to use any housing services in the community. This condition also included some additional stay in the emergency shelter.
**Outcomes**	Housing stability, mental health, substance use, employment, income
**Notes**	

Risk of bias table

**Table:** 

Bias	Authors' judgement	Support for judgement
Random sequence generation (selection bias)	High risk	Due to a combination of service availability and program eligibility, only 474 families had all four randomization arms available to them; 1,544 families had three randomization options; and 264 families had two randomization options. Families without at least one option in addition to usual care were not enrolled in the study.
Allocation concealment (selection bias)	Unclear risk	The article did not address the allocation concealment process.
Blinding of participants and personnel (performance bias)	High risk	Families who were assigned to one of the three active interventions were provided priority access to that intervention. Families assigned to usual care initially continued to stay in shelters.
Blinding of outcome assessment (detection bias)	Unclear risk	No details regarding the outcome assessment process or whether the researchers were blinded when collecting the data were provided. “Most measures were reported by family heads and refer to the period immediately before the follow‐up surveys”.
Incomplete outcome data (attrition bias)	Low risk	No evidence of incomplete outcome reporting or attrition bias was detected.
Selective reporting (reporting bias)	Low risk	“We pre‐selected 18 outcomes, three or four from each domain for presentation in the executive summary of technical reports and in this paper, to avoid cherry‐picking of possibly spurious statistically significant findings”
Other bias	Low risk	No other potential biases were detected.

Gulcur 2003

**Table:** 

**Methods**	**Design**: A cost analysis based on a randomised trial
**Participants**	**Population**: Chronically homeless individuals with severe mental illness and often substance abuse (including people living on the streets and those who had lived on the streets previously, but who resided in psychiatric hospitals immediately before study entry)
**Interventions**	**Intervention**:
	Housing First programme provided immediate access to independent apartments and supportive services, without prerequisites for sobriety or participation in psychiatric treatment and support services through a multidisciplinary assertive community treatment team
	**Comparator**:
	Continuum of care programme (control)
**Outcomes**	**Cost or cost‐effectiveness**: Costs associated with days spent on the streets, such as costs of outpatient services and societal costs were excluded; overall costs accrued by the control group were significantly higher than the intervention group (p<0.05); costs decreased to a greater extent among individuals recruited from the hospital than the sample recruited from the streets
**Notes**	

Risk of bias table

**Table:** 

Bias	Authors' judgement	Support for judgement
Random sequence generation (selection bias)	Unclear risk	
Allocation concealment (selection bias)	Unclear risk	
Blinding of participants and personnel (performance bias)	Unclear risk	
Blinding of outcome assessment (detection bias)	Unclear risk	
Incomplete outcome data (attrition bias)	Unclear risk	
Selective reporting (reporting bias)	Unclear risk	
Other bias	Unclear risk	

Herman et al., 2011

**Table:** 

**Methods**	**Design**: Randomized control trial
	**Setting**: New York, United States
	**Follow up**: 18 months
**Participants**	**Population**: Homeless mentally ill participants after hospital discharge
	**Sample size**: Total sample n = 150, CTI n = 77, Control n = 73
**Interventions**	**Intervention**:
	CTI: Nine‐month case management program delivered in three phases of 3 months each; Phase 1: "Transition to the community": providing intensive support and assessing the resources that exist for the transition of care to community providers. The CTI worker generally makes detailed arrangements in only the handful of areas seen as most critical for community survival of that individual. Phase 2: "Try out": CTI worker can focus on assessing the degree to which this support system is functioning as planned. In this phase, the worker will intervene only when modification in the system is needed or when a crisis occurs. Phase 3: "Transfer of care": focuses on completing the transfer of responsibility to community resources that will provide long‐term support.
	**Comparator**:
	“Usual” community‐based services depending on the individual's needs, preferences and living situation. These services usually included various types of case management and clinical treatment.
**Outcomes**	Housing stability, Hospitalization
**Notes**	

Risk of bias table

**Table:** 

Bias	Authors' judgement	Support for judgement
Random sequence generation (selection bias)	Low risk	“Participants were randomized independently by gender and by diagnosis of lifetime substance use disorder. To reduce variation of key factors, we randomized individuals in these four strata in permuted blocks of randomly varying size”.
Allocation concealment (selection bias)	High risk	“The names of eligible participants and their respective randomization stratum were given to an administrator who did not need to be blind to treatment status”.
Blinding of participants and personnel (performance bias)	High risk	Blinding of participants and personnel was not possible due to the nature of the intervention and study design.
Blinding of outcome assessment (detection bias)	Low risk	“These assessments were carried out by trained interviewers blind to the participant's group assignment".
Incomplete outcome data (attrition bias)	Low risk	No evidence of incomplete outcome data reporting was detected.
Selective reporting (reporting bias)	Low risk	No evidence of reporting outcomes selectively was detected.
Other bias	Low risk	No other potential biases were detected.

Holtgrave et al., 2013

**Table:** 

**Methods**	**Design**: Cost‐utility analysis based on data from the Housing and Health study
**Participants**	**Population**: Homeless and unstably housed people with HIV in Baltimore, Chicago and Los Angeles
**Interventions**	**Intervention**:
	Housing and health services
	**Comparator**:
	No supportive housing
**Outcomes**	**Cost or cost‐effectiveness**: Among Housing and Health Study participants, 0.01567 HIV transmissions were averted per person, and QALYs increased by 0.0324 due to improvements in perceived stress; averting one case of HIV transmission saved 9 years of life, 11.55 undiscounted QALYs, and 5.33 QALYs discounted at 3%; the cost per QALY‐ gained by the provision of housing services in the Housing and Health Study was $62,493
**Notes**	

Risk of bias table

**Table:** 

Bias	Authors' judgement	Support for judgement
Random sequence generation (selection bias)	Unclear risk	
Allocation concealment (selection bias)	Unclear risk	
Blinding of participants and personnel (performance bias)	Unclear risk	
Blinding of outcome assessment (detection bias)	Unclear risk	
Incomplete outcome data (attrition bias)	Unclear risk	
Selective reporting (reporting bias)	Unclear risk	
Other bias	Unclear risk	

Hunter et al., 2017

**Table:** 

**Methods**	**Design**: A cost analysis based on a before‐ and‐after study using administrative databases
**Participants**	**Population**: Homeless individuals who participated in the Housing for Health programme
**Interventions**	**Intervention**:
	Pre‐ and post‐ permanent supportive housing component of the Housing for Health programme
	**Comparator**:
	N/A
**Outcomes**	**Cost or cost‐effectiveness**: Permanent supportive housing was associated with an 80% reduction in emergency room visits, a 61% reduction in days spent as an inpatient, a 47% reduction in medical health outpatient visits, a 44% reduction in mental health outpatient visits, a 28% reduction in general relief receipt, and a two‐fold increase in days spent incarcerated; a 20% programme cost offset was observed for direct service costs for 1 year before housing provision versus 1 year after housing provision, suggesting that permanent supportive housing expenses might be partially offset by savings other Los Angeles county funds
**Notes**	

Risk of bias table

**Table:** 

Bias	Authors' judgement	Support for judgement
Random sequence generation (selection bias)	Unclear risk	
Allocation concealment (selection bias)	Unclear risk	
Blinding of participants and personnel (performance bias)	Unclear risk	
Blinding of outcome assessment (detection bias)	Unclear risk	
Incomplete outcome data (attrition bias)	Unclear risk	
Selective reporting (reporting bias)	Unclear risk	
Other bias	Unclear risk	

Hurlburt et al., 1996

**Table:** 

**Methods**	**Design**: 4‐arm randomized control trial
	**Setting**: San Diego, CA, United States
	**Follow up**: up to 24 months
	**Recruitment**: Eligible individuals were referred to the San Diego project from a variety of sources
	**Randomization**: Participants were randomized but procedures were not reported
	**Allocation**: Allocation was not reported
	**Blinding**: Blinding was not reported
	**Timing of outcome assessment**: At baseline and every 6 months for 24 months
	**Outcome assessor**: Research staff members
**Participants**	**Population**: Individuals diagnosed with severe and persistent mental illness, who were either currently homeless or at high risk of becoming homeless
	**Sample size**: Total n=362
	Intervention (comprehensive CM with housing vouchers) n = 90‐91
	Control (comprehensive CM without vouchers) n = 90‐91
	Intervention (traditional CM with vouchers) n = 90‐91
	Control(traditional CM without vouchers) n = 90‐91
**Interventions**	**Intervention**:
	Comprehensive or traditional case management and housing vouchers from the Department of Housing and Urban Development to local housing authorities, allowing participants to choose and obtain independent housing in the community; comprehensive case management included private mental health services (under contract with the county) with smaller maximum caseloads; available to clients 24 h a day, 7 days per week; a formal team approach was used with participants, and access to housing support groups and employment search support was offered.
	**Comparator**:
	Control group: no housing vouchers but provided with case management
**Outcomes**	Housing stability
**Notes**	

Risk of bias table

**Table:** 

Bias	Authors' judgement	Support for judgement
Random sequence generation (selection bias)	Unclear risk	Participants were randomized to one of four treatment conditions. However, no description of random sequence generation was provided.
Allocation concealment (selection bias)	Unclear risk	No description of allocation concealment was provided.
Blinding of participants and personnel (performance bias)	High risk	Blinding of participants and personnel was not possible due to the nature of the intervention and study design.
Blinding of outcome assessment (detection bias)	Unclear risk	No description of blinding of outcome assessment was provided.
Incomplete outcome data (attrition bias)	Unclear risk	“On the other hand, clients without access to Section 8 certificates were 3.4 times more likely to achieve other types of community housing in the first six months than clients with access to Section 8 certificates“
Selective reporting (reporting bias)	Low risk	No evidence of reporting outcome selectively was detected.
Other bias	Low risk	No other potential biases were detected.

Hwang et al., 2011

**Table:** 

**Methods**	**Design**: Quasi‐experimental trial
	**Setting**: Toronto, Canada
	**Follow up**: 18 months
	**Recruitment**: This study sought to enroll applicants to the supportive housing program
	**Randomization**: No randomization was performed
	**Allocation**: Not mentioned
	**Blinding**: Not mentioned
	**Timing of outcome assessment**: At baseline, and every 6 months for up to 18 months
	**Outcome assessor**: Not mentioned
**Participants**	**Population**: Financially disadvantaged homeless adults
	**Sample size**: Total n = 112, Intervention n = 46, TAU n = 66
**Interventions**	**Intervention**:
	Supportive housing program located in one building with 84 units; tenants had access to a drop‐in centre offering meals and outreach services, as well as a medical and dental clinic providing free services; individuals received rental subsidies and paid rent tailored to their income (not exceeding 30% of income); the programme partnered with COTA Health, a mental health and community support services organisation that provided onsite support to tenants.
	**Comparator**:
	Those who were waitlisted for the same program
**Outcomes**	Housing stability, mental health, quality of life, substance use, hospital admission
**Notes**	

Risk of bias table

**Table:** 

Bias	Authors' judgement	Support for judgement
Random sequence generation (selection bias)	High risk	This is a quasi‐experimental study without randomization to treatment condition.
Allocation concealment (selection bias)	High risk	This is a quasi‐experimental study without allocation concealment.
Blinding of participants and personnel (performance bias)	High risk	Blinding of participants and personnel was not possible due to the nature of the intervention and study design.
Blinding of outcome assessment (detection bias)	Unclear risk	No description of blinding of outcome assessment was provided.
Incomplete outcome data (attrition bias)	Low risk	“There was no significant difference in the proportion of participants lost to follow‐up between the intervention and usual care groups at 18 months”
		“Participants who were lost to follow‐up did not significantly differ from participants who had data for all three follow‐up interviews with respect to demographic characteristics, housing status, health status, quality of life, substance use, or health care utilization at baseline”
Selective reporting (reporting bias)	Low risk	No evidence of reporting outcome selectively was detected.
Other bias	High risk	“There were significant differences between groups for baseline demographics, including race and country of birth”

Kashner, 2002

**Table:** 

**Methods**	**Design**: Randomized control trial
	**Setting**: Bedford and Northampton (MA), Topeka (KS), St Cloud (MN), USA
	**Follow up**: 12 months
	**Recruitment**: Participants were recruited from homeless centres and addiction units at 4 VA medical centres
	**Randomization**: Patients were randomized using random patient identification numbers
	**Allocation**: The coordinator opened a sealed envelope with the ID number, in sequence, to reveal the allocation
	**Blinding**: Neither patient nor assessors were blinded to study assignments.
	**Timing of outcome assessment**: At baseline and every 3 months for 1 year
	**Outcome assessor**: Trained interviewers under supervision of master's‐ or doctorate‐level prepared social workers and psychologists.
**Participants**	**Population**: Homeless veterans with substance dependence
	**Sample size**: Total n = 162, Intervention n = 127, Control n=35
**Interventions**	**Intervention**:
	Compensated work therapy, which provided work opportunities (continued employment, higher wages, hours, promotion, and responsibility) based on measures of participant work performance and health behaviour (sobriety and use of recommended addiction services) as determined using client observation, random drug screenings, and chart reviews; the intervention combined elements of supported employment (non‐trivial wages paid from revenues earned from private sector contracts) and stepwise programmes (clinician supervision and Veterans Affairs related workshops); participants were offered employment as soon as a compensated work therapy‐sponsored job became available, usually within 6 days.
	**Comparator**:
	Control group: access to comprehensive rehabilitation, addictions, psychiatric, and medical services
**Outcomes**	Housing stability, mental health, substance use, hospital admissions
**Notes**	

Risk of bias table

**Table:** 

Bias	Authors' judgement	Support for judgement
Random sequence generation (selection bias)	Low risk	Research assistants admitted patients, checked eligibility, administered baseline questionnaires, and then called a national coordinator in Dallas, TX to receive a patient identification number. Once the subject was assigned to a number, the coordinator revealed treatment assignment by opening, in sequence, sealed envelopes.
Allocation concealment (selection bias)	Low risk	The closed assignments in sealed envelopes were based on random numbers generated by an SPSS program (SPSS Inc, Chicago,Ill)
Blinding of participants and personnel (performance bias)	High risk	Blinding of participants and personnel was not possible due to the nature of the intervention and study design.
Blinding of outcome assessment (detection bias)	High risk	After baseline interview, neither patients nor assessors were blinded to study assignment.
Incomplete outcome data (attrition bias)	Unclear risk	Biases associated with non randomly occurring missing data and variable intervals between repeated measures may also be small. [not clear]
Selective reporting (reporting bias)	Low risk	No evidence of reporting outcomes selectively was provided.
Other bias	High risk	Findings were limited to…a low consent rate (47%) and a 1:4 sampling ratio that further limited statistical power. control group subjects wanting to entre CWT after completing the study had incentives to also underreport symptoms

Korr & Joseph, 1996

**Table:** 

**Methods**	**Design**: Randomized control trial
	**Setting**: Chicago, Ill, United States
	**Follow up**: 6 months
**Participants**	**Population**: Homeless mentally ill adults aged 18 or older
	**Sample size**: Total sample n = 114, Intervention n = 48, control n = 47, At risk n = 19
**Interventions**	**Intervention**:
	Bridge services that provide assertive outreach and service coordination through staff who work entirely on the street and in the homes of clients to link the client to entitlements such as the Supplementary Security Income and to mental health treatment services, especially medication. They also assist in teaching living skills, linking to rehabilitative services including supported employment. In most cases, the agency also serves as a representative payee to receive the client's disability check
	**Comparator**:
	Control: whatever community services were available at the time of discharge
**Outcomes**	Housing stability, Hospitalization
**Notes**	

Risk of bias table

**Table:** 

Bias	Authors' judgement	Support for judgement
Random sequence generation (selection bias)	Unclear risk	No description of random sequence generation was provided.
Allocation concealment (selection bias)	Unclear risk	No description of allocation concealment was provided.
Blinding of participants and personnel (performance bias)	High risk	Blinding of participants and personnel was not possible due to the nature of the intervention and study design.
Blinding of outcome assessment (detection bias)	Unclear risk	No description of blinding of outcome assessment was provided.
Incomplete outcome data (attrition bias)	High risk	Some data on control group were missing until about six months after the first clients were admitted to the project.
Selective reporting (reporting bias)	Low risk	No evidence of reporting outcomes selectively was detected.
Other bias	Low risk	No other potential biases were found.

Lako et al., 2018

**Table:** 

**Methods**	**Design**: Randomized control trial
	**Setting**: Multi‐city, The Netherlands
	**Follow up**: 9 months
**Participants**	**Population**: Women over the age of 18 staying at a shelter due to intimate partner violence
	**Sample size**: Total sample n = 136, Intervention n = 70, Usual care n = 66
**Interventions**	**Intervention**: CTI, consisting of three phases: (1) transition to the community; (2) try‐out; (3) transfer of care.
	**Comparator**: Care‐as‐usual: Most organizations provided support during regular meetings (1‐3 h per week) for 13‐52 weeks. All organizations employed a strengths based approach.
**Outcomes**	Quality of life, mental health
**Notes**	

Risk of bias table

**Table:** 

Bias	Authors' judgement	Support for judgement
Random sequence generation (selection bias)	Low risk	"generated the randomization sequence using a computer random number generator"
Allocation concealment (selection bias)	Low risk	"The numbers were saved in a secured digital file and concealed until assignment. […] Women were unaware of condition assignment until they met their CTI worker/case manager"
Blinding of participants and personnel (performance bias)	High risk	Women could not be blinded to the intervention, as they received case management services.
Blinding of outcome assessment (detection bias)	High risk	"information about condition assignment was withheld from the research assistants who conducted the follow‐up interviews, some of them became aware of the assigned condition of a few women because the women told them about the services received”
Incomplete outcome data (attrition bias)	Low risk	Minimal drop‐out, drop‐out is even across intervention groups. Authors to an ITT analysis.
Selective reporting (reporting bias)	High risk	"We decided not to report six outcome measures in this work for several reasons, such as missing data (change in protocol; see Online Resource for more information about the excluded outcomes). However, we analyzed and reported these outcomes to the funding bodies (available on request)"
Other bias	High risk	For non‐Dutch women more data were missing due to the necessary shortening of the questionnaire.

Lapham et al., 1996

**Table:** 

**Methods**	**Design**: Randomized control trial
	**Recruitment**: Clients were recruited into Project H&ART by staff of a day shelter for homeless persons, outreach or clinic staff of Albuquerque Health Care for the Homeless (HCH), or by one of the community agencies which provides other services to homeless persons.
	**Setting**: Housing programs and motel‐like accommodations in Albuquerque, New Mexico, US
	Follow‐up: 10 months
**Participants**	**Population**: Homeless alcohol abusers
	**Eligibility criteria**: Individuals had to be homeless, single adult alcohol abusers who had been in the Albuquerque area for at least three months.
	**Sample size**: Total n = 469; Group‐1 n = 161, Group‐2 n = 164, Group‐3 n = 92, Group‐4 n = 52.
	**Baseline characteristics**: “Clients ranged in age from 18‐67, with a median age of 37 years. Baseline comparisons among persons in the four intervention groups revealed no differences in age group, years of education, race/ethnicity, or classification as having alcohol, drug, housing stability, employment, or legal problems. In each of the groups over 85% of the clients reported alcohol as their primary substance of abuse. The majority of the clients who entered Project H&ART were males; females represented only 13% of the client population. About 41% were non‐Hispanic white (referred to subsequently as “white”); 31% Hispanic white (Hispanic); 18% Native American; and 10% belonged to other race groups. There were some differences in demographic characteristics among members of the different race/ethnic and gender groups. Whites had somewhat higher education levels, with about one third of the population having completed more than 12 years of school. Women were significantly less likely than men to be veterans”
**Interventions**	**Group 1**, the high intensity group, received case management and substance abuse counselling services, along with four months of housing in four‐plex apartment buildings staffed by residence managers who provided peer support.
	**Group 2**, medium intensity group, received four months of housing in similar apartments with support services from peer residence managers. Clients in Group 2 were expected to seek treatment for their alcohol and drug abuse on their own initiatives, from services normally available in the community.
	**Group 3**, low intensity group, received four months of apartment‐ or motel‐based housing and no additional services.
	***About halfway through the 16‐month intervention phase, Group 3 housing services were discontinued due to safety concerns for staff and clients. Individuals randomized to the new low intensity nonhoused group (designated Group 4) received referrals and bus fare to local and statewide alcohol treatment agencies and were paid to provide health services utilization data at twice weekly check‐ins.
	*In all three groups subjects were required to be abstinent from substances of abuse and were subjected to random, and “on demand” breath and urine testing. Those who could not maintain sobriety were discharged from the program.
**Outcomes**	**Housing stability**: Percentage of participants who had stable housing was measured using the Personal History Form (PHF).
	**Substance use**: the number of days of alcohol use using the Addiction Severity Index (ASI).
	**Employment**: Number of days of employment was measured using the Personal History Form.
**Notes**	The only exception to the randomization process was that after two women were randomly assigned to the nonhoused control group a decision was made to randomize all subsequent female participants to one of the housed groups

Risk of bias table

**Table:** 

Bias	Authors' judgement	Support for judgement
Random sequence generation (selection bias)	Unclear risk	Participants were randomized to treatment conditions. However, No description of random sequence generation was provided.
Allocation concealment (selection bias)	Unclear risk	No description of allocation concealment was provided.
Blinding of participants and personnel (performance bias)	High risk	Blinding of participants and personnel was not possible due to the nature of the intervention and study design.
Blinding of outcome assessment (detection bias)	Unclear risk	No description of blinding of outcome assessment was provided.
Incomplete outcome data (attrition bias)	High risk	“Hispanics had higher follow‐up rates than the other groups…persons classified as an employment problem, however, were more likely to have received a 10‐month follow‐up interview…a higher percentage of Group 1 clients received follow‐up interviews, compared to members of the other groups; members of Group 2 had the lowest follow‐up rates”.
Selective reporting (reporting bias)	Low risk	No evidence of reporting outcomes selectively was detected.
Other bias	High risk	“The only exception to the randomization process was that after two women were randomly assigned to the nonhoused control group a decision was made to randomize all subsequent female participants to one of the housed groups”.

Larimer, 2009

**Table:** 

**Methods**	**Design**: Cost analysis based on a quasi‐experimental study
**Participants**	**Population**: Chronically homeless individuals who incurred the highest total costs in 2004 for use of alcohol‐related hospital emergency services, the sobering centre, and incarceration at King County jail (Seattle, WA, USA)
**Interventions**	**Intervention**:
	Housing First Programme
	**Comparator**:
	Waitlist control
**Outcomes**	**Cost or cost‐effectiveness**: A significant difference in total costs was identified between the Housing First and control groups whereby Housing First participants accrued an approximate 53% reduction in costs compared with controls during the first 6 months of the study (relative rate 0.47; 95% CI, 0.25‐0.88); housed participants had $3,569 fewer costs per month during the housed period than control participants; housing costs were $1,120 per person per month but housed participants had $3,569 fewer costs per month during the housed period, yielding a total mean cost offset of $2,449 per person per month for Housing First participants
**Notes**	

Risk of bias table

**Table:** 

Bias	Authors' judgement	Support for judgement
Random sequence generation (selection bias)	Unclear risk	
Allocation concealment (selection bias)	Unclear risk	
Blinding of participants and personnel (performance bias)	Unclear risk	
Blinding of outcome assessment (detection bias)	Unclear risk	
Incomplete outcome data (attrition bias)	Unclear risk	
Selective reporting (reporting bias)	Unclear risk	
Other bias	Unclear risk	

Latimer et al., 2019

**Table:** 

**Methods**	**Design**: A cost‐effectiveness analysis using data from the At Home/Chez Soi randomised controlled trials
**Participants**	**Population**: Adults with mental disorders (at least one of six disorders, including psychotic disorder, major depressive disorder, and post‐traumatic stress disorder) who were absolutely homeless or precariously housed with previous episodes of absolute homelessness
**Interventions**	**Intervention**:
	Housing First plus intensive case management
	**Comparator**:
	Treatment as usual
**Outcomes**	**Cost or cost‐effectiveness**: The cost of providing the Housing First with intensive case management intervention was CAN$14,496; 46% of this cost was offset by a reduction in costs associated with health care, social services, and justice‐related services; compared with treatment as usual, Housing First plus intensive case management was associated with an additional cost of $7,868 and 140 days spent stably housed, with the incremental cost‐effectiveness ratio of $56 per day of stable housing (95% CI 30‐85); Housing First with intensive case management was considered cost‐effective if society was willing to pay at least $56 for each additional day of stable housing
**Notes**	

Risk of bias table

**Table:** 

Bias	Authors' judgement	Support for judgement
Random sequence generation (selection bias)	Unclear risk	
Allocation concealment (selection bias)	Unclear risk	
Blinding of participants and personnel (performance bias)	Unclear risk	
Blinding of outcome assessment (detection bias)	Unclear risk	
Incomplete outcome data (attrition bias)	Unclear risk	
Selective reporting (reporting bias)	Unclear risk	
Other bias	Unclear risk	

Lehman 1997

**Table:** 

**Methods**	**Design**: Randomized control trial
	**Setting**: Baltimore, MD, United States
	**Follow up**: 12 months
**Participants**	**Population**: Homeless persons with mental illness
	**Sample size**: Total sample n = 152, Intervention n = 77, control n = 75
**Interventions**	**Intervention**:
	The experimental condition was the ACT program, modeled after the ACT program first developed by Stein and Test. Each patient was assigned to a "mini‐team" consisting of a clinical case manager (caseload, 10‐12 patients), an attending psychiatrist, and a consumer advocate. The entire ACT team, including the consumer advocates, worked together in decision making and each staff member was knowledgeable about most of the patients. Team‐work was fostered through daily sign‐out rounds and twice‐weekly treatment planning meetings.
	**Control**:
	The comparison condition consisted of services as usual in Baltimore. The public mental health system in Baltimore encompasses 7 community mental health centers operating under a nonprofit, private, local mental health authority, which was developed as part of the Robert Wood Johnson Foundation Program on Chronic Mental illness. Several community‐based psychiatric inpatient and emergency facilities, including those affiliated with 2 major teaching institutions; provide acute inpatient and crisis‐oriented care.
**Outcomes**	Housing stability, Mental health, Quality of life, Hospitalization
**Notes**	

Risk of bias table

**Table:** 

Bias	Authors' judgement	Support for judgement
Random sequence generation (selection bias)	Unclear risk	No description of random sequence generation was provided.
Allocation concealment (selection bias)	Unclear risk	No description of allocation concealment was provided.
Blinding of participants and personnel (performance bias)	High risk	Blinding of participants and personnel was not possible due to the nature of the intervention and study design.
Blinding of outcome assessment (detection bias)	Unclear risk	Source of data was not reported for service use. Quality of life and health survey were self‐reported, and there was no description of blinding of outcome assessment provided.
Incomplete outcome data (attrition bias)	High risk	“Attrition by 12 months was higher among comparison subjects (23%) than among ACT program subjects (13%)” No description of reasons for exclusion‐missing participants.
Selective reporting (reporting bias)	High risk	Not all outcomes of interest were reported.
Other bias	Low risk	**No other potential biases detected**.

Lehman 1999

**Table:** 

**Methods**	**Design**: A cost‐effectiveness based on a published RCT, a health care payer's perspective. Inclusive of direct treatment costs. Currency year was not reported.
**Participants**	**Population**: Homeless persons with severe and persistent mental illness in Baltimore, Maryland
**Interventions**	**Intervention**: ACT
	**Comparator**: Usual community services available in Baltimore
**Outcomes**	**Cost/cost‐effectiveness**:
	Mean ACT cost per case was $8,244.
	The overall average cost per ACT patient was $15,732 less than the cost per usual‐care patient.
	The median total cost per case for the ACT patients was $26,193 compared with $33, 827 for the usual care patients. However, the total per case cost did not reach statistical significance.
	ACT led to lower costs and more day housed than usual care. The cost‐effectiveness ratios were $241 per day housed for the ACT patients compared with $415 per day housed for the usual care patients. In other words, each day of stable housing was achieved for $174 less in direct treatment costs by the ACT program than by usual care, a relative efficiency.
**Notes**	

Risk of bias table

**Table:** 

Bias	Authors' judgement	Support for judgement
Random sequence generation (selection bias)	Unclear risk	
Allocation concealment (selection bias)	Unclear risk	
Blinding of participants and personnel (performance bias)	Unclear risk	
Blinding of outcome assessment (detection bias)	Unclear risk	
Incomplete outcome data (attrition bias)	Unclear risk	
Selective reporting (reporting bias)	Unclear risk	
Other bias	Unclear risk	

Lenz‐Rashid, 2017

**Table:** 

**Methods**	**Design**: A cost analysis based on a cross‐sectional, descriptive before‐ and‐after study
**Participants**	**Population**: Children who had resided in Cottage Housing Serna Village (CA, USA) supportive housing programme with one or more of their parents sometime between 2002 and 2009 in Northern California
**Interventions**	**Intervention**:
	Cottage Housing supportive housing placement
	**Comparator**:
	N/A
**Outcomes**	**Cost or cost‐effectiveness**: The total child welfare costs for all families after clients graduated or exited the Cottage Housing Incorporated programme decreased by $1,017,630 compared with before they entered the programme ($295,632 vs $1,313,262)
**Notes**	

Risk of bias table

**Table:** 

Bias	Authors' judgement	Support for judgement
Random sequence generation (selection bias)	Unclear risk	
Allocation concealment (selection bias)	Unclear risk	
Blinding of participants and personnel (performance bias)	Unclear risk	
Blinding of outcome assessment (detection bias)	Unclear risk	
Incomplete outcome data (attrition bias)	Unclear risk	
Selective reporting (reporting bias)	Unclear risk	
Other bias	Unclear risk	

Lim et al., 2018

**Table:** 

**Methods**	**Design**: Cost analysis from the perspective of government (Medicaid)
**Participants**	**Population**: Adults with serious mental illness and chronic homelessness or dual diagnoses of mental illness and substance use
**Interventions**	**Intervention**:
	Placed and unplaced individuals to the New York City supportive housing programme (housing placement not contingent on adhering to treatment or services)
	**Comparator**:
	NA
**Outcomes**	**Cost or cost‐effectiveness**: The housing programme was associated with total Medicaid cost savings (−$9,526 [95% CI −$19,038 to –2,003])
**Notes**	

Risk of bias table

**Table:** 

Bias	Authors' judgement	Support for judgement
Random sequence generation (selection bias)	Unclear risk	
Allocation concealment (selection bias)	Unclear risk	
Blinding of participants and personnel (performance bias)	Unclear risk	
Blinding of outcome assessment (detection bias)	Unclear risk	
Incomplete outcome data (attrition bias)	Unclear risk	
Selective reporting (reporting bias)	Unclear risk	
Other bias	Unclear risk	

Lipton et al., 1988

**Table:** 

**Methods**	**Design**: Randomized control trial
	**Setting**: New York City, United States
	**Follow up**: 12 months
	**Recruitment**: Recruitment occurred in the emergency room of the Bellevue Hospital
	**Randomization**: Randomization procedures were not mentioned
	**Allocation**: Allocation procedures were not mentioned
	**Blinding**: Not mentioned
	**Timing of outcome assessment**: At index admission and discharge and every 4 months for a year
	**Outcome assessor**: Mental health professionals who were trained on all study instruments
**Participants**	**Population**: Patients presenting to the Bellevue Hospital (New York, NY) psychiatric emergency service who were homeless, chronic mentally ill, and in need of inpatient psychiatric treatment.
	**Sample size**: Total n = 52, Intervention n = 26, TAU n = 26
**Interventions**	**Intervention**:
	The programme provided a furnished room, and offered individualised case management, coordination of public assistance or social security benefits, medication monitoring, money management, meals, activity therapy, and, when appropriate, referrals to psychosocial and rehabilitation programmes.
	**Comparator**:
	Control subjects received routine discharge planning.
**Outcomes**	Housing stability, mental health, hospital admission
**Notes**	

Risk of bias table

**Table:** 

Bias	Authors' judgement	Support for judgement
Random sequence generation (selection bias)	Unclear risk	No description of random sequence generation provided.
Allocation concealment (selection bias)	Unclear risk	No description of allocation concealment provided.
Blinding of participants and personnel (performance bias)	High risk	Blinding of participants and personnel was not possible was not possible due to the nature of the intervention and study design.
Blinding of outcome assessment (detection bias)	Unclear risk	No description of blinding of outcome assessment was provided.
Incomplete outcome data (attrition bias)	High risk	One of the study's limitations was the case attrition, particularly among the control subjects.
Selective reporting (reporting bias)	Low risk	No evidence of reporting outcomes selectively.
Other bias	High risk	Another unanticipated concern resulted from the extent of subjects' unreliable reporting on use of medication and social and psychiatric services, and on activities of daily living.

Malte et al., 2017

**Table:** 

**Methods**	**Design**: Randomized control trial
	**Setting**: Seattle, TX, United States
	**Follow up**: 12 months
**Participants**	**Population**: Participants were homeless veterans enrolled in addictions treatment, predominantly male and unmarried
	**Sample size**: Total sample size: 181, ICM n = 91, HSG control n = 90
**Interventions**	**Intervention**:
	ICM: Caseload was 20. Case management provided (a) support in obtaining/maintaining housing through education about resources, coordination with VA and community housing program providers, assistance in establishing housing program eligibility, and problem solving around threats to housing stability; (b) support for SUD and related issues that affect housing status through treatment engagement/re‐engagement, referrals for needed services (e.g., psychiatric, medical, vocational), and addressing substance use issues proactively; and (c) promotion of residential stability through life skills training
	**Comparator**:
	HSG (control): drop‐in housing support group held weekly in the Addiction Treatment Centre. The group focused on gaining support from fellow study participants and learning from those who successfully obtained housing. Group facilitators provided education about housing resources and assistance with housing‐related issues.
**Outcomes**	Housing stability, mental health, substance use, hospitalisation
**Notes**	

Risk of bias table

**Table:** 

Bias	Authors' judgement	Support for judgement
Random sequence generation (selection bias)	Low risk	“Randomization was computer generated”
Allocation concealment (selection bias)	Low risk	“REsearch staff and participants were blinded to assignment until completion of baseline assessment”
Blinding of participants and personnel (performance bias)	High risk	Blinding of participants and personnel was not possible due to the nature of the intervention and study design.
Blinding of outcome assessment (detection bias)	High risk	“Research staff members completing study assessments were not blinded to study condition after baseline, which may have affected data collection”.
Incomplete outcome data (attrition bias)	Low risk	No evidence of incomplete outcome data was detected.
Selective reporting (reporting bias)	Low risk	No evidence of reporting outcome selectively was detected.
Other bias	High risk	“Neither specific housing placements available to participants nor SUD treatment received were controlled within or across conditions and likely changed during the course of the study, which may have affected outcomes”.

Mares & Rosenheck, 2011

**Table:** 

**Methods**	**Design**: Cost analysis based on a prospective cohort study
**Participants**	**Population**: Chronically homelessness individuals
**Interventions**	**Intervention**:
	Comprehensive housing and health‐ care services through the federal Collaborative Initiative on Chronic Homelessness programme
	**Comparator**:
	Usual care
**Outcomes**	**Cost or cost‐effectiveness**: Collaborative Initiative on Chronic Homelessness participants incurred higher total health‐care costs than the usual care group (US$4,544 vs $3,325; p<0.001)
**Notes**	

Risk of bias table

**Table:** 

Bias	Authors' judgement	Support for judgement
Random sequence generation (selection bias)	Unclear risk	
Allocation concealment (selection bias)	Unclear risk	
Blinding of participants and personnel (performance bias)	Unclear risk	
Blinding of outcome assessment (detection bias)	Unclear risk	
Incomplete outcome data (attrition bias)	Unclear risk	
Selective reporting (reporting bias)	Unclear risk	
Other bias	Unclear risk	

Marshall et al., 1995

**Table:** 

**Methods**	**Design**: Randomized control trial
	**Setting**: Oxford, United Kingdom
	**Follow up**: 14 months
**Participants**	**Population**: Homeless individuals with severe and persistent psychiatric disorders
	**Sample size**: Total sample n = 80, Intervention n = 40, Control n = 40
**Interventions**	**Intervention**:
	Case‐managers chose how much time to offer each subject. Each client was offered an assessment of need from a case‐manager, a discussion of the findings of this assessment with the subject's career, intervention from the case manager to meet needs that were identified, monitoring of the subject's progress by the case‐manager and further assistance should needs arise. Case‐managers were free to choose how far they would personally assist the subject with transport, counselling, organisation of activity programmes, assistance with completion of forms, crisis intervention, help with finding accommodation, assistance with benefits, finding work or places on training courses, and help with obtaining furnishings and domestic appliances.
	**Comparator**:
	Those randomized to the control group continued to receive any assistance that they had been receiving before the study
**Outcomes**	Housing stability, mental health, quality of life, hospitalisation, Employment
**Notes**	

Risk of bias table

**Table:** 

Bias	Authors' judgement	Support for judgement
Random sequence generation (selection bias)	Low risk	“Randomization was by permuted block”
Allocation concealment (selection bias)	Unclear risk	Randomization by sealed envelopes, not clear if opaque.
Blinding of participants and personnel (performance bias)	High risk	Blinding of participants and personnel was not possible due to the nature of the intervention and study design.
Blinding of outcome assessment (detection bias)	Unclear risk	No description of blinding of outcome assessment was provided.
Incomplete outcome data (attrition bias)	Unclear risk	Insufficient reporting of attrition/exclusion (no reasons for missing data provided). Lost to follow‐up reported at 7 months, not at 14 months. Reasons for attrition reported only for the experimental sample.
Selective reporting (reporting bias)	Low risk	No evidence of reporting outcome selectively was detected.
Other bias	Low risk	No evidence of other biases was detected.

Martinez & Burt, 2006

**Table:** 

**Methods**	**Design**: Controlled Before and After Study
	**Setting**: San Francisco, United States
	**Follow up**: 4 years
	**Recruitment**: Outreach workers went to local shelters, street sites, and food lines to enrol eligible homeless individuals
	**Randomization**: A computer program to randomly assign each person a waiting list number and then award the subsidies to those with numbers at the top of the list.
	**Allocation**: Not mentioned
	**Blinding**: Not mentioned
	**Timing of outcome assessment**: 6‐ and 12‐ month increments 12 and 24 months before receipt of housing and 12 and 24 after
	**Outcome assessor**: Researchers. No more details available
**Participants**	**Population**: Formerly homeless single adults with disabilities
	**Sample size**: Total n= 236
**Interventions**	**Intervention**:
	Two supportive housing programmes, both buildings house residents in single‐room‐occupancy units and couple rent subsidies with an array of on‐site services provided by a local interagency collaborative, including case management, psychiatric care, health care, and vocational training. Service receipt is voluntary and abstinence from drug or alcohol use is not a requirement of residency
	**Comparator**:
	This is a controlled before‐and‐after study. Outcomes are compared 24 months prior to providing the intervention and 24 months after.
**Outcomes**	Hospital admission
**Notes**	

Risk of bias table

**Table:** 

Bias	Authors' judgement	Support for judgement
Random sequence generation (selection bias)	High risk	The original study design is a controlled before and after trial without randomization.
Allocation concealment (selection bias)	High risk	The original study design is a controlled before and after trial without allocation concealment
Blinding of participants and personnel (performance bias)	High risk	Blinding of participants and personnel was not possible due to the nature of the intervention and study design
Blinding of outcome assessment (detection bias)	Unclear risk	No description of blinding of outcome assessment was provided
Incomplete outcome data (attrition bias)	Unclear risk	No description of incomplete outcome data was provided,
Selective reporting (reporting bias)	Low risk	No evidence of selective outcome reporting was detected
Other bias	Low risk	No other potential biases were detected.

McHugo et al., 2004

**Table:** 

**Methods**	**Design**: Randomized control trial
	**Setting**: Washington DC, United States
	**Follow up**: 18 months
	**Recruitment**: Two social workers joined the existing outreach teams within the District to identify and assess eligible consumers
	**Randomization**: Participants were randomized to conditions and random assignment was stratified by the presence or absence of substance use disorder.
	**Allocation**: Allocation procedures were not mentioned
	**Blinding**: Not mentioned
	**Timing of outcome assessment**: At baseline and 3,6,9,12,15, and 18 month follow‐up.
	**Outcome assessor**: The investigators. No further details available
**Participants**	**Population**: Homeless adults with severe mental illness
	**Sample size**: Total n = 125, Integrated housing n = 63, Parallel housing n = 62
**Interventions**	**Intervention**:
	Integrated housing programme (case management and housing services provided by teams within a single agency); additional comprehensive mental health services were provided through intensive case management and housing services through dedicated teams that controlled a variety of housing settings; agency did not adhere to the scattered‐site model and congregate settings were considered appropriate for some individuals.
	**Comparator**:
	Parallel housing condition: case management services were provided by mobile assertive community treatment teams and housing by routine community‐based landlords. Mental health services were provided by assertive community treatment (ACT) teams from three community mental health agencies, and housing services were provided by community‐based realtors and landlords. The ACT teams assisted clients in finding and affording housing, but the teams had no control over housing stock.
**Outcomes**	Housing stability, mental health, quality of life, substance use
**Notes**	

Risk of bias table

**Table:** 

Bias	Authors' judgement	Support for judgement
Random sequence generation (selection bias)	Unclear risk	No description of random sequence generation provided, but they mention that random assignment was stratified by the presence or absence of substance use disorder
Allocation concealment (selection bias)	Unclear risk	No description of allocation concealment provided.
Blinding of participants and personnel (performance bias)	High risk	Blinding of participants and personnel was not possible due to the nature of the intervention and study design.
Blinding of outcome assessment (detection bias)	Unclear risk	No description of blinding of outcome assessment provided.
Incomplete outcome data (attrition bias)	High risk	They lost 11.5% of the integrated housing and 20% of the parallel housing service groups, and the dropouts were significantly different in having spent more months homeless during their lifetime than completers.
Selective reporting (reporting bias)	Low risk	No evidence of reporting outcomes selectively detected.
Other bias	Low risk	No other potential biases detected.

Morse et al., 1992

**Table:** 

**Methods**	**Design**: 3‐arm Randomized control trial
	**Setting**: St. Louis, MO, United States
	**Follow up**: 12 months
**Participants**	**Population**: Homeless people with serious psychiatric disorders
	**Sample size**: Total sample n = 178, Treatment team n = 52, Drop‐in n = 62, Outpatient n = 64
**Interventions**	**Intervention**:
	Continuous treatment team: included a “no‐reject” policy, provision of community‐based services for an unlimited time, and a flexible, individualized approach to address clients' multiple needs. Clinical case managers to work intensively with clients, in a ratio of one staff member for every ten clients. In addition to outreach, service activities were targeted to three areas‐individual change, environmental change, and support for bridging the gap between clients' needs and environmental resources and demands.
	Drop‐in centre: Centres provided homeless people with respite from life on the street during the daytime, when the emergency shelters were closed, and offered food, clothing, showers, and some recreational opportunities such as card playing. Social workers were available to refer clients to social services; client‐to‐staff ratio was about 40 to 1.
	**Comparator**:
	Outpatient treatment: Traditional outpatient treatment was provided at a mental health clinic operated by the Missouri Department of Mental Health. The program offered psychotherapy, psychiatric medication, and assistance in obtaining social services.
**Outcomes**	Housing stability, mental health, substance use, income
**Notes**	

Risk of bias table

**Table:** 

Bias	Authors' judgement	Support for judgement
Random sequence generation (selection bias)	Unclear risk	Participants were randomized to treatment conditions. However, no further description of random sequence generation was provided.
Allocation concealment (selection bias)	Unclear risk	No description of allocation concealment was provided.
Blinding of participants and personnel (performance bias)	High risk	Blinding of participants and personnel was not possible due to the nature of the intervention and study design.
Blinding of outcome assessment (detection bias)	Unclear risk	No description of blinding of outcome assessment was provided.
Incomplete outcome data (attrition bias)	Low risk	“There were no main effects of attrition or treatment condition on any of the dependent variables, nor were there any significant interactions of attrition and treatment condition”.
Selective reporting (reporting bias)	Low risk	No evidence of reporting outcomes selectively was detected.
Other bias	Low risk	No other potential biases were detected.

Morse et al., 1997

**Table:** 

**Methods**	**Design**: 3‐arm randomized control trial
	**Setting**: St. Louis, MO, United States
	**Follow up**: 18 months
**Participants**	**Population**: Homeless mentally ill individuals
	**Sample size**: Total sample n = 165, ACT and ACT with community workers n = 105, BCM n = 60
**Interventions**	**Intervention**:
	Assertive community treatment: Principles included intensive individualized treatment, responsibility for providing or coordinating all services needed by the client, persistent follow‐up, and in vivo service delivery. No time limit was placed on treatment. The team conducted individual treatment activities, such as building a therapeutic alliance, linking clients with medication services, helping clients cope with symptoms and solve practical problems in daily living. The team also made interventions to improve clients social environment and resources and provided supportive services, such as monitoring medications, providing payee and money management services, and assisting with transportation.
	Assertive Community Treatment with community workers: The approach operated similarly to the ACT only condition with one exception; clients were also assigned a paraprofessional community worker whose role was to assist with activities of daily living and to be available for leisure activities.
	**Comparator**:
	In the broker case management condition, the case manager's role was to develop an individualized service plan for the client, arrange for and purchase mental health and psychosocial services from various service providers, monitor the quality of purchased services and adjust the mix of services based on the client's changing needs.
**Outcomes**	Housing stability, Mental health, Substance use, Income, quality of life, hospitalisation
**Notes**	

Risk of bias table

**Table:** 

Bias	Authors' judgement	Support for judgement
Random sequence generation (selection bias)	Unclear risk	Participants were randomly assigned to treatment conditions. However, Description of random sequence generation was not provided.
Allocation concealment (selection bias)	Unclear risk	No description of allocation concealment was provided.
Blinding of participants and personnel (performance bias)	High risk	Blinding of participants and personnel was not possible due to the nature of the intervention and study design.
Blinding of outcome assessment (detection bias)	Unclear risk	No description of blinding of outcome assessment was provided.
Incomplete outcome data (attrition bias)	Low risk	“The rate of attrition from the study did not significantly differ across the three treatment conditions. Comparison of clients who remained in the study and those who dropped out revealed no significant differences in background characteristics or scores on the dependent variables at baseline”
Selective reporting (reporting bias)	Low risk	No evidence of reporting outcomes selectively was detected.
Other bias	Low risk	No evidence of other biases was detected.

Morse et al., 2006

**Table:** 

**Methods**	**Design**: Randomized control trial and cost‐consequence analysis from a societal perspective
	**Setting**: St. Louis, MO, United States
	**Follow up**: 24 months
**Participants**	**Population**: Homeless clients with severe mental illness and substance use disorder
	**Sample size**: Total sample n = 149, IACT n = 46, ACTO n = 54, SC n = 49, NIACT n = 79 (Morse et al., 2008)
**Interventions**	**Intervention**:
	**Integrated ACT** (and **New Integrated ACT** in Morse et al., 2008) had a substance abuse specialist on staff and provided substance abuse services directly as part of the ACT team. These services included individual substance abuse counseling and bi‐weekly treatment groups
	**ACT only** team was instructed to refer clients to other community providers for outpatient or individual substance abuse services and to 12‐step groups.
	**Comparator**:
	Participants assigned to the **standard care control** condition were shown a list of community agencies that provided mental health and substance abuse treatment. Research staff also provided these participants with current information about openings at the various agencies and provided linkage assistance to help participants access services at these agencies.
**Outcomes**	Housing stability, Mental health, Substance use
	**Cost/Cost‐effectiveness**:
	IACT and control groups had significantly lower total costs than the ACTO condition, but there was no significant difference in total costs between IACT and control groups.
	Clients in the ACTO and IACT were significantly more satisfied with their treatment and had significant more days in stable housing.
**Notes**	

Risk of bias table

**Table:** 

Bias	Authors' judgement	Support for judgement
Random sequence generation (selection bias)	High risk	There was no description of randomization in Morse et al., 2006, and Morse et al., 2008 was a Quasi‐experimental trial with no random sequence generation involved.
Allocation concealment (selection bias)	High risk	There was no description of allocation concealment in Morse et al., 2006, and Morse et al., 2008 was a Quasi‐experimental trial with no allocation concealment involved.
Blinding of participants and personnel (performance bias)	High risk	Blinding of participants and personnel was not possible due to the nature of the intervention and study design.
Blinding of outcome assessment (detection bias)	Unclear risk	No description of blinding of outcome assessment was provided.
Incomplete outcome data (attrition bias)	High risk	Only 149 of the original 196 provided data for Morse et al., 2006. Analyses indicated that the remaining sample differed from the original at the P<.05 level of significance on two variables; the final study sample reported significantly fewer days of alcohol use and significantly more days of stable housing at baseline than individuals who did not remain in the trial. No adequate description of attrition was provided in Morse et al., 2008.
Selective reporting (reporting bias)	Low risk	No evidence of reporting outcomes selectively was detected.
Other bias	Low risk	In Morse et al., 2008, “Clients in the NIACT condition did appear to have more psychiatric symptoms and more serious drug abuse problems at baseline than clients in the other conditions. Although we used statistical adjustment to control for these initial differences, one cannot have the same confidence in our findings as one would have with a randomized experiment”.

Nyamathi et al., 2001

**Table:** 

**Methods**	**Design**: Randomized longitudinal trial
	**Recruitment**: A convenience sample, as well as street outreach activities
	**Setting**: inner‐city of Los Angeles, CA, US
	**Follow‐up**: 6 months
**Participants**	**Population**: Homeless women and their intimate partners
	**Eligibility criteria**: (a) 18 to 50 years of age, (b) homeless, and (c) had an intimate partner willing to participate in the study. Potential participants were excluded if they were incoherent as a result of mental illness or drug use, as determined by the research nurse.
	**Sample size**: Total n=948; peer mentored group n=258, nurse case‐managed program n=360, standard care program n=330
	**Baseline characteristics**: Age Mean (SD): NCM women: 35.8 (8.4), NCM partners: 38.4 (8.4), Peer‐mentored women: 30.0 (7.6), Peer‐mentored partners: 32.5 (8.2), Standard care women: 37.0 (8.5), Standard care partners: 39.3 (9.3). Ethnicity: Percentage of African American women in: NCM group: 65.8%, Peer‐mentored: 41.4%, Standard care: 80.2%. Percentage of Hispanic/Latino women in: NCM group: 21.9%, Peer‐mentored: 46.5%, Standard care: 10.8%. Percentage of Anglo American women in: NCM group: 11.4%,Peer‐mentored: 10.1%, Standard care: 7.2%. More than 70% of the participants in the NCM group resided primarily in homeless shelters, as compared to about 40% in the peer‐mentored group and about 20% in the standard care group. More than half of the standard care group members had resided primarily in conventional housing over the past month. Persons in the NCM group were less likely than others to have resided in either conventional housing or sober‐living shelters.
**Interventions**	**Peer support intervention**: Women and their intimate partners assigned to the peer‐mentored program received the same intervention as those in the nurse case‐managed program, except that the role of the nurse was assumed by a female peer mentor who matched the participants' ethnicity. These individuals had led lifestyles similar to their clients, experiencing such things as homelessness and/or drug and alcohol addiction. Now sober and living in stable home environments, peer mentors were trained extensively by the research team to administer the peer‐mentored program and questionnaires, as well as to facilitate referrals to health and social services.
	**Standard Care**: Participants were administered the instrument packet by the research staff and received a standard traditional 15‐min HIV antibody pretest as well as posttest counselling by the research nurses or outreach workers. HIV pretest counselling included an assessment and discussion of drug and sexual behaviours that place one at risk for HIV/AIDS and an explanation of the meaning of negative and positive HIV antibody test results. HIV posttest counselling reinforced this information, provided the result of the HIV antibody test, and reinforced the meaning of either the negative or positive test result.
**Outcomes**	**Mental health**: Psychological well‐being was measured by the Mental Health Index (MHI‐5). Depression, anxiety, and hostility were measured by the Brief Symptom Inventory (BSI). Self‐esteem was measured using a revised version of the Coopersmith (1967) Self‐Esteem Inventory (SEI).
	**Quality of life**: Life satisfaction was measured by a series of faces with expressions ranging from very happy to very sad.
	**Substance use**: Drug and alcohol use was assessed by a minimally revised Drug History Form.
**Notes**	

Risk of bias table

**Table:** 

Bias	Authors' judgement	Support for judgement
Random sequence generation (selection bias)	Unclear risk	Participants were randomized to conditions. However there was no description of random sequence generation provided.
Allocation concealment (selection bias)	Unclear risk	No description of allocation concealment was provided.
Blinding of participants and personnel (performance bias)	High risk	Blinding of participants and personnel was not possible due to the nature of the intervention and study design.
Blinding of outcome assessment (detection bias)	Unclear risk	No description of blinding of outcome assessment was provided.
Incomplete outcome data (attrition bias)	High risk	“Overall follow‐up rates were highest (78%) in the peer‐mentored program, followed by 67% in the NCM program, and 64% in the standard care program”.
Selective reporting (reporting bias)	Low risk	No evidence of reporting outcomes selectively was detected.
Other bias	Low risk	No other potential biases were detected.

Nyamathi et al., 2016

**Table:** 

**Methods**	**Design**: Randomized clinical trial and cost analysis
	**Recruitment**: from prisons and jails
	**Setting**: Residential drug treatment (RDT) facility contracted by the state and Los Angeles County correctional agencies, Los Angeles, CA, US
	**Follow‐up**: At baseline, 6 months, and 12 months
**Participants**	**Population**: Homeless men recently released from county jails with a history of drug use
	**Eligibility criteria**: (a) had a history of drug use prior to their latest incarceration, (b) were 18–60 years of age, (c) resided in one participating RDT program, and (d) were considered to be homeless prior to discharge from incarceration. Exclusion criteria included not speaking English and being judged to be cognitively impaired by the research staff.
	**Sample size**: Total n=600; PC‐NCM n=195, PC n=196, Usual care n=209.
	**Baseline characteristics**: the sample of 60 parolees reported a mean age of 40 (SD=10.4) and 11.5 years of education. The men were predominantly African American (46%) or Latino (33%) and nearly two thirds were never married; yet 62% reported having children. While all participants were screened as being homeless, 88% were living on the street/halfway houses or someone else's apartment 6 months prior to their most recent incarceration, while 12% were transitioning in residential drug treatment programmes or a prior incarceration within the 6‐month period. In terms of health, one‐third reported that they were of fair or poor health. More than two‐thirds (70%) reported having committed a violent crime. These participants were released from county jails (55%) and state prisons (45%), respectively. The vast majority of these participants (84%) had reported a history of lifetime stimulant use, and 85% had used marijuana. Close to 60% reported having more than one sexual partner in the 6 months immediately prior to their most recent incarceration. No program differences were found in any of the demographic variables.
**Interventions**	**Intermediate peer coaching**:
	Participants received weekly peer coaching interaction. The main foci of each session included building effective coping skills, personal assertiveness, self‐management, therapeutic nonviolent communication (NVC), and self‐esteem building. Further, the sessions were dedicated to avoidance of health‐risk behaviours, increasing access to medical and psychiatric treatment and improving compliance with medications, skill‐building, and personal empowerment. Discussions also centred on strategies to assist in seeking support and assistance from community agencies as parolees prepare for completion of the residential drug treatment program. Integrated throughout, skill building in communication and negotiation and issues of empowerment were highlighted.
	**Usual care**:
	For the usual care (UC) program, participants received all recovery and rehabilitation services available at the RDT site, including substance abuse services, assistance with independent living skills, job skills assistance, literacy, various counselling services, and discharge planning.
**Outcomes**	**Housing stability**: Percentage of patients in different housing settings using a structured questionnaire.
	**Substance use**: Alcohol and drug use was assessed using the modified version of the Texas Christian University (TCU) Drug History form.
	**Employment**: Percentage of patients with full‐time, part‐time employment and percentage of patients who are unemployed using a structured questionnaire.
	**Cost/Cost‐effectiveness**:
	The amount of cash spent on program activities was about the same for all three groups of participants: 32,583 for the PC‐NCM participants (M = $167.09; SD = $79.51), $33,375 for PC (M = $170.28; SD = $76.20), and $33,293 for UC (M = 159.30; SD = $76.61).
	The PC‐NCM group consumed the most staff time (more than half or 54% of the total recorded staff time), followed by PC group with about 44% of the staff time, while the UC group used the least staff time, with only 2.11% of the staff time.
	On an annualized basis, participants of the PC‐NCM group on average consumed $593.26; participants in the PC group on average consumed $488.92; and participants of the UC group consumed $59.92.
**Notes**	

Risk of bias table

**Table:** 

Bias	Authors' judgement	Support for judgement
Random sequence generation (selection bias)	Low risk	“Prior to the baseline questionnaire, Urn randomization was utilised. The program allows researchers to randomize study subjects to two or three randomization groups while balancing on 2‐20 variables”
Allocation concealment (selection bias)	Low risk	The use of Urn randomization; an on‐site computer based randomization scheme ensures allocation was concealed at baseline.
Blinding of participants and personnel (performance bias)	High risk	Blinding of participants and personnel was not possible due to the nature of the intervention and study design.
Blinding of outcome assessment (detection bias)	Unclear risk	No description of blinding of outcome assessment was provided.
Incomplete outcome data (attrition bias)	Low risk	No differential attrition was noticed and the loss of subjects among the three assignment conditions appeared to be at similar rates, all within five percentage points from one another.
Selective reporting (reporting bias)	High risk	“…we opted to present the data from the second follow‐up because of the longer observation period for the purpose of justice‐related outcomes….for health status and employment status, we used only the second 6‐month data to avoid conflicting reports”.
Other bias	Low risk	No other potential biases were found.

Okin et al., 2000

**Table:** 

**Methods**	**Design**: A cost‐benefit analysis based on a prospective, pre‐post study, a hospital's perspective. No statistical approach was used to adjust for confounding factors. Cost data were reported in 1997 US$.
**Participants**	**Population**: Adult who used the ED five times or more in 12 months
**Interventions**	Before and after CM intervention
**Outcomes**	**Cost/Cost‐Effectiveness**:
	The median total hospital service cost decreased from $21,022 in the year before case management enrolment to $14,910 in the year after enrolment (median change = $22,406, P = .06, 95% CI: −$6,361 to −$430).
	When the total cost of case management services to the 53 patients, calculated at $296,738, was subtracted from the $429,464 savings realised in other hospital services, there was a net cost saving of 132,726, indicating that for each dollar invested in the case management program, there was a $1.44 reduction in other hospital costs.
**Notes**	

Risk of bias table

**Table:** 

Bias	Authors' judgement	Support for judgement
Random sequence generation (selection bias)	Unclear risk	
Allocation concealment (selection bias)	Unclear risk	
Blinding of participants and personnel (performance bias)	Unclear risk	
Blinding of outcome assessment (detection bias)	Unclear risk	
Incomplete outcome data (attrition bias)	Unclear risk	
Selective reporting (reporting bias)	Unclear risk	
Other bias	Unclear risk	

Orwin et al., 1994

**Table:** 

**Methods**	**Design**: 3‐ arm randomized control trial
	**Setting**: Minneapolis, United States
	**Follow up**: 24 months
**Participants**	**Population**: Homeless persons with alcohol or other drug use problems
	**Sample size**: Total sample n = 260, ICM n = 82, Intermediate CM n = 117 control n = 61
**Interventions**	**Intervention**:
	Intensive Case Management: The intensive case managers were to focus more on outreach and field work, maintaining closer and more frequent contact with clients and in a variety of settings. The intensive group was designed on a modified team model that periodically redistributed clients among the team so that team members became familiar with each other's clients
	**Comparator**:
	Intermediate Case Management: Intermediate case managers were expected to be office based, although their goals also included outreach. Managers were not expected to develop close relationships with the clients. The majority of their time was spent on practical issues, such as assisting with entitlement procurement and establishing representative payee arrangements.
	Control group (Episodic or Usual care): This group received only episodic case management services‐ the services normally available through the county.
**Outcomes**	Housing stability, mental health, substance use, employment
**Notes**	

Risk of bias table

**Table:** 

Bias	Authors' judgement	Support for judgement
Random sequence generation (selection bias)	Unclear risk	No description of random sequence generation was provided.
Allocation concealment (selection bias)	Unclear risk	No description of allocation concealment was provided.
Blinding of participants and personnel (performance bias)	High risk	Blinding of participants and personnel was not possible due to the nature of the intervention and study design.
Blinding of outcome assessment (detection bias)	High risk	“In Minneapolis, all interviews were conducted by case managers”
Incomplete outcome data (attrition bias)	High risk	“… attrition was actually higher in the treatment group (57%) than in the comparison group (32%)”
Selective reporting (reporting bias)	Low risk	No evidence of reporting outcomes selectively was detected.
Other bias	High risk	Randomization occurred prior to screening.
		Also, “…baseline nonequivalence analyses suggested that the assignment process resulted in the treatment group being demonstrably worse off at baseline than the comparison group”

Pankratz et al., 2017

**Table:** 

**Methods**	**Design**: Quasi‐experimental trial
	**Setting**: Waterloo, Ontario, Canada
	**Follow up**: 6 months
	**Recruitment**: Homeless individuals were surveyed using the Vulnerability Index‐Service Prioritization Decision Assistance Prescreen Tool (VI‐SPDAT)
	**Randomization**: No randomization was performed
	**Allocation**: Decisions regarding the allocation were made by housing services and the STEP Home team based on vulnerability
	**Blinding**: Not reported in the publication
	**Timing of outcome assessment**: At baseline and 6 months after
	**Outcome assessor**: Three individuals with lived experience of homelessness, mental illness, and substance use as well as a graduate student
**Participants**	**Population**: Individuals experiencing chronic homelessness
	**Sample size**: Total n=60, HAWS n=28, Non‐HAWS n=32
**Interventions**	**Intervention**:
	The housing assistance with support rent assistance pilot provides participants with a CAN$350 to use towards rent.
	**Comparator**:
	The comparison group (non‐HAWS) received STEP Home support only which includes: street outreach, housing liaison support, intensive support, peer support, and informal circle of friends support.
**Outcomes**	Housing stability, quality of life, income
**Notes**	

Risk of bias table

**Table:** 

Bias	Authors' judgement	Support for judgement
Random sequence generation (selection bias)	High risk	Decisions regarding the allocation of the intervention to the eligible participants on the priority list were made by Housing Services and the STEP Hometeam and were informed by VI‐SPDAT data and worker experience with the individuals. The researchers were not involved in the decision‐making about allocation. No random sequence was generated.
Allocation concealment (selection bias)	High risk	The allocation was not done by the researchers and no allocation concealment was addressed in the article.
Blinding of participants and personnel (performance bias)	High risk	The intervention group received both rent assistance plus housing support, while the comparison group received only housing support. Therefore, it was technically difficult to blind participants.
Blinding of outcome assessment (detection bias)	Unclear risk	There was no description addressing blinding of outcome assessment.
Incomplete outcome data (attrition bias)	Low risk	No evidence of incomplete outcome reporting or attrition bias was detected.
Selective reporting (reporting bias)	Low risk	No evidence of reporting outcomes selectively was detected.
Other bias	Low risk	No other potential biases were detected.

Pauley et al., 2016

**Table:** 

**Methods**	**Design**: Cost analysis based on a feasibility, before‐and‐after study
**Participants**	**Population**: All residents of the three participating supportive inner‐city housing facilities who received service in the 16‐month study period
**Interventions**	**Intervention**:
	Inner City Access Programme, which combines supportive housing services and health care for homeless, underhoused, and marginalised populations using the shelter system
	**Comparator**:
	N/A
**Outcomes**	**Cost or cost‐effectiveness**: Participating in the programme was associated with a 60% decrease in average cost per client (US$5,357 vs $2,159)
**Notes**	

Risk of bias table

**Table:** 

Bias	Authors' judgement	Support for judgement
Random sequence generation (selection bias)	Unclear risk	
Allocation concealment (selection bias)	Unclear risk	
Blinding of participants and personnel (performance bias)	Unclear risk	
Blinding of outcome assessment (detection bias)	Unclear risk	
Incomplete outcome data (attrition bias)	Unclear risk	
Selective reporting (reporting bias)	Unclear risk	
Other bias	Unclear risk	

Poremski et al., 2015

**Table:** 

**Methods**	**Design**: Randomized control trial
	**Setting**: Montreal, Canada
	**Follow up**: 8 months
	**Recruitment**: From among the 204 participants of the Montreal site of the At Home/Chez Soi study.
	**Randomization**: Stratified randomization, with blocking within strata was used. Randomization was stratified by ICM team and by past work experience (having worked in the past 5 years, or not).
	**Allocation**: Allocations were supplied in opaque envelopes.
	**Blinding**: Interviewers could not be blinded to group assignment
	**Timing of outcome assessment**: At baseline and every 3 months for an 8 months period of good fidelity
	**Outcome assessor**: Interviewers. No details available
**Participants**	**Population**: Individuals with mental illness who are precariously housed or have been homeless for at least seven nights
	**Sample size**: Total n = 90, Intervention n = 45, control n = 45
**Interventions**	**Intervention**:
	Individual placement and support, which helped participants to obtain and maintain competitive employment of their choice; employment specialists were trained and supervised by a senior member of an experienced local individual placement and support service, and worked closely with the clinical teams.
	**Comparator**:
	Control group: No Individual Placement and Support but participants were free to seek employment by any means of their choice, with some support from their case managers.
**Outcomes**	Housing stability, substance use, hospital admissions, employment, income
**Notes**	

Risk of bias table

**Table:** 

Bias	Authors' judgement	Support for judgement
Random sequence generation (selection bias)	Low risk	Stratified randomization, with blocking within strata, was used to assign participants to either IPS or usual vocational services.
		Randomization was stratified by ICM team and by past work experience (having worked in the past 5 years, or not).
Allocation concealment (selection bias)	Low risk	Allocation concealment was achieved by supplying allocations in opaque envelopes. Group assignment was only revealed after the end of the interview.
Blinding of participants and personnel (performance bias)	High risk	Blinding of participants and personnel was not possible due to the nature of the intervention and study design.
Blinding of outcome assessment (detection bias)	High risk	Due to the nature of the questionnaires used to measure satisfaction with services, interviewers could not be blinded to group assignment.
Incomplete outcome data (attrition bias)	Unclear risk	"Multiple imputation (MI) by chained equations… was used to impute the 10.5% of employment data that were missing (Individuals who died during the study were excluded from the analysis and imputation)… Two sensitivity analyses were conducted. In the first, missing days were treated as days during which the person had not worked" Unclear distribution of missing data between the two groups, and sensitivity analysis only assessed one side of outcome. Numerical outcome of sensitivity analysis not reported.
Selective reporting (reporting bias)	Low risk	No evidence if reporting outcomes selectively was provided.
Other bias	Unclear risk	Unclear whether good fidelity was a pre‐specified condition for data inclusion. However, this was partially addressed by the second sensitivity analysis: "the period considered for analysis was extended to the entire study period, from randomization to the last point in the study and the corresponding estimated fidelity rating included as a time‐ dependent covariate”. Possible recruitment bias: "However, as implementation of the intervention progressed, recruitment increased".

Rich & Clark, 2005

**Table:** 

**Methods**	**Design**: Quasi‐experimental trial
	**Setting**: Tampa St. Petersburg, Florida, United States
	**Follow up**: 12 months
	**Recruitment**: Eligible participants were asked to participate by research personnel
	**Randomization**: No randomization was performed
	**Allocation**: Allocation procedures were not mentioned
	**Blinding**: Not mentioned
	**Timing of outcome assessment**: At baseline and 6 and 12 months afterwards
	**Outcome assessor**: Not mentioned
**Participants**	**Population**: Homeless persons with severe mental illness
	**Sample size**: Total n = 152, Comprehensive housing program n = 83, Specialized case management n = 69
**Interventions**	**Intervention**:
	Two comprehensive housing programmes with extensive housing services including: guaranteed access to housing; the provision of housing support services; case management; and priority linkages to psychiatric, psychosocial, and vocational services; case management and housing support services were maintained as long as the consumer had a need, and for most participants extended throughout the project.
	**Comparator**:
	Specialized Case management: The activities of this “blended” case management program include active outreach and engagement, some on‐site counselling, medication and medication management, vouchers and assistance with obtaining housing and linkages for other psychiatric, substance abuse and other psychosocial services. All clients were eligible for housing vouchers for short‐term rent and deposit support
**Outcomes**	Housing stability, mental health, quality of life, substance use, income
**Notes**	

Risk of bias table

**Table:** 

Bias	Authors' judgement	Support for judgement
Random sequence generation (selection bias)	High risk	This was a quasi‐experimental study without randomization.
Allocation concealment (selection bias)	High risk	This was a quasi‐experimental study without allocation concealment.
Blinding of participants and personnel (performance bias)	High risk	Blinding of participants and personnel was not possible due to the nature of the intervention and study design.
Blinding of outcome assessment (detection bias)	Unclear risk	No description of blinding of outcome assessment was provided.
Incomplete outcome data (attrition bias)	High risk	Proportion of missing data varies across intervention groups. "The comprehensive housing group had (83% retention) while the specialised case management group had (56% retention). Also, they found a differential drop out rate as a function of type of intervention and gender with regard to the income variable.
Selective reporting (reporting bias)	Low risk	No evidence of reporting outcome selectively was detected.
Other bias	High risk	This study lacked a no treatment control group, findings could have been the result of other confounding influences including the passage of time, regression to the mean or spontaneous remission.

Rosenblum et al., 2002

**Table:** 

**Methods**	**Design**: Quasi‐experimental trial
	**Setting**: New York city, United States
	**Follow up**: 4 months
**Participants**	**Population**: Homeless substance users
	**Sample size**: Total sample n = 250 [Description not provided]
**Interventions**	**Intervention**:
	Experimental subjects are seen by a social worker at the time that they are recruited for the study. The social worker provides intensive case management (ICM), which includes a comprehensive needs assessment, multiple sessions to increase probability of appropriate and completed referrals, and incentives (such as phone cards, public transportation tokens, waist wallets, grooming and sanitary supplies) for service engagement.
	**Comparator**:
	Control subjects could choose to refer themselves to the social worker. They are not given incentives for repeated sessions
**Outcomes**	Housing stability, substance use, hospitalization, income
**Notes**	

Risk of bias table

**Table:** 

Bias	Authors' judgement	Support for judgement
Random sequence generation (selection bias)	High risk	The study sample was divided into experimental and control groups without random sequence generation.
Allocation concealment (selection bias)	High risk	The study sample was divided into experimental and control groups without allocation concealment.
Blinding of participants and personnel (performance bias)	High risk	Blinding of participants and personnel was not possible due to the nature of the intervention and study design.
Blinding of outcome assessment (detection bias)	Unclear risk	No description of blinding of outcome assessment was provided.
Incomplete outcome data (attrition bias)	High risk	Study not complete at the time of publication. Of the 250 subjects inducted into the study, the N that is reported on is 184 due to the availability of results with no further explanation.
Selective reporting (reporting bias)	Low risk	No evidence of reporting outcome selectively was detected.
Other bias	Low risk	No other potential biases were detected.

Rosenheck et al., 2003

**Table:** 

**Methods**	**Design**: 3‐arm randomized control trial and cost‐effective analysis
	**Setting**: San Fransisco and San Deigo (CA), New Orleans (LA), Cleveland (OH), USA
	**Follow up**: 3 years
	**Recruitment**: Through outreach assessment from a 43‐site program
	**Randomization**: Through a telephone call to the central evaluation staff, who identified the next assignment from a deck of cards specific to each site.
	**Allocation**: Not reported in the publication
	**Blinding**: Neither veterans nor staff could be masked to group assignment.
	**Timing of outcome assessment**: At baseline and 6,12,18,24, and 36 months
	**Outcome assessor**: Trained evaluation assistants
**Participants**	**Population**: Veterans who are homeless (live in a homeless shelter or on the streets), or had been homeless for 1 month or longer, with a diagnosis of a major psychiatric disorder or alcohol or drug disorder
	**Sample size**: Total n = 460, Intervention (HUD‐VASH) n = 182, CM only n = 90, Control (Usual care) n = 188
**Interventions**	**Intervention**:
	Case management and priority access to housing vouchers; housing vouchers were administered by local housing authorities, and case managers facilitated access and use of the voucher, and supported transitions to independent living; the case management model used was modified from the assertive community treatment and encouraged at least weekly face‐to‐face contact, community‐based service delivery, and more intensive involvement in crisis situations.
	**Comparator**:
	Case management‐only group: case managers were to provide the same intensity of the services in the HUD‐VASH condition and used whatever housing resources could be obtained (no voucher).
	Control group: The standard care condition consisted of case management provided by Health Care for Homeless Veterans program outreach workers (short‐term broker model of CM).
**Outcomes**	Housing stability, mental health, quality of life, substance use, employment, income
	**Cost or cost‐effectiveness**: From the perspective of the health‐care system, Veterans Affairs health costs for HUD‐VASH participants were 18% higher (US$6,962) than those in the standard care group; from a societal perspective, HUD‐VASH clients consumed 15% ($6,200) more resources than standard care clients; each additional day housed among HUD‐ VASH clients cost $58 (95% CI 4 to 111) from the perspective of Veterans Affairs, $50 (–17 to 117) from the perspective of the health‐care system, and $45 (–19 to –108) from a societal perspective
**Notes**	

Risk of bias table

**Table:** 

Bias	Authors' judgement	Support for judgement
Random sequence generation (selection bias)	Unclear risk	Participants were randomly assigned to the study arm through a telephone call to the central evaluation staff who identified the next assignment from a deck of cards specific to each site.
Allocation concealment (selection bias)	Unclear risk	Description of allocation concealment not provided.
Blinding of participants and personnel (performance bias)	High risk	Blinding of participants and personnel was not possible due to the nature of the intervention and study design
Blinding of outcome assessment (detection bias)	Unclear risk	Description of blinding of outcome assessment not provided.
Incomplete outcome data (attrition bias)	High risk	Comparison across groups showed significant differences in follow‐up rates within each assessment period from the 6‐month through the 3‐year assessment,with higher follow‐up rates in the voucher plus case management group than in the case management only group; follow‐up rates were even lower in the standard care group.
Selective reporting (reporting bias)	Low risk	No evidence of reporting outcomes selectively was detected.
Other bias	Low risk	No other risk of biases identified.

Sadowski et al., 2009

**Table:** 

**Methods**	**Design**: Randomized control trial and cost analysis
	**Setting**: Chicago, United States
	**Follow up**: up to 18 months
	**Recruitment**: Hospital social workers referred any in‐patient who did not have housing to the study team
	**Randomization**: Randomization was stratified by study hospital using a random‐numbers table
	**Allocation**: a 1:1 concealed allocation to the conditions using numbered envelopes.
	**Blinding**: All clinicians caring for study participants were blinded to study group assignment
	**Timing of outcome assessment**: At baseline and 18 months
	**Outcome assessor**: The investigators
**Participants**	**Population**: Chronically ill homeless adults. Adult participants who were fluent in English or Spanish, without stable housing during the 30 days before admission to hospital, with no child dependents, who had at least one of 15 chronic medical illnesses documented in the medical record.
	**Sample size**: Total n = 407, Intervention n = 201, TAU n = 206
**Interventions**	**Intervention**:
	Housing and case management intervention based on the Housing First model. This intervention was developed by a consortium of 14 hospitals, respite care centres, and housing agencies in Chicago, which had three integrated components: provision of transitional housing at respite care centres, subsequent placement in stable housing, and case management, which was provided on‐site at primary study sites, respite care facilities, and stable housing sites; n = 201
	**Comparator**:
	Usual care: Participants were referred back to the original hospital social worker and received the usual discharge planning services with no continued relationship after hospital discharge. Typically patients would be provided transportation to an overnight shelter if no other accommodation could be arranged before discharge.
**Outcomes**	Housing stability, hospital admission
	**Cost or cost‐effectiveness**: Housing and case management intervention was associated with lower total cost than usual care (–US$6,307 [95% CI –16,616 to 4,002]; p=0.23)
**Notes**	

Risk of bias table

**Table:** 

Bias	Authors' judgement	Support for judgement
Random sequence generation (selection bias)	Low risk	Using a random‐numbers table, one investigator (L.S.S.) placed each group assignment into a sealed opaque envelope and stored it until needed for enrolment.
		Participants were randomized in a 1:1 allocation to the intervention group or the usual care group using numbered envelopes opened by the participant after the baseline interview.
Allocation concealment (selection bias)	Low risk	All clinicians caring for the study participants on the wards and in the Emergency departments and clinics were blinded to study group assignment.
Blinding of participants and personnel (performance bias)	High risk	Blinding of participants and personnel was not possible due to the nature of the intervention and study design.
Blinding of outcome assessment (detection bias)	Low risk	Blinded collection of study data: Research personnel who collected outcome data from medical records were blinded to study group assignment.
Incomplete outcome data (attrition bias)	Low risk	No evidence of incomplete outcome data reporting.
Selective reporting (reporting bias)	Low risk	No evidence of reporting outcomes selectively.
Other bias	High risk	They lacked electronic access to medical records of the other study hospitals, So they had to rely on participants interviews to identify these outcomes, which probably biased results for all hospitals against the intervention group.

Schinka et al., 1998

**Table:** 

**Methods**	**Design**: Cost analysis based on a pseudorandomised experimental study
**Participants**	**Population**: Men with moderate to severe substance dependence with consecutive voluntary admissions to the substance abuse treatment programme of a metropolitan Veterans Affairs hospital (undisclosed location)
**Interventions**	**Intervention**:
	Supportive housing
	**Comparator**:
	Inpatient treatment
**Outcomes**	**Cost or cost‐effectiveness**: The weekly per‐patient cost for inpatient treatment was US$1,674 ($719 for personnel costs and $955 for housing costs); for the supportive housing patients, the weekly cost was $899 ($624 for personnel costs and $275 for housing costs); the cost‐saving was $775; the mean costs of a successful treatment were $9,524 and $4,291 for the inpatient and supportive housing groups, respectively
**Notes**	

Risk of bias table

**Table:** 

Bias	Authors' judgement	Support for judgement
Random sequence generation (selection bias)	Unclear risk	
Allocation concealment (selection bias)	Unclear risk	
Blinding of participants and personnel (performance bias)	Unclear risk	
Blinding of outcome assessment (detection bias)	Unclear risk	
Incomplete outcome data (attrition bias)	Unclear risk	
Selective reporting (reporting bias)	Unclear risk	
Other bias	Unclear risk	

Shern et al., 2000

**Table:** 

**Methods**	**Design**: Randomized control trial
	**Setting**: Manhattan, New York, United States
	**Follow up**: 24 months
**Participants**	**Population**: Street‐dwelling individuals with psychiatric disabilities
	**Sample size**: Total sample n = 168, Intervention n = 91, control n = 77
**Interventions**	**Intervention**:
	Intervention participants had access to the Choices program which included: 1. Outreach and engagement designed to foster the development of rudimentary relationships between Choices staff and homeless individuals 2. Invitation to attend and join the Choices centre, where resources (showers, food) were available for experimental study participants 1am‐7pm daily. Participation in structured group activities was not required, but assistance was available to anyone requesting help in obtaining health, mental health, dental and social services and in developing and implementing individual rehabilitation plans. Additionally, the centre provided an opportunity for members to meet new friends and socialize. 3. Respite housing in 10 bed, informal church‐based shelters or in blocks of YMCA rooms rented by the program and overseen by program staff. 4. In‐community and on‐site rehabilitation services to assist individuals in finding and maintaining community‐based housing. The Choices program structure was similar to an ICM program with a 13:1 client to case manager ratio.
	**Comparator**:
	Control participants received information about "standard treatment" ‐ that is the existing array of homelessness and speciality mental health services in New York City.
**Outcomes**	Housing stability, mental health, quality of life
**Notes**	

Risk of bias table

**Table:** 

Bias	Authors' judgement	Support for judgement
Random sequence generation (selection bias)	Unclear risk	No description of random sequence generation was provided.
Allocation concealment (selection bias)	Unclear risk	No description of allocation concealment was provided.
Blinding of participants and personnel (performance bias)	High risk	Blinding of participants and personnel was not possible due to the nature of the intervention and study design.
Blinding of outcome assessment (detection bias)	Unclear risk	No description of blinding of outcome assessment was provided.
Incomplete outcome data (attrition bias)	Low risk	There were differences due to missing informations on different subjects but adjustments were made to minimize bias and keep summary statistic approach fairly representing study findings.
Selective reporting (reporting bias)	Low risk	No evidence of reporting outcome selectively was detected.
Other bias	Low risk	No other potential biases were detected.

Shinn et al., 2015

**Table:** 

**Methods**	**Design**: Randomized control trial
	**Setting**: Westchester County, New York, United States
	**Follow up**: 24 months
**Participants**	**Population**: Shelter‐dwelling families with child. A mother diagnosed with mental illness or substance use
	**Sample size**: Total sample n = 200, Intervention n = 97, Usual care n = 103
**Interventions**	**Intervention**:
	Family Critical Time Intervention (FCTI): A community‐based service model for families using homeless shelters. Multidisciplinary teams help connect the family with social services and form supportive relationships with families and friends. This FCTI targets the critical time of transition from the shelter to housing in the community through three phases:
	Transition to Community Phase: A family arrives in the shelter and is assessed thoroughly by a case manager, who then works intensely with the mother, up to three times per week. Try‐Out Phase: The case manager reduces contact during this phase, but still supports the family through adjusting the support systems as they move into the community. Transfer to Care: This phase involves long‐term linkage with community‐based services to allow the family to take full responsibility for accessing services.
	**Comparator**:
	Case management with a caseload of 24 families or more. The families, after meeting caseworker standards for housing readiness, had access to scattered site subsidized housing.
**Outcomes**	Housing stability, Mental health
**Notes**	

Risk of bias table

**Table:** 

Bias	Authors' judgement	Support for judgement
Random sequence generation (selection bias)	Low risk	Families were randomly assigned to intervention or control at a 1:1 ratio following screening for inclusion into study. Also, a random number table was used to stratify families by size.
Allocation concealment (selection bias)	Low risk	The enrolment coordinator called the main research office and was given a randomly chosen group assignment for the family.
Blinding of participants and personnel (performance bias)	High risk	Blinding of participants and personnel was not possible due to the nature of the intervention and study design.
Blinding of outcome assessment (detection bias)	Low risk	“Teachers were blind to the purpose of the study and to group assignment”
Incomplete outcome data (attrition bias)	High risk	“Baseline data were available for 198 mothers, of which 145 (73%) completed three or four interviews after baseline, 31 (16%) completed two, and 22 (11%) completed one post baseline interview”. However, no further description of attrition was provided.
Selective reporting (reporting bias)	Low risk	No evidence of reporting outcomes selectively was detected.
Other bias	High risk	“The absence of a true baseline assessment of maternal mental health precludes the ability to rule out alternative explanations for improvements in maternal mental health beyond rehousing”.
		Also, “… its reliance of mother self report if outcomes and services. Using a single reporter introduces measurement error that can mask true effects”.

Shumway et al., 2008

**Table:** 

**Methods**	**Design**: Randomized control trial and cost‐effectiveness analysis
	**Setting**: San Francisco, United States
	**Follow up**: 24 months
**Participants**	**Population**: Homeless or vulnerably housed frequent emergency department users with psychological problems
	**Sample size**: Total sample n = 252, Case management n = 167, Usual care n = 85
**Interventions**	**Intervention**:
	Patients randomized to case management received long‐term clinical case management that included assessment, crisis intervention, individual and group supportive therapy, assistance in obtaining stable housing and income entitlements, linkage to medical care providers, referral to substance abuse services when needed, and ongoing assertive community outreach to maintain continuity of care with a maximum caseload of 15 patients.
	**Comparator**:
	Patients randomized to usual care were eligible to receive case management services at the conclusion of the 24‐month study period.
**Outcomes**	Housing stability, Mental health, Substance use, Hospitalisation, Income
	**Cost/Cost‐effectiveness**:
	Emergency department costs were significantly lower among CM patients than among usual care patients. The costs of medical inpatient services, psychiatric emergency services, Psychiatric inpatient services, medical outpatient services, and physicians' professional fees did not differ between CM and usual care patients.
	When the costs of the ED Case Management Program were considered, total hospital costs were similar for CM and usual care patients.
**Notes**	

Risk of bias table

**Table:** 

Bias	Authors' judgement	Support for judgement
Random sequence generation (selection bias)	Low risk	Participants were randomized using stratification and unequal probability procedures (2 to 1) to ensure more participants were assigned to the intervention study arm.
Allocation concealment (selection bias)	Unclear risk	No information was provided on whether allocation was concealed during recruitment and study assignment.
Blinding of participants and personnel (performance bias)	High risk	Due to the nature of the intervention and population, blinding of participants and personnel was not possible.
Blinding of outcome assessment (detection bias)	Unclear risk	No information was provided on whether outcome assessors were blinded to study assignment.
Incomplete outcome data (attrition bias)	Low risk	Attrition was minimal and addressed properly.
Selective reporting (reporting bias)	Low risk	No evidence of selective outcome reporting was detected.
Other bias	Low risk	No evidence of other biases was detected.

Siegel et al., 2006

**Table:** 

**Methods**	**Design**: Quasi‐experimental trial
	**Setting**: New York City, United States
	**Follow up**: 18 months
	**Recruitment**: Tenants were referred to housing form hospitals, shelters, the streets, and clinics
	**Randomization**: No randomization was performed
	**Allocation**: No allocation procedures were performed
	**Blinding**: Not mentioned
	**Timing of outcome assessment**: At baseline, and 6‐,12‐, 18‐ months afterwards
	**Outcome assessor**: Not mentioned
**Participants**	**Population**: Homeless individuals with severe mental illness
	**Sample size**: Total n=157, Supported housing n=75, Community residences n=82
**Interventions**	**Intervention**:
	Tenants mostly living alone, resided in studio or one‐bedroom apartments located in the city and paid 30% of their income towards rent; sobriety and treatment not preconditions for housing; an assertive community team saw tenants at least once a week and provided medication and money management.
	**Comparator**:
	Tenants live in a renovated residential hotel in studio apartments, each with a bathroom and kitchenette. Thirty percent of units in the hotel are for persons with mental illness. All tenants are prescreened for evidence of six months of clean and sober behaviour, and they can be asked to leave if they do not maintain good neighbour status. On‐site crisis services are continuously available to tenants in coordination with a New York City psychiatric emergency service. On‐site case managers are available to all tenants.
**Outcomes**	Housing stability, mental health, quality of life, hospital admission
**Notes**	

Risk of bias table

**Table:** 

Bias	Authors' judgement	Support for judgement
Random sequence generation (selection bias)	High risk	Participants could not be randomly assigned to housing types
Allocation concealment (selection bias)	High risk	Participants could not be randomized hence allocation concealment was not available.
Blinding of participants and personnel (performance bias)	High risk	Blinding of participants and personnel was not possible due to study design and the nature of the intervention.
Blinding of outcome assessment (detection bias)	Unclear risk	No description of blinding of outcome assessment.
Incomplete outcome data (attrition bias)	High risk	Eight participants in supported housing and ten in community residences completed only a baseline interview and were dropped from the study. The 18 persons were not statistically significant from the remaining group on any baseline characteristics. Overall, 80 percent of persons were available for the 12 months interview.
Selective reporting (reporting bias)	Low risk	No evidence of selective reporting were detected.
Other bias	Low risk	No other potential biases were detected.

Sosin et al., 1995

**Table:** 

**Methods**	**Design**: 3‐arm randomized control trial
	**Setting**: Chicago, Illinois, United States
	**Follow up**: 12 months
**Participants**	**Population**: Graduates of a short‐term inpatient substance use programmes who lacked housing
	**Sample size**: Total sample size n = 419, CM only n = 96, CM with Supported housing n = 136, control n = 187
**Interventions**	**Intervention**:
	The **case management only intervention** provided the progressive independence case management services under a scheme in which workers also helped clients find housing in the community. The **housing intervention** provided the case management model along with supported housing in one of three blocks of twenty apartments, found in recently renovated buildings serving those with low incomes. Both interventions were meant to last for up to eight months, although less intensive case management services could (but rarely did) last longer. Those who suffered two relapses or repeatedly violated program rules could not remain in the housing. They could continue case management as long as they agreed to a new contract that would guard against further relapses.
	**Control**:
	The clients placed in the **control condition** were referred by the relevant short‐term program staff to an outpatient or inpatient substance abuse agency, to welfare offices (as needed), and to an address of some kind. In the current paper, we ask whether, and by what mechanisms, each of the two treatment interventions reduced substance abuse and homelessness beyond the progress achieved by individuals placed in the control condition.
**Outcomes**	Housing stability, Substance use
**Notes**	

Risk of bias table

**Table:** 

Bias	Authors' judgement	Support for judgement
Random sequence generation (selection bias)	High risk	Study allocation followed a random approach but investigators did not specify random sequence generation procedures
Allocation concealment (selection bias)	High risk	Due to the nature of recruitment and assignment, allocation concealment was not possible.
Blinding of participants and personnel (performance bias)	High risk	Due to the nature of the intervention and population, blinding of participants and personnel was not possible.
Blinding of outcome assessment (detection bias)	Unclear risk	No information was provided on whether blinding of outcome assessment was undertaken.
Incomplete outcome data (attrition bias)	Unclear risk	No information was provided on the degree of missing data and how attrition was addressed
Selective reporting (reporting bias)	Low risk	No evidence of selective outcome reporting was detected.
Other bias	Low risk	No evidence of selective outcome reporting was detected.

Srebnik et al., 2013

**Table:** 

**Methods**	**Design**: Cost analysis based on a before‐and‐ after study without control group
**Participants**	**Population**: Adults who met the federal definition of individuals who are chronically homeless with significant disabling physical or psychiatric conditions who were referred either from King County Public Health's REACH homeless outreach team (Seattle, WA, USA) or from medical respite with incurred inpatient paid claims of at least US$10,000 in the previous year
**Interventions**	**Intervention**:
	Received a Housing First programme (Begin at Home)
	**Comparator**:
	Individuals who did not receive the Begin at Home intervention
**Outcomes**	**Cost or cost‐effectiveness**: The difference in service use associated cost reductions between the Housing First participants and comparison group of $36,579 outweighed the programme operating costs of $18,600 per person per year
**Notes**	

Risk of bias table

**Table:** 

Bias	Authors' judgement	Support for judgement
Random sequence generation (selection bias)	Unclear risk	
Allocation concealment (selection bias)	Unclear risk	
Blinding of participants and personnel (performance bias)	Unclear risk	
Blinding of outcome assessment (detection bias)	Unclear risk	
Incomplete outcome data (attrition bias)	Unclear risk	
Selective reporting (reporting bias)	Unclear risk	
Other bias	Unclear risk	

Stahler et al., 1995

**Table:** 

**Methods**	**Design**: Randomized control trial
	**Setting**: Philadelphia, United States
	**Follow up**: 6 months
**Participants**	**Population**: Adult males experiencing homelessness with alcohol and/or drug problems and stable mental health
	**Sample size**: Total sample size n = 722, Group 1 n = 220, Group 2 n = 200, Group 3 n = 302
**Interventions**	**Intervention**:
	Group 1: Integrative Comprehensive Residential Services: A 6‐month treatment program with a variety of services on site, such as individual counselling, group therapy, lectures, life skills preparation, job search skills training, and vocational and educational training
	Group 2: On‐Site Shelter‐Based Intensive Case Management: 4 to 9 months of ICM, case workers referred patients to a community network of services, caseloads were approximately 15 clients per case manager
	**Comparator**:
	Group 3: Usual Care Shelter Services with Case Management: City‐staffed case managers with caseloads of approximately 50‐75 per manager. The case managers primarily linked with ancillary supportive services and aided them in finding stable housing
**Outcomes**	Housing stability, mental health, substance use, employment
**Notes**	

Risk of bias table

**Table:** 

Bias	Authors' judgement	Support for judgement
Random sequence generation (selection bias)	Unclear risk	Participants were randomized to treatment conditions. However, no description of random sequence generation was provided.
Allocation concealment (selection bias)	Unclear risk	No description of allocation concealment was provided.
Blinding of participants and personnel (performance bias)	High risk	Blinding of participants and personnel was not possible due to the nature of the intervention and study design.
Blinding of outcome assessment (detection bias)	Unclear risk	No description of blinding of outcome assessment was provided.
Incomplete outcome data (attrition bias)	Low risk	"… 76% were located and interviewed approximately 6 months after discharge. Clients who received follow‐up interviews were compared with clients who did not on a number of baseline variables to examine the selection bias of the follow‐up sample. No differences between groups (p < .05) were found on any of the variables assessed, including alcohol and cocaine use (recent and lifetime), housing history, and age”
Selective reporting (reporting bias)	Low risk	No evidence of reporting outcomes selectively was detected.
Other bias	Low risk	No other potential biases were detected.

Stefancic & Tsemberis, 2007

**Table:** 

**Methods**	**Design**: 3‐arm randomized control trial
	**Setting**: Mount Vernon county, New York, United States
	**Follow up**: Around 4 years
	**Recruitment**: Staff at each agency conducted outreach by contacting eligible participants
	**Randomization**: Randomization procedures were not mentioned in the study
	**Allocation**: Allocation procedures were not mentioned
	**Blinding**: Not mentioned
	**Timing of outcome assessment**: Housing status at 20 months and housing retention over 47 months
	**Outcome assessor**: Not mentioned
**Participants**	**Population**: Individuals with severe mental illness who were chronic recidivists in the county homeless shelter system
	**Sample size**: Total n = 260, Pathways n = 105, Consortium n = 104, TAU n = 51
**Interventions**	**Intervention**:
	Pathways to Housing and a newly formed consortium of treatment and housing agencies from the county operating Housing First offered immediate access to permanent independent housing, without requiring treatment compliance or abstinence from drugs or alcohol.
	**Comparator**:
	The Treatment as Usual (TAU) group received traditional housing and treatment services.
**Outcomes**	Housing stability
**Notes**	

Risk of bias table

**Table:** 

Bias	Authors' judgement	Support for judgement
Random sequence generation (selection bias)	Unclear risk	No description of random sequence generation provided.
Allocation concealment (selection bias)	Unclear risk	No description of allocation concealment provided.
Blinding of participants and personnel (performance bias)	High risk	Blinding of participants and personnel was not possible due to the nature of the intervention and study design.
Blinding of outcome assessment (detection bias)	Unclear risk	No description of blinding of outcome assessment provided.
Incomplete outcome data (attrition bias)	Low risk	No evidence of incomplete outcome data reporting.
Selective reporting (reporting bias)	Low risk	No evidence of reporting outcome selectively.
Other bias	Low risk	No other potential biases detected.

Stergiopoulos et al., 2015

**Table:** 

**Methods**	**Design**: Randomized control trial and cost analysis
	**Setting**: Toronto, Montreal, Moncton, Winnipeg, Vancouver, Canada
	**Follow up**: Total n = 1198, Intervention n = 689, TAU n = 509
	**Recruitment**: Participants were recruited from community agencies and institutions serving homeless individuals
	**Randomization**: Using adaptive techniques, randomization was automated by the central data collection system
	**Allocation**: The allocation algorithm was concealed from researchers and participants
	**Blinding**: Researchers and participants were not blinded to treatment assignment
	**Timing of outcome assessment**: Every 3 months for up to 24 months
	**Outcome assessor**: Interviewers who used active outreach. No further details available
**Participants**	**Population**: Absolutely homeless or precariously housed individuals with mental illness and moderate support needs
	**Sample size**: Total n = 1198, Intervention n = 689, TAU n = 509
**Interventions**	**Intervention**:
	Scattered‐site supportive housing with mobile, off‐site intensive care management services, offering rapid, low‐barrier permanent housing in independent units with supports fostering participant empowerment, choice, personalised goals, hope, and resilience; participants paid up to 30% of their income toward rent, with a monthly rent supplement of CAN$375–600, paid by the programme directly to landlords.
	**Comparator**:
	Treatment as Usual (TAU): Participants were able to access a variety of traditional housing programs and community services which include services geared to the homeless population (drop‐in centres, emergency shelters, meal programs, street outreach services, supportive and alternative housing), as well as several mental health services available to both homeless and housed individuals.
**Outcomes**	Housing stability, mental health, quality of life, substance use, hospital admission
	**Cost or cost‐effectiveness**: The mean annual cost of supportive housing with intensive case management services was CAN$14,177 per participant, resulting in a mean net cost offset of $4,849 per participant per year, or 34% of the cost of the intervention.
**Notes**	

Risk of bias table

**Table:** 

Bias	Authors' judgement	Support for judgement
Random sequence generation (selection bias)	Low risk	Randomization was performed via adaptive randomization procedures. Adaptive randomization can ensure better balance between groups in small and moderate sized studies than strict randomization, by continually adjusting the probability of assignment to either group, depending on existing group assignment.
Allocation concealment (selection bias)	Low risk	The allocation algorithm was concealed from both the participants and the research staff.
Blinding of participants and personnel (performance bias)	High risk	Blinding of participants and personnel was not possible due to the nature of the intervention and study design.
Blinding of outcome assessment (detection bias)	High risk	Several aspects of the study prohibited blinding, including the nature of the administered questionnaires (detailed housing history and service use), location of participant interviews (some participants elected to be interviewed at their place of residence)
Incomplete outcome data (attrition bias)	Low risk	No evidence of incomplete outcome data provided
Selective reporting (reporting bias)	Low risk	No evidence of reporting outcomes selectively is provided
Other bias	Low risk	No other potential risk of biases

Stergiopoulos et al., 2019

**Table:** 

**Methods**	**Design**: Extension of a multicenter randomized control trial
	**Setting**: Toronto, Canada
	**Follow up**: Up to 6 years
	**Recruitment**: Participants were recruited from community agencies and institutions serving homeless individuals
	**Randomization**: Using adaptive techniques, randomization was automated by the central data collection system
	**Allocation**: The allocation algorithm was concealed from researchers and participants
	**Blinding**: Researchers and participants were not blinded to treatment assignment
	**Timing of outcome assessment**: Every 6 months for up to 6 years
	**Outcome assessor**: Interviewers who used active outreach. No further details available
**Participants**	**Population**: Absolutely homeless or precariously housed individuals with mental illness with moderate or high support needs
	**Sample size**:
	Total = 414
	High needs: Intervention n = 79, TAU n = 62
	Moderate needs: Intervention n = 160, TAU n = 113
**Interventions**	**Intervention**:
	Permanent housing with assertive community treatment offering multidisciplinary team‐based care, available 24 h per day and 7 days per week, and provided services primarily in the community, for participants with high support needs; permanent housing with intensive case management support, for up to 12 h per day for 7 days a week, with a case load of 17 participants per case manager for participants with moderate support needs; participants with moderate needs who self‐identified as ethnoracial individuals were provided with ethnoracial‐specific intensive case management services.
	**Comparator**:
	Participants assigned to TAU had access to a variety of housing, health, and social services in the community, including primary, specialty and hospital care, case management, and supportive housing.
**Outcomes**	Housing stability, quality of life, substance use
**Notes**	

Risk of bias table

**Table:** 

Bias	Authors' judgement	Support for judgement
Random sequence generation (selection bias)	Low risk	Participants were randomly assigned (1:1) to the intervention or TAU groups using computer‐based adaptive randomisation procedures at the study centre.
Allocation concealment (selection bias)	Low risk	Treatment allocation for each participant was sent to the interviewer's laptop at the end of the baseline interview by the central data centre for the study.
Blinding of participants and personnel (performance bias)	High risk	Due to the nature of the intervention, blinding of participants and personnel was not possible.
Blinding of outcome assessment (detection bias)	High risk	Personnel who conducted outcome assessments were not blinded to study assignment.
Incomplete outcome data (attrition bias)	Low risk	Attrition rates were within the anticipated levels and were accounted for in the sample size calculations. Multiple amputations with more than 20 models were performed to account for missing data when appropriate.
Selective reporting (reporting bias)	Low risk	No evidence of selective outcome reporting was detected.
Other bias	Low risk	No evidence for other biases was detected.

Susser et al., 1997

**Table:** 

**Methods**	**Design**: Randomized control trial and cost‐effectiveness analysis (societal perspective). Cost data were presented in 1992 US$.
	**Setting**: New York, United States
	**Follow up**: 18 months
**Participants**	**Population**: Homeless mentally ill men in a men's shelter in New York City
	**Sample size**: Total sample n = 96, CTI n = 48, TAU n = 48
**Interventions**	**Intervention**:
	CTI: To implement the first component of CTI, the clinical team devised a plan for the transfer of care from the shelter to other formal and informal supports. The plan focused on specific areas of potential discontinuity that were related to the risk of homelessness for that individual‐for instance, medication adherence and/or money management. Each man was then assigned to a CTI worker to implement the plan. CTI work entailed visiting the family home or community residence, being present at appointments, and locating patients and giving advice in times of crisis. To implement the second component of CTI, during the first 2 weeks after discharge the CTI worker spent time with the client in the community and observed his physical and social surroundings and daily habits. Subsequent support was individually tailored.
	**Comparator**:
	TAU: For usual services, the men were referred to mental health and rehabilitation programs that were generally of high quality. Following the usual model of discharge from an institution, the staff of the on‐site shelter psychiatry program were available to these agencies for consultation on request, but they did not actively seek a role in the client's care after discharge. The men were also referred as needed to community agencies for substance abuse, general health, income support, education, legal advocacy, and other services.
**Outcomes**	Housing stability, Mental health
	**Cost/Cost‐effectiveness**:
	Over 18 months, the total cost incurred by CTI clients was numerically higher than those receiving usual care ($52,374 vs $51,649).
	CTI significantly reduced nonhomeless nights compared to usual care (508 vs 450, p = .01).
	Over 18 months, the CTI was considered cost‐effective if the society were willing to pay $152 per nonhomeless night.
**Notes**	

Risk of bias table

**Table:** 

Bias	Authors' judgement	Support for judgement
Random sequence generation (selection bias)	Unclear risk	Participants were randomized. However, no description of random sequence generation was provided.
Allocation concealment (selection bias)	Unclear risk	No description of allocation concealment was provided.
Blinding of participants and personnel (performance bias)	High risk	Blinding of participants and personnel was not possible due to the nature of the intervention and study design.
Blinding of outcome assessment (detection bias)	Low risk	“These assessments were administered by a trained interviewer blind to the client's group status”.
Incomplete outcome data (attrition bias)	Low risk	"The 18‐month follow‐up data on homelessness were complete for 94 of the 96 participants (2 from USO ‐ control)… In a conservative approach, these two men who failed to complete the study were assigned 0 homeless nights for the period after they left the study”
Selective reporting (reporting bias)	Low risk	No evidence of reporting outcomes selectively was detected.
Other bias	Low risk	No other potential biases were detected.

Toro et al., 1997

**Table:** 

**Methods**	**Design**: Randomized control trial
	**Setting**: Buffalo, United States
	**Follow up**: 18 months
**Participants**	**Population**: Mentally ill homeless adults with children
	**Sample size**: Total sample size n=202 cases, Intervention n = 101, control n = 101
**Interventions**	**Intervention**:
	Demonstration Employment Project ‐ Training and Housing (DEPTH) ‐ holistic approach that combines services concerned with job training ‐ placement and locating permanent housing and support services ll targeted to the individual's specific needs and oriented toward the long term goal of helping the person escape homelessness. Central to DEPTH's services was intensive case management, offering access and linkage to services.
	**Comparator**:
	Control: Those in the no‐treatment control group received none of the DEPTH's services but were free to seek whatever other services were available to them in the community during the follow‐up period
**Outcomes**	Housing stability, mental health, substance use, income
**Notes**	

Risk of bias table

**Table:** 

Bias	Authors' judgement	Support for judgement
Random sequence generation (selection bias)	Unclear risk	Participants were randomized to one of two conditions. However, description of random sequence generation was not provided.
Allocation concealment (selection bias)	Low risk	“Because baseline interviews were conducted before random assignment, all research participants and interviewers were not informed of condition”.
Blinding of participants and personnel (performance bias)	High risk	Blinding of participants and personnel was not possible due to the nature of the intervention and study design.
Blinding of outcome assessment (detection bias)	High risk	“Although all interviewers and clients were not informed of condition at the baseline interview (because random assignment did not occur until after the interview) and interviewers were not told about assignments afterwards, during the course of the follow‐up interviews the interviewers did sometimes become aware that particular clients were being served by the DEPTH Program. This awareness was unavoidable, because clients would sometimes spontaneously indicate their participation in DEPTH. Their awareness of this information may have affected some of the follow‐up data collected”.
Incomplete outcome data (attrition bias)	Low risk	Even though there was high attrition rate (Almost 50% were lost to follow‐up by the end of the study period), mean substitution was used to eliminate all missing data points in the repeated measures design.
Selective reporting (reporting bias)	Low risk	No evidence of reporting outcomes selectively was detected.
Other bias	Low risk	No other potential biases were found.

Towe et al., 2019

**Table:** 

**Methods**	**Design**: Randomized control trial
	**Setting**: New York, NY, United States
	**Follow up**: 12 months
**Participants**	**Population**: Homeless single adults living in HIV emergency shelters
	**Sample size**: Total sample size n = 236, EHPA n = 119, Usual care n = 117
**Interventions**	**Intervention**:
	Enhanced Housing Placement Assistance (EPHA) is a rapid‐rehousing program where participants were immediately assigned a case manager who worked to identify available and affordable housing for participants as quickly as possible, provided rent and move‐in assistance, and delivered intensive housing stabilization services up to one year post‐enrollment.
	**Comparator**:
	Usual care participants were immediately referred to an organization that assists with finding housing for people living with HIV/AIDS and offering housing stabilization services as needed within 3 month post enrollment.
**Outcomes**	Housing stability
**Notes**	

Risk of bias table

**Table:** 

Bias	Authors' judgement	Support for judgement
Random sequence generation (selection bias)	Low risk	Rooms were randomized to the treatment or Usual Care arm of the study using a computer‐generated random number list
Allocation concealment (selection bias)	High risk	Recruitment and allocation procedures indicate that allocation was not concealed
Blinding of participants and personnel (performance bias)	High risk	Due to the nature of the intervention, blinding of participants and personnel was not possible
Blinding of outcome assessment (detection bias)	Low risk	Outcome assessors were blinded to study assignment
Incomplete outcome data (attrition bias)	Low risk	All missing data were addressed adequately and the characteristics of those who adhered to the intervention resembled those who were lost to follow‐up
Selective reporting (reporting bias)	Low risk	No evidence of selective outcome reporting was detected.
Other bias	Low risk	No evidence of other biases was detected

Tsemberis et al., 2004

**Table:** 

**Methods**	**Design**: Randomized control trial
	**Setting**: New York, United States
	**Follow up**: 24 months
	**Recruitment**: Participants were referred by hospitals, drop‐in centres, outreach teams, direct outreach by project staff, self‐referrals, and other agencies
	**Randomization**: Participants were randomized but the procedures were not mentioned in the paper
	**Allocation**: Allocation procedures were not mentioned
	**Blinding**: Interviewers were blind to participants' assignment for baseline interviews but not for follow‐up interviews
	**Timing of outcome assessment**: Every 6 months for up to 24 months
	**Outcome assessor**: Interviewers trained to engage individuals who are homeless and mentally ill
**Participants**	**Population**: Homeless individuals with dual diagnoses
	**Sample size**: Total n = 225, Intervention n = 99, TAU n = 126
**Interventions**	**Intervention**:
	Pathways to housing; immediate provision of an apartment of the participant's own without any prerequisites for psychiatric treatment or sobriety; participants were also offered treatment, support, and other services by the programme's assertive community treatment team with two modifications (a nurse practitioner and housing specialist).
	**Comparator**:
	Usual care: Continuum of Care supportive housing programs subscribe to the abstinence‐sobriety model based on the belief that without strict adherence to treatment and sobriety, housing stability is not possible.
**Outcomes**	Housing stability, mental health, quality of life, substance use, hospital admission
**Notes**	

Risk of bias table

**Table:** 

Bias	Authors' judgement	Support for judgement
Random sequence generation (selection bias)	Unclear risk	Description of random sequence generation was not provided.
Allocation concealment (selection bias)	Unclear risk	Description of allocation concealment not provided.
Blinding of participants and personnel (performance bias)	High risk	Blinding of participants and personnel was not possible due to the nature of the intervention and study design
Blinding of outcome assessment (detection bias)	High risk	Participants were blind to participants' assignment for baseline interviews but not for follow‐up interviews.
Incomplete outcome data (attrition bias)	Low risk	No evidence of incomplete outcome data reporting.
Selective reporting (reporting bias)	Low risk	No evidence of reporting outcomes selectively.
Other bias	Low risk	No other potential biases detected.

Upshur et al., 2015

**Table:** 

**Methods**	**Design**: Randomized control trial
	**Setting**: Not specified, United States
	**Follow up**: 6 months
**Participants**	**Population**: Homeless women with alcohol use problems
	**Sample size**: Total sample n = 82, Intervention n = 42, Control n = 40
**Interventions**	**Intervention**:
	**Project RENEWAL intervention**: Intervention patients received the guideline‐based Primary Care Provider brief intervention for problem alcohol use, and referral to the CM for ongoing follow‐up visits for 6 months.
	1) providing evidence‐based training and supports to the medical leadership and randomized intervention PCPs;
	2) modifying the electronic medical record (EMR) to provide alcohol screening results and alcohol‐specific notes for PCP and care manager (CM) visits; and
	3) training a CM specifically designated to provide intervention participants with alcohol education materials, ongoing self‐management support, linkage to formal addiction treatment services and self‐help groups, and wellness counseling and goal setting.
	**Comparator**:
	**Usual care**: Usual care patients did not receive referrals to, or outreach from, the study‐trained CM, and their PCPs were not provided any alcohol intervention training or patient materials. They delivered usual care for medical conditions, including any behavioral health or drug or alcohol use problems
**Outcomes**	Housing stability, Mental health, Substance use
**Notes**	

Risk of bias table

**Table:** 

Bias	Authors' judgement	Support for judgement
Random sequence generation (selection bias)	Low risk	“All PCPs (MDs, PAs and NPs) who provided ongoing primary care services at the clinic were randomly assigned to intervention or usual care condition by a computer program”
Allocation concealment (selection bias)	Unclear risk	No description of allocation concealment was provided.
Blinding of participants and personnel (performance bias)	High risk	Blinding of participants and personnel was not possible due to the nature of the intervention and study design.
Blinding of outcome assessment (detection bias)	Low risk	*“*…follow‐up research interviews by the RC who was not involved with any intervention training or clinical care activities and was not aware of type of treatment received by any participants”
Incomplete outcome data (attrition bias)	Unclear risk	No description of reasons some women dropped out at 3 and 6 months. Also, no description of differences between those who were retained and those who dropped out.
Selective reporting (reporting bias)	Low risk	No evidence of reporting outcomes selectively was detected.
Other bias	Low risk	No other potential biases were detected.

Weinreb et al., 2016

**Table:** 

**Methods**	**Design**: Cluster randomized control trial
	**Setting**: Queens and Bronx, New York, United States
	**Follow up**: 6 months
**Participants**	**Population**: Homeless mothers who screened positive for major depressive disorder
	**Sample size**: Total sample n = 67, Intervention n = 42, Control n = 25
**Interventions**	**Intervention**:
	Integrated Care Model for Homeless Mothers (ICMHM): provided an appointment with the primary care physician (PCP) and with the care manager to initiate depression treatment following the intervention model and to address any other health care needs
	**Comparator**:
	Usual Care: provided appointments with the PCP who initiated treatment as usual, which could include antidepressant medication and recommendation for psychotherapy outside the clinic. Women in the usual‐care group received general case management services that were available to all families receiving health services at the clinic. These services included, for example, assistance with obtaining public benefits, linking with community resources for family activities, outside mental health or substance use services, and meeting children's educational needs.
**Outcomes**	Housing stability, Mental health, Employment
**Notes**	

Risk of bias table

**Table:** 

Bias	Authors' judgement	Support for judgement
Random sequence generation (selection bias)	Unclear risk	The study was conducted at 2 randomly selected and then assigned (one to intervention and one to usual care) primary care clinics. However, there was no description of random sequence generation provided.
Allocation concealment (selection bias)	Unclear risk	No description of allocation concealment was provided
Blinding of participants and personnel (performance bias)	High risk	Blinding of participants and personnel was not possible due to the nature of the intervention and study design.
Blinding of outcome assessment (detection bias)	Unclear risk	No description of blinding of outcome assessment was provided.
Incomplete outcome data (attrition bias)	Low risk	“Demographic, physical, and mental health baseline characteristics were similar between those who completed the study and those who did not”
Selective reporting (reporting bias)	Low risk	No evidence of reporting outcomes selectively was detected.
Other bias	Low risk	No other potential biases were detected.

Wolff, 1997

**Table:** 

**Methods**	**Design**: A cost‐consequence analysis based on an RCT. Cost data were presented in 1992 US$.
**Participants**	**Population**: Individuals with severe mental illness who were at risk for homelessness
**Interventions**	**Intervention**:
	ACT alone
	ACT with community workers
	**Comparator**:
	Brokered case management (purchase of services)
**Outcomes**	**Cost/Cost‐effectiveness**:
	The total costs for ACT only, ACT with community workers, and brokered case management were $49,510, $39,913, and $45,076, respectively. There was no statistically different in total costs across 3 groups.
	The ACT with community workers (and the effect of ACT only) were statistically associated with better more contacts with case managers, improved client satisfaction, and fewer mental health symptoms.
**Notes**	

Risk of bias table

**Table:** 

Bias	Authors' judgement	Support for judgement
Random sequence generation (selection bias)	Unclear risk	
Allocation concealment (selection bias)	Unclear risk	
Blinding of participants and personnel (performance bias)	Unclear risk	
Blinding of outcome assessment (detection bias)	Unclear risk	
Incomplete outcome data (attrition bias)	Unclear risk	
Selective reporting (reporting bias)	Unclear risk	
Other bias	Unclear risk	

Wolitski 2009

**Table:** 

**Methods**	**Design**: Randomized control trial
	**Setting**: Baltimore, MD; Chicago, IL; Los Angeles, CA, United States
	**Follow up**: 18 months
	**Recruitment**: Participants were recruited by agencies providing the service
	**Randomization**: Participants were randomized by a computer using a randomly generated sequences with blocking
	**Allocation**: Study staff used a computer to learn the condition assignment
	**Blinding**: Neither staff nor participants were blinded
	**Timing of outcome assessment**: At baseline and 6‐, 12‐, and 18 months after
	**Outcome assessor**: Not reported
**Participants**	**Population**: Homeless or unstably housed people with HIV
	**Sample size**: Total n = 630, Intervention n = 315, Control n = 315
**Interventions**	**Intervention**:
	A federal programme providing immediate housing opportunities for people with AIDS in the form of rental assistance with case management.
	**Comparator**:
	Control group: customary housing services with case management.
**Outcomes**	Housing stability, mental health, hospitalisation
**Notes**	

Risk of bias table

**Table:** 

Bias	Authors' judgement	Support for judgement
Random sequence generation (selection bias)	Low risk	Participants were randomized by computer (randomly generated sequences with blocking) to either the treatment or comparison groups.
Allocation concealment (selection bias)	High risk	Neither staff nor participants were blinded to the intervention.
Blinding of participants and personnel (performance bias)	High risk	Blinding of participants and personnel was not possible due to the nature of the intervention and study design.
Blinding of outcome assessment (detection bias)	Unclear risk	No description of blinding of outcomes assessment was provided.
Incomplete outcome data (attrition bias)	High risk	Retention for follow‐up assessments ranged from 82% to 96%. Retention rates in the treatment condition were significantly higher than those in the comparison condition at the 6‐ and 18‐ month follow‐up assessment.
Selective reporting (reporting bias)	Low risk	No evidence of reporting outcomes selectively was detected.
Other bias	Unclear risk	Self‐reported data obtained in this study may be affected by socially desirable responding and recall biases, which were minimized by using A‐CASI for more sensitive questions and a 90‐day recall period rather than a longer period.

Yoon et al., 2017

**Table:** 

**Methods**	**Design**: Randomized controlled trial
	**Recruitment**: Some potential participants were referred during VA primary care visits; others visited the study office in response to posted fliers advertising the study.
	**Setting**: VA primary care clinics, 2 east coast and 2 west coast cities, US
	**Follow‐up**: 6 months
**Participants**	**Population**: Homeless veterans
	**Eligibility criteria**: Had to be homeless and had to use Veterans Affairs Primary care
	**Sample size**: Total n = 375, intervention n = 195, control n = 180
	**Baseline characteristics**: “Baseline characteristics of both study arms were not significantly different, except for marital status. In both groups, most patients were men (intervention n = 188 (96%); usual care n = 171 (95%)). In both groups, the largest proportion of patients was white (peer mentor: 45% [N = 88] white, 34% [N = 66] black, 4% [N = 8] Hispanic, and 17% [N = 33] other; usual care: 43% [N = 78]
	white, 37% [N = 66] black, 5% [N = 9] Hispanic, and 15% [N = 27] other). The mean(SD) age of patients in the two groups was 52 (10). Most patients in both cohorts had at least one mental health condition; the largest proportions had depression or anxiety or both”
**Interventions**	**Peer mentor intervention**:
	“Patients in the peer mentor intervention received regular contacts with an assigned peer mentor over a six‐month period in addition to their usual primary care from their Patient Aligned Care Teams (PACT) or Homeless Patient Aligned Care Teams (H‐PACT) clinical team. Peer mentors were salaried employees embedded in the primary care team and were formerly homeless veterans who had extensive experience with VA health care services.
	Responsibilities focused on facilitating didactic exercises, serving as a role model, assisting veterans to articulate goals and needs, teaching problem‐solving techniques, and providing assistance navigating the health care system. Mentors scheduled routine visits with their assigned patients over a six‐month period to reinforce care plans identified in the clinic visit”.
	**Usual care**:
	“Patients randomly assigned to usual care continued to receive primary care without any other additional services”.
**Outcomes**	**Hospitalization**: inpatient admissions and emergency department visits were identified using the VA Medical Statistical Analysis System files
**Notes**	

Risk of bias table

**Table:** 

Bias	Authors' judgement	Support for judgement
Random sequence generation (selection bias)	Unclear risk	Participants were randomized to conditions. However there was no description of random sequence generation provided.
Allocation concealment (selection bias)	Unclear risk	No description of allocation concealment was provided.
Blinding of participants and personnel (performance bias)	High risk	Blinding of participants and personnel was not possible due to the nature of the intervention and study design.
Blinding of outcome assessment (detection bias)	Unclear risk	No description of blinding of outcome assessment was provided.
Incomplete outcome data (attrition bias)	Unclear risk	No description of attrition rate or reasons for dropping out were provided.
Selective reporting (reporting bias)	Low risk	No evidence of reporting outcomes selectively was detected.
Other bias	Low risk	No other potential biases were detected.

Young et al., 2009

**Table:** 

**Methods**	**Design**: Quasi‐experimental trial
	**Setting**: Florida and California, United States
	**Follow up**: 6 months
	**Recruitment**: All eligible clients were invited to participate in the study immediately upon admittance into the CCISC‐RT or ACT‐SH programs
	**Randomization**: No randomization procedures were performed
	**Allocation**: No allocation procedures were mentioned
	**Blinding**: Not mentioned
	**Timing of outcome assessment**: At baseline and 6 months follow‐up
	**Outcome assessor**: An independent research team not affiliated with either treatment program
**Participants**	**Population**: Individuals who were homeless or at risk of homelessness due to incarceration and who had severe co‐occurring mental health and substance use disorders.
	**Sample size**: Total n = 163, ACT‐SH n = 67, CCISC‐RT n = 96
**Interventions**	**Intervention**:
	CCISC‐RT; Residential treatment facility with services that included comprehensive screening and assessment and individualised treatment planning, including case management, individual counselling, group therapy, recreational therapy, vocational training, and medication management as needed; CCISC‐RT staff did random urine screening of clients, and abstinence was expected of all clients in the programme, however, relapse did not result in immediate discharge from the programme.
	**Comparator**:
	The ACT‐SH program offers consumers housing, individually tailored treatment, rehabilitation, and support services based on their most salient needs ranging from grocery shopping to filling prescriptions.
**Outcomes**	Housing stability, mental health, substance use
**Notes**	

Risk of bias table

**Table:** 

Bias	Authors' judgement	Support for judgement
Random sequence generation (selection bias)	High risk	This is a quasi‐experimental study with no random sequence generation.
Allocation concealment (selection bias)	High risk	Allocation concealment was not possible due to study design.
Blinding of participants and personnel (performance bias)	High risk	Blinding of participants and personnel was not possible due to the nature of the intervention and study design.
Blinding of outcome assessment (detection bias)	Low risk	“An independent research team not affiliated with either treatment program conducted participant interviews to enhance the truthfulness and confidentiality of participant responses”
Incomplete outcome data (attrition bias)	Low risk	No evidence of incomplete outcome data reporting.
Selective reporting (reporting bias)	Low risk	No evidence of reporting outcomes selectively.
Other bias	Low risk	No other potential biases detected.

#### Characteristics of excluded studies

**Table:** 

Adair et al., 2016	
**Reason for exclusion**	Irrelevant outcomes
Adair et al., 2017	
**Reason for exclusion**	Irrelevant outcomes
Aidala et al., 2016	
**Reason for exclusion**	Wrong study design
Akin et al., 2017	
**Reason for exclusion**	Wrong population
Alimohamed‐Janmohamed et al., 2010	
**Reason for exclusion**	Wrong intervention
Allen, 2006	
**Reason for exclusion**	Wrong study design
Alphs et al., 2014	
**Reason for exclusion**	Wrong publication type
Althaus et al., 2011	
**Reason for exclusion**	Wrong intervention
Anderson et al., 2002	
**Reason for exclusion**	Wrong study design
Anderson et al., 2003	
**Reason for exclusion**	Wrong study design
Aubry 2015	
**Reason for exclusion**	Wrong study design
Ball et al., 2005	
**Reason for exclusion**	Wrong intervention
Bamberger, 2016	
**Reason for exclusion**	Wrong study design
Barker 2017	
**Reason for exclusion**	Wrong intervention
Barlow et al., 2007	
**Reason for exclusion**	Wrong intervention
Bassuk et al. 2014	
**Reason for exclusion**	Wrong intervention
Baumgartner & Herman, 2012	
**Reason for exclusion**	Irrelevant outcomes
Bearman et al., 1997	
**Reason for exclusion**	Wrong intervention
Beaudoin 2016	
**Reason for exclusion**	Wrong study design
Bell et al., 2015	
**Reason for exclusion**	Wrong population
Benston, 2015	
**Reason for exclusion**	Wrong study design
Black et al., 2012	
**Reason for exclusion**	Wrong study design
Bradford et al., 2005	
**Reason for exclusion**	Wrong intervention
Broner et al., 2009	
**Reason for exclusion**	Wrong study design
Brown et al., 1991	
**Reason for exclusion**	Wrong intervention
Brush & Powers, 2001	
**Reason for exclusion**	Wrong intervention
Buchanan et al., 2009	
**Reason for exclusion**	Irrelevant outcomes
Burns & Santos, 1995	
**Reason for exclusion**	Wrong publication type
Bybee & Sullivan, 2005	
**Reason for exclusion**	Wrong intervention
CADTH 2014	
**Reason for exclusion**	Wrong publication type
Calsyn et al., 1998	
**Reason for exclusion**	Wrong study design
Calsyn et al., 2005	
**Reason for exclusion**	Wrong population
Carlson et al., 2016	
**Reason for exclusion**	Wrong study design
Chilvers et al., 2006	
**Reason for exclusion**	Wrong study design
Chinman et al., 2000	
**Reason for exclusion**	Wrong study design
Chung 2018	
**Reason for exclusion**	Wrong intervention
Clark et al., 2016	
**Reason for exclusion**	Wrong study design
Coldwell & Bender 2007	
**Reason for exclusion**	Wrong study design
Collard et al., 2014	
**Reason for exclusion**	Wrong study design
Collins et al., 2012	
**Reason for exclusion**	Irrelevant outcomes
Collins, 2013	
**Reason for exclusion**	Wrong population
Collins et al., 2020	
**Reason for exclusion**	Wrong publication type
Cooper et al., 2010	
**Reason for exclusion**	Wrong study design
De Vet 2013	
**Reason for exclusion**	Wrong study design
Devine et al., 1995	
**Reason for exclusion**	Wrong intervention
Dickey, 2000	
**Reason for exclusion**	Wrong study design
Dieterich 2017	
**Reason for exclusion**	Wrong study design
Dixon et al., 1997	
**Reason for exclusion**	Wrong population
Dixon et al., 1998	
**Reason for exclusion**	Irrelevant outcomes
Dore et al., 2015	
**Reason for exclusion**	Wrong intervention
Draine & Solomon, 1994	
**Reason for exclusion**	Wrong study design
Drake et al., 1997	
**Reason for exclusion**	Wrong intervention
Drummond et al., 2016	
**Reason for exclusion**	Wrong population
Erkel et al., 1994	
**Reason for exclusion**	Wrong population
Fitzpatrick‐Lewis et al., 2011	
**Reason for exclusion**	Wrong study design
Fowler & Schoeny, 2015	
**Reason for exclusion**	Wrong intervention
Fowler & Schoeny, 2017	
**Reason for exclusion**	Irrelevant outcomes
Francis et al., 1999	
**Reason for exclusion**	Wrong intervention
French et al., 2002	
**Reason for exclusion**	Wrong intervention
Gensichen et al., 2006	
**Reason for exclusion**	Wrong population
Gibson et al., 2017	
**Reason for exclusion**	Wrong study design
Glendening & Shinn, 2017	
**Reason for exclusion**	Wrong intervention
Goering et al., 2015	
**Reason for exclusion**	Irrelevant outcomes
Goodson et al., 2000	
**Reason for exclusion**	Wrong population
Grajo et al., 2019	
**Reason for exclusion**	Irrelevant outcomes
Greeson et al., 2015	
**Reason for exclusion**	Wrong intervention
Guehne et al., 2017	
**Reason for exclusion**	Wrong study design
Gulcur et al., 2007	
**Reason for exclusion**	Irrelevant outcomes
Gutman & Raphael‐Greenfield, 2017	
**Reason for exclusion**	Irrelevant outcomes
Haas et al., 1993	
**Reason for exclusion**	Wrong study design
Hadley & Holahan, 2003	
**Reason for exclusion**	Wrong intervention
Hampton & Chafetz, 2002	
**Reason for exclusion**	Wrong study design
Haskett et al., 2017	
**Reason for exclusion**	Wrong intervention
Health Quality Ontario, 2016	
**Reason for exclusion**	Wrong study design
Helfrich et al., 2011	
**Reason for exclusion**	Wrong intervention
Herinckx et al., 1997	
**Reason for exclusion**	Wrong population
Herman et al., 2008	
**Reason for exclusion**	Wrong population
Herman & Mandiberg, 2010	
**Reason for exclusion**	Wrong study design
Herman, 2014	
**Reason for exclusion**	Wrong study design
Hetling et al., 2016	
**Reason for exclusion**	Wrong population
Hoell et al., 2017	
**Reason for exclusion**	Wrong study design
Holl et al., 2016	
**Reason for exclusion**	Wrong study design
Holter, 1998	
**Reason for exclusion**	Wrong intervention
Hultman et al., 1995	
**Reason for exclusion**	Could not be retrieved
Hunt et al., 2013	
**Reason for exclusion**	Wrong study design
Hwang 2005	
**Reason for exclusion**	Wrong study design
Hwang et al., 2011	
**Reason for exclusion**	Wrong intervention
Hwang 2012	
**Reason for exclusion**	Irrelevant outcomes
Hwang 2014	
**Reason for exclusion**	Wrong study design
Johnson, 2005	
**Reason for exclusion**	Wrong population
Kangovi et al., 2017	
**Reason for exclusion**	Wrong population
Karper et al., 2008	
**Reason for exclusion**	Wrong intervention
Kasprow & Rosenheck, 2007	
**Reason for exclusion**	Wrong study design
Kertesz	
**Reason for exclusion**	Wrong intervention
Kertesz et al., 2007	
**Reason for exclusion**	Wrong intervention
Killaspy et al., 2006	
**Reason for exclusion**	Wrong population
Kleinman et al., 2017	
**Reason for exclusion**	Wrong intervention
Kneipp et al., 2011	
**Reason for exclusion**	Wrong population
Koffarnus et al., 2013	
**Reason for exclusion**	Irrelevant outcomes
Kuerbis 2011	
**Reason for exclusion**	Wrong population
Kumar & Klein, 2013	
**Reason for exclusion**	Wrong study design
Kyle & Dunn, 2008	
**Reason for exclusion**	Wrong study design
Lam et al., 1996	
**Reason for exclusion**	Wrong intervention
Larsen & Nordentoft, 2010	
**Reason for exclusion**	Wrong study design
Leaver et al., 2007	
**Reason for exclusion**	Wrong study design
LePage	
**Reason for exclusion**	Wrong intervention
LePage 2006	
**Reason for exclusion**	Wrong intervention
LePage & Garcia‐Rea, 2012	
**Reason for exclusion**	Wrong intervention
Lester et al., 2007	
**Reason for exclusion**	Irrelevant outcomes
Leventhal & Dupéré, 2011	
**Reason for exclusion**	Wrong population
Levitt et al., 2013	
**Reason for exclusion**	Wrong intervention
Lindberg et al., 2010	
**Reason for exclusion**	Wrong intervention
Lucas et al., 2008	
**Reason for exclusion**	Wrong study design
Luchenski et al., 2018	
**Reason for exclusion**	Wrong study design
Ly 2015	
**Reason for exclusion**	Wrong intervention
Macnaughton et al., 2016	
**Reason for exclusion**	Irrelevant outcomes
Mangalore & Knapp, 2007	
**Reason for exclusion**	Wrong population
Manuel et al., 2011	
**Reason for exclusion**	Irrelevant outcomes
Marks et al., 2000	
**Reason for exclusion**	Wrong intervention
Martin et al., 1997	
**Reason for exclusion**	Wrong intervention
Masson et al., 2002	
**Reason for exclusion**	Wrong intervention
McCormick & White, 2016	
**Reason for exclusion**	Wrong intervention
McGrew & Danner, 2009	
**Reason for exclusion**	Wrong study design
McLaughlin, 2011 no.2(?) OM to check	
**Reason for exclusion**	Wrong intervention
McMorrow et al., 2014	
**Reason for exclusion**	Wrong population
Meyer & Morrissey, 2007	
**Reason for exclusion**	Wrong publication type
Milby et al., 2005	
**Reason for exclusion**	Wrong intervention
Milby n.d.	
**Reason for exclusion**	Wrong publication type
Morgenstern et al., 2009	
**Reason for exclusion**	Wrong population
Mueser 1998	
**Reason for exclusion**	Wrong publication type
Munoz & Panadero, 2002	
**Reason for exclusion**	Could not be retrieved
Muser et al., 2015	
**Reason for exclusion**	Wrong study design
Needels et al., 2005	
**Reason for exclusion**	Wrong population
Nelson et al., 2007	
**Reason for exclusion**	Wrong study design
Nelson et al., 2014	
**Reason for exclusion**	Wrong study design
No Author(s) Listed 2018	
**Reason for exclusion**	Wrong publication type
No Author Listed 2017	
**Reason for exclusion**	Wrong publication type
Noether et al., 2005	
**Reason for exclusion**	Wrong intervention
Noh, 2018	
**Reason for exclusion**	Wrong intervention
Nordentoft & Jessen‐Petersen, 1992	
**Reason for exclusion**	Could not be retrieved
Nossel et al., 2016	
**Reason for exclusion**	Wrong intervention
Nugent et al., 1998	
**Reason for exclusion**	Wrong study design
Nuttbrock et al., 1997	
**Reason for exclusion**	Wrong intervention
Nuttbrock et al., 1998	
**Reason for exclusion**	Wrong intervention
Nyamathi et al., 2014	
**Reason for exclusion**	Wrong study design
Nyamathi et al., 2015	
**Reason for exclusion**	Wrong study design
Nyamathi et al., 2017	
**Reason for exclusion**	Wrong intervention
Osypuk et al., 2019	
**Reason for exclusion**	Wrong population
Parpouchi et al., 2016	
**Reason for exclusion**	Irrelevant outcomes
Parpouchi et al., 2018	
**Reason for exclusion**	Irrelevant outcomes
Pollack et al., 2019	
**Reason for exclusion**	Wrong population
Pope et al., 1993	
**Reason for exclusion**	Wrong intervention
Poulin et al., 2010	
**Reason for exclusion**	Wrong intervention
Price et al., 2015	
**Reason for exclusion**	Wrong intervention
Prince, 2013	
**Reason for exclusion**	Wrong publication type
Protocol ACTRN12616000162415	
**Reason for exclusion**	Wrong publication type
Protocol ISRCTN15900054	
**Reason for exclusion**	Wrong publication type
Protocol ISRCTN44050004	
**Reason for exclusion**	Wrong publication type
Protocol ISRCTN66721740	
**Reason for exclusion**	Wrong publication type
Protocol NCT00057161	
**Reason for exclusion**	Wrong publication type
Protocol NCT00490581	
**Reason for exclusion**	Wrong publication type
Protocol NCT00621465	
**Reason for exclusion**	Wrong publication type
Protocol NCT01346514	
**Reason for exclusion**	Wrong publication type
Protocol NCT01430741	
**Reason for exclusion**	Wrong publication type
Protocol NCT01550757	
**Reason for exclusion**	Wrong publication type
Protocol NCT01570712	
**Reason for exclusion**	Wrong publication type
Protocol NCT01932801	
**Reason for exclusion**	Wrong publication type
Protocol NCT02258425	
**Reason for exclusion**	Wrong publication type
Protocol NCT02440360	
**Reason for exclusion**	Wrong publication type
Protocol NCT02553616	
**Reason for exclusion**	Wrong publication type
Protocol NCT02723058	
**Reason for exclusion**	Wrong publication type
Protocol NCT02816294	
**Reason for exclusion**	Wrong publication type
Protocol NCT03334825	
**Reason for exclusion**	Wrong publication type
Protocol NCT03766165	
**Reason for exclusion**	Wrong publication type
Protocol NCT03770221	
**Reason for exclusion**	Wrong publication type
Protocol NCT03779204	
**Reason for exclusion**	Wrong publication type
Protocol NCT03910218	
**Reason for exclusion**	Wrong publication type
Protocol NCT04012697	
**Reason for exclusion**	Wrong publication type
Protocol NCT04135703	
**Reason for exclusion**	Wrong publication type
Protocol NTR1640	
**Reason for exclusion**	Wrong publication type
Protocol NTR3254	
**Reason for exclusion**	Wrong publication type
Protocol NTR3463	
**Reason for exclusion**	Wrong publication type
Protocol NTR896	
**Reason for exclusion**	Wrong publication type
Rahav et al., 1995	
**Reason for exclusion**	Wrong intervention
Rash et al., 2017	
**Reason for exclusion**	Irrelevant outcomes
Read, 2008	
**Reason for exclusion**	Wrong intervention
Rew et al., 2018	
**Reason for exclusion**	Wrong intervention
Rezansoff et al., 2016	
**Reason for exclusion**	Irrelevant outcomes
Rog et al., 2014	
**Reason for exclusion**	Wrong study design
Rosenblum et al., 2005	
**Reason for exclusion**	Wrong intervention
Rosenblum et al., 2006	
**Reason for exclusion**	Wrong population
Rosenheck 1997	
**Reason for exclusion**	Wrong intervention
Rosenheck & Seibyl, 1998	
**Reason for exclusion**	Wrong intervention
Sacks et al., 1999	
**Reason for exclusion**	Wrong study design
Sacks et al., 2010	
**Reason for exclusion**	Wrong intervention
Sajatovic et al., 2017	
**Reason for exclusion**	Wrong study design
Schumacher et al., 2002	
**Reason for exclusion**	Wrong intervention
Schumacher et al., 2007	
**Reason for exclusion**	Wrong intervention
Shern et al., 1997	
**Reason for exclusion**	Wrong study design
Slesnick & Erdem, 2012	
**Reason for exclusion**	Wrong intervention
Slesnick & Erdem, 2013	
**Reason for exclusion**	Wrong intervention
Slesnick et al., 2015	
**Reason for exclusion**	Wrong intervention
Sokol and Fisher 2016	
**Reason for exclusion**	Wrong population
Somers 2013	
**Reason for exclusion**	Irrelevant outcomes
Somers 2013	
**Reason for exclusion**	Irrelevant outcomes
Stahler et al., 1993	
**Reason for exclusion**	Wrong study design
Stergiopoulos 2017	
**Reason for exclusion**	Wrong population
Tomita et al., 2014	
**Reason for exclusion**	Irrelevant outcomes
Tomita & Herman, 2015	
**Reason for exclusion**	Irrelevant outcomes
Torrey, 1990	
**Reason for exclusion**	Wrong intervention
Tsai et al., 2010	
**Reason for exclusion**	Wrong study design
Valencia 1999	
**Reason for exclusion**	Wrong publication type
Vallesi et al., 2019	
**Reason for exclusion**	Wrong publication type
Vanderplasschen et al., 2007	
**Reason for exclusion**	Wrong study design
Veldhuizen et al., 2015	
**Reason for exclusion**	Irrelevant outcomes
Whittaker et al., 2017	
**Reason for exclusion**	Wrong intervention
Wood et al., 1998	
**Reason for exclusion**	Wrong intervention
Wu et al., 2008	
**Reason for exclusion**	Wrong study design
Yamin et al., 2015	
**Reason for exclusion**	Wrong study design
Zerger et al., 2014	
**Reason for exclusion**	Wrong study design
Zhang et al., 2013	
**Reason for exclusion**	Wrong intervention
Zhang & Slesnick, 2018	
**Reason for exclusion**	Wrong intervention
Zlotnick et al., 2012	
**Reason for exclusion**	Wrong study design
Zulman et al., 2017	
**Reason for exclusion**	Wrong population
Zur et al., 2014	
**Reason for exclusion**	Wrong intervention

Footnotes

Characteristics of studies awaiting classification

Footnotes

Characteristics of ongoing studies

Footnotes

## Summary of findings tables

**Additional tables**

## Data and analyses

1. Housing stability

**Table:** 

**Outcome or Subgroup**	**Studies**	**Participants**	**Statistical Method**	**Effect Estimate**
1.1 Number of participants in stable housing at 18 months or later	2	966	Odds Ratio (M‐H, Random, 95% CI)	3.58 [2.36, 5.43]

## Sources of support

### Internal sources

- Stakeholders, Canada

Community scholars and other persons with lived experience provided assistance in priority setting for the review.

### External sources

- Inner City Health Associates, Canada

Financial Support

**Feedback**

## Appendix A

### Search Strategies

**Database: Ovid MEDLINE(R) and Epub Ahead of Print, In‐Process & Other Non‐Indexed Citations, Daily and Versions(R) <1946 to February 07, 2020>**

Search Date: 10 February 2020

‐‐‐‐‐‐‐‐‐‐‐‐‐‐‐‐‐‐‐‐‐‐‐‐‐‐‐‐‐‐‐‐‐‐‐‐‐‐‐‐‐‐‐‐‐‐‐‐‐‐‐‐‐‐‐‐‐‐‐‐‐‐‐‐‐‐‐‐‐‐‐‐‐‐‐‐‐‐‐‐

1 vulnerable populations/(10018)

2 poverty areas/(5952)

3 ((deprived or destitute? or impoverished or low income or marginalised or marginalized or needy or poverty or vulnerable) adj2 (adolesc$ or child$ or famil$ or men or people or youth? or women)).tw,kf. (15485)

4 homeless persons/(7479)

5 homeless youth/(1256)

6 runaway behavior/(311)

7 (homeless$ or runaway?).tw,kf. (11735)

8 (temporar$ adj2 (accommodat$ or home? or hous$)).tw,kf. (324)

9 ((based or housed or residen$ or temporar$) adj2 shelter?).tw,kf. (367)

10 or/1‐9 (43949)

11 exp program evaluation/(74262)

12 (effectiveness or initiative? or prevent$ or program$ or reduc$ or strateg$ or treatment?).tw. (8639907)

13 or/11‐12 (8662128)

14 MEDLINE.ab. (112073)

15 systematic review.tw. (146319)

16 meta analysis.pt. (110858)

17 randomized controlled trial.pt,kf. (503446)

18 controlled clinical trial.pt,kf. (93772)

19 pragmatic clinical trial.pt,kf. (1355)

20 controlled before‐after studies/(479)

21 interrupted time series analysis/(777)

22 controlled before‐after studies/(479)

23 (randomised or randomized).ab,kf. (564420)

24 placebo.ab. (205083)

25 clinical trials as topic/(190100)

26 randomly.ab. (326872)

27 trial.ti. (212932)

28 (before adj2 after adj5 (design$ or study or trial)).tw,kf. (10410)

29 (interrupt$ adj2 time series).tw,kf. (2932)

30 intervention?.ti. (131223)

31 ((multicenter or multicentre or multi center or multi centre) adj5 (design$ or study or trial)).ti,kf. (39530)

32 ((preintervention? or pre intervention? or postintervention? or post intervention?) adj5 (study or trial)).tw,kf. (1820)

33 (pre adj2 post adj2 (design$ or method$ or study or trial)).tw,kf. (6576)

34 ((pre test or pretest or (posttest or post test)) adj2 (design$ or method$ or study or trial)).tw,kf. (4987)

35 ((quasi experimental or quasiexperimental or quasi randomi$ or quasirandomi$) adj2 (design$ or method$ or study or trial)).tw,kf. (10323)

36 (repeated measures adj2 (design$ or method$ or study or trial)).tw,kf. (6003)

37 (time adj2 series adj (design? or study)).tw,kf. (1789)

38 (time points adj3 (month$ or more or multiple or three)).ab. (10152)

39 *economics/(10660)

40 exp *"Costs and Cost Analysis"/(68872)

41 economics, nursing/(3996)

42 economics, medical/(9054)

43 economics, pharmaceutical/(2913)

44 exp economics, hospital/(24214)

45 economics, dental/(1910)

46 exp "Fees and Charges"/(30123)

47 exp budgets/(13622)

48 ((budget$ or economic$ or cost or costs or costly or costing or expenditure or expenditures or expense or expenses or financial or finance or finances or financed) adj6 (analys$ or analyz$ or effect$ or evaluat$ or impact$)).ti,kf. (60202)

49 ((budget$ or economic$ or cost or costs or costly or costing or price or prices or pricing or pharmacoeconomic$ or pharmaco‐economic$ or expenditure or expenditures or expense or expenses or financial or finance or finances or financed) adj6 (analys$ or analyz$ or effect$ or evaluat$ or impact$)).ab./freq=2 (63169)

50 (cost$ adj2 (effective$ or utilit$ or benefit$ or minimi$ or analy$ or outcome or outcomes)).ab,kf. (156692)

51 (value adj2 (money or monetary)).tw,kf. (2318)

52 exp models, economic/(14702)

53 economic model$.ab,kf. (3214)

54 markov chains/(13977)

55 markov.tw,kf. (21720)

56 monte carlo method/(27767)

57 monte carlo.tw,kf. (47846)

58 exp decision theory/(11804)

59 (decision$ adj2 (tree$ or analy$ or model$)).tw,kf. (22896)

60 or/14‐59 (1990223)

61 animals/not (humans/and animals/) (4638842)

62 60 not 61 (1867106)

63 10 and 13 and 62 (4503)

‐‐‐‐‐‐‐‐‐‐‐‐‐‐‐‐‐‐‐‐‐‐‐‐‐‐‐‐‐‐‐‐‐‐‐‐‐‐‐‐‐‐‐‐‐‐‐‐‐‐‐‐‐‐‐‐‐‐‐‐‐‐‐‐‐‐‐‐‐‐‐‐‐‐‐‐‐‐‐‐

**Database: EBM Reviews ‐ Cochrane Central Register of Controlled Trials <January 2020>**

Search Date: 10 February 2020

‐‐‐‐‐‐‐‐‐‐‐‐‐‐‐‐‐‐‐‐‐‐‐‐‐‐‐‐‐‐‐‐‐‐‐‐‐‐‐‐‐‐‐‐‐‐‐‐‐‐‐‐‐‐‐‐‐‐‐‐‐‐‐‐‐‐‐‐‐‐‐‐‐‐‐‐‐‐‐‐

1 vulnerable populations/(271)

2 poverty areas/(269)

3 ((deprived or destitute? or impoverished or low income or marginalised or marginalized or needy or poverty or vulnerable) adj2 (adolesc$ or child$ or famil$ or men or people or youth? or women)).tw. (2265)

4 homeless persons/(314)

5 homeless youth/(31)

6 runaway behavior/(3)

7 (homeless$ or runaway?).tw. (901)

8 (temporar$ adj2 (accommodat$ or home? or hous$)).tw. (20)

9 ((based or housed or residen$ or temporar$) adj2 shelter?).tw. (40)

10 or/1‐9 (3660)

11 exp program evaluation/(5930)

12 (effectiveness or initiative? or prevent$ or program$ or reduc$ or strateg$ or treatment?).tw. (1012478)

13 or/11‐12 (1013092)

14 10 and 13 (2982)

‐‐‐‐‐‐‐‐‐‐‐‐‐‐‐‐‐‐‐‐‐‐‐‐‐‐‐‐‐‐‐‐‐‐‐‐‐‐‐‐‐‐‐‐‐‐‐‐‐‐‐‐‐‐‐‐‐‐‐‐‐‐‐‐‐‐‐‐‐‐‐‐‐‐‐‐‐‐‐‐

**Database: EBM Reviews ‐ Cochrane Database of Systematic Reviews <2005 to February 4, 2020>, EBM Reviews ‐ Database of Abstracts of Reviews of Effects <1st Quarter 2016>, EBM Reviews ‐ Health Technology Assessment <4th Quarter 2016>, EBM Reviews ‐ NHS Economic Evaluation Database <1st Quarter 2016>**

Search Date: 10 February 2020

‐‐‐‐‐‐‐‐‐‐‐‐‐‐‐‐‐‐‐‐‐‐‐‐‐‐‐‐‐‐‐‐‐‐‐‐‐‐‐‐‐‐‐‐‐‐‐‐‐‐‐‐‐‐‐‐‐‐‐‐‐‐‐‐‐‐‐‐‐‐‐‐‐‐‐‐‐‐‐‐

1 ((deprived or destitute? or impoverished or low income or marginalised or marginalized or needy or poverty or vulnerable) adj2 (adolesc$ or child$ or famil$ or men or people or youth? or women)).tw. (275)

2 (homeless$ or runaway?).tw. (152)

3 (temporar$ adj2 (accommodat$ or home? or hous$)).tw. (9)

4 ((based or housed or residen$ or temporar$) adj2 shelter?).tw. (8)

5 or/1‐4 (419)

6 (effectiveness or initiative? or prevent$ or program$ or reduc$ or strateg$ or treatment?).tw. (56120)

7 5 and 6 (397)

8 remove duplicates from 7 (397)

‐‐‐‐‐‐‐‐‐‐‐‐‐‐‐‐‐‐‐‐‐‐‐‐‐‐‐‐‐‐‐‐‐‐‐‐‐‐‐‐‐‐‐‐‐‐‐‐‐‐‐‐‐‐‐‐‐‐‐‐‐‐‐‐‐‐‐‐‐‐‐‐‐‐‐‐‐‐‐

**Database: Embase <1974 to 2020 February 07>**

Search Date: 10 February 2020

‐‐‐‐‐‐‐‐‐‐‐‐‐‐‐‐‐‐‐‐‐‐‐‐‐‐‐‐‐‐‐‐‐‐‐‐‐‐‐‐‐‐‐‐‐‐‐‐‐‐‐‐‐‐‐‐‐‐‐‐‐‐‐‐‐‐‐‐‐‐‐‐‐‐‐‐‐‐‐‐

1 vulnerable population/(15963)

2 ((deprived or destitute? or impoverished or low income or marginalised or marginalized or needy or poverty or vulnerable) adj2 (adolesc$ or child$ or famil$ or men or people or youth? or women)).tw. (17102)

3 exp homeless person/(1817)

4 runaway behavior/(354)

5 (homeless$ or runaway?).tw. (13681)

6 (temporar$ adj2 (accommodat$ or home? or hous$)).tw. (414)

7 ((based or housed or residen$ or temporar$) adj2 shelter?).tw. (418)

8 or/1‐7 (46024)

9 exp program evaluation/(23164)

10 (effectiveness or initiative? or prevent? or prevention or program? or programme? or reduce or reducing or reduction or strategies or strategy or treatment?).tw. (9782726)

11 or/9‐10 (9787585)

12 meta‐analys$.tw. (209138)

13 systematic review.tw. (181593)

14 randomized controlled trial/(589023)

15 crossover procedure/(62044)

16 double‐blind procedure/(169219)

17 (randomi?ed or randomly).tw. (1210412)

18 time series analysis/(25145)

19 (crossover$ or cross‐over$).tw. (104516)

20 placebo.ab. (292599)

21 (doubl$ adj blind$).tw. (206008)

22 assign$.ab. (379064)

23 allocat$.ab. (145168)

24 (before adj2 after adj3 (design$ or study or trial)).tw. (10203)

25 (interrupt$ adj2 time series).tw. (3655)

26 intervention?.ti. (172602)

27 ((multicenter or multicentre or multi center or multi centre) adj3 (design$ or study or trial)).ti. (53468)

28 ((preintervention? or pre intervention? or postintervention? or post intervention?) adj5 (study or trial)).tw. (2653)

29 (pre adj2 post adj2 (design$ or method$ or study or trial)).tw. (9708)

30 ((pre test or pretest or (posttest or post test)) adj2 (design$ or method$ or study or trial)).tw. (6391)

31 ((quasi experimental or quasiexperimental or quasi randomi$ or quasirandomi$) adj2 (design$ or method$ or study or trial)).tw. (12189)

32 (repeated measures adj2 (design$ or method$ or study or trial)).tw. (6957)

33 (time adj2 series adj (design? or study)).tw. (2033)

34 (time points adj3 (month$ or more or multiple or three)).ab. (15643)

35 *health economics/(15308)

36 *pharmacoeconomics/(4942)

37 ((budget$ or economic$ or cost or costs or costly or costing or expenditure or expenditures or expense or expenses or financial or finance or finances or financed) adj6 (analys$ or analyz$ or effect$ or evaluat$ or impact$)).ti. (78778)

38 ((budget$ or economic$ or cost or costs or costly or costing or price or prices or pricing or pharmacoeconomic$ or pharmaco‐economic$ or expenditure or expenditures or expense or expenses or financial or finance or finances or financed) adj6 (analys$ or analyz$ or effect$ or evaluat$ or impact$)).ab./freq=2 (97039)

39 (cost$ adj2 (effective$ or utilit$ or benefit$ or minimi$ or analy$ or outcome or outcomes)).ab. (215617)

40 (value adj2 (money or monetary)).tw. (3183)

41 *economic model/(673)

42 economic model$.ab. (4443)

43 *markov chain/(863)

44 markov.tw. (27260)

45 *monte carlo method/(6477)

46 [monte carlo.tw,kf.] (0)

47 *decision theory/(511)

48 (decision$ adj2 (tree$ or analy$ or model$)).tw. (31869)

49 or/12‐48 (2458233)

50 (exp animal/or animal.hw. or nonhuman/) not (exp human/or human cell/or (human or humans).ti.) (6314639)

51 49 not 50 (2199430)

52 8 and 11 and 51 (4391)

‐‐‐‐‐‐‐‐‐‐‐‐‐‐‐‐‐‐‐‐‐‐‐‐‐‐‐‐‐‐‐‐‐‐‐‐‐‐‐‐‐‐‐‐‐‐‐‐‐‐‐‐‐‐‐‐‐‐‐‐‐‐‐‐‐‐‐‐‐‐‐‐‐‐‐‐‐‐‐‐

**Database: EBSCO CINAHL**

Search Date: 10 February 2020

‐‐‐‐‐‐‐‐‐‐‐‐‐‐‐‐‐‐‐‐‐‐‐‐‐‐‐‐‐‐‐‐‐‐‐‐‐‐‐‐‐‐‐‐‐‐‐‐‐‐‐‐‐‐‐‐‐‐‐‐‐‐‐‐‐‐‐‐‐‐‐‐‐‐‐‐‐‐‐‐

S49 S7 AND S10 AND S48 Limiters ‐ Exclude MEDLINE records 1,857

S48 S11 OR S12 OR S13 OR S14 OR S15 OR S16 OR S17 OR S18 OR S19 OR S20 OR S21 OR S22 OR S23 OR S24 OR S25 OR S26 OR S27 OR S28 OR S29 OR S30 OR S31 OR S32 OR S33 OR S34 OR S35 OR S36 OR S37 OR S38 OR S39 OR S40 OR S41 OR S42 OR S43 OR S44 OR S45 OR S46 OR S47 1,307,171

S47 (decision* N2 (tree* or analy* or model*)) 9,163

S46 monte carlo 2,617

S45 markov 3,313

S44 economic model* 2,385

S43 (value N2 (money or monetary)) 1,140

S42 AB (cost* N2 (effective* or utilit* or benefit* or minimi* or analy* or outcome or outcomes)) 42,798

S41 ((budget* or economic* or cost or costs or costly or costing or price or prices or pricing or pharmacoeconomic* or pharmaco‐economic* or expenditure or expenditures or expense or expenses or financial or finance or finances or financed) N6 (analys* or analyz* or effect* or evaluat* or impact*)) 101,480

S40 (MH "Fees and Charges+") 13,772

S39 (MH "Economics, Pharmaceutical") 2,103

S38 (MH "Costs and Cost Analysis+") 109,160

S37 AB (time points N3 (month* or more or multiple or three)) 4,967

S36 (time N2 series N (design* or study)) 12

S35 (repeated measures N2 (design* or method* or study or trial)) 4,705

S34 ((quasi experimental or quasiexperimental or quasi randomi* or quasirandomi*) N2 (design* or method* or study or trial)) 16,603

S33 ((pre test or pretest or (posttest or post test)) N2 (design* or method* or study or trial)) 42,299

S32 (pre N2 post N2 (design* or method* or study or trial)) 5,114

S31 ((preintervention* or pre intervention* or postintervention* or post intervention*) N3 (study or trial)) 1,676

S30 ((multicenter or multicentre or multi center or multi centre) N5 (design* or study or trial)) 186,123

S29 TI (intervention* OR trial) 177,525

S28 (interrupt* N2 time series) 1,672

S27 (before N2 after N5 (design* or study or trial)) 6,191

S26 allocat* 41,255

S25 blind* 99,788

S24 placebo* 59,460

S23 (crossover or cross‐over or cross over) 25,344

S22 (randomi* OR randomly) 295,055

S21 MH "Pretest‐Posttest Design+" 40,350

S20 MH "Prospective Studies" 407,317

S19 MH "Concurrent Prospective Studies" 183

S18 MH "Comparative Studies" 239,994

S17 MH "Quasi‐Experimental Studies+" 14,154

S16 MH "Control Group" 11,288

S15 MH "Clinical Trials+" 273,962

S14 MH "Crossover Design" 17,943

S13 MH "Random Assignment") 57,406

S12 systematic review 122,874

S11 meta analys* 73,739

S10 S8 OR S9 2,240,601

S9 (effectiveness or initiative* or prevent* or program* or reduc* or strateg* or treatment*) 2,240,601

S8 (MH "Program Evaluation") 37,851

S7 S1 OR S2 OR S3 OR S4 OR S5 OR S6 27,699

S6 ((based or housed or residen* or temporar*) N2 shelter*) 278

S5 (temporar* N2 (accommodat* or home* or hous*)) 194

S4 (homeless* or runaway*) 10,701

S3 (MH "Homeless Persons") 5,130

S2 ((deprived or destitute* or impoverished or low income or marginalised or marginalized or needy or poverty or vulnerable) N2 (adolesc* or child* or famil* or men or people or youth* or women)) 14,358

S1 (MH "Poverty Areas") 3,070

‐‐‐‐‐‐‐‐‐‐‐‐‐‐‐‐‐‐‐‐‐‐‐‐‐‐‐‐‐‐‐‐‐‐‐‐‐‐‐‐‐‐‐‐‐‐‐‐‐‐‐‐‐‐‐‐‐‐‐‐‐‐‐‐‐‐‐‐‐‐‐‐‐‐‐‐‐‐‐‐

**Database: EBSCO PsycINFO**

Search Date: 10 February 2020

‐‐‐‐‐‐‐‐‐‐‐‐‐‐‐‐‐‐‐‐‐‐‐‐‐‐‐‐‐‐‐‐‐‐‐‐‐‐‐‐‐‐‐‐‐‐‐‐‐‐‐‐‐‐‐‐‐‐‐‐‐‐‐‐‐‐‐‐‐‐‐‐‐‐‐‐‐‐‐‐

S22 S1 OR S2 OR S3 OR S4 OR S5 OR S6 OR S7 OR S8 OR S9 OR S10 OR S11 OR S12 OR S13 OR S14 OR S15 OR S16 OR S17 OR S18 OR S19 OR S20 OR S21 245,175

S21 TI ((decision* N2 (tree* or analy* or model*))) OR AB ((decision* N2 (tree* or analy* or model*))) 12,001

S20 TI monte carlo OR AB monte carlo 4,493

S19 TI markov OR AB markov 3,639

S18 TI economic model* OR AB economic model* 1,485

S17 AB (cost* N1 (effective* or utilit* or benefit* or minimi* or analy* or outcome or outcomes)) 23,784

S16 TI (((budget* or economic* or cost or costs or costly or costing or pharmacoeconomic* or pharmaco‐economic* or expenditure or expenditures or expense or expenses or financial or finances or financed) N1 (analys* or analyz* or effect* or evaluat* or impact*))) OR AB (((budget* or economic* or cost or costs or costly or costing or pharmacoeconomic* or pharmaco‐economic* or expenditure or expenditures or expense or expenses or financial or finances or financed) N1 (analys* or analyz* or effect* or evaluat* or impact*))) 26,351

S15 AB (time points N3 (month* or more or multiple or three)) 5,343

S14 (time N2 series N (design* or study)) 6

S13 TI ((repeated measures N2 (design* or method* or study or trial))) OR AB ((repeated measures N2 (design* or method* or study or trial))) 4,214

S12 TI (((quasi experimental or quasiexperimental or quasi randomi* or quasirandomi*) N2 (design* or method* or study or trial))) OR AB (((quasi experimental or quasiexperimental or quasi randomi* or quasirandomi*) N2 (design* or method* or study or trial))) 9,693

S11 TI (((pre test or pretest or (posttest or post test)) N2 (design* or method* or study or trial))) OR AB (((pre test or pretest or (posttest or post test)) N2 (design* or method* or study or trial))) Display

S10 TI ((pre N2 post N2 (design* or method* or study or trial))) OR AB ((pre N2 post N2 (design* or method* or study or trial))) Display

S9 ((preintervention* or pre intervention* or postintervention* or post intervention*) N2 (study or trial)) Display

S8 TI (((multicenter or multicentre or multi center or multi centre) N3 (design* or study or trial))) OR AB (((multicenter or multicentre or multi center or multi centre) N3 (design* or study or trial))) Display

S7 (interrupt* N2 time series) Display

S6 (before N2 after N5 (design* or study or trial)) Display

S5 TI ((double blind* OR double‐blind* OR single blind* OR single‐blind*) OR AB (double blind* OR double‐blind* OR single blind* OR single‐blind*) N2 (design* or study or trial)) Display

S4 TI ((crossover or cross‐over or cross over)) OR AB ((crossover or cross‐over or cross over)) Display

S3 TI (((randomi* OR randomly) N2 (allocat* OR assign* OR blind* OR control* OR design OR study OR trial)) OR AB (((randomi* OR randomly) N2 (allocat* OR assign* OR blind* OR control* OR design OR study OR trial)) Display

S2 TX systematic review Display

S1 TX meta analys* Display

‐‐‐‐‐‐‐‐‐‐‐‐‐‐‐‐‐‐‐‐‐‐‐‐‐‐‐‐‐‐‐‐‐‐‐‐‐‐‐‐‐‐‐‐‐‐‐‐‐‐‐‐‐‐‐‐‐‐‐‐‐‐‐‐‐‐‐‐‐‐‐‐‐‐‐‐‐‐‐‐

**Database: Epistemonikos**

‐‐‐‐‐‐‐‐‐‐‐‐‐‐‐‐‐‐‐‐‐‐‐‐‐‐‐‐‐‐‐‐‐‐‐‐‐‐‐‐‐‐‐‐‐‐‐‐‐‐‐‐‐‐‐‐‐‐‐‐‐‐‐‐‐‐‐‐‐‐‐‐‐‐‐‐‐‐‐‐

TI,AB (destitute or homeless or impoverished or low income or marginalised or marginalized or needy or temporary accommodation* or temporary housing or temporary shelter)

AND

TI,AB (effectiveness or initiative* or prevent* or program* or reduc* or strateg* or treatment*)
