# Corrigendum

**DOI:** 10.1002/cl2.1238

**Published:** 2022-04-12

The authors of Moledina et al. (2021) have supplied the following correction to their article.

Co‐authors “Ginetta Salvalaggio, Gary Bloch, David Ponka, Tim Aubrey, Claire Kendall” have been added.

Correct author group and affiliations are:

Aliza Moledina^1^, Olivia Magwood^2^, Eric Agbata^3^, Jui‐Hsia Hung^4^, Ammar Saad^5^, Kednapa Thavorn^6^, Ginetta Salvalaggio^8^, Gary Bloch^9^, David Ponka^7^, Tim Aubrey^10^, Claire Kendall^7^, Kevin Pottie^7^

^1^Faculty of Medicine, University of Ottawa, Ottawa, Canada

^2^C.T. Lamont Primary Health Care Research Centre, Bruyere Research Institute, Ottawa, Canada

^3^School of Epidemiology, Public Health and Preventive Medicine, Bruyere Research Institute, Ottawa, Canada

^4^School of Epidemiology and Public Health, Faculty of Medicine, University of Ottawa, Ottawa, Canada

^5^Department of Epidemiology, C.T. Lamont Primary Care Research Centre, Bruyere Research Institute, University of Ottawa, Ottawa, Canada

^6^Clinical Epidemiology Program, Ottawa Hospital Research Institute, Ottawa, Canada

^7^Family Medicine, University of Ottawa, Ottawa, Canada

^8^University of Alberta, Alberta, Canada

^9^Department of Family Medicine, University of Toronto, Toronto, Canada

^10^School of Psychology & Centre for Research on Educational and Community Services, University of Ottawa, Ottawa, Canada

The original version is also corrected and can be found on the below link: https://doi.org/10.1002/cl2.1154. The authors apologise for the error.

## References

[cl21238-bib-0001] Moledina, A. , Magwood, O. , Agbata, E. , Hung, J.‐H. , Saad, A. , Thavorn, K. , Salvalaggio, G. , Bloch, G. , Ponka, D. , Aubrey, T. , Kendall, C. , & Pottie, K. (2021). A comprehensive review of prioritised interventions to improve the health and wellbeing of persons with lived experience of homelessness. Campbell Systematic Reviews, 17, e1154. 10.1002/cl2.1154 PMC8356292 37131928

